# Exploring Simple Drug Scaffolds from the Generated Database Chemical Space Reveals a Chiral Bicyclic Azepane with Potent Neuropharmacology

**DOI:** 10.1021/acs.jmedchem.4c02549

**Published:** 2025-04-24

**Authors:** Aline Carrel, Adonis Yiannakas, Jaap-Jan Roukens, Ines Reynoso-Moreno, Markus Orsi, Amol Thakkar, Josep Arus-Pous, Daniele Pellegata, Jürg Gertsch, Jean-Louis Reymond

**Affiliations:** a Department of Chemistry, Biochemistry and Pharmaceutical Sciences, University of Bern, Freiestrasse 3, Bern 3012, Switzerland; b Institute of Biochemistry and Molecular Medicine, University of Bern, Gertrud-Woker Strasse 5, Bern 3012, Switzerland

## Abstract

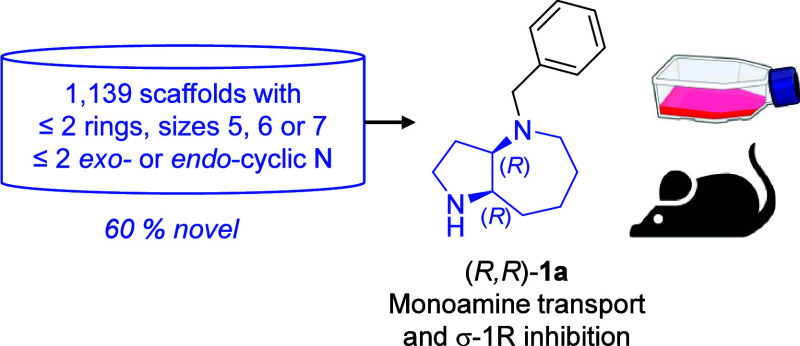

To assess how much structural diversity remains unexploited in simple drug scaffolds, we investigated ring systems functionalized with amine handles. Starting from the ring systems database GDB-4c, we enumerated 1139 possible amines and diamines with up to two five-, six-, or seven-membered rings. From the 680 cases not listed in PubChem, we synthesized several unprecedented *cis*- and *trans*-fused azepanes and tested possible targets predicted using the polypharmacology browser PPB2. From this screening campaign, an *N*-benzylated azepane emerged as a potent inhibitor of monoamine transporters with some selectivity toward norepinephrine (NET, SLC6A2) and dopamine transporter (DAT, SLC6A3) inhibition (IC_50_ < 100 nM) in combination with σ-1R inhibition (IC_50_ ≈ 110 nM). The *in vitro* profile, favorable pharmacokinetic properties, and preliminary behavioral and metabolomic effects in mice suggest a potential of *N*-benzylated bicyclic azepanes to target neuropsychiatric disorders. These experiments highlight the potential of simple but still unexplored scaffolds for drug discovery.

## Introduction

When considering the vastness of chemical space available for drug design,^1−5^ the currently dominant view is that, although only a limited number of building blocks is available, sufficient innovation can be obtained by combining these building blocks using known chemistry to form diverse screening compounds, as realized in billion sized libraries such as REAL^6,7^ or DNA-encoded libraries.^8−11^ Due to their combinatorial assembly, however, these molecules tend to populate the upper limits of Lipinski’s “rule of 5” chemical space.^12^ By exploring the small molecule chemical space systematically with the generated databases (GDBs), which enumerate all possible molecules up to 11, 13, and 17 atoms of C, N, O, S and halogens following simple rules of chemical stability and synthetic feasibility,^13−18^ it is however apparent that a vast and unexploited reservoir of novelty remains to be discovered well below the “rule of 5” limit at the level of the building blocks themselves, which may feature novel molecular frameworks and fragments.^19,20^ Due to their small size, many GDB molecules are even compatible with the more restricted criteria for CNS drug discovery.^21^ However, most new scaffolds derived from the GDBs contain three or more rings and are therefore quite complex and challenging to synthesize.^22−25^ Similarly, small scaffolds designed as isosteric replacements for benzene rings or piperazine feature nontrivial, strained spiro- and bicyclic systems.^26−31^ Scaffolds from diversity-oriented synthesis (DOS) and activity-directed synthesis (ADS) also contain polycyclic structures.^32−35^

**Figure 1:**
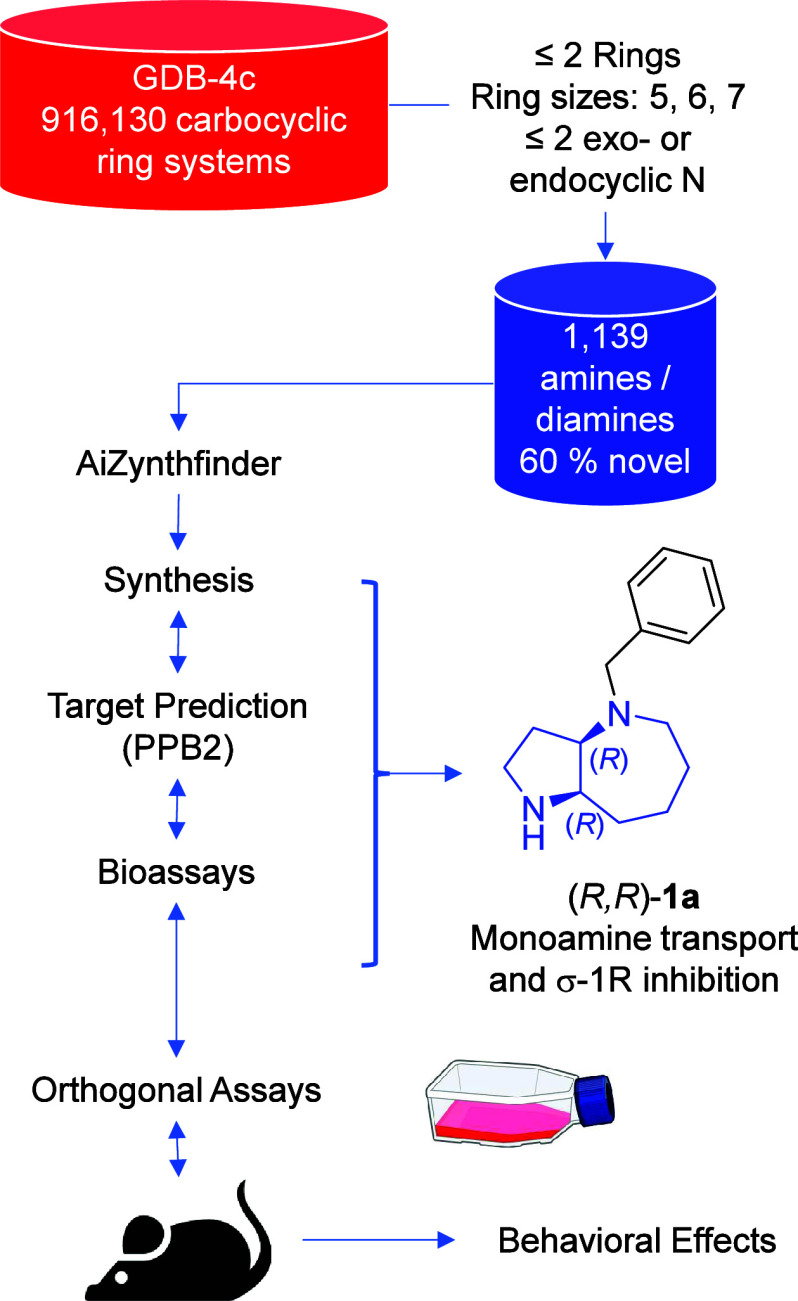
Discovery workflow leading from the GDB-4c database to bicyclic azepane (*R,R*)-**1a** as a potent monoamine transport and σ-1R inhibitor.

Here, we asked the question of whether original scaffolds might still be found featuring only straightforward structural elements by investigating mono- and bicyclic ring systems containing only five-, six-, or seven-membered rings, which are generally unstrained and in principle readily accessible. We focused on amine and diamine derivatives related to piperazine and aminocyclohexane, which are versatile scaffolds used ubiquitously for drug design.^36,37^ As discussed below, an enumeration approach, starting from the ring systems database GDB4c,^38^ revealed that many of these relatively simple scaffolds are indeed novel and should be synthetically accessible, as suggested by the retrosynthetic program AiZynthfinder.^39−41^ We investigated *cis*- and *trans*-fused azepanes accessible via Beckmann rearrangement of cyclohexanone oximes, from which we discovered the *N*-benzylated (*R,R*)-**1a**, a small molecule within the range of GDB-17 and acting as a nanomolar norepinephrine (NE) transporter (NET, SLC6A2) inhibitor also acting on the dopamine (DA) transporter (DAT, SLC6A3), the serotonine (5-HT) transporter (SERT, SLC6A4) and the σ-1 receptor. Orthogonal cellular assays using liquid chromatography electrospray ionization mass spectrometry (LC-ESI-MS/MS) confirmed the stereoselective uptake inhibition of NE and DA by (*R,R*)-**1a** in PC-12 cells and were compared to clinically used NET/DAT and SERT uptake inhibitors. The first pharmacokinetic data (i.v. and p.o.) further supported the outstanding brain penetrance of (*R,R*)-**1a**. The potent and unusual *in vivo* pharmacology of this compound suggests that it might find application for addressing unmet medical needs in neurological diseases. These experiments highlight the potential of simple but still unexplored scaffolds for drug discovery (Figure 1).

## Results and Discussion

### Enumeration, Novelty, and Retrosynthetic Analysis

**Figure 2:**
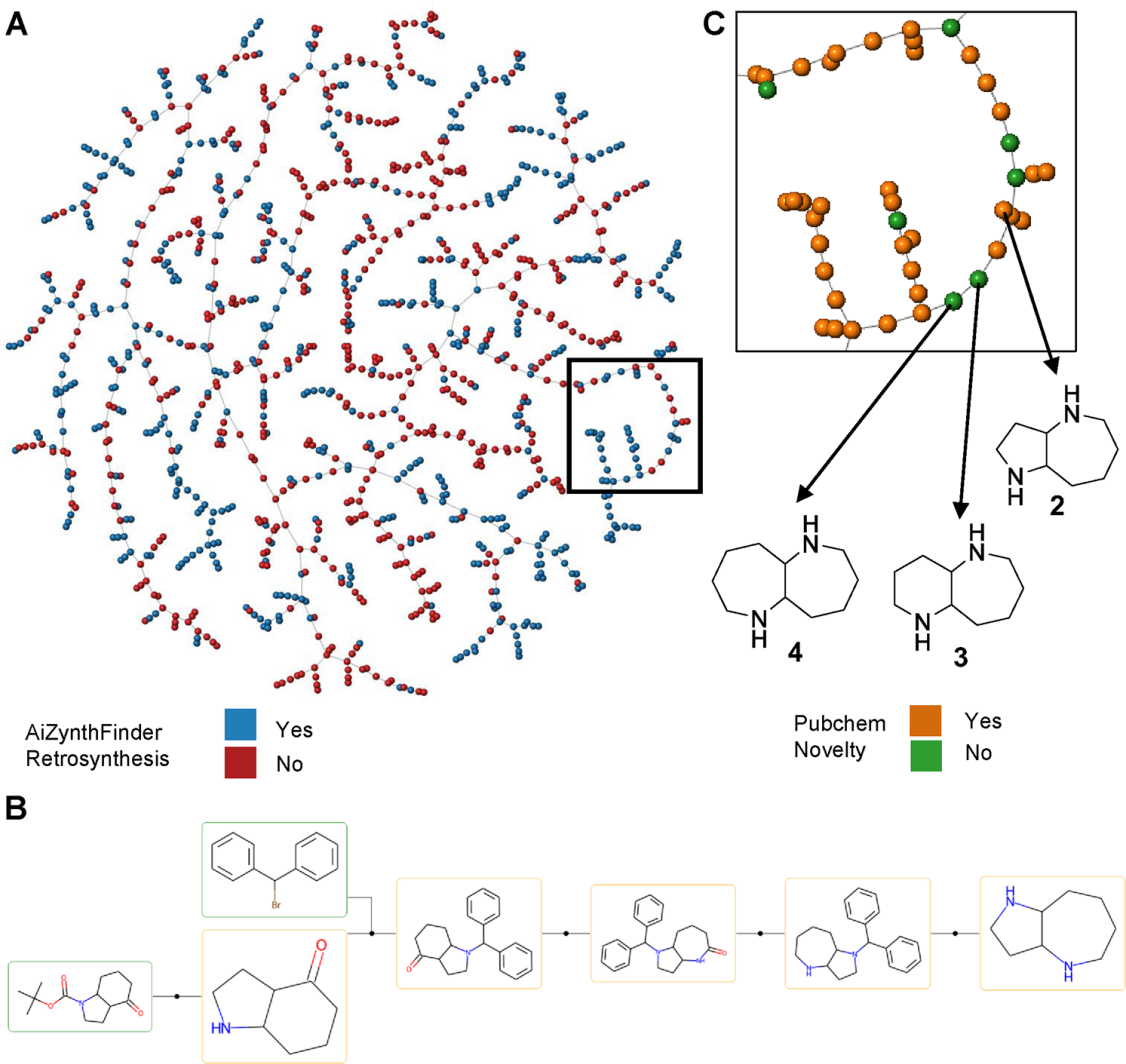
Data set overview and selection of fused azepanes. (A) TMAP colored by AiZynthfinder retrosynthesis success (yes, no) and close-up view colored by PubChem occurrence (yes, no). the interactive TMAP is accessible at https://tm.gdb.tools/map4/MAP4_GDBDiamines_NETI/, each molecule is linked to the set of retroynthetic routes proposed by Aizynthfinder. (B) Aizynthfinder retrosynthesis of **2**. (C) structure of the (5,7)-, (6,7)-, and (7,7)-diamines **2**, **3**, and **4**.

To obtain a list of all possible nonaromatic mono- and bicyclic amine and diamine scaffolds with ring sizes 5, 6, or 7, we extracted the corresponding 24 ring systems from the GDB4c database (Figure S1). For each ring system, we enumerated all possible combinations of single and double C → N and C–H → C–NH_2_ exchanges, except for hydrazines (N–N), aminals (N–C–N), and quaternary ammonium ions. This enumeration produced 1139 scaffolds, not considering stereochemistry, with an average molecular weight of 157 ± 22 Da. These scaffolds comprised monocyclic as well as spiro, fused and bridged bicyclic systems with one or two nitrogen atoms occurring as primary, secondary, or tertiary amines. A comparative analysis showed that 680 (60%) of these scaffolds were not listed in PubChem and were labeled here as novel. Novelty was almost entirely confined to bicyclic scaffolds containing at least one 7-membered ring, in line with the much lower frequency of 7-membered rings compared to 5- and 6-membered rings in known molecules. Note that the number of enumerated scaffolds was particularly large for those containing either two primary amines or one secondary and one primary amine (Table 1). An interactive TMAP^42^ color-coded by novelty, ring and amine types, stereochemical complexity, and synthetic accessibility (see below) provided an overview of the available diversity in the data set (Figure 2A).

**Table 1:** Number of Novel/Enumerated Scaffolds Amine and Diamine Scaffolds^a^

**N-types**^b^	**N**	**NH**	**NH**_**2**_	N/N	**N/NH**	**N/NH**_**2**_	**NH/NH**	**NH/NH**_**2**_	**NH**_**2**_**/NH**_**2**_	**total**
ring sizes^c^										
5	0/0	0/1	0/1	0/0	0/0	0/0	0/0	0/1	0/2	0/5
6	0/0	0/1	0/1	0/0	0/0	0/0	0/1	0/2	0/3	0/8
7	0/0	0/1	0/1	0/0	0/0	0/0	0/1	0/2	0/3	0/8
5, 5	0/2	0/6	0/8	0/0	0/0	0/4	0/6	3/20	12/25	15/71
5, 6	0/2	0/13	0/15	0/0	0/2	0/9	0/15	19/58	39/52	58/166
5, 7	0/2	0/14	7/16	0/0	0/4	5/11	0/22	70/75	64/64	146/208
6, 6	0/3	0/9	0/12	0/1	0/2	0/8	0/17	21/44	24/46	45/142
6, 7	0/3	0/19	9/22	0/1	0/7	13/17	9/35	107/111	89/93	227/308
7, 7	0/3	3/12	9/15	0/1	2/5	12/12	18/30	75/75	70/70	189/223
ring types^d^										
monocyclic	0/0	0/3	0/3	0/0	0/0	0/0	0/2	0/5	0/8	0/21
spirocyclic	0/0	0/24	8/24	0/0	0/0	0/0	6/42	94/121	89/93	197/304
fused	0/6	2/21	6/27	0/0	1/9	13/27	9/46	98/131	98/121	227/388
bridged	0/9	1/28	11/37	0/3	1/11	17/34	12/37	103/131	111/136	256/426
total	0/15	3/76	25/91	0/3	2/20	30/61	27/127	295/388	298/358	680/1139

a The number of scaffolds is given for each category as novel/total, novel = does not appear in PubChem. Total = as enumerated in this work from GDB-4c.

b N-types given as *N* = tertiary amine, NH = secondary amine, NH_2_ = primary amine.

c Ring sizes for monocyclic or bicyclic ring systems.

d Ring types for monocyclic and bicyclic ring systems.

A retrosynthetic analysis of the data set using the computer-assisted synthesis planning (CASP) program AiZynthfinder^39−41^ suggested that 573 molecules (50%) were accessible from commercial precursors, including many of the known scaffolds, which were commercially available as free or protected amines. Details of the AiZynthfinder retrosynthetic routes are accessible for each diamine via the interactive version of the TMAP in Figure 2A. Scaffolds containing primary amines, which, as noted above, featured the largest diversity and novelty, led to rather challenging retrosyntheses, in part reflecting their stereochemical complexity. On the other hand, closer inspection of the retrosynthetic routes generated by AiZynthfinder showed that azepanes (7-membered azacycles) were often predicted to be accessible by Beckmann rearrangement from the parent cyclohexanone oximes. One striking example was the fused azepane, (5,7)-diamine **2**, which was only documented with a single Scifinder occurrence relating to a patent application without stereochemistry assignment or synthesis (Figure 2B). We set out to explore the synthesis of this scaffold as well as of the corresponding and unknown (6,7)- and (7,7)-diamines **3** and **4** (Figure 2C).

### Synthesis of Fused Azepanes

**Scheme 1:**
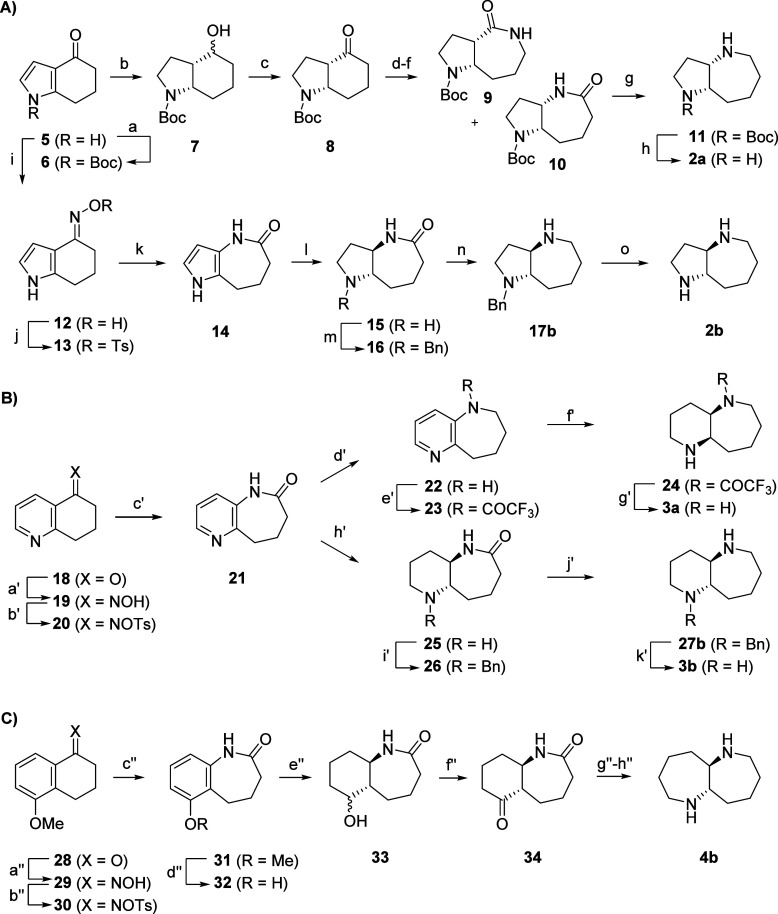
Synthesis of Fused Azepanes (A) Conditions: (a) Boc_2_O, DIPEA, DMAP, ACN, 22 °C, 24 h → **6** (quant.); b) PtO_2_, H_2_ (10 bar), 2 equiv AcOH, *i*PrOH, 50 °C, 6 h → **7** (71%); (c) DMP, DCM, 22 °C, 30 min → **8** (quant.); (d) NH_2_OH·HCl, pyr, 22 °C, 2 h; (e) *p*-TsCl, pyr, 22 °C, 2 h; (f) KOAc, EtOH/H_2_O, 100 °C, 16 h → **9** and **10** (57% over three steps, **10**:**9**, 1.3:1); (g) LiAlH_4_, THF, 22 °C, 4 h → **11** (46%); (h) TFA, DCM, 22 °C, 2 h → **2a** (89%); (i) NH_2_OH·HCl, pyr, 22 °C, 2 h → **12** (quant., *E*:*Z* 4:1); j) *p*-TsCl, pyr, 22 °C, 2 h → **13** (89%); (k) KOAc, EtOH/H_2_O, reflux, 12 h → **14** (71%); l) 10% Pd/C, H_2_ (25 bar), AcOH, 100 °C, 3 days → **15** (46%); (m) BnBr, K_2_CO_3_, MeOH, 22 °C, 2 h → **16** (95%), (n) LiAlH_4_, THF, reflux, 16 h → **17b** (73%); (o) Pd/C, H_2_ (1 bar), AcOH, MeOH, 24 h → **2b** (quant.); (B) conditions: (a′) NH_2_OH·HCl, NaOAc, MeOH/H_2_O, reflux, 4 h → **19** (98%); (b′) *p*-TsCl, KOH, acetone/H_2_O, reflux, 2 h → **20** (65%); (c′) KOAc, MeOH/H_2_O, reflux, 24 h → **21** (91%); (d′) LiAlH_4_, THF, reflux, 6 h → **22** (81%); (e′) TFAA, pyr, DCM, 22 °C, 2 h → **23** (90%); (f′) Rh/C, H_2_ (10 bar), *i*PrOH, 70 °C, 5 days → **24** (98%); (g′) LiOH, THF/H_2_O, reflux, 24 h → **3a** (78%); (h′) 10% Pd/C, H_2_ (20 bar), AcOH, 100 °C, 2 days → **25** (32%); (i′) BnBr, K_2_CO_3_, MeOH, 22 °C, 2 h → **26** (64%), (j′) LiAlH_4_, THF, 0 to 22 °C, 22 h → **27b** (79%); (k′) Pd/C, H_2_ (1 bar), AcOH, MeOH, 24 h → **3b** (quant.); (C) conditions: (a″) NH_2_OH·HCl, NaOH, EtOH, reflux, 2 h → **29** (quant.); (b″) *p*-TsCl, pyr, acetone/H_2_O, 22 °C, 24 h → **30** (quant.); (c″) AcOH, H_2_O, 70 °C, 24 h → **31** (75%); (d″) BBr_3_, DCM, −78 to 22 °C, 24 h → **32** (84%); (e″) Rh/C, H_2_ (20 bar), 2 equiv AcOH, *i*PrOH, 70 °C, 5 days → **33** (43%); (f″) DMP, DCM, 22 °C, 1 h → **34** (82%); (g″) H_2_SO_4_, NaN_3_, CHCl_3_, 0 to 22 °C, 24 h; (h″) LiAlH_4_, THF, 0 °C to reflux, 24 h → **4b** (43% over two steps).

The synthesis proposed by AiZynthfinder for the (5,7)-diamine **2** succeeded using both the *N*-diphenylmethyl protecting group suggested by the program and a simpler *N*-benzyl protecting group. Due to the high cost of the commercial precursor **8** identified by AiZynthfinder, we started from pyrrolocyclohexanone **5**, which was converted to **6** by Boc protection followed by hydrogenation of the pyrrole ring under mild conditions (PtO_2_, 10 bar H_2_, 2 equiv AcOH, *i*PrOH, 50 °C, 6 h) to form the *cis*-fused pyrrolidinocyclohexanol intermediate **7** and reoxidation to form ketone **8**. Oxime formation, tosylation, and Beckmann rearrangement then provided lactams **9** and **10** as a 1:1.3 mixture of regioisomers, reflecting the *Z*/*E* ratio of the oxime intermediate. Lactam **10** was separated from its regioisomer and reduced with LiAlH_4_ to the corresponding azepane **11**, which was deprotected to yield the *cis*-fused (5,7)-diamine **2a**, completing an overall 8-step sequence in 9% overall yield (Scheme 1A, first line, and Scheme S1).

By performing the Beckmann rearrangement on tosylate **13**, obtained from **5** via the separable *E-*oxime **12** (*E*-**12/***Z*-**12** 4:1), we obtained pyrrololactam **14**. In this case, hydrogenation required harsher conditions (25 bar H_2_, Pd/C, AcOH, 100 °C, 3 days) and gave access to the *trans-*fused lactam **15**, which was *N*-benzylated to **16**. Reduction of lactam **16** with LiAlH_4_ provided the monobenzylated (5,7)-diamine **17b**, which was hydrogenated to the *trans*-fused (5,7)-diamine **2b**, realizing an overall 7-step sequence in 16% overall yield (Scheme 1A second line).

Although AiZynthfinder did not propose a synthesis for the unknown (6,7)- and (7,7)-diamines **3** and **4**, the Beckmann rearrangement approach proved suitable in both cases. The synthesis of the (6,7)-diamine **3** started with dihydroquinolinone **18**, which reacted stereoselectively with hydroxylamine to the *E*-oxime **19**. Tosylation to the corresponding *E-*tosylate **20** and Beckmann rearrangement then provided lactam **21**. Reducing lactam **21** with LiAlH_4_ to the pyridinoazepane **22** and trifluoroacetylation of the formed secondary amine gave trifluoroacetamide **23**, whose pyridine ring was hydrogenated (10 bar H_2_, Rh/C, *i*PrOH, 70 °C, 5 d) to yield the *cis*-fused piperidine **24**, and finally the corresponding *cis*-fused (6,7)-diamine **3a** after deprotection (7-steps, 32% overall yield from **18**). As for the (5,7)-diamine above, direct hydrogenation of lactam **21** under somewhat stronger conditions (20 bar H_2_, Pd/C, AcOH, 100 °C, 2 days) yielded the *trans-*fused piperidine **25**, which was *N*-benzylated to **26**, reduced with LiAlH_4_ to **27b**, and deprotected to the free *trans-*(6,7)-diamine **3b** (7-steps, 9% overall yield from **18**) (Scheme 1B).

The (7,7)-diamine **4** was obtained starting from 5-methoxytetralone **28** (Scheme 1C). Ring expansion via *E*-oxime **29** and oxime tosylate **30** gave lactam **31**. Demethylation with BBr_3_ provided phenol **32**, whose hydrogenation provided the *trans-*fused cyclohexanol **33** and ketone **34** after reoxidation of the alcohol. While a second Beckmann rearrangement via the *E*-oxime failed in this case, a Schmidt reaction, inspired from a related bicyclic azepane synthesis,^43^ followed by reduction of the crude bis-lactam with LiAlH_4_, provided the *trans*-fused (7,7)-diamine **4b** (8-steps, 10% overall yield from **28**). Unfortunately, the *cis*-fused (7,7)-diamine **4a** could not be obtained despite repeated attempts (Scheme S2).

The ring fusion stereochemistry of **2a**, **2b**, **3a**, **3b**, and **4b** was confirmed by X-ray crystallography of their hydrochloride salts (Figure S2). Overall, *cis*-fused azepanes resulted from hydrogenations of aromatic precursors conducted with only small amounts or no acid (Scheme 1, steps b and f′), while *trans*-fused azepanes were formed when hydrogenation of the aromatic rings required more forcing conditions, sometimes using acetic acid as solvent (Scheme 1, steps l, h′, and e″).

### Identification of Fused Azepane **1a** as a Potent NET Inhibitor

The availability of large data sets of bioactive compounds and their associated biological activities, such as the ChEMBL database,^44^ has allowed to develop models to predict probable targets of any molecule.^45−50^ These tools are meant to help address the possible polypharmacology of any active compound,^49,51^ or to identify the target of active compounds discovered in phenotypic screens.^52^ Here, we used the polypharmacology browser PPB2, which assigns possible targets of a query molecule based on its structural similarity to known bioactive compounds from ChEMBL,^50^ to identify possible targets of our fused azepanes **2**, **3**, and **4**. We did not consider ring fusion stereochemistry because PPB2, similar to most target prediction tools, uses molecular fingerprints that do not account for stereochemistry.

Considering that benzylamines are often biologically active, we also included the five possible singly *N*-benzylated analogs in our search since these were partly available as synthetic intermediates. For each query molecule, we ran PPB2 using the two methods recommended as best performing, which use the Morgan fingerprint ECFP4^53,54^ either alone or in combination with the pharmacophore fingerprint XFP.^55^ Sorting the list of the top-20 proposed targets by rank across the different compounds and methods indicated DAT, NET, and SERT as three probable targets for our compounds, together with σ1-R and the histamine H3 receptors (Table S2, Figures S3–S10).

**Scheme 2:**
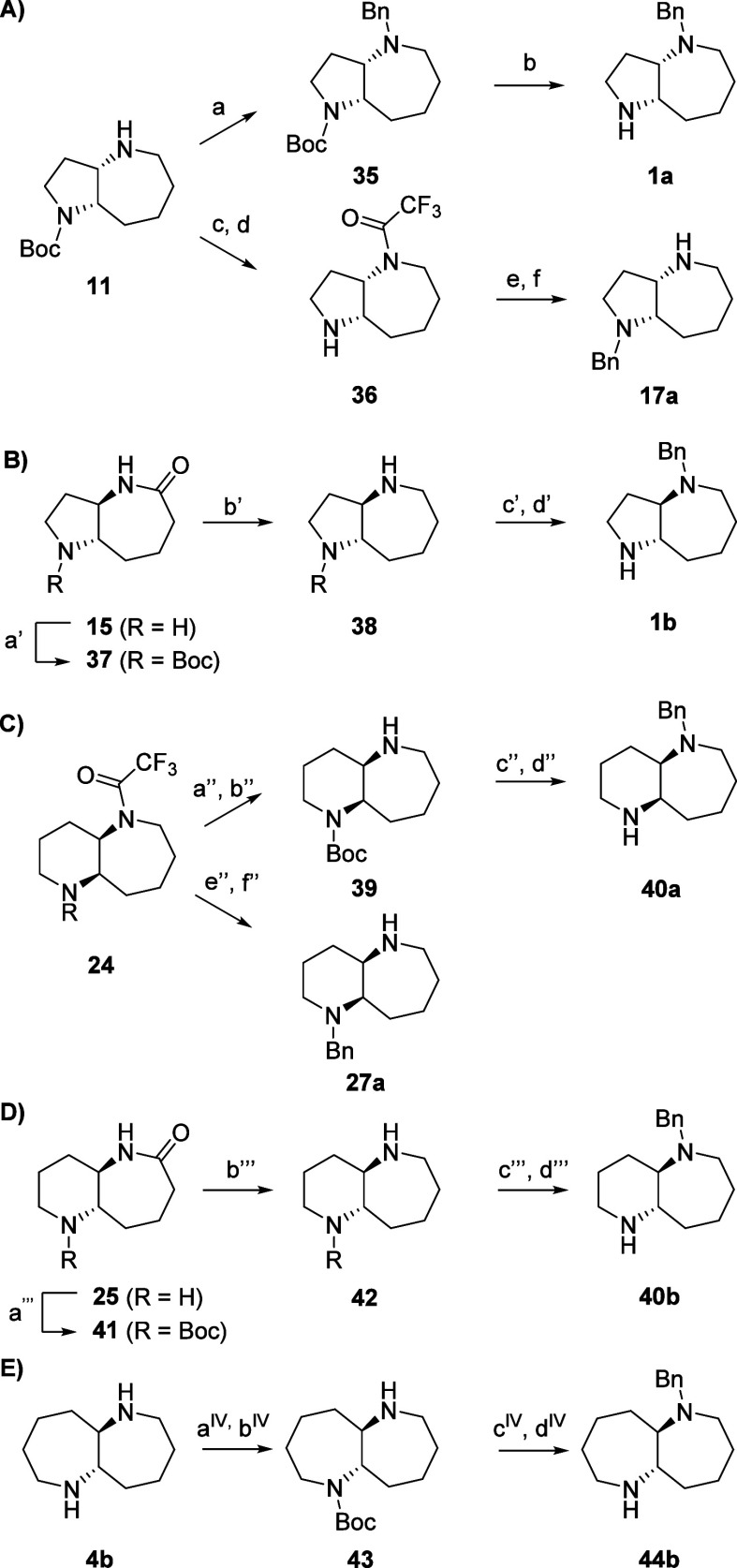
Synthesis of Fused *N*-Benzylated Azepanes (A) Conditions: (a) BnBr, K_2_CO_3_, MeOH, 22 °C, 2 h, 89%; (b) TFA, DCM, 22 °C, 2 h, 64%; (c) TFAA, pyr, DCM, 22 °C, 2 h; d) TFA, DCM, 22 °C, 2 h, 48% over two steps; (e) BnBr, K_2_CO_3_, MeOH, 22 °C, 2 h; (f) LiOH, THF/H_2_O, reflux, 24 h, 39% over two steps; (B) conditions: (a′) Boc_2_O, NEt_3_, DMAP, DCM, 22 °C, 2 h, 64%; (b′) LiAlH_4_, THF, 0 °C then 22 °C, 3 h, 45%; (c′) BnBr, K_2_CO_3_, MeOH, 22 °C, 2 h; (d′) TFA, DCM, 22 °C, 2 h, 70% over two steps; (C) conditions: (a″) Boc_2_O, NEt_3_, DMAP, DCM, 22 °C, 2 h; (b″) LiOH, THF/H_2_O, reflux, 24 h, 76% over two steps; (c″) BnBr, K_2_CO_3_, MeOH, 22 °C, 2 h; (d″) TFA, DCM, 22 °C, 2 h, 79% over two steps; (e″) BnBr, K_2_CO_3_, MeOH, 22 °C, 2 h; (f″) LiOH, THF/H_2_O, reflux, 24 h, 45% over two steps; (D) conditions: (a″′) Boc_2_O, NEt_3_, DCM, 22 °C; 80% b″′) LiAlH_4_, THF, 0 °C, 4 h, 45%; c″′) BnBr, K_2_CO_3_, MeOH, 22°C, 2 h; d″′) TFA, DCM, 22 °C, 2 h, 76% over two steps; (E) Conditions: (a^IV^) Boc_2_O, NEt_3_, DMAP, DCM, 22 °C, 24 h; (b^IV^) TFA, CHCl_3_, 0°C, 90 min, 10% over two steps; (c^IV^) BnBr, K_2_CO_3_, MeOH, 22 °C, 24 h; (d^IV^) TFA, DCM, 22 °C, 2 h, 67% over two steps.

**Figure 3:**
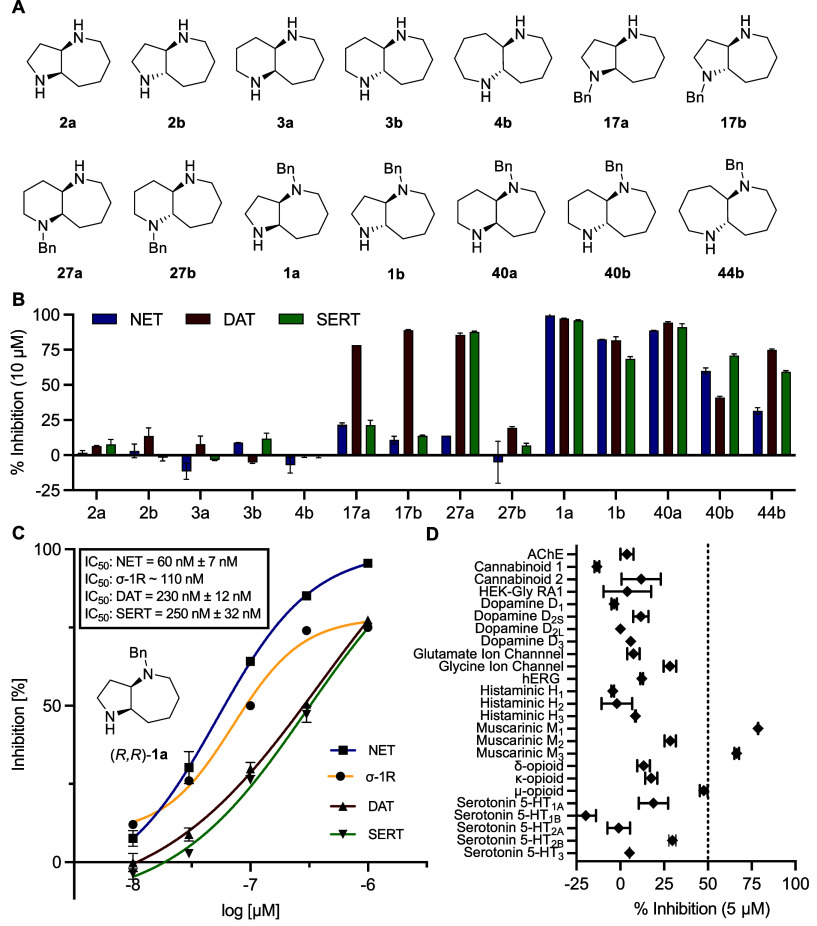
(A) Structures of tested free diamines and monobenzylated diamines. (B) Bar plot of activity on the three targets. Inhibition was measured at 10 μM, high values stand for strong inhibition. (C) IC_50_ curves of (*R,R*)-**1**a against monoamine transporters and σ-1R. (D) *In vitro* polypharmacology on additional less relevant targets of (*R,R*)-**1a**. Data are shown as mean value ± SD of two independent measurements each performed in duplicates. The σ-1R IC_50_ was estimated from duplicate dilution series. The experiments were conducted by Eurofins Cerep SA (France) using radioactive ligand displacement assays (see the Supporting Information for details).

Encouraged by these predictions, we completed the synthesis of the benzylated diamines **1a**/**b**, **17a**, **40a/b**, and **44b** by orthogonal protection/benzylation/deprotection schemes from available intermediates (Scheme 2, Figure 3A). We then subjected the compounds to initial activity assays at 10 μM for DAT, NET, and SERT, for which radioligand displacement assays were available from a commercial provider (Eurofins Cerep SA). While the free diamines were all inactive in these assays, several *N*-benzylated compounds showed strong inhibition against the three targets, in particular compounds *N-*benzylated at the azepane ring (**1a/b**, **40a/b**, and **44b**), with a preference for *cis*-fused versus *trans*-fused compounds (**1a**> **1b**, **40a**> **40b**). The strongest activity *in vitro* was observed with **1a**, the azepane benzylated analog of **2a**, against NET (Figure 3B).

### Target Profile and Structure–Activity Relationships of (*R,R*)-**1a**

Resolution of **1a** enantiomers was achieved by chiral-phase HPLC on the Boc-protected analog **35**, and assignment of their absolute configuration by X-ray crystallography of their hydrochloride salts (Figure S3). Determination of IC_50_ values showed that (*R,R*)-**1a** was a potent NET inhibitor (IC_50_ = 60 ± 7 nM), while its (*S,S*)-**1a** enantiomer was approximately 26-fold less active (IC_50_ = 1.6 ± 0.1 μM). The more active enantiomer (*R,R*)-**1a** also displayed significant activity on DAT (IC_50_ = 230 ± 12 nM) and SERT (IC_50_ = 250 ± 32 nM) (Figure 3C). In addition, (*R,R*)-**1a** showed potent inhibitory effects on the σ-1R (IC_50_ ≈ 110 nM), a chaperone involved in dopaminergic signaling,^56^ which was also indicated as a possible target in our target prediction with PPB2. On the other hand, (*R,R*)-**1a** was mostly inactive against additional targets either predicted by PPB2, such as histamine receptors, or relevant in the context of possible neuroactivity (Figure 3D). In terms of in vitro ADME parameters, (*R*,*R*)-**1a** showed no significant degradation in human liver microsome assay and acceptable human plasma protein binding (49% at 1 μM).

### Targeted Metabolomics Confirms that (*R,R*)-**1a** Inhibits Norepinephrine and Dopamine Uptake but Not Secretion by PC12 Cells

**Figure 4:**
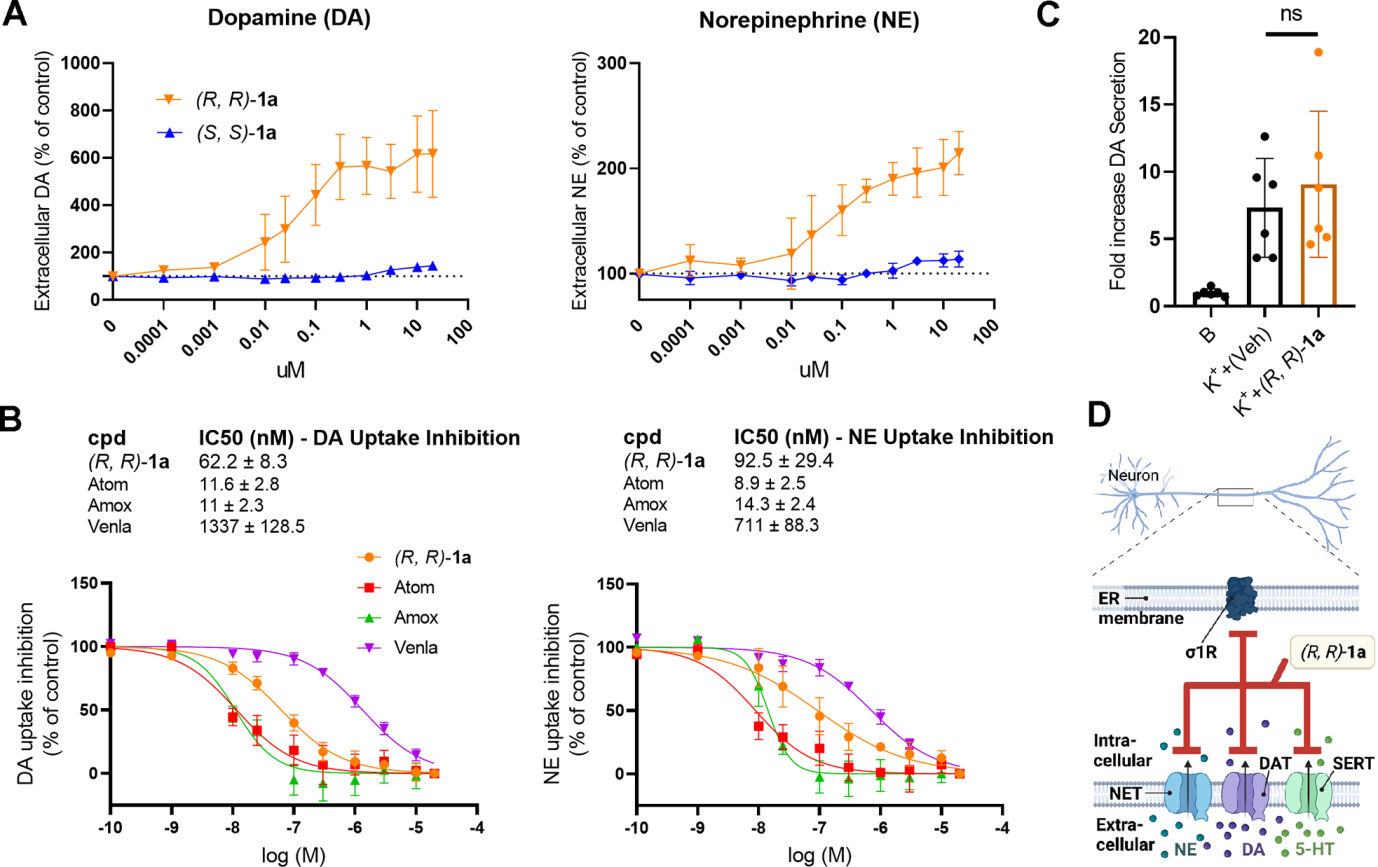
Effects of (*R,R*)-**1a** on norepinephrine (NE) and dopamine (DA) uptake inhibition in PC12 cells. (A) NE and DA levels in the extracellular assay buffer (1 μM of neurotransmitters) were quantified by LC-ESI-MS/MS after 120 min of incubation with DMSO (VehC), (*R,R*)-**1a**, (*S,S*)-**1a**, atomoxetine (Atom), amoxepine (Amox), or venlafaxine (Venla). Data are shown as mean values ± SD from at least three independent experiments. (B) IC_50_ values were determined by fitting the dose–response curves of the uptake inhibitors. (C) DA secretion was evaluated after 24 h of incubation with DMSO or 10 μM (*R,R*)-**1a**. DA release was induced by a 10 min exposure to 59 mM K^+^, and the DA levels in the assay buffer were quantified using LC-ESI-MS/MS and normalized to constitutive secretion. Data are expressed as the mean ± SD from two independent experiments. (D) Schematic representation of the cellular target profile of (*R,R*)-**1a**.

In line with the profiling assays above revealing significant NET, DAT, and SERT neurotransmitter binding inhibition, one would expect that (*R,R*)-**1a** would reduce noradrenaline and dopamine transport in PC12 cells, a well-established neuronal model expressing both DAT and NET.^57,58^ The orthogonal cellular system was optimized to measure the uptake of NE and DAT by LC-ESI-MS/MS under more physiological conditions. (*R,R*)-**1a** treatment of PC12 cells and infusion of neurotransmitters led to a dose-dependent increase of extracellular dopamine and norepinephrine levels (Figure 4A). Although approximately ten times less potent, the profile of (*R,R*)-**1a** on NET and DAT was comparable to atomoxetine (Atom) and amoxetine (Amox), two clinically used NET-selective monoamine transport reuptake inhibitors (Figure 4B). Since PC12 cells do not express SERT, no effect on 5-HT levels were observed (Figure S11). However, the SSRI venlafaxine (Venla) was about 10-fold less potent on DA and NE uptake inhibition in this assay. To assess whether vesicular monoamine transport or secretion was affected, PC12 cells were treated with DMSO or 10 μM (*R,R*)-**1a**, followed by an assessment of depolarization-mediated DA secretion. Notably, the long-term inhibition of monoamine transport by (*R,R*)-**1a** did not affect the vesicular secretion of monoamines induced by 59 mM K^+^, confirming that (*R,R*)-**1a** effectively inhibited monoamine uptake via plasma membrane-localized DAT and NET without affecting vesicular monoamine secretion (Figure 4C). A schematic overview of target profile of (*R,R*)-**1a** is shown in Figure 4D.

### Basic Pharmacokinetic Data on (*R,R*)-**1a**

**Figure 5:**
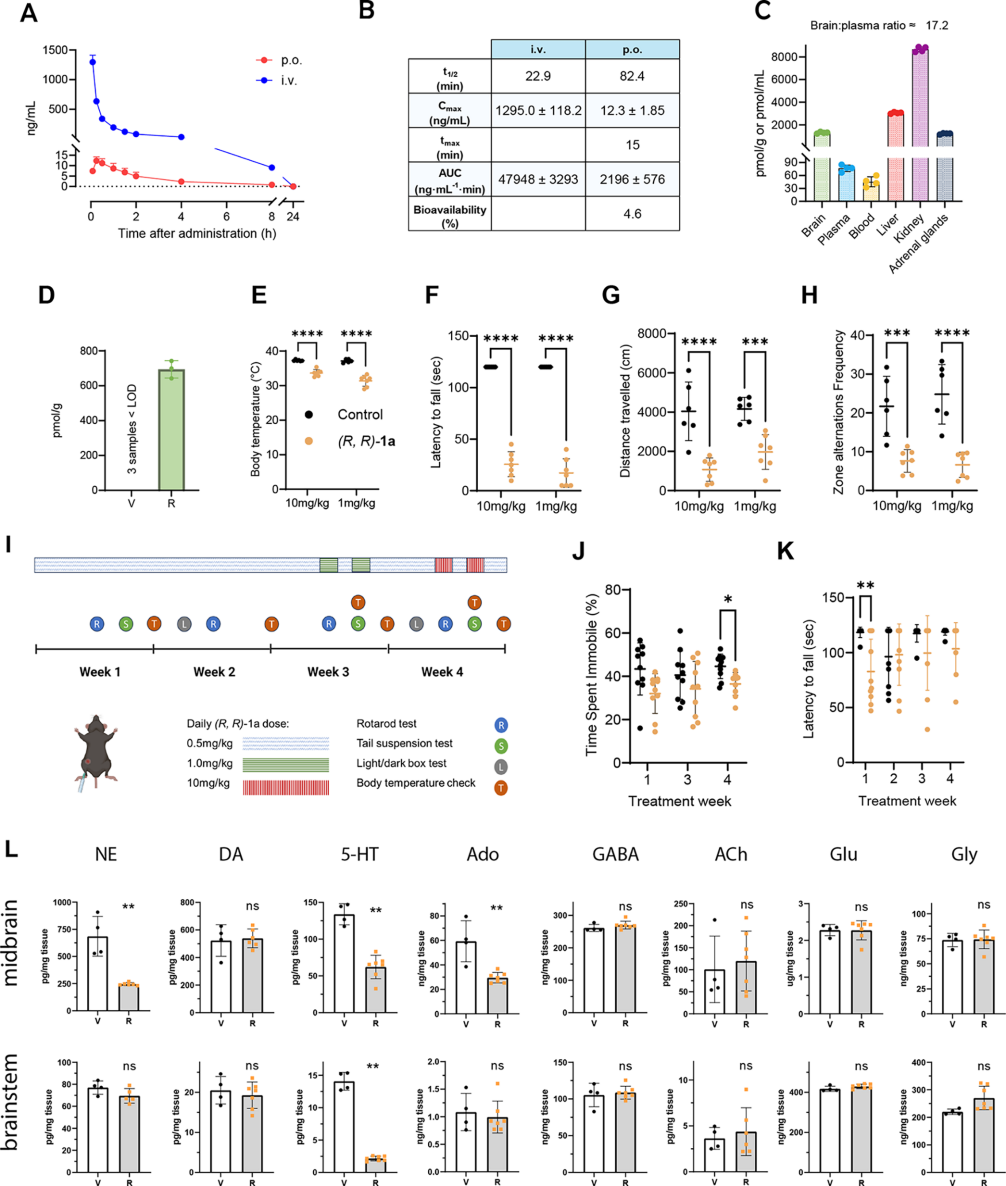
Pharmacokinetics (PK) and pharmacological effects of (*R,R*)-**1a** in and in adult mice. (A) Plasma concentrations of (*R,R*)-**1a** after oral (p.o.) and intravenous (i.v.) administration. Blood samples were collected from the tail vein starting 5 min after drug administration and up to 24 h. C57BL/6JRJ mice (8–9 weeks old, 2 males and 2 females) were administered with 5 mg/kg (*R,R*)-**1a** either using saline (0.9%NaCl) + 16% DMSO + 4.8% Tween 80 or 100% saline as a vehicle for oral or intravenous administration, respectively. (B) Summary of PK data from intravenious (i.v.) and peroral (p.o,) administration of 5 mg/kg of (*R,R*)-**1a.** Data show mean values ± SD of four mice (2 males and 2 females). (C) Evaluation of biodistribution after oral administration. One week after the last blood sampling, the mice were orally administered with 5 mg/kg (*R,R*)-**1a** using saline as a vehicle. Mice were euthanized by decapitation after isoflurane-induced anesthesia 30 min after administration. Then, blood and tissues were harvested. The dose was calculated considering the free salt molecular weight, and 5 mL/kg administration volume. Data show mean values ± SD of four mice (2 males and 2 females). (D) Concentration of (*R,R*)-**1a** measured in brain after 1 mg/kg i.p. One hour post administration (*n* = 3), V = vehicle, R = (*R,R*)-**1a**. (E–H) Effects of acute administration of (*R,R*)-**1a** (10 or 1 mg/kg, i.p., *n* = 7) in mice compared to control vehicle (*n* = 6). (E) Body temperature decrease. (F) Decreased latency in rotatory rod performance. (G) Decrease in the overall distance traveled and (H) in the zone alteration frequency during the dark/light box test indicative of reduced activity. (I–K) Effects of chronic administration of (*R,R*)-**1a** (daily 0.5 mg/kg, i.p.) in mice (*n* = 10/group). (I) Schedule of drug administration and testing. (J) Time spent immobile in the tail suspension assay was unaffected at week 3 and decreased slightly at week 4. (K) Latency in the rotatory rod performance only slightly decreased in week 1. (L) Neurotransmitter concentrations in midbrain and brainstem following acute treatment, measured by LC-ESI-MS/MS. Results show data from individual mice, each measured in two independent LC-ESI-MS/MS analyses of each tissue sample. V = vehicle, R = (*R,R*)-**1a**. Statistical differences were calculated using the two-tailed unpaired student’s *t*-test. ns, not significant, *P < 0.05, **P < 0.01, and ***P < 0.001. Data are plotted as means ± SD.

To assess the oral bioavailability and pharmacokinetics of (*R,R*)-**1a** we first generated a multiple reaction monitoring (MRM)-based quantitative LC-ESI-MS/MS method for this compound (see methods). Next, the compound was administered intravenously (i.v.) at 5 mg/kg at 5 mL/kg to C57BL6/J mixed adult male and female mice. Blood samples were taken from the tail veins over 24 h (Figure 5A). As shown in Figure 5B, (*R,R*)-**1a** showed a *C*_max_ of 1295.0 ± 118.2 nM and a *t*_1/2_ of 22.9 min. After 8 h, about 10 ng/mL were still found in the blood. After 1 week of washout period, mice were administered (*R,R*)-**1a** by oral gavage (p.o.) at 5 mg/kg at 5 mL/kg. Blood samples were taken from the tail veins over 24 h. This experiment provided a *C*_max_ of 12.3 ± 1.85 nM and a *t*_1/2_ of 82.4 min. After 1 week of washout, mice were administered again 5 mg/kg p.o. at 5 mL/kg and mice were euthanized 30 min after administration and organs were collected. The compound rapidly accumulated in different tissues, primarily the kidney (>9000 pmol/g at 30 min) (Figure 5C). The calculated brain to plasma ratio at 30 min was 17.2 and the volume distribution 0.28 (13.2 L/kg). In addition to the brain, the adrenal glands also accumulated (*R,R*)-**1a** strongly. Using an oral formulation of saline only, the absolute oral bioavailability was calculated as 4.6%. Given the outstanding brain penetrance of (*R,R*)-**1a** (brain-to-plasma ratio Kp > 15), already low doses led to brain concentrations in the higher nanomolar range, i.e., concentrations that target monoamine transporters.

### (*R,R*)-**1a** Acts as an Acute Sedative in Mice without Showing Chronic Sedative Effects

Since changes in the availability of monoamines in multiple brain regions, including the midbrain, are known to regulate the expression of coping behavior in rodents,^59,60^ we performed a preliminary evaluation of (*R,R*)-**1a** on mouse behavior. Adult male mice (8–10 weeks old, body weight matched, Figure S12A) were acclimatized to handling over 10 days and trained for an additional 2 days to perform the rotarod test as a simple motor coordination task.^61^ For the sake of reducing stress to mice, intraperitoneal (i.p.) administrations were used for the behavioral assays. One mg/kg of (*R,R*)-**1a** administered i.p. resulted in a brain concentration of 694 ± 50 pmol/g at 1 h, which was approximately 30% of the concentration seen with 5 mg/kg p.o administration at 30 min (Figure 5D).Upon treatment with (*R,R*)-**1a** (1.0 or 10 mg/kg, i. p.), an unexpected pronounced sedative or lethargic phenotype was observed already after 40 min, coupled with a significant decrease in core body temperature (Figure 5E). Treated animals furthermore exhibited severe deficits in motor coordination, illustrated by the inability to perform a modest Rotarod task (Figure 5F). Importantly, no signs of acute toxicity were observed, such as piloerection, hunched posture, convulsions, or gastrointestinal distress, and the mice were very calm when handled. Furthermore, we examined freely moving exploratory and anxiety-related behaviors using the light/dark box task.^62^ Treated mice exhibited dramatically decreased mobility, which was well visible in reduced distance covered (Figure 5G) and zone alternation frequency (Figure 5H), as well as in reduced overall mobility (Figure S12B), mean velocity (Figure S12C), cumulative zone frequency (Figure S12D), and limited meandering behaviors (Figure S12E).

To evaluate the effects of chronic administration of a lower dose, we administered daily doses of 0.5 mg/kg of (*R,R*)-**1a** or vehicle control for 4 weeks to adult male and female mice (8 weeks old, body weight matched) with higher doses at the end of week 3 (1 mg/kg) and 4 (10 mg/kg), to assess tolerance (Figure 5I). Upon weekly inspection, body temperature was unchanged and only decreased upon acute treatment with 10 mg/kg at the end of week 4, but not with 1 mg/kg at the end of week 3, suggestive of tolerance (Figure S13A/B). Treated animals tended to display increased mobility compared to controls in the tail suspension test, indicative of increased stress resilience,^63^ although this effect only reached statistical significance in week 4 in response to 10 mg/kg (*R,R*)-**1a** (Figure 5J). Furthermore, motor coordination was only slightly affected in week 1, but the effect did not persist afterward, even in weeks 3 or 4, when the animals received higher doses (Figure 5K). The sedation of acute doses and the lack of decrease in body weight between treated and control mice at the end of the study period suggests an atypical and nonamphetamine-type neuropharmacology that could also be mediated by σ-1R (Figure S13C). This was also confirmed by the fact that there was no significant difference in behavior between treated and control animals in the light/dark box assay in which NET/DAT inhibitors typically show anxiogenic effects (Figure S13D).

### Acute Administration of (*R,R*)-**1a** Reduces Neurotransmitter Levels in Mice Brain

Quantifying neurotransmitter levels in midbrain and brainstem regions of mice sacrificed 1 h after treatment with 10 mg/kg (*R,R*)-**1a** showed significantly decreased NE, 5-hydroxytryptamine (5-HT), and adenosine levels in the midbrain and 5-HT levels in the brainstem compared to control, while other neurotransmitters (DA, γ-aminobutyric acid, acetylcholine, glutamate, and glycine) were not affected (Figure 5L). The observed decrease in monoamine levels upon acute administration, although not fully understood at this point, aligns with documented responses observed for substances acting on monoamine transport, such as SSRIs.^64^

By contrast, chronic exposure did not yield significant changes in neurotransmitter levels in the midbrain and brainstem, suggesting the involvement of mechanisms associated with drug adaptation and tolerance (Figure S14). Notably, a trend toward increased DA (*p* = 0.16) was only observed in chronically treated mice. Overall, the behavioral effects observed with (*R*,*R*)-**1a** display a unique and novel profile, which might also be mediated by its inhibition of σ-1R.

### Structural Modification of (*R,R*-**1a**) Suggests a Druggable Profile of *N*-Benzylated Bicyclic Azepanes

**Figure 6:**
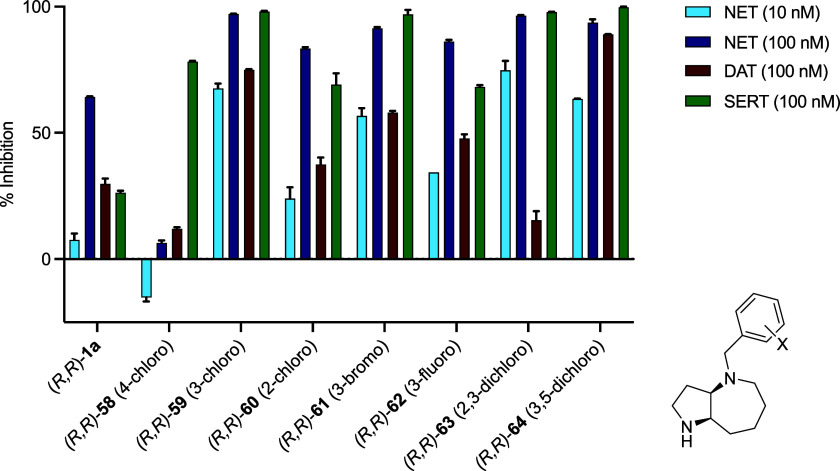
Preliminary structure–activity relationship (SAR) data of halogenated analogues of (*R,R*)-**1a**. Bar plot showing the comparative inhibitory activity on monoamine transporters NET, DAT, and SERT. Data are shown as mean of two measurements each performed in triplicates. The experiments were conducted by Eurofins Cerep SA (France) using a radioactive ligand displacement assay.

To test if (*R,R*)-**1a** might be amenable to optimization by structural variations, we prepared a series of halogenated analogs of (*R,R*)-**1a** by debenzylation of the enantiomerically pure intermediate (*R,R*)-**35** and reductive alkylation with the corresponding halogenated benzaldehydes (Scheme S3). While the *p*-chloro substituent strongly reduced NET inhibition, *o-* and particularly *m-*chloro and *m*-bromo substituents strongly increased potency against NET as well as against DAT and SERT (Figure 6). This showed the potential to further develop (*R,R*)-**1a** as a lead compound with potentially differential effects toward these targets.

## Conclusions

In summary, an analysis of the 1,139 possible mono- and bicyclic diamines comprising only five-, six-, or seven-membered rings revealed that many of these simple scaffolds are still unknown. Focusing on fused azepanes predicted by AiZynthfinder to be synthetically accessible via a Beckmann rearrangement from the parent cyclohexanone oximes, we realized the stereoselective synthesis of *cis*- and *trans*-fused diamines **2**, **3**, and **4** and their regioselectively mono-*N*-benzylated analogs. Activity screening against the DAT, NET and SERT, predicted as targets using the polypharmacology browser PPB2, and resolution of active enantiomers, revealed that the *N*-benzylated *cis*-fused (5,7)-diamine (*R,R*)-**1a** displayed nanomolar inhibition of NET, DAT and SERT, as well as σ-1R as predicted by PPB2. Even though NET, DAT, and SERT accept a broad range of chemotypes,^65^ molecules reaching nanomolar potencies on these transporters are relatively rare, and (*R,R*)-**1a** possesses a unique and novel target profile. Although with an initial nonoptimized peroral formulation in saline, this compound only reached an initial weak oral bioavailability of about 4.5%, it showed an outstanding brain penetrance (Kp > 15) and acceptable *C*_max_ and *t*_1/2_ values, thus reaching high nanomolar to micromolar concentrations in the brain. Interestingly, (*R,R*)-**1a** has 17 non-hydrogen atoms and falls in the range of GDB-17. Structure–activity profiling showed that halogenation of the benzyl group strongly modulated activity and selectivity against the three monoamine transporters, indicating that the *N*-benzylated bicyclic azepane scaffold is tunable and therefore offers a feasible starting point for CNS drug development.

DA and NE uptake inhibition by (*R,R*)-**1a** was confirmed in both radioligand assays and an orthogonal cellular assay using PC-12 cells. Acute inhibition of monoamine transporters can paradoxically reduce overall neurotransmitter levels due to decreased recycling, increased metabolism, and autoreceptor-mediated suppression of release.^66^ Neuropharmacological experiments with (*R,R*)-**1a** in mice showed an unexpectedly strong but reversible sedative effect upon acute administration, coinciding with an acute and apparently specific drop of NE, 5-HT, and adenosine levels in the midbrain. Only 5-HT was strongly reduced in the brainstem, which is interesting because median raphe serotonergic neurons projecting to the interpeduncular nucleus control preference and aversion.^67^

While the metabolomic data confirmed the apparent specificity of (*R,R*)-**1a** toward monoamine regulation, possibly via targeting the locus coeruleus, the nontoxic but sedative neuropharmacological effects at higher doses were unexpected and need further characterization. In the absence of the typical agitation-like behaviors or weight loss effects normally seen with NET inhibitors, the antidepressant-like effects observed upon chronic administration of (*R,R*)**-1a** could be explored for the treatment of neuropsychiatric disorders associated with monoamine dysregulation. Overall, our study highlights the potential of simple but still unexplored scaffolds for drug discovery from *in silico* design to preliminary *in vivo* pharmacological validation. By introducing a novel monoamine transporter inhibitor scaffold,^66^ our study also highlights how interdisciplinary research in academia can drive innovative early-stage drug discovery.^68^

## Experimental Section

### Chemical Synthesis

#### *tert*-Butyl-4-oxo-4,5,6,7-tetrahydro-1*H*-indole-1-carboxylate (**6**)^69^

To a solution of commercially available 1,5,6,7-tetrahydro-4*H*-indol-4-one 5 (10.0 g, 74.0 mmol, 1.0 equiv) in CH_3_CN (370 mL, 0.2 M) was added Boc_2_O (17.7 g, 81.1 mmol, 1.1 equiv), DIPEA (19.3 mL, 111 mmol, 1.5 equiv), and DMAP (cat.). The mixture was stirred at 22 °C for 24 h. Then, the solvent was evaporated, and the crude was purified by flash column chromatography on silica gel (30% EtOAc in heptane) to isolate Boc-protected amine **6** (17.4 g, 74.0 mmol, quant.) as an orange solid. The analytical data is in accordance with literature-reported data.^70^ R_f_ = 0.25 (30% EtOAc in heptane); ^1^H NMR (400 MHz, CDCl_3_, 298 K): δ [ppm] = 7.13 (d, *J* = 3.5 Hz, 1H); 6.51 (d, *J* = 3.5 Hz, 1H), 3.10 (t, *J* = 6.2 Hz, 2H), 2.44 (t, *J* = 4.4 Hz, 2H), 2.12 (quint., *J* = 6.4 Hz, 2H), 1.58 (s, 9H); HR-MS (ESI): (*m*/*z*) = calculated for C_13_H_17_NO_3_Na^+^ [*M*+Na]^+^: 258.1101, found: 258.1097.

#### *tert*-Butyl-4-hydroxyoctahydro-1*H*-indole-1-carboxylate ((±)-**7**)^70^

A mixture of ketone **6** (8.93 g, 38.0 mmol, 1.0 equiv), PtO_2_ (PtO_2_ on activated charcoal, 890 mg, 10 wt % of substrate) and AcOH (2.40 mL, 41.8 mmol, 1.1 equiv) in *^i^*PrOH (50 mL) was stirred in a sealed autoclave at 50 °C under H_2_ pressure (10 bar) for 6 h. After cooling to room temperature, the reaction mixture was carefully filtered over Celite, washed with MeOH, and evaporated to dryness. The crude was purified by flash column chromatography on silica gel (30%–60% EtOAc in heptane) to afford aliphatic bicycle (±)-**7** (6.54 g, 27.1 mmol, 71%) as a yellow oil. R_f_ = 0.20 (30% EtOAc in heptane); ^1^H NMR (400 MHz, MeOD-*d*_4_, 298 K): δ [ppm] = 3.88–3.93 (m, 1.4H), 3.73–3.80 (m, 0.6H), 3.43–3.49 (m, 0.6H) 3.36–3.40 (m, 0.5H), 3.25–3.28 (m, 0.4H), 2.42–2.55 (m, 0.6H), 2.20 (br, 0.4H), 1.84–1.98 (m, 3H), 1.59–1.70 (m, 2.5H), 1.45 (s, 9H), 1.00–1.40 (m, 3H); ^13^C NMR (100 MHz, MeOD-*d*_4_, 298 K): δ [ppm] = 156.1, 2X 80.6, 2X 70.3, 69.0, 58.6, 58.2, 56.0, 46.1, 46.0, 45.6, 45.3, 2X 30.0, 28.8, 28.1, 27.6, 23.5, 22.7, 22.4, 18.7; due to the presence of alcohol isomers in the NMR measurement, structural assignment is not possible; HR-MS (ESI): (*m*/*z*) = calculated for C_13_H_23_NO_3_Na^+^ [*M*+Na]^+^: 264.1570, found: 264.1566.

#### *tert*-Butyl-4-oxooctahydro-1*H*-indole-1-carboxylate ((±)-**8**):^70^

A solution of alcohol (±)-**7** (6.70 g, 27.8 mmol, 1.0 equiv) in DCM (275 mL, 0.1 M) was cooled to 0 °C, and DMP (23.5 g, 55.4 mmol, 2.0 equiv) was added. The reaction was stirred at 22 °C for 30 min. Then, it was quenched by the addition of sat. NaHCO_3_ solution (125 mL) and Na_2_S_2_O_3_ solution (2 M, 125 mL). The water phase was washed with DCM (3 × 250 mL). The combined organic phases were washed with dionized H_2_O (250 mL), dried over Na_2_SO_4_, and filtered, and the solvent was evaporated in vacuo. The crude was purified by column chromatography on silica gel (30% EtOAc in heptane) to yield ketone (±)-**8** (6.63 g, 27.7 mmol, quant.) as a white solid. R_f_ = 0.20 (25% EtOAc in heptane); ^1^H NMR (400 MHz, MeOD-*d*_4_, 298 K): δ [ppm] = 4.07–4.12 (m, 1H; H-*C*(7a)), 3.34–3.44 (m, 2H; H-*C* (2)), 2.87–2.93 (m, 1H; H-*C*(3a)), 2.38–2.47 (m, 1H; H-*C* (5)), 2.27–2.34 (m, 1H; H-*C* (5)), 2.16–2.25 (m, 2H; H-*C* (3 and 7)), 1.95–2.03 (m, 1H; H-*C* (3)), 1.84–1.91 (m, 1H; H-*C* (6)), 1.58–1.72 (m, 2H; H-*C* (6 and 7)), 1.47 (s, 9H; H_3_-*C*_Boc_); ^13^C NMR (100 MHz, MeOD-*d*_4_, 298 K): δ [ppm] = 213.3 (C (4)), 156.1 ((*C*=O)_Boc_), 81.1 ((C_q_)_Boc_), 60.3 (C(7a)), 53.1 (C(3a)), 46.4 (C (2)), 39.4 (C (5)), 28.7 ((*C*H_3_)_Boc_), 2X 28.0 (C (3 and 7)), 22.0 (C (6)); HR-MS (ESI): (*m*/*z*) = calculated for C_13_H_21_NO_3_Na^+^ [*M*+Na]^+^: 262.1414, found: 262.1408.

#### *tert*-Butyl-5-oxooctahydropyrrolo[3,2-*b*]azepine-1(2*H*)-carboxylate ((±)-**10**)

Ketone (±)-**8** (2.0 g, 8.36 mmol, 1.0 equiv) was dissolved in pyridine (25 mL), and NH_2_OH•HCl (1.16 g, 16.7 mmol, 2.0 equiv) was added. The reaction mixture was stirred at 22 °C for 2 h. Then, the pyridine was evaporated, and the residue was taken up in dionized H_2_O (25 mL) and extracted with EtOAc (3 × 50 mL). The combined organic layers were dried over Na_2_SO_4_ and filtered, and the solvent was evaporated to afford a mixture of oxime isomers as a white sticky solid. The intermediate product was dissolved in pyridine (25 mL) was added freshly recrystallized *p*-TsCl (1.81 g, 9.49 mmol, 1.2 equiv) and the solution was stirred at 22 °C for 2 h. After completion, the solvent was evaporated, and the crude was taken up in dionized H_2_O (25 mL) and extracted with EtOAc (3 × 50 mL). The organic phases were dried over Na_2_SO_4_ and filtered, and the solvent was evaporated to yield a mixture of tosyl isomers as yellow sticky solid. The intermediate product was further reacted with potassium acetate (2.34 g, 23.8 mmol, 3.0 equiv) in a mixture of EtOH (50 mL) and deionized H_2_O (50 mL). The reaction was stirred and refluxed for 16 h. After cooling to room temperature and evaporation of the EtOH, the remaining aqueous solution was adjusted to pH = 10 by the addition of aqueous NaOH solution (1 M, 6 mL). The aqueous phase was extracted with DCM (3 × 200 mL). The combined organic layers were dried over Na_2_SO_4_ and filtered, and the solvent was removed under reduced pressure. The crude product was purified by flash column chromatography on silica gel (80% EtOAc in heptane to EtOAc) to isolate the major lactam regioisomer (±)-**10** (690 mg, 2.70 mmol, 32%, ratio 1.3:1) as a white solid. R_f_ = 0.27 (EtOAc); ^1^H NMR (400 MHz, MeOD-*d*_4_, 298 K): δ [ppm] = 4.20–4.24 (m, 1H; H–C(3a)), 3.78–3.83 (m, 1H; H–C(8a)), 3.58–3.64 (m, 1H; H–C (2)), 3.25–3.29 (m, 1H; H–C (2)), 2.61–2.70 (m, 1H; H–C (6)), 2.31 (br, 1H; H–C (7)), 2.18–2.24 (m, 1H; H–C (6)), 2.05–2.15 (m, 1H; H–C (3)), 1.86–1.93 (m, 1H; H–C (3)), 1.71–1.77 (m, 2H; H–C (7 and 8)), 1.30–1.46 (m, 10H; H3-CBoc and H–C (8)); ^13^C NMR (100 MHz, MeOD-*d*_4_, 298 K): δ [ppm] = 177.6 (C (5)), 156.0 ((C = O)Boc), 81.2 ((C_q_)Boc), 60.8 (C(8a)), 55.6 (C(3a)) 45.1 (C (2)), 33.1 (C (6)), 31.3 (C (3)), 28.7 ((CH_3_)Boc), 27.4 (C (7)), 26.9 (C (8)); HR-MS (ESI): (*m*/*z*) = calculated for C_13_H_23_N_2_O_3_^+^ [M + H]^+^: 255.1703, found: 255.1686.

#### *tert*-Butyl-(3a*S*,8a*S*)-4-oxooctahydropyrrolo[3,2-*c*]azepine-1(2*H*)-car*b*oxylate ((±)-**9**)

Minor lactam regioisomer (±)-**9** (523 mg, 2.06 mmol, 25%, ratio 1:1.3) was isolated as a white solid from the above reaction. R_f_ = 0.20 (80% EtOAc in heptane); ^1^H NMR (400 MHz, MeOD-*d*_4_, 298 K): δ [ppm] = 3.93–3.99 (m, 1H), 3.44–3.52 (m, 2H), 3.38–3.42 (m, 1H), 3.18–3.23 (m, 1H), 3.10–3.16 (m, 1H), 2.29–2.36 (m, 1H), 2.22 (br, 1H), 1.84–2.09 (m, 2H), 1.72–1.82 (m, 1H), 1.62–1.67 (m, 1H), 1.46 (s, 9H; H_3_-*C*_Boc_); ^13^C NMR (100 MHz, MeOD-*d*_4_, 298 K): δ [ppm] = 176.6 (C (4)), 155.9 ((*C*=O)_Boc_), 81.20 ((C_q_)_Boc_), 56.8 (*C*H), 47.3 (*C*H), 46.4 (*C*H_2_), 39.1 (*C*H_2_), 28.7 ((*C*H_3_)_Boc_), 27.3 ((*C*H_2_), 27.0 ((*C*H_2_), 25.2 (CH_2_); HR-MS (ESI): (*m*/*z*) = calculated for C_13_H_23_N_2_O_3_^+^ [*M*+H]^+^: 255.1703, found: 255.1687.

#### *tert*-Butyl-octahydropyrrolo[3,2-*b*]azepine-1(2*H*)-carboxylate ((±)-**11**)

A solution of lactam (±)-**10** (568 mg, 2.23 mmol, 1.0 equiv) in dry THF (24 mL, 0.1 M) was cooled to 0 °C, LiAlH_4_ (1 M in THF, 3.4 mL, 3.40 mmol, 1.5 equiv) was added dropwise and the reaction was stirred at 22 °C for 4 h. The reaction was then worked up using Fieser's protocol, and the crude was purified by flash column chromatography on silica gel (10% MeOH in DCM + 0.1% NEt_3_) to give azepane (±)-**11** (248 mg, 1.03 mmol, 46%) as a colorless oil. R_f_ = 0.20 (10% MeOH in DCM + 0.1% NEt_3_); ^1^H NMR (400 MHz, MeOD-*d*_4_, 298 K): δ [ppm] = 3.76–3.81 (m, 1H; H-*C*(8a)), 3.53–3.59 (m, 1H; H-*C*(3a)), 3.39 (t, *J* = 8.9 Hz, 1H; H-*C* (2)), 3.22–3.29 (m, 1H; H-*C* (2)), 3.03–3.06 (m, 1H; H-*C* (5)), 2.46–2.53 (m, 1H; H-*C* (5)), 2.03–2.10 (m, 1H; H-*C* (3)), 1.70–1.92 (m, 4H; H-*C* (3, 6, 7 and 8)), 1.59–1.61 (m, 1H; H-*C* (8)), 1.45 (m, 11H; H_3_-*C*_Boc_ and H-*C* (6, 7)), ^13^C NMR (100 MHz, MeOD-*d*_4_, 298 K): δ [ppm] = 156.4 ((*C*=O)_Boc_), 80.8 ((C_q_)_Boc_), 64.0 (C(8a)), 61.9 (C(3a)), 49.0 (C (5)), 45.3 (C (2)), 32.9 (C (6 or 7)), 31.3 (C (3)), 30.1 (C (8)), 28.8 ((*C*H_3_)_Boc_), 27.4 (C (6 or 7)); HR-MS (ESI): (*m*/*z*) = calculated for C_13_H_25_N_2_O_2_^+^ [*M*+H]^+^: 241.1911, found: 241.1912.

#### Decahydropyrrolo[3,2-*b*]azepine ((±)-**2a**)

To a solution of mono-Boc-protected diamine (±)-**11** (90.0 mg, 0.37 mmol, 1.0 equiv) in DCM (4 mL, 0.1 M) was added TFA (0.4 mL, 10 vol %). The reaction was stirred at 22 °C for 2 h. Then, it was washed with NaOH (1 M, 2 × 4 mL); the organic phase was dried over Na_2_SO_4_ and filtered; and the solvent was evaporated to obtain the final free diamine (±)-**2a** (46.5 mg, 0.33 mmol, 89%) as a yellow oil. ^1^H NMR (400 MHz, MeOD-*d*_4_, 298 K): δ [ppm] = 3.59–3.64 (m, 1H; H-*C*(3a)), 3.50–3.56 (m, 1H; H-*C*(8a)), 3.32–3.37 (m, 1H; H-*C* (2)), 3.18–3.23 (m, 1H; H-*C* (5)), 3.04–3.11 (m, 1H; H-*C* (2)), 2.51–2.58 (m, 1H; H-*C* (5)), 2.36–2.45 (m, 1H; H-*C* (3)), 2.04–2.11 (m, 1H; H-*C* (8)), 1.94–1.95 (m, 1H; H-*C* (7)), 1.74–1.84 (m, 3H; H-*C* (3, 6 and 8)), 1.52–1.63 (m, 1H; H-*C* (6)), 1.35–1.45 (m, 1H; H-*C* (7)); ^13^C NMR (100 MHz, MeOD-*d*_4_, 298 K): δ [ppm] = 65.0 (C(8a)), 62.8 (C(3a)), 51.4 (C (5)), 45.3 (C (2)), 33.7 (C (3)), 32.7 (C (6)), 29.7 (C (8)), 25.5 (C (7)); HR-MS (ESI): (*m*/*z*) = calculated for C_8_H_17_N_2_^+^ [*M*+H]^+^: 141.1386, found: 141.1381.

#### (*E*)-1,5,6,7-Tetrahydro-4*H*-indol-4-one oxime (*E*-**12**)^71^

To a solution of commercially available 1,5,6,7-tetrahydro-4H-indol-4-one 5 (10.4 g, 76.9 mmol, 1.0 equiv) in pyridine (80 mL, 1 M) was added NH_2_OH·HCl (10.7 g, 154 mmol, 2.0 equiv). The mixture was stirred at 22 °C for 2 h, then the pyridine was evaporated, and the residue was diluted with dionized H_2_O (150 mL) and extracted with EtOAc (3 × 200 mL). The organic layers were dried over Na_2_SO_4_ and filtered, and the solvent was removed in vacuo. The crude product was purified by flash column chromatography on silica gel (50% EtOAc in heptane) to isolate the major (*E*)-oxime isomer *E*-**12** (9.24 g, 61.5 mmol, 80%; E/Z 4:1) as a yellow solid. R_f_ = 0.18 (50% EtOAc in heptane); ^1^H NMR (400 MHz, MeOD-*d*_4_, 298 K): δ [ppm] = 6.84 (d, *J* = 3.0 Hz, 1H; H-*C* (2)), 6.62 (d, *J* = 3.0 Hz, 1H; H-*C* (3)), 2.72 (t, *J* = 6.2 Hz, 2H; H_2_-*C* (7)), 2.38–2.42 (m, 2H; H_2_-*C* (5)), 1.96 (quint., *J* = 3.7 Hz, 2H; H_2_-*C* (6)); ^13^C NMR (100 MHz, MeOD-*d*_4_, 298 K): δ [ppm] = 152.8 (C (4)), 136.9 (C(7a)), 117.7 (C (3)), 112.3 (C(3a)), 111.4 (C (2)), 30.6 (C (5)), 24.9 (C (6)), 24.2 (C (7)); HR-MS (ESI): (*m*/*z*) = calculated for C_8_H_11_N_2_O^+^ [*M*+H]^+^: 151.0866, found: 151.0863.

#### (*Z*)-1,5,6,7-Tetrahydro-4*H*-indol-4-one oxime (*Z*-**12**)^71^

Minor (*Z*)-oxime isomer *Z*-**12** (2.31, 15.4 mmol, 13%, Z/E 1:4) was isolated as a white solid from the above reaction. R_f_ = 0.31 (50% EtOAc in heptane); ^1^H NMR (400 MHz, MeOD-*d*_4_, 298 K): δ [ppm] = 6.57 (d, *J* = 3.0 Hz, 1H; H-*C* (2)), 6.30 (d, *J* = 3.0 Hz, 1H; H-*C* (3)), 2.67 (t, *J* = 6.5 Hz, 2H; H_2_-*C* (5 or 7)), 2.63 (t, *J* = 6.2 Hz, 2H; H_2_-*C* (5 or 7)), 1.89 (quint., *J* = 6.3 Hz, 2H; H_2_-*C* (6)); ^13^C NMR (100 MHz, MeOD-*d*_4_, 298 K): δ [ppm] = 156.0 (C (4)), 135.8 (C(7a)), 118.7 (C (2)), 114.4 (C(3a)), 103.8 (C (3)), 23.8 (C (5, 6 or 7)), 23.7 (C (5, 6 or 7)), 23.3 (C (5, 6 or 7)); HR-MS (ESI): (*m*/*z*) = calculated for C_8_H_11_N_2_O^+^ [*M*+H]^+^: 151.0866, found: 151.0863.

#### (*E*)-1,5,6,7-Tetrahydro-4*H*-indol-4-one*O*-tosyl oxime (**13**)^72^

To a solution of (*E*)-oxime isomer *E*-**12** (2.18 g, 14.5 mmol, 1.0 equiv) in pyridine (20 mL) was added freshly recrystallized *p*-TsCl (3.32 g, 17.4 mmol, 1.2 equiv). The mixture was stirred at 22 °C for 2 h, then the pyridine was evaporated in vacuo, and the crude product was purified by flash column chromatography on silica gel (EtOAc) to afford tosylate **13** (3.94 g, 12.9 mmol, 89%) as a yellow solid. R_f_ = 0.23 (60% EtOAc in heptane); ^1^H NMR (400 MHz, CDCl_3_, 298 K): δ [ppm] = 7.91 (d, *J* = 8.3 Hz, 2H; H-*C*_tos._, 7.31 (d, *J* = 8.0 Hz, 2H; H-*C*_tos._, 6.62 (t, *J* = 2.7 Hz, 1H; H-*C* (3)), 6.37 (t, *J* = 2.8 Hz, 1H; H-*C* (2)), 2.72 (t, *J* = 6.5 Hz, 2H; H_2_-*C* (5)), 2.63 (t, *J* = 6.2 Hz, 2H; H_2_-*C* (7)), 2.42 (s, 3H; H_3_-*C*_tos_), 1.91 (quint., *J* = 6.3 Hz, 2H; H_2_-*C* (6)); ^13^C NMR (100 MHz, CDCl_3_, 298 K): δ [ppm] = 161.0 (C (4)), 144.6 ((C_q_)_tos._), 137.1 (C(7a)), 133.3 ((C_q_)_tos._), 129.5 ((*C*H)_tos._), 129.1 ((*C*H)_tos._), 118.3 (C (3)), 111.9 (C(3a)), 104.8 (C (2)), 24.3 (C (5)), 22.3 (C (6 or 7)), 22.3 (C (6 or 7)), 21.8 ((*C*H_3_)_tos._); HR-MS (ESI): (*m*/*z*) = calculated for C_15_H_17_N_2_O_3_S^+^ [*M*+H]^+^: 305.0954, found: 305.0945.

#### 4,6,7,8-Tetrahydropyrrolo[3,2-*b*]azepin-5(1*H*)-one (**14**)^72^

A mixture of tosylate **13** (2.04 g, 6.70 mmol, 1.0 equiv) and KOAc (1.98 g, 20.2 mmol, 3.0 equiv) in EtOH (50 mL) and dionized H_2_O (50 mL) was stirred and refluxed for 12 h. After cooling to room temperature and evaporation of the EtOH, the remaining aqueous solution was adjusted to pH = 10 by the addition of aqueous NaOH solution (1 M, 6 mL). The aqueous phase was extracted with DCM (3 × 200 mL). The combined organic layers were dried over Na_2_SO_4_and filtered, and the solvent was removed under reduced pressure. The crude product was purified by flash column chromatography on silica gel (EtOAc) to afford lactam **14** (0.71 g, 4.73 mmol, 71%) as a white solid. R_f_ = 0.27 (EtOAc); ^1^H NMR (400 MHz, MeOD-*d*_4_, 298 K): δ [ppm] = 6.51 (d, *J* = 2.9 Hz, 1H; H-*C* (3), 5.76 (d, *J* = 2.9 Hz, 1H; H-*C* (2)), 2.83 (t, *J* = 6.8 Hz, 2H; H_2_-*C* (8)), 2.52–2.55 (m, 2H; H_2_-*C* (6)) 1.99–2.05 (m, 2H; H_2_-*C* (7)); ^13^C NMR (100 MHz, MeOD-*d*_4_, 298 K): δ [ppm] = 176.7 (C (5)), 120.4 (C(3a)), 119.0 (C(8a)), 116.6 (C (3)), 102.0 (C (2)), 37.0 (C (6)), 27.5 (C (8)), 21.8 (C (7)); HR-MS (ESI): (*m*/*z*) = calculated for C_8_H_11_N_2_O^+^ [*M*+H]^+^: 151.0866, found: 151.0862, C_8_H_10_N_2_ONa^+^ [*M*+Na]^+^: 173.0691, found: 173.0680.

#### Octahydropyrrolo[3,2-*b*]azepin-5(1H)-one ((±)-**15**)

To a solution of lactam **14** (380 mg, 2.53 mmol, 1.0 equiv) in AcOH (5 mL) was added Pd/C (10% Pd on activated charcoal, 38.0 mg, 10 wt % of substrate). The solution was stirred in a sealed autoclave at 100 °C under H_2_ pressure (25 bar) for 3 days. After cooling to room temperature, the reaction mixture was diluted with MeOH, and the catalyst was removed by filtration over Celite. The crude product was purified by flash column chromatography on silica gel (10%–20% MeOH in DCM + 1% NH_3_) to afford aliphatic bicycle (±)-**15** (181 mg, 1.17 mmol, 46%) as a light brown solid. R_f_ = 0.20 (20% MeOH in DCM + 1% NH_3_); ^1^H NMR (400 MHz, CDCl_3_, 298 K): δ [ppm] = 6.70 (br, 1H; H-*N* (4), 3.37 (qd, *J* = 9.0, 4.2 Hz, 1H; H-*C*(3a)), 3.05–3.11 (m, 2H; H-*C* (2)), 2.57–2.63 (m, 1H; H-*C*(8a)), 2.54 (s, 1H; H-*N* (1)), 2.44–2.48 (m, 2H; H_2_-*C* (6)), 2.28–2.37 (m, 1H; H-*C* (3)), 2.20–2.26 (m, 1H; H-*C* (8)), 1.88–1.96 (m, 1H; H-*C* (7)), 1.60–1.77 (m, 2H; H-*C* (3 and 7)), 1.29–1.39 (m, 1H; H-*C* (8)); ^13^C NMR (100 MHz, CDCl_3_, 298 K): δ [ppm] = 178.3 (C (5)), 65.2 (C(8a)), 58.0 (C(3a)), 43.7 (C (2)), 37.2 (C (6)), 35.1 (C (8)), 31.90 (C (3)), 21.9 (C (7)); HR-MS (ESI): (*m*/*z*) = calculated for C_8_H_15_N_2_O^+^ [*M*+H]^+^: 155.1179, found: 155.1176.

#### 1-Benzyloctahydropyrrolo[3,2-*b*]azepin-5(1*H*)-one ((±)-**16**)

To a solution of amine (±)-**15** (455 mg, 2.95 mmol, 1.0 equiv) in MeOH (25 mL) was added benzyl bromide (530 μL, 4.45 mmol, 1.5 equiv) and K_2_CO_3_ (629 mg, 4.55 mmol, 1.5 equiv). The mixture was stirred at 22 °C for 2 h. Then, the solvent was evaporated, and the residue was diluted with dionized H_2_O and extracted with DCM (3 × 50 mL). The organic layers were dried over Na_2_SO_4_and filtered, and the solvent was removed in vacuo to give benzylated amine (±)-**16** (682 mg, 2.79 mmol, 95%) as a colorless oil. R_f_ = 0.20 (2% MeOH in DCM + 0.1% NEt_3_); ^1^H NMR (400 MHz, MeOD-*d*_4_, 298 K): δ [ppm] = 7.24–7.31 (m, 5H; H-*C*_arom._), 4.05 (d, *J* = 12.8 Hz, 1H; H-*C*_benz._), 3.69–3.75 (m, 1H; H-*C*(3a)), 3.20 (d, *J* = 12.8 Hz, 1H; H-*C*_benz._), 2.87 (td, *J* = 9.3, 3.6 Hz, 1H; H-*C* (2)), 2.62–2.70 (m, 1H; H-*C* (6)), 2.32–2.43 (m, 3H; H-*C* (2, 6 and 8)), 2.12–2.22 (m, 1H; H-*C* (3)), 2.00–2.07 (m, 2H; H-*C*(7 and 8a)), 1.65–1.73 (m, 1H; H-*C* (3)), 1.56–1.63 (m, 1H; H-*C* (7)), 1.42–1.52 (m, 1H; H-*C* (8)); ^13^C NMR (100 MHz, MeOD-*d*_4_, 298 K): δ [ppm] = 180.6 (C (5)), 139.2 ((C_q_)_arom._), 130.4 ((*C*H)_arom._), 129.3 ((*C*H)_arom._), 128.3 ((*C*H)_arom._), 71.5 (C(8a)), 58.4 ((*C*H_2_)_benz._), 58.1 (C(3a)), 52.2 (C (2)), 37.6 (C (6)), 34.0 (C (8)), 28.1 (C (3)), 22.7 (C (7)); HR-MS (ESI): (*m*/*z*) = calculated for C_15_H_21_N_2_O^+^ [*M*+H]^+^: 245.1648, found: 245.1645.

#### 1-Benzyldecahydropyrrolo[3,2-*b*]azepine ((±)-**17b**)

A solution of lactam (±)-**16** (660 mg, 2.70 mmol, 1.0 equiv) in dry THF (27 mL, 0.1 M) was cooled to 0 °C, and LiAlH_4_ (1 M in THF, 24.3 mL, 24.3 mmol, 9.0 equiv) was added dropwise. The reaction was heated to reflux and stirred for 16 h. The reaction mixture was then cooled to room temperature and worked up using Fieser's protocol. The crude was purified by flash column chromatography on silica gel (10% MeOH in DCM + 1% NH_3_) to give azepane (±)-**17** (451 mg, 1.96 mmol, 73%) as a colorless oil. R_f_ = 0.20 (10% MeOH in DCM + 1% NH_3_); ^1^H NMR (400 MHz, MeOD-*d*_4_, 298 K): δ [ppm] = 7.26–7.36 (m, 5H; H-*C*_arom._), 4.04 (d, *J* = 12.6 Hz, 1H; H-*C*_benz._), 3.13–3.20 (m, 2H; H-*C*_benz._ and H-*C*(3a)), 2.95–3.01 (m, 1H; H-*C* (5, 6, 7 or 8)), 2.80–2.87 (m, 2H; H-*C* (2 and 5, 6, 7 or 8)), 2.36 (q, *J* = 9.4 Hz, 1H; H-*C* (2)), 2.22–2.30 (m, 2H; H-*C*(8a and H-*C* (5, 6, 7 or 8)), 2.09–2.17 (m, 1H; H-*C* (3)), 1.82–1.89 (m, 1H; H-*C* (5, 6, 7 or 8)), 1.69–1.79 (m, 3H; H-*C* (5, 6, 7 or 8)), 1.39–1.48 (m, 2H; H-*C* (3 and 5, 6, 7 or 8); ^13^C NMR (100 MHz, MeOD-*d*_4_, 298 K): δ [ppm] = 138.9 ((C_q_)_arom._), 130.7 ((*C*H)_arom._), 129.3 ((*C*H)_arom._), 128.3 ((*C*H)_arom._), 72.4 (C(8a)), 62.5 (C(3a)), 59.2 (*C*H_2_)_benz._), 52.9 (C (2)), 49.6 (C (5, 6, 7 or 8)), 31.1 (C (5, 6, 7 or 8)), 30.6 (C (3)), 28.9 (C (5, 6, 7 or 8)), 26.1 (C (5, 6, 7 or 8)); HR-MS (ESI): (*m*/*z*) = calculated for C_15_H_23_N_2_^+^ [*M*+H]^+^: 231.1856, found: 231.1854.

#### Decahydropyrrolo[3,2-*b*]azepine ((±)-**2b**)

To a solution of monobenzylated diamine (±)-**17** (147 mg, 0.64 mmol, 1.0 equiv) in MeOH (7 mL, 0.1 M) was added Pd/C (10% Pd on activated charcoal, 15.2 mg, 10 wt % of substrate) and AcOH (75 μL, 1.30 mmol, 2.0 equiv). The reaction mixture was stirred at 22 °C under an atmosphere of hydrogen (1 bar, balloon) for 24 h. It was then filtered over Celite, washed with MeOH and evaporated to dryness. The residue was taken up in DCM and washed with NaOH (1 M, 2 × 4 mL) and the organic phase was dried over Na_2_SO_4_ to give final free diamine (±)-**2b** as a yellow oil (90.0 mg, 0.64 mmol, quant.). ^1^H NMR (400 MHz, MeOD-*d*_4_, 298 K): δ [ppm] = 2.88–3.01 (m, 4H), 2.79–2.85 (m, 1H), 2.67–2.73 (m, 1H), 2.13–2.22 (m, 1H), 2.04–2.11 (m, 1H), 1.63–1.82 (m, 4H), 1.52–1.61 (m, 1H), 1.29–1.40 (m, 1H); ^13^C NMR (100 MHz, MeOD-*d*_4_, 298 K): δ [ppm] = 66.8 (*C*H), 64.2 (*C*H), 49.3 (*C*H_2_), 44.9 (*C*H_2_), 34.5 (*C*H_2_), 32.9 (*C*H_2_), 29.2 (*C*H_2_), 25.8 (*C*H_2_); HR-MS (ESI): (*m*/*z*) = calculated for C_8_H_17_N_2_^+^ [*M*+H]^+^: 141.1386, found: 141.1385.

#### (*E*)-7,8-Dihydroquinolin-5(6H)-one oxime (**19**)^73^

To a solution of commercially available 7,8-Dihydroquinolin-5(6*H*)-one **18** (4.90 g, 33.3 mmol, 1.0 equiv) in MeOH (27 mL) and dionized H_2_O (9 mL) was added NH_2_OH·HCl (7.09 g, 102 mmol, 3.0 equiv) and sodium acetate (13.7 g, 167 mmol, 5.0 equiv). The mixture was refluxed for 4 h. The reaction was allowed to cool to room temperature and concentrated under reduced pressure. The formed solid was collected by vacuum filtration and washed with dionized H_2_O to give *(E)*-oxime isomer **19** (5.32 g, 32.8 mmol, 98%) as a white solid. R_f_ = 0.24 (60% EtOAc in heptane); ^1^H NMR (400 MHz, MeOD-*d*_4_, 298 K): δ [ppm] = 8.37 (dd, *J* = 4.9, 1.7 Hz, 1H; H-*C* (2)), 8.31 (dd, *J* = 8.0, 1.7 Hz, 1H; H-*C* (4)), 7.25 (dd, *J* = 8.0, 4.8 Hz, 1H; H-*C* (3)), 2.92 (t, *J* = 6.2 Hz, 2H; H_2_-*C* (8)), 2.79 (t, *J* = 6.6 Hz, 2H; H_2_-*C* (6)), 1.93 (quint., *J* = 6.4 Hz, 2H; H_2_-*C* (7)); ^13^C NMR (100 MHz, MeOD-*d*_4_, 298 K): δ [ppm] = 159.2 (C(8a)), 153.3 (C (5)), 149.6 (C (2)), 133.6 (C (4)), 129.2 (C(4a)), 123.3 (C (3)), 33.0 (C (8)), 23.9 (C (6)), 21.7 (C (7)); HR-MS (ESI): (*m*/*z*) = calculated for C_9_H_11_N_2_O^+^ [*M*+H]^+^: 163.0866, found: 163.0866.

#### (*E*)-7,8-Dihydroquinolin-5(6H)-one O-tosyl oxime (**20**)^74^

To a solution of oxime **19** (1.51 g, 9.31 mmol, 1.0 equiv) in acetone (70 mL) and dionized H_2_O (29 mL) was added freshly recrystallized *p*-TsCl (2.67 g, 14.0 mmol, 1.5 equiv) and KOH (0.52 g, 9.31 mmol, 1.0 equiv). After heating the mixture to reflux for 2 h, it was cooled down, concentrated under reduced pressure and the resulting solid was taken up in dionized H_2_O (50 mL). The aqueous phase was extracted with EtOAc (3 × 100 mL). The organic layers were washed with Na_2_CO_3_ (100 mL), dried over Na_2_SO_4_, and filtered, and the solvent was removed in vacuo to obtain tosylate **20** (1.90 g, 6.01 mmol, 65%) as a white solid. R_f_ = 0.22 (60% EtOAc in heptane); ^1^H NMR (400 MHz, CDCl_3_, 298 K): δ [ppm] = 8.55 (dd, *J* = 4.8, 1.7 Hz, 1H; H-*C* (2 or 4)), 8.15 (dd, *J* = 8.0, 1.5 Hz, 1H; H-*C* (2 or 4)), 7.93 (d, *J* = 8.3 Hz, 2H; H-*C*_tos._), 7.36 (d, *J* = 8.2 Hz, 2H; H-*C*_tos._), 7.17 (dd, *J* = 8.1, 4.8 Hz, 1H; H-*C* (3)), 2.96 (t, *J* = 6.2 Hz, 2H; H_2_-*C* (8)), 2.86 (t, *J* = 6.6 Hz, 2H; H_2_-*C* (6)), 2.45 (s, 3H; H_3_-*C*_tos._), 1.94 (quint., *J* = 6.5 Hz, 2H; H_2_-*C* (7)); ^13^C NMR (100 MHz, CDCl_3_, 298 K): δ [ppm] = 161.5 (C (5)), 159.9 (C(8a)), 151.8 (C (2 or 4)), 145.4 ((C_q_)_tos._), 133.2 (C (2 or 4)), 132.7 ((C_q_)_tos._), 129.8 ((*C*H)_tos._), 129.1 ((*C*H)_tos._), 124.4 (C(4a)), 122.1 (C (3)), 32.3 (C (8)), 25.0 (C (6)), 21.9 ((*C*H_3_)_tos._), 20.5 (C (7)), HR-MS (ESI): (*m*/*z*) = calculated for C_16_H_17_N_2_O_3_S^+^ [*M*+H]^+^: 317.0954, found: 317.0947, C_16_H_16_N_2_O_3_SNa^+^ [*M*+Na]^+^: 339.0779, found: 339.0765.

#### 5,7,8,9-Tetrahydro-6*H*-pyrido[3,2-*b*]azepin-6-one (**21**)^74^

Tosylate **20** (2.69 g, 8.50 mmol, 1.0 equiv) was dissolved in EtOH (40 mL) and dionized H_2_O (80 mL), potassium acetate (2.56 g, 25.6 mmol, 3.0 equiv) was added, and the mixture was refluxed for 24 h. After cooling to room temperature and evaporation of the EtOH, the remaining aqueous solution was adjusted to pH = 10 by the addition of aqueous NaOH solution (1 M, 10 mL). The aqueous phase was extracted with chloroform (3 × 150 mL). The combined organic layers were dried over Na_2_SO_4_ and filtered, and the solvent was removed under reduced pressure to yield lactam **21** (1.25 g, 7.70 mmol, 91%) as a light brown solid. R_f_ = 0.25 (80% EtOAc in heptane); ^1^H NMR (400 MHz, MeOD-*d*_4_, 298 K): δ [ppm] = 8.28 (dd, *J* = 4.9, 1.5 Hz, 1H; H-*C* (2 or 4), 7.45 (dd, *J* = 8.0, 1.5 Hz, 1H; H-*C* (2 or 4)), 7.34 (dd, *J* = 8.0, 4.9 Hz, 1H; H-*C* (3)), 2.98–3.02 (m, 2H; H_2_-*C* (9)) 2.32–2.35 (m, 4H; H_2_-*C* (7 and 8)); ^13^C NMR (100 MHz, MeOD-*d*_4_, 298 K): δ [ppm] = 176.7 (C (6)), 155.6 (C(9a)), 146.4 (C (2 or 4)), 136.6 (C(4a)), 131.1 (C (2 or 4)), 124.1 (C (3)), 33.8 (C (7 or 8)), 33.5 (C (9)), 28.7 (C (7 or 8)); HR-MS (ESI): (*m*/*z*) = calculated for C_9_H_11_N_2_O^+^ [*M*+H]^+^: 163.0866, found: 163.0864, C_9_H_10_N_2_ONa^+^ [*M*+Na]^+^: 185.0691, found: 185.0683.

#### 6,7,8,9-Tetrahydro-5*H*-pyrido[3,2-*b*]azepine (**22**)^74^

A solution of lactam **21** (1.25 g, 7.70 mmol, 1.0 equiv) in dry THF (31 mL) was cooled to 0 °C. Then, LiAlH_4_ (1 M in THF, 69.4 mL, 69.4 mmol, 9.0 equiv) was added dropwise. The mixture was stirred at reflux for 6 h. The reaction was cooled down and worked up using Fieser's protocol. The crude product was purified by flash column chromatography on silica gel (2% MeOH in DCM + 0.1% NEt_3_) to afford azepane **22** (0.92 g, 6.21 mmol, 81%) as a white solid. R_f_ = 0.25 (2% MeOH in DCM + 0.1% NEt_3_); ^1^H NMR (400 MHz, MeOD-*d*_4_, 298 K): δ [ppm] = 7.88 (dd, *J* = 4.8, 1.5 Hz, 1H; H-*C* (4)), 7.23 (dd, *J* = 8.0, 1.3 Hz, 1H; H-*C* (2)), 7.06 (dd, *J* = 8.0, 4.8 Hz, 1H; H-*C* (3)), 3.02–3.05 (m, 2H; H_2_-*C* (6)), 2.94–2.97 (m, 2H; H_2_-*C* (9)), 1.80–1.85 (m, 2H; H_2_-*C* (7)), 1.67–1.73 (m, 2H; H_2_-*C* (8)); ^13^C NMR (100 MHz, MeOD-*d*_4_, 298 K): δ [ppm] = 154.3 (C(9a)), 148.6 (C(4a)), 140.7 (C (4)), 128.0 (C (2)) 123.2 (C (3)), 49.1 (C (6)), 38.8 (C (9)), 32.4 (C (7)), 26.2 (C (8)); HR-MS (ESI): (*m*/*z*) = calculated for C_9_H_13_N_2_^+^ [*M*+H]^+^: 149.1073, found: 149.1075.

#### Trifluoro-1-(6,7,8,9-tetrahydro-5*H*-pyrido[3,2-*b*]azepin-5-yl)ethanone (**23**)

To a solution of amine **22** (1.58 g, 10.7 mmol, 1.0 equiv) in DCM (164 mL) was added TFAA (1.78 mL, 12.8 mmol, 1.2 equiv) and pyridine (1.12 mL, 13.9 mmol, 1.3 equiv). The mixture was stirred at 22 °C for 2 h. The organic phase was washed with dionized H_2_O (100 mL) and brine (100 mL), dried over Na_2_SO_4_, and filtered, and the solvent was removed in vacuo to give protected amine **23** (2.34 g, 9.58 mmol, 90%) as a light yellow solid. R_f_ = 0.18 (20% EtOAc in heptane); ^1^H NMR (400 MHz, MeOD-*d*_4_, 298 K): δ [ppm] = 8.46 (dd, *J* = 4.9, 1.4 Hz, 1H; H-*C* (2 or 4); 7.75–7.78 (m, 1H; H-*C* (2 or 4), 7.38 (dd, *J* = 7.9, 4.9 Hz, 1H; H-*C* (3), 4.62–4.67 (m, 1H; H-*C* (6)), 2.98–3.10 (m, 2H; H_2_-*C* (9)), 2.85–2.92 (m, 1H; H-*C* (6)), 1.97–2.10 (m, 2H; H-*C*(7/8)), 1.88–1.94 (m, 1H; H-*C* (7)), 1.48–1.59 (m, 1H; H-*C* (8)); ^13^C NMR (100 MHz, MeOD-*d*_4_, 298 K): δ [ppm] = 162.0 (C(9a)), 156.6 (q, *J*_C–F_ = 35.7 Hz, *C*OCF_3_), 150.0 (C (2 or 4)), 138.0 (C(4a)), 137.4 (C (2 or 4)), 123.7 (C (3)), 117.6 (q, *J*_C–F_ = 287.9 Hz, CO*C*F_3_), 50.8 (C (6)), 37.4 (C (9)), 29.6 (C (7)), 25.7 (C (8)); ^19^F-NMR (300 MHz, MeOD-*d*_4_, 298 K): δ [ppm] = −69.6; HR-MS (ESI): (*m*/*z*) = calculated for C_11_H_12_N_2_OF_3_^+^ [*M*+H]^+^: 245.0896, found: 245.0897.

#### 1-(Decahydro-5*H*-pyrido[3,2-*b*]azepin-5-yl)-trifluoroethanone ((±)-**24**)

To a solution of protected amine **23** (1.34 g, 5.49 mmol, 1.0 equiv) in *^i^*PrOH (28 mL) was added Rh/C (5%, Rh on activated charcoal, 134 mg, 10 wt % of substrate). The mixture was stirred in a sealed autoclave at 70 °C under H_2_ pressure (10 bar) for 24 h. After cooling to room temperature, the reaction mixture was diluted with MeOH, and the catalyst was removed by filtration over Celite. The filtrate was concentrated to yield the aliphatic bicycle (±)-**24** (1.35 g, 5.39 mmol, 98%) as a yellow solid. R_f_ = 0.17 (5% MeOH in DCM + 0.1% NEt_3_); ^1^H NMR (400 MHz, MeOD-*d*_4_, 298 K): δ [ppm] = 4.40 (quint., *J* = 4.1 Hz, 1H), 4.07–4.12 (m, 0.6H), 3.90–3.96 (m, 1.6H), 3.73–3.77 (m, 1H), 3.41–3.48 (m, 0.6H), 3.09–3.13 (m, 1H), 2.99–3.03 (m, 0.7H), 2.91–2.97 (m, 1H), 2.65–2.80 (m, 2H), 2.00–2.26 (m, 3H), 1.78–1.95 (m, 4H), 1.53–1.75 (m, 7H), 1.29–1.44 (m, 2H); ^13^C NMR (100 MHz, MeOD-*d*_4_, 298 K): δ [ppm] = 158.3 (q, *J*_C–F_ = 36.0 Hz, *C*OCF_3_), 118.3 (q, *J*_C–F_ = 287.3 Hz, CO*C*F_3_), 60.0 (*C*H)_rot._, 57.4 (*C*H)_rot._, 57.0 (*C*H)_rot._, 56.7 (*C*H)_rot._, 43.7 (*C*H_2_)_rot._, 43.6 (*C*H_2_)_rot._, 41.9 (*C*H_2_)_rot._, 41.3 (*C*H_2_)_rot._, 31.2 (*C*H_2_)_rot._, 30.7 (*C*H_2_)_rot._, 28.5 (*C*H_2_)_rot._, 27.1 (*C*H_2_)_rot._, 26.4 (*C*H_2_)_rot._, 26.3 (*C*H_2_)_rot._, 25.5 (*C*H_2_)_rot._, 25.3 (*C*H_2_)_rot._, 23.1 (*C*H_2_)_rot._, 22.6 (*C*H_2_)_rot._; ^19^F-NMR (300 MHz, MeOD-*d*_4_, 298 K): δ [ppm] = −68.2, −68.3; due to the presence of trifluoroacetamide rotamers in the NMR measurement, structural assignment is not possible; HR-MS (ESI): (*m*/*z*) = calculated for C_11_H_18_N_2_OF_3_^+^ [*M*+H]^+^: 251.1366, found: 251.1367, C_11_H_17_N_2_OF_3_Na^+^ [*M*+Na]^+^: 273.1191, found: 273.1186.

#### Decahydro-1*H*-pyrido[3,2-*b*]azepine ((±)-**3a**)

Monotrifluoroacetamide-protected diamine (±)-**24** (100 mg, 0.40 mmol, 1.0 equiv) was dissolved in a mixture of THF and dionized H_2_O (4 mL, 1:1, 0.1 M), and LiOH (48.0 mg, 2.00 mmol, 5.0 equiv) was added. The mixture was refluxed for 24 h. Then it was cooled to room temperature and concentrated under reduced pressure. The aqueous phase was extracted with EtOAc (3 × 10 mL). The organic layers were dried over Na_2_SO_4_and filtered, and the solvent was removed in vacuo to obtain the final free diamine (±)-**3a** (47.7 mg, 0.31 mmol, 78%) as a brown solid. ^1^H NMR (400 MHz, MeOD-*d*_4_, 298 K): δ [ppm] = 3.01–3.07 (m, 1H); 2.94–2.99 (m, 1H), 2.87 (td, *J* = 6.5, 2.9 Hz, 1H), 2.77–2.80 (m, 1H), 2.58–2.67 (m, 2H), 1.92–1.99 (m, 1H), 1.35–1.85 (m, 9H); ^13^C NMR (100 MHz, MeOD-*d*_4_, 298 K): δ [ppm] = 59.0 (*C*H), 57.4 (*C*H), 49.7 (*C*H_2_), 46.7 (*C*H_2_), 35.0 (*C*H_2_), 32.9 (*C*H_2_), 32.4 (*C*H_2_), 23.2 (*C*H_2_), 22.3 (*C*H_2_); HR-MS (ESI): (*m*/*z*) = calculated for C_9_H_19_N_2_^+^ [*M*+H]^+^: 155.1543, found: 155.1536.

#### Decahydro-6*H*-pyrido[3,2-*b*]azepin-6-one (**25**)

To a solution of lactam **21** (2.02 g, 12.5 mmol, 1.0 equiv) in AcOH (25 mL) was added Pd/C (10% Pd on activated charcoal, 200 mg, 10 wt % of substrate). The solution was stirred in a sealed autoclave at 100 °C under H_2_ pressure (20 bar) for 2 days. After cooling to room temperature, the reaction mixture was diluted with MeOH, and the catalyst was removed by filtration over Celite. The crude product was purified by flash column chromatography on silica gel (5% MeOH in DCM + 1% NH3) to afford aliphatic bicycle (±)-**25** (676 mg, 4.02 mmol, 32%) as a white solid. Rf = 0.20 (5% MeOH in DCM + 1% NH3); ^1^H NMR (400 MHz, MeOD-*d*_4_, 298 K): δ [ppm] = 4.66–4.70 (m, 1H; H-*C* (2), 3.50–3.54 (m, 1H; H-*C*(4a)), 3.07–3.08 (m, 1H; H-*C*(8a)), 2.49–2.56 (m, 1H; H-*C* (2)), 2.33–2.35 (m, 2H; H-*C* (7)), 1.84–1.94 (m, 3H; H-*C* (3, 4, 8 or 9)), 1.70–1.83 (m, 3H; H-*C* (3, 4, 8 or 9)), 1.58–1.68 (m, 2H; H-*C* (3 or 9)); ^13^C NMR (100 MHz, MeOD-*d*_4_, 298 K): δ [ppm] = 173.6 (C (6)), 60.5 (C(4a)), 50.5 (C(8a)), 43.2 (C (2)), 33.7 (C (7)), 31.6 (C (4 or 8)), 27.2 (C (4 or 8)), 20.2 (C (3 or 9)), 19.8 (C (3 or 9)); HR-MS (ESI): (*m*/*z*) = calculated for C9H17N2O^+^ [*M*+H]^+^: 169.1335, found: 169.1339.

#### 1-Benzyldecahydro-6*H*-pyrido[3,2-*b*]azepin-6-one (**26**)

To a solution of amine (±)-**25** (98 mg, 0.59 mmol, 1.0 equiv) in MeOH (5.8 mL) was added benzyl bromide (83 μL, 0.70 mmol, 1.2 equiv) and K_2_CO_3_ (120 mg, 0.87 mmol, 1.5 equiv). The mixture was stirred at 22 °C for 22 h. Then, the solvent was evaporated, and the residue was diluted with dionized H_2_O and extracted with EtOAc (3 × 20 mL). The organic layers were dried over Na_2_SO_4_ and filtered, and the solvent was removed in vacuo. The crude product was purified by flash chromatography on silica gel (80% EtOAc in heptane) to afford benzylated amine (±)-**26** (99 mg, 0.38 mmol, 64%) as a colorless oil. Rf = 0.22 (80% EtOAc in heptane); ^1^H NMR (400 MHz, MeOD-d4, 298 K): δ [ppm] = 7.35–7.37 (m, 2H; H_2_-*C*arom.), 7.28–7.31 (m, 2H; H_2_-*C*arom.), 7.20–7.24 (m, 1H; H-*C*arom.), 4.61–4.65 (m, 1H; H-*C* (2)), 3.88 (d, *J* = 13.4 Hz, 1H; H-*C*benz.,), 3.68 (d, *J* = 13.4 Hz, 1H; H-*C*benz.), 3.42–3.46 (m, 1H; H-*C*(4a)), 2.65–2.67 (m, 1H; H-*C*(9a)), 2.47–2.54 (m, 1H; H-*C* (2)), 2.26–2.32 (m, 2H; H-*C* (7)), 2.04–2.16 (m, 2H; H-*C* (4 and 9)), 1.95–1.99 (m, 1H; H-*C* (8)), 1.70–1.81 (m, 2H; H-*C* (3 and 4)), 1.43–1.63 (m, 3H; H-*C* (3, 8 and 9)); ^13^C NMR (100 MHz, MeOD-d4, 298 K): δ [ppm] = 173.5 (C (6)), 142.0 ((Cq)arom.), 1294 ((*C*H)arom.), 129.3 ((*C*H)arom.), 127.9 ((*C*H)arom.), 61.7 (C(4a)), 55.6 C(9a)), 52.3 ((*C*H2)benz.), 43.6 (C (2)), 33.7 (C (7)), 28.0 (C (9)), 27.0 (C (4)), 20.3 (C (3 or 8)), 20.2 (C (3 or 8)); HR-MS (ESI): (*m*/*z*) = calculated for C16H23N2O^+^ [*M*+H]^+^: 259.1805, found: 259.1805.

#### 1-Benzyldecahydro-1*H*-pyrido[3,2-*b*]azepine (**27b**)

A solution of lactam (±)-**26** (220 mg, 0.85 mmol, 1.0 equiv) in dry THF (8.5 mL, 0.1 M) was cooled to 0 °C and LiAlH_4_ (1 M in THF, 1.3 mL, 1.3 mmol, 1.5 equiv) was added dropwise. The reaction was stirred at 22 °C for 16 h. The reaction mixture was then cooled to room temperature and worked up using Fiesers protocol. The crude was purified by flash column chromatography on silica gel (5% MeOH in DCM + 1% NH3) to give azepane (±)-**27b** (164 mg, 0.67 mmol, 79%) as a colorless oil. Rf = 0.18 (5% MeOH in DCM + 1% NH3); ^1^H NMR (400 MHz, MeOD-d4, 298 K): δ [ppm] = 7.35–7.37 (m, 2H; H_2_-*C*arom.), 7.27–7.31 (m, 2H; H_2_-*C*arom.), 7.19–7.23 (m, 1H; H-*C*arom.), 3.86 (d, *J* = 13.2 Hz, 1H; H-*C*benz.), 3.66 (m, *J* = 13.2 Hz, 1H; H-*C*benz.), 2.79–2.82 (m, 2H; H-*C* (2, 3, 4, 6, 7, 8)), 2.60 (m, 1H; H-*C*(9a)), 1.96–2.08 (m, 4H; H-*C* (2, 3, 4, 6, 7, 8)), 1.77–1.91 (m, 3H; H-*C*(4a and H-*C* (2, 3, 4, 6, 7, 8)), 1.54–1.59 (m, 2H; H-*C* (2, 3, 4, 6, 7, 8)), 1.44–1.50 (m, 1H; H-*C* (2, 3, 4, 6, 7, 8)), 1.30–1.40 (m, 3H; H-*C* (9 and 2, 3, 4, 6, 7, 8)); ^13^C NMR (100 MHz, MeOD-d4, 298 K): δ [ppm] = 142.0 ((Cq)arom.), 129.3 ((*C*H)arom.), 129.2 ((*C*H)arom.), 127.8 ((*C*H)arom.), 2X 58.0 (C(8a and 2, 3, 4, 6, 7 or 8)), 56.4 (C(9a)), 52.3 (*C*H2)benz.), 2X 28.4 (C (9 and 2, 3, 4, 6, 7 or 8)), 26.0 (C (2, 3, 4, 6, 7 or 8)), 2X 25.6 (C (2, 3, 4, 6, 7 or 8)), 21.2 (C (2, 3, 4, 6, 7 or 8)); HR-MS (ESI): (*m*/*z*) = calculated for C16H25N2^+^ [*M*+H]^+^: 245.2012, found: 245.2011.

#### Decahydro-1*H*-pyrido[3,2-*b*]azepine ((±)-**3b**)

To a solution of monobenzylated diamine (±)-**27b** (50 mg, 0.20 mmol, 1.0 equiv) in MeOH (2 mL, 0.1 M) was added Pd/C (10% Pd on activated charcoal, 5.0 mg, 10 wt % of substrate) and AcOH (23 μL, 0.04 mmol, 2.0 equiv). The reaction mixture was stirred at 22 °C under an atmosphere of hydrogen (1 bar, balloon) for 24 h. It was then filtered over Celite, washed with MeOH, and evaporated to dryness. The residue was taken up in DCM and washed with NaOH (1 M, 2 × 4 mL), and the organic phase was dried over Na_2_SO_4_ to give final free diamine (±)-**3b** as a yellow oil (30.9 mg, 0.20 mmol, quant.). ^1^H NMR (400 MHz, MeOD-*d*_4_, 298 K): δ [ppm] = 2.77–2.86 (m, 2H), 1.98–2.10 (m, 2H), 1.75–1.86 (m, 3H), 1.45–1.65 (m, 6H), 1.29–1.41 (m, 4H); ^13^C NMR (100 MHz, MeOD-*d*_4_, 298 K): δ [ppm] = 66.8 (*C*H), 57.9 (*C*H_2_), 50.9 (*C*H), 32.5 (*C*H_2_), 30.8 (*C*H_2_), 30.3 (*C*H_2_), 26.5 (*C*H_2_), 25.4 (*C*H_2_), 20.6 (*C*H_2_); HR-MS (ESI): (*m*/*z*) = calculated for C_9_H_19_N_2_^+^ [*M*+H]^+^: 155.1543, found: 155.1542

#### (*E*)-5-Methoxy-3,4-dihydronaphthalen-1(2*H*)-one Oxime (**29**)^75^

To a solution of commercially available 5-methoxy-3,4-dihydronaph-thalen-1(2H)-one **28** (25 g, 142 mmol, 1.0 equiv) in EtOH (285 mL, 0.2 M) was added NH_2_OH•HCl (23.7 g, 341 mmol, 2.4 equiv) and grinded NaOH (30 g, 750 mmol, 5.3 equiv). The mixture was stirred under reflux for 2 h. The mixture was allowed to cool to room temperature, and the solvent was evaporated. Dionized water (300 mL) was added, and the mixture was extracted with chloroform (3 × 300 mL). The combined organic layers were dried over Na_2_SO_4_and filtered, and the solvent was removed in vacuo to give *(E)*-oxime isomer **29** (27.1 g, 142 mmol, quant.) as a white solid. R_f_ = 0.21 (10% EtOAc in heptane); ^1^H NMR (400 MHz, MeOD-*d*_4_, 298 K): δ [ppm] = 7.50 (d, *J* = 8.0, 1H; H-*C* (8)), 7.11 (t, *J* = 8.1 Hz, 1H; H-*C* (7)), 6.87 (d, *J* = 8.0, 1H; H-*C* (6)), 3.81 (s, 3H; H_3_-*C*), 2.68–2.74 (m, 4H; H_2_-*C* (2 and 4)), 1.79 (quint., *J* = 6.4 Hz, 2H; H_2_-*C* (3)); ^13^C NMR (100 MHz, MeOD-*d*_4_, 298 K): δ [ppm] = 158.1 (C (5)), 155.6 (C (1)), 133.4 (C(8a)), 129.5 (C(4a)), 127.3 (C (7)), 117.2 (C (8)), 111.0 (C (6)), 55.9 (*C*H_3_), 24.1 (C (2 or 4)), 23.3 (C (2 or 4)), 22.2 (C (3)); HR-MS (ESI): (*m*/*z*) = calculated for C_11_H_14_NO_2_^+^ [*M*+H]^+^: 192.1019, found: 192.1010.

#### (*E*)-5-Methoxy-3,4-dihydronaphthalen-1(2*H*)-one-*O*-tosyl Oxime (**30**)^76^

To a solution of oxime **29** (27.1 g, 142 mmol, 1.0 equiv) in pyridine (285 mL, 0.5 M) was added freshly recrystallized *p*-TsCl (32.4 g, 170 mmol, 1.2 equiv), and the mixture was stirred at 22 °C for 24 h. Then, the pyridine was evaporated, and the residue was taken up in dionized H_2_O (250 mL) and extracted with DCM (3 × 250 mL). The organic layers were washed with HCl (1 M, 250 mL) and sat. NaHCO_3_ (250 mL), dried over Na_2_SO_4_, filtered, and evaporated to yield tosylate **30** (49.2 g, 142 mmol, quant.) as a yellow solid. R_f_ = 0.22 (60% EtOAc in heptane); ^1^H NMR (400 MHz, CDCl_3_, 298 K): δ [ppm] = 7.94 (d, *J* = 8.3 Hz, 2H; H-*C*_tos._), 7.48 (d, *J* = 7.9 Hz, 1H; H-*C* (8)), 7.35 (d, *J* = 8.1 Hz, 2H; H-*C*_tos._), 7.14 (t, *J* = 8.1 Hz, 1H; H-*C* (7)), 6.86 (d, *J* = 8.1 Hz, 1H; H-*C* (6)), 3.81 (s, 3H; H_3_-*C*), 2.79 (t, *J* = 6.7 Hz, 2H; H_2_-*C* (2)), 2.69 (t, *J* = 6.2 Hz, 2H; H_2_-*C* (4)), 2.44 (s, 3H; H_3_-*C*_tos._), 1.80 (quint., *J* = 6.4 Hz, 2H; H_2_-*C* (3)); ^13^C NMR (100 MHz, CDCl_3_, 298 K): δ [ppm] = 162.7 (C (1)) 156.8 (C (5)), 145.0 ((C_q_)_tos._), 133.0 ((C_q_)_tos._), 130.5 (C(4a)), 129.7 ((*C*H)_tos._), 129.2 (C(8a)), 129.1 ((*C*H)_tos._), 126.8 (C (7)), 117.3 (C (8)), 112.0 (C (6)), 55.7 (*C*H_3_), 25.0 (C (2)), 22.1 (C (4)), 21.8 ((*C*H_3_)_tos._), 20.6 (C (3)); HR-MS (ESI): (*m*/*z*) = calculated for C_18_H_20_NO_4_S^+^ [*M*+H]^+^: 346.1108, found: 346.1100.

#### 6-Methoxy-1,3,4,5-tetrahydro-2*H*-benzo[*b*]azepin-2-one (**31**)^77^

To tosylate **30** (49.0 g, 142 mmol, 1.0 equiv) in dionized H_2_O (600 mL) was added AcOH (760 mL), and the mixture was stirred at 70 °C for 24 h. Then, the solvent was evaporated, and the remaining brown oil was cooled to 0 °C and quenched by the addition of NaHCO_3_ (500 mL) and Na_2_CO_3_ (600 mL). Then the aqueous phase was extracted with EtOAc (3 × 400 mL) and the organic phases were dried over Na_2_SO_4_and filtered, and the solvent was evaporated to yield lactam **31** (20.2 g, 106 mmol, 75%) as a brown solid. R_f_ = 0.24 (50% EtOAc in heptane); ^1^H NMR (400 MHz, MeOD-*d*_4_, 298 K): δ [ppm] = 7.18 (t, *J* = 8.1, 1H; H-*C* (8)), 6.83 (d, *J* = 8.3 Hz, 1H; H-*C* (7)), 6.64 (d, *J* = 2.9, 1H; H-*C* (9)), 3.83 (s, 3H; H_3_-*C*), 2.83 (t, *J* = 7.1 Hz, 2H; H_2_-*C* (5)), 2.24–2.28 (m, 2H; H_2_-*C* (3)), 2.13–2.19 (m, 2H; H_2_-*C* (4)); ^13^C NMR (100 MHz, MeOD-*d*_4_, 298 K): δ [ppm] = 177.7 (C (2)), 158.9 (C (6)), 140.7 (C(9a)), 128.5 (C (8)), 123.7 (C(5a)), 115.6 (C (9)), 109.0 (C (7)), 56.3 (*C*H_3_), 34.0 (C (3)), 29.3 (C (4)), 22.6 (C (5)); HR-MS (ESI): (*m*/*z*) = calculated for C_11_H_14_NO_2_^+^ [*M*+H]^+^: 192.1019, found: 192.1019.

#### 6-Hydroxy-1,3,4,5-tetrahydro-2*H*-benzo[b]azepin-2-one (**32**)^78^

To a solution of lactam **31** (5.00 g, 26.1 mmol, 1.0 equiv) in dry DCM (260 mL, 0.1 M) was added BBr_3_ (1 M in DCM, 40 mL, 40 mmol, 1.5 equiv) at −78 °C. The mixture was warmed to 22 °C and stirred for 24 h. Then, it was cooled to 0 °C and quenched by the addition of dionized H_2_O (60 mL). The precipitate was filtered, washed with dionized H_2_O, dried, and purified by flash column chromatography on silica gel (EtOAc to 10% MeOH in EtOAc) to yield alcohol **32** (3.90 g, 22.0 mmol, 84%) as a light brown solid. R_f_ = 0.24 (EtOAc); ^1^H NMR (400 MHz, MeOD-*d*_4_, 298 K): δ [ppm] = 7.02 (t, *J* = 8.0, 1H; H-*C* (8)), 6.66 (dd, *J* = 8.2, 1.0, 1H; H-*C* (7 or 9)), 6.52 (dd, *J* = 7.8, 0.8 Hz, 1H; H-*C* (7 or 9)), 2.81 (t, *J* = 7.1 Hz, 2H; H_2_-*C* (5)), 2.25–2.29 (m, 2H; H_2_-*C* (3)), 2.12–2.20 (m, 2H; H_2_-*C* (4)); ^13^C NMR (100 MHz, MeOD-*d*_4_, 298 K): δ [ppm] = 177.8 (C (2)), 156.5 (C(6 or 9a)), 140.8 (C(6 or 9a)), 128.2 (C (8)), 122.1 (C(5a)), 114.3 (C (7 or 9)), 113.4 (C (7 or 9)), 34.0 (C (3)), 29.2 (C (4)), 22.7 (C (5)); HR-MS (ESI): (*m*/*z*) = calculated for C_10_H_12_NO_2_^+^ [*M*+H]^+^: 178.0863, found: 178.0857, C_10_H_11_NO_2_Na^+^ [*M*+Na]^+^: 200.0687, found: 200.0676.

6-Hydroxydecahydro-2*H*-benzo[b]azepin-2-one ((±)-**33**): A mixture of aromatic compound **32** (1.53 g, 8.63 mmol, 1.0 equiv), Rh/C (5%, Rh on activated charcoal, 153 mg, 10 wt % of substrate) and AcOH (1.0 mL, 17.3 mmol, 2.0 equiv) in *^i^*PrOH (50 mL) was stirred in a sealed autoclave at 70 °C under H_2_ pressure (20 bar) for 5 days. After cooling to room temperature, the reaction mixture was filtered over Celite, washed with MeOH, and evaporated to dryness. The crude was purified by flash column chromatography on silica gel (80% EtOAc in heptane to EtOAc to 10% MeOH in EtOAc) to afford aliphatic bicycle (±)-**33** (684 mg, 3.73 mmol, 43%) as a yellow oil. R_f_ = 0.20 (80% EtOAc in heptane); ^1^H NMR (400 MHz, MeOD-*d*_4_, 298 K): δ [ppm] = 3.70–3.75 (m, 1H; H-*C* (6)), 3.13–1.16 (m, 1H; H-*C*(9a)), 2.42–2.54 (m, 2H; H_2_-*C* (3)), 1.90–2.05 (m, 4H; H-*C*(4, 5a, 5 and 9)), 1.72–1.85 (m, 2H; H-*C* (5 and 8)), 1.45–1.61 (m, 4H; H-*C* (4, 7 and 9)), 1.29–1.42 (m, 1H; H-*C* (8)); ^13^C NMR (100 MHz, MeOD-*d*_4_, 298 K): δ [ppm] = 180.6 (C (2)), 73.1 (C (6)), 54.6 (C(9a)), 46.5 (C(5a)), 38.2 (C (3)), 29.5 (C (7)), 27.9 (C (4, 5 or 9)), 24.7 (C (4, 5 or 9)), 23.6 (C (4, 5 or 9)), 22.8 (C (8)); HR-MS (ESI): (*m*/*z*) = calculated for C_10_H_18_NO_2_^+^ [*M*+H]^+^: 184.1332, found: 184.1326.

#### Octahydro-1*H*-benzo[b]azepine-2,6-dione ((±)-**34**)

A solution of alcohol (±)-**33** (1.10 g, 6.00 mmol, 1.0 equiv) in DCM (60 mL, 0.1 M) was cooled to 0 °C and DMP (3.30 g, 7.78 mmol, 1.3 equiv) was added. The reaction was stirred at 22 °C for 1 h. Then it was quenched by the addition of sat. NaHCO_3_ solution (35 mL) and Na_2_S_2_O_3_ solution (2 M, 35 mL). The water phase was washed twice with DCM (3 × 100 mL). The combined organic phases were washed with dionized H_2_O, dried over Na_2_SO_4_and filtered, and the solvent was evaporated in vacuo. The crude was purified by column chromatography on silica gel (5%–10% MeOH in EtOAc) to yield ketone (±)-**34** (0.89 g, 4.91 mmol, 82%) as a white solid. R_f_ = 0.20 (5% MeOH in EtOAc); ^1^H NMR (400 MHz, MeOD-*d*_4_, 298 K): δ [ppm] = 4.27–4.28 (m, 1H; H-*C*(5a)), 2.90–2.92 (m, 1H; H-*C*(9a)), 2.49–2.56 (m, 1H; H-*C* (3)), 2.42–2.48 (m, 1H; H-*C* (7)), 2.23–2.36 (m, 3H; H-*C* (3, 7 and 9)), 2.07–2.19 (m, 2H; H-*C* (5 and 8)), 1.98–2.06 (m, 2H; H-*C* (5 and 8)), 1.79–1.86 (m, 1H; H-*C* (4)), 1.68–1.77 (m, 1H; H-*C* (4)), 1.52–1.61 (m, 1H; H-*C* (9)); ^13^C NMR (100 MHz, MeOD-*d*_4_, 298 K): δ [ppm] = 212.8 (C (6)), 181.1 (C (2)), 55.8 (C(5a)), 52.3 (C(9a)), 41.6 (C (7)), 37.2 (C (3)), 30.9 (C (5 or 8)), 29.0 (C (9)), 22.4 (C (5 or 8)), 20.2 (C (4)); HR-MS (ESI): (*m*/*z*) = calculated for C_10_H_16_NO_2_^+^ [*M*+H]^+^: 182.1176, found: 182.1170, C_20_H_33_N_2_O_4_^+^ [2*M*+H]^+^: 363.2284, found: 363.2267.

#### Dodecahydroazepino[3,2-*b*]azepine (*meso*-**4b**)

Ketone (±)-**34** (471 mg, 2.60 mmol, 1.0 equiv) was dissolved in CHCl_3_ (26 mL, 0.1 M) and the solution was cooled to 0 °C. Then, conc. H_2_SO_4_ (1.11 mL, 20.8 mmol, 8.0 equiv) was added dropwise. Upon completion of the addition, NaN_3_ (372 mg, 5.72 mmol, 2.2 equiv) was added portionwise at 0 °C. *It is critical that the temperature during addition is maintained at 0 °C.* The reaction mixture was then warmed to 22 °C and stirred vigorously for 24 h. The viscous bottom layer (aqueous) was separated from the top layer, cooled to 0 °C, and carefully quenched by the dropwise addition of a sat. NaHCO_3_ solution to pH 12. *It is critical that the pH of the aqueous phase is >10 so that residual HN*_*3*_*(toxic, explosive) is deprotonated.* Upon completion of the addition, 25% *^i^*PrOH in CHCl_3_ (25 mL) was added, and the reaction mixture was stirred vigorously for 1 h. The filtrate was then separated, and the aqueous phase was extracted with 25% *^i^*PrOH in CHCl_3_ (3 × 25 mL). All organic layers were combined, dried over Na_2_SO_4_, and filtered, and the solvent was evaporated to yield a yellow solid. The intermediate product was dissolved in dry THF (26 mL, 0.1 M) and cooled to 0 °C. Then, LiAlH_4_ (1 M in THF, 23.4 mL, 23.4 mmol, 9.0 equiv) was added dropwise. The mixture was stirred at reflux for 24 h. Then, it was cooled down and worked up using Fieser’s protocol. The crude product was purified by flash column chromatography on silica gel (10%–15% MeOH in DCM + 1% NH_3_) to afford final free diamine *meso*-**4b** (186 mg, 1.11 mmol, 43%) as a white solid. ^1^H NMR (400 MHz, MeOD-*d*_4_, 298 K): δ [ppm] = 3.01–3.07 (m, 2H), 2.72–2.79 (m, 2H), 2.66–2.67 (m, 2H), 1.89–1.94 (m, 2H), 1.60–1.75 (m, 10H); ^13^C NMR (100 MHz, MeOD-*d*_4_, 298 K): δ [ppm] = 65.9 (*C*H), 49.6 (*C*H_2_), 36.2 (*C*H_2_), 29.7 (*C*H_2_), 24.4 (*C*H_2_); HR-MS (ESI): (*m*/*z*) = calculated for C_10_H_21_N_2_^+^ [*M*+H]^+^: 169.1699, found: 169.1696.

#### *tert*-Butyl-4-benzyloctahydropyrrolo[3,2-*b*]azepine-1(2*H*)-carboxylate ((±)-**35**)

To a solution of mono-Boc-protected diamine (±)-**11** (397 mg, 1.65 mmol, 1.0 equiv) in MeOH (17 mL, 0.1 M) was added benzyl bromide (235 μL, 1.98 mmol, 1.2 equiv) and K_2_CO_3_ (343 mg, 2.48 mmol, 1.5 equiv). The mixture was stirred at 22 °C for 2 h. Then, the solvent was evaporated, and the residue was diluted with dionized H_2_O (25 mL) and extracted with EtOAc (3 × 50 mL). The organic layer was dried over Na_2_SO_4_and filtered, and the solvent was removed in vacuo. The crude was purified by column chromatography on silica gel (10% EtOAc in heptane) to give orthogonally protected diamine (±)-**35** (487 mg, 1.47 mmol, 89%) as a yellow oil. R_f_ = 0.20 (10% EtOAc in heptane); ^1^H NMR (400 MHz MeOD-*d*_4_, 298 K): δ [ppm] = 7.35–7.37 (m, 2H; H_2_-*C*_arom._), 7.27–7.31 (m, 2H; H_2_-*C*_arom._), 7.19–7.23 (m, 1H; H-*C*_arom._), 3.96 (d, *J* = 13.7, 1H; H_2_-*C*_benz_), 3.77–3.83 (m, 1H; H-*C*(8a)), 3.42–3.49 (m, 1H; H-*C* (2)), 3.35 (d, *J* = 13.7, 1H; H_2_-*C*_benz._), 3.14–3.27 (m, 2H; H-*C*(2 and 3a)), 2.62–2.65 (m, 1H; H-*C* (5)), 2.34–2.40 (m, 1H; H-*C* (5)), 2.25–2.28 (m, 1H; H-*C* (3)), 1.89–2.07 (m, 2H; H-*C* (3 and 8)), 1.65–1.75 (m, 2H; H-*C* (7 and 8)), 1.50–1.52 (m, 1H; H-*C* (6)), 1.46 (s, 9H, H_3_-*C*_Boc_), 1.29–1.34 (m, 2H; H-*C* (6 and 7)); ^13^C NMR (100 MHz, MeOD-*d*_4_, 298 K): δ [ppm] = 156.2 ((*C*=O)_Boc-rot._, 156.1((*C*=O)_Boc-rot._, 141.2 ((C_q_)_arom._), 129.9 ((*C*H)_arom._), 129.2 ((*C*H)_arom._), 127.9 ((*C*H)_arom._), 80.8 ((C_q_)_Boc_)_rot._, 80.7 ((C_q_)_Boc_)_rot._, 68.0 (C(3a))_rot_, 67.4 (C(3a))_rot._, 64.2 (C(8a))_rot._, 63.8 (C(8a))_rot._, 61.3 (*C*H_2_)_benz._)), 53.5 (C (5)), 45.5 (C(2)_rot._, 44.9 (C(2)_rot._, 32.4 (C (6), 31.1 (C (3 or 8), 30.4 (C (3 or 8))_rot._, 30.3 (C (3 or 8))_rot._, 28.8 ((*C*H_3_)_Boc_), 28.1 (C (7))_rot._, 28.0 (C (7))_rot._; HR-MS (ESI): (*m*/*z*) = calculated for C_20_H_31_N_2_O_2_^+^ [M + H]^+^: 331.2380, found: 331.2374.

#### 4-Benzyldecahydropyrrolo[3,2-*b*]azepine ((±)-**1a**)

To a solution of orthogonally protected diamine (±)-**35** (50 mg, 0.15 mmol, 1.0 equiv) in DCM (1.5 mL, 0.1 M) was added TFA (0.15 mL, 10 vol %). The reaction was stirred at 22 °C for 2 h. Then, it was washed with NaOH (1 M, 2 × 4 mL); the organic phase was dried over Na_2_SO_4_and filtered, and the solvent was evaporated. The crude was purified by column chromatography on silica gel (10% MeOH in DCM + 0.1% NH_3_) to yield monobenzylated diamine (±)-**1a** (22.0 mg, 0.96 mmol, 64%) as a yellow oil. R_f_ = 0.20 (10% MeOH in DCM + 0.1% NH_3_); ^1^H NMR (400 MHz, MeOD-*d*_4_, 298 K): δ [ppm] = 7.35–7.37 (m, 2H; H_2_-*C*_arom._), 7.27–7.30 (m, 2H; H_2_-*C*_arom._), 7.19–7.22 (m, 1H; H-*C*_arom._), 3.92 (d, *J* = 13.7, 1H; H_2_-*C*_benz._), 3.39 (d, *J* = 13.7, 1H; H_2_-*C*_benz._), 3.21–3.26 (m, 1H; H-*C*(3a)), 3.18–3.21 (m, 1H; H-*C*(8a)), 3.06–3.11 (m, 1H; H-*C* (2)), 2.77–2.82 (m, 1H; H-*C* (2)), 2.70–2.75 (m, 1H; H-*C* (5)), 2.26–2.33 (m, 1H; H-*C* (5)), 2.13–2.20 (m, 1H; H-*C* (3)), 2.00–2.09 (m, 1H; H-*C* (8)), 1.74–1.83 (m, 2H; H-*C* (3 and 7)), 1.58–1.63 (m, 1H; H-*C* (8)), 1.42–1.54 (m, 2H; H_2_-*C* (6)), 1.29–1.40 (m, 1H; H-*C* (7)); ^13^C NMR (100 MHz, MeOD-*d*_4_, 298 K): δ [ppm] = 141.4 ((C_q_)_arom._), 129.9 ((*C*H)_arom._), 129.2 ((*C*H)_arom._), 127.9 ((*C*H)_arom._), 69.7 (C(3a)), 64.5 (C(8a)), 61.4 (*C*H_2_)_benz._), 54.7 (C (5)), 45.2 (C (2), 33.2 (C (3), 32.3 (C (8), 31.9 (C (6), 27.7 (C (7)); HR-MS (ESI): (*m*/*z*) = calculated for C_15_H_23_N_2_^+^ [*M*+H]^+^: 231.1856, found: 231.1847. Enantiomeric separation of (±)-**35** was performed by Reach Separation on a preparative chiral-HPLC (conditions: Chiralcel OD-H (30 mm × 250 mm, 5 μm), ambient, 42 mL/min, MeOH).

#### *tert*-Butyl-(3a*R*,8a*R*)-4-benzyloctahydropyrrolo[3,2-*b*]azepine-1(2H)-carboxylate ((*R,R*)*-***35**)

More active enantiomer, see (±)-**35** for full assignment. Chiral-HPLC: t_R_ = 3.691 min, conditions: Chiralcel OD-H (30 mm × 250 mm, 5 μm), ambient, 42 mL/min, MeOH).

#### *tert*-Butyl-(3a*S*,8a*S*)-4-benzyloctahydropyrrolo[3,2-*b*]azepine-1(2H)-carboxylate ((*S,S*)*-***35**)

Less active enantiomer, see (±)-**35** for full assignment. Chiral-HPLC: t_R_ = 5.019 min, conditions: Chiralcel OD-H (30 mm × 250 mm, 5 μm), ambient, 42 mL/min, MeOH).

#### (3a*R*,8a*R*)-4-Benzyldecahydropyrrolo[3,2-*b*]azepine ((*R,R*)*-***1a**)

Orthogonally protected diamine (*R,R*)-**35** (102 mg, 0.31 mmol, 1.0 equiv) was dissolved in DCM (3.1 mL, 0.1 M), and TFA (0.3 mL, 10 vol %) was added. The reaction was stirred at 22 °C for 2 h. Then it was washed with NaOH (1 M, 2 × 5 mL) and the organic phase was dried over Na_2_SO_4_and filtered, and the solvent was evaporated. The crude was purified by column chromatography on silica gel (10% MeOH in DCM + 1% NH_3_) to yield monobenzylated diamine (*R,R*)-**1a** (49 mg, 0.21 mmol, 68%) as a yellow oil. Diffusion-controlled crystallization of an analytical sample from HCl in MeOH and Et_2_O yielded the title compound as HCl salt and allowed to determine the (*R,R*) stereochemistry at the bridgehead by X-ray diffraction studies. See racemate (±)-**1a** for the full assignment.

#### (3a*S*,8a*S*)-4-Benzyldecahydropyrrolo[3,2-*b*]azepine ((*S,S*)*-***1a**)

Orthogonally protected diamine (*S,S*)-**35** (105 mg, 0.32 mmol, 1.0 equiv) was dissolved in DCM (3.2 mL, 0.1 M) and TFA (0.3 mL, 10 vol %) was added. The reaction was stirred at 22 °C for 2 h. Then it was washed with NaOH (1 M, 2 × 5 mL) and the organic phase was dried over Na_2_SO_4_, filtered and the solvent was evaporated. The crude was purified by column chromatography on silica gel (10% MeOH in DCM + 1% NH_3_) to yield monobenzylated diamine (*S,S*)-**1a** (54 mg, 0.23 mmol, 72%) as a yellow oil. See racemate (±)-**1a** for full assignment.

#### Trifluoro-1-octahydropyrrolo[3,2-*b*]azepin-4(1*H*)-yl)ethanone ((±)-**36**)

To a solution of mono-Boc-protected diamine (±)-**11** (115 mg, 0.48 mmol, 1.0 equiv) in DCM (5 mL, 0.1 M) was added TFAA (100 μL, 0.72 mmol, 1.5 equiv) and pyridine (46 μL, 0.58 mmol, 1.2 equiv). The reaction mixture was stirred at 22 °C for 2 h. The organic phase was washed with dionized H_2_O (5 mL) and NaHCO_3_ (5 mL), dried over Na_2_SO_4_, and filtered, and the solvent was removed in vacuo to give a light-yellow oil. The intermediate product was dissolved in DCM (5 mL, 0.1 M), and TFA (0.5 mL, 10 vol %) was added. The reaction was stirred at 22 °C for 2 h. Then, it was washed with NaOH (1 M, 2 × 5 mL); the organic phase was dried over Na_2_SO_4_and filtered, and the solvent was evaporated. The crude was purified by column chromatography on silica gel (5% MeOH in DCM + 0.1% NH_3_) to give monotrifluoroacetamide-protected diamine (±)-**36** (55.0 mg, 0.23 mmol, 48%) as a yellow oil. R_f_ = 0.18 (5% MeOH in DCM + 0.1% NH_3_); ^1^H NMR (400 MHz, MeOD-*d*_4_, 298 K): δ [ppm] = 4.70–4.74 (m, 1H), 4.54–4.56 (m, 1.3H), 3.94 (d, *J* = 13.5, 1.3H), 3.74–3.78 (m, 1.2H), 3.48–3.61 (m, 3H), 3.39–3.43 (m, 1.8H), 3.23–3.27 (m, 1.3H), 3.00–3.11 (m, 2.3H), 2.69–2.76 (m, 1.4H), 2.37–2.44 (m, 1.2H), 2.27–2.35 (m, 1.5H), 2.12–2.21 (m, 2.5H), 1.97–2.04 (m, 1.4H), 1.70–1.93 (m, 8H), 1.51–1.67 (m, 4H); ^13^C NMR (100 MHz, MeOD-*d*_4_, 298 K): δ [ppm] = 157.5 (*C*OCF_3_), 118.1 (q, CO*C*F_3_), 63.5 (*C*H)_rot._, 63.0 (*C*H)_rot._, 62.3 (*C*H)_rot._, 60.4 (*C*H)_rot._, 49.3 (*C*H_2_)_rot._, 46.5 (*C*H_2_)_rot._, 45.9 (*C*H_2_)_rot._, 44.6 (*C*H_2_)_rot._, 32.8 (*C*H_2_)_rot._, 32.4 (*C*H_2_)_rot._, 30.0 (*C*H_2_)_rot._, 29.3 (*C*H_2_)_rot._, 28.9 (*C*H_2_)_rot._, 28.6 (*C*H_2_)_rot._, 23.2 (*C*H_2_)_rot._, 22.6 (*C*H_2_)_rot._; due to the presence of trifluoroacetamide rotamers in the NMR measurement, structural assignment is not possible; HR-MS (ESI): (*m*/*z*) = calculated for C_10_H_16_N_2_OF_3_^+^ [*M*+H]^+^: 237.1209, found: 237.1206.

#### 1-Benzyldecahydropyrrolo[3,2-*b*]azepine ((±)-**17a**)

To a solution of monotrifluoroacetamide-protected diamine (±)-**36** (55.0 mg, 0.23 mmol, 1.0 equiv) in MeOH (2.3 mL, 0.1 M) was added benzyl bromide (33 μL, 0.28 mmol, 1.2 equiv) and K_2_CO_3_ (48.4 mg, 0.35 mmol, 1.5 equiv). The mixture was stirred at 22 °C for 2 h. Then, the solvent was evaporated, and the residue was diluted with dionized H_2_O and extracted with EtOAc (3 × 10 mL). The organic layers were dried over Na_2_SO_4_and filtered, and the solvent was removed in vacuo to give a yellow oil. The intermediate product was dissolved in a mixture of THF and dionized H_2_O (3 mL, 1:1), and LiOH (27.5 mg, 1.15 mmol, 5.0 equiv) was added. The mixture was refluxed for 24 h. Then, it was cooled to room temperature and concentrated under reduced pressure. The aqueous phase was extracted with EtOAc (3 × 6 mL). The organic layers were dried over Na_2_SO_4_and filtered, and the solvent was removed in vacuo. The crude was purified by column chromatography on silica gel (10% MeOH in DCM + 0.1% NH_3_) to give monobenzylated diamine (±)-**17a** (21.0 mg, 0.09 mmol, 39%) as a yellow oil. R_f_ = 0.20 (10% MeOH in DCM + 0.1% NH_3_); ^1^H NMR (400 MHz, MeOD-*d*_4_, 298 K): δ [ppm] = 7.24–7.31 (m, 5H; H-*C*_arom._), 3.97 (d, *J* = 12.6, 1H; H-*C*_benz._), 3.45–3.46 (m, 1H; H-*C*(3a)) 3.12–3.19 (m, 2H; H-*C*_benz._ and *C* (5)), 2.81–2.85 (m, 1H; H-*C* (2)), 2.45–2.52 (m, 2H; H-*C*(5 and 8a)), 2.06–2.17 (m, 2H; H-*C* (2 and 3)), 1.97–2.02 (m, 1H; H-*C* (6)), 1.88–1.91 (m, 1H; H-*C* (7)), 1.77–1.81 (m, 1H; H-*C* (8)), 1.54–1.64 (m, 2H; H-*C* (6 and 8)), 1.40–1.46 (m, 1H; H-*C* (3)), 1.29–1.37 (m, 1H; H-*C* (7)); ^13^C NMR (100 MHz, MeOD-*d*_4_, 298 K): δ [ppm] = 139.5 ((C_q_)_arom._), 130.5 ((*C*H)_arom._), 129.3 ((*C*H)_arom._), 128.3 ((*C*H)_arom._), 70.9 (C(8a)), 63.8 (C(3a)), 59.9 (*C*H_2_)_benz._), 53.5 (C (2)), 50.9 (C (5), 33.6 (C (8), 32.6 (C (3), 30.4 (C (6), 25.7 (C (7)); HR-MS (ESI): (*m*/*z*) = calculated for C_15_H_23_N_2_^+^ [*M*+H]^+^: 231.1856, found: 231.1851.

#### *tert*-Butyl-5-oxooctahydropyrrolo[3,2-*b*]azepine-1(2*H*)-carboxylate ((±)-**37**)

To a solution of amine (±)-**15** (181 mg, 1.17 mmol, 1.0 equiv) in DCM (12 mL, 0.1 M) was added Boc_2_O (308 mg, 1.41 mmol, 1.2 equiv), NEt_3_ (213 μL, 1.53 mmol, 1.3 equiv) and DMAP (cat.). The reaction mixture was stirred at 22 °C for 2 h. Then the solvent was evaporated, and the crude was purified by column chromatography on silica gel (10% MeOH in DCM + 1% NH_3_) to give Boc-protected amine (±)-**37** (191 mg, 0.75 mmol, 64%) as a white solid. R_f_ = 0.20 (10% MeOH in DCM + 1% NH_3_); ^1^H NMR (400 MHz, MeOD-*d*_4_, 298 K): δ [ppm] = 3.81–3.88 (m, 1H; H-*C*(3a)), 3.69–3.74 (m, 1H; H-*C* (2)), 3.22–3.28 (m, 1H; H-*C* (2)) 3.07–3.13 (m, 1H; H-*C*(8a)), 2.79 (br, 1H; H-*C* (8)), 2.68–2.75 (m, 1H; H-*C* (6)), 2.36 (dd, *J* = 14.2, 6.9 Hz, 1H; H-*C* (6)), 2.05–2.11 (m, 1H; H-*C* (3)), 1.95–1.99 (m, 1H; H-*C* (7)), 1.76–1.87 (m, 1H; H-*C* (3)), 1.56–1.66 (m, 1H; H-*C* (7)), 1.46 (s, 9H; H_3_-*C*_Boc_), 1.37–1.40 (m, 1H; H-*C* (8)); ^13^C NMR (100 MHz, MeOD-*d*_4_, 298 K): δ [ppm] = 180.4 (C (5)), 156.6 ((*C*=O)_Boc_, 81.2 ((C_q_)_Boc_), 65.0 (C(8a)), 59.5 (C(3a)), 46.6 (C (2)), 37.6 (C (6)), 35.8 (C (8)), 29.8 (C (3)), 28.7 ((*C*H_3_)_Boc_), 22.7 (C (7)); HR-MS (ESI): (*m*/*z*) = calculated for C_13_H_23_N_2_O_3_^+^ [*M*+H]^+^: 255.1703, found: 255.1679.

#### *tert*-Butyl-octahydropyrrolo[3,2-*b*]azepine-1(2*H*)-carboxylate ((±)-**38**)

A solution of lactam (±)-**37** (141 mg, 0.55 mmol, 1.0 equiv) in dry THF (5.5 mL, 0.1 M) was cooled to 0 °C, and LiAlH_4_ (1 M in THF, 0.83 mL, 0.83 mmol, 1.5 equiv) was added dropwise. The reaction was stirred at 22 °C for 3 h. Then, it was worked up using Fieser's protocol, and the crude was purified by flash column chromatography on silica gel (10% MeOH in DCM + 0.1% NEt_3_) to give azepane (±)-**38** (60.0 mg, 0.25 mmol, 45%) as a white solid. R_f_ = 0.20 (10% MeOH in DCM + 0.1% NEt_3_); ^1^H NMR (400 MHz, MeOD-*d*_4_, 298 K): δ [ppm] = 3.67 (dd, *J* = 11.1, 8.2 Hz, 1H; H-*C* (2)), 3.39–3.45 (m, 1H; H-*C*(8a)), 3.14–3.25 (m, 2H; H-*C*(2 and 3a)) 2.95–3.01 (m, 1H; H-*C* (5)), 2.84–2.90 (m, 1H; H-*C* (5)), 2.55 (br, 1H; H-*C* (8)), 1.99 (quint, *J* = 5.8 Hz, 1H; H-*C* (3)), 1.54–1.83 (m, 5H; H-*C* (3, 6 and 7)), 1.45 (s, 9H; H_3_-*C*_Boc_), 1.26–1.29 (m, 1H; H-*C* (8)); ^13^C NMR (100 MHz, MeOD-*d*_4_, 298 K): δ [ppm] = 156.4 ((*C*=O)_Boc_, 80.9 ((C_q_)_Boc_), 64.5 (C(3a or 8a)), 64.0 (C(3a or 8a)), 50.0 (C (5)), 46.3 (C (2)), 33.6 (C (8)), 32.6 (C (3)), 28.8 ((*C*H_3_)_Boc_), 28.5 (C (6)), 26.2 (C (7)); HR-MS (ESI): (*m*/*z*) = calculated for C_13_H_25_N_2_O_2_^+^ [*M*+H]^+^: 241.1911, found: 241.1905.

#### 4-Benzyldecahydropyrrolo[3,2-*b*]azepine ((±)-**1b**)

To a solution of mono-Boc-protected diamine (±)-**38** (49.2 mg, 0.20 mmol, 1.0 equiv) in MeOH (2 mL, 0.1 M) was added benzyl bromide (29 μL, 0.24 mmol, 1.2 equiv) and K_2_CO_3_ (41.5 mg, 0.30 mmol, 1.5 equiv). The mixture was stirred at 22 °C for 2 h. Then, the solvent was evaporated, and the residue was diluted with deionized H_2_O and extracted with EtOAc (3 × 10 mL). The organic layers were dried over Na_2_SO_4_ and filtered, and the solvent was removed in vacuo to give a white solid. The intermediate product was dissolved in DCM (2 mL, 0.1 M), and TFA (0.2 mL, 10 vol %) was added. The reaction was stirred at 22 °C for 2 h. Then, it was washed with NaOH (1 M, 2 × 4 mL); the organic phase was dried over Na_2_SO_4_ and filtered, and the solvent was evaporated. The crude was purified by column chromatography on silica gel (10% MeOH in DCM + 0.1% NH_3_) to give monobenzylated diamine (±)-**1b** (33.3 mg, 0.14 mmol, 70%) as a yellow oil. R_f_ = 0.20 (10% MeOH in DCM + 0.1% NH_3_); ^1^H NMR (400 MHz, MeOD-*d*_4_, 298 K): δ [ppm] = 7.25–7.33 (m, 5H; H-*C*_arom._, 3.83 (d, *J* = 13.4, 1H; H-*C*_benz._), 3.46 (d, *J* = 13.4, 1H; H-*C*_benz._) 2.90–3.04 (m, 3H; H-*C*(2 and 8a)), 2.67–2.83 (m, 3H; H-*C*(3a and 5)), 2.25–2.34 (m, 1H; H-*C* (3)), 2.02–2.06 (m, 1H; H-*C* (8)), 1.81–1.88 (m, 1H; H-*C* (3)), 1.75–1.80 (m, 1H; H-*C* (6)), 1.62–1.71 (m, 1H; H-*C* (7)), 1.39–1.55 (m, 3H; H-*C* (6, 7 and 8)); ^13^C NMR (100 MHz, MeOD-*d*_4_, 298 K): δ [ppm] = 139.8 ((C_q_)_arom._), 130.6 ((*C*H)_arom._), 129.3 ((*C*H)_arom._), 128.3 ((*C*H)_arom._), 72.0 (C(3a)), 62.4 (C(8a)), 60.6 (*C*H_2_)_benz._), 53.6 (C (5)), 45.0 (C (2), 35.1 (C (3), 34.0 (C (8), 27.2 (C (6), 23.8 (C (7)); HR-MS (ESI): (*m*/*z*) = calculated for C_15_H_23_N_2_^+^ [*M*+H]^+^: 231.1856, found: 231.1847.

#### *tert*-Butyl-decahydro-1*H*-pyrido[3,2-*b*]azepine-1-carboxylate ((±)-**39**)

To a solution of monotrifluoroacetamide-protected diamine (±)-**24** (102 mg, 0.41 mmol, 1.0 equiv) in DCM (4 mL, 0.1 M) was added Boc_2_O (107 mg, 0.49 mmol, 1.2 equiv), NEt_3_ (74 μL, 0.53 mmol, 1.3 equiv), and DMAP (cat.). The reaction mixture was stirred at 22 °C for 2 h. Then, the solvent was evaporated to give a white solid. The intermediate product was dissolved in a mixture of THF and dionized H_2_O (4 mL, 1:1), and LiOH (49.1 mg, 2.05 mmol, 5.0 equiv) was added. The mixture was refluxed for 24 h. Then, it was cooled to room temperature and concentrated under reduced pressure. The aqueous phase was extracted with EtOAc (3 × 15 mL). The organic layers were dried over Na_2_SO_4_ and filtered, and the solvent was removed in vacuo. The crude was purified by column chromatography on silica gel (10% MeOH in DCM + 0.1% NEt_3_) to give mono-Boc-protected diamine (±)-**39** (79.3 mg, 0.31 mmol, 76%) as a yellow oil. R_f_ = 0.20 (10% MeOH in DCM + 0.1% NEt_3_); ^1^H NMR (400 MHz, MeOD-*d*_4_, 298 K): δ [ppm] = 4.33–4.35 (t, *J* = 8.0 Hz, 1H), 3.88–3.92 (m, 1H), 2.91–2.99 (m, 2H) 2.63–2.72 (m, 2H), 1.96–2.05 (m, 1H), 1.84–1.86 (m, 2H), 1.74–1.78 (m, 1H), 1.66–1.69 (m, 1H), 1.46 (s, 9H; H_3_-*C*_Boc_), 1.36–1.44 (m, 5H); ^13^C NMR (100 MHz, MeOD-*d*_4_, 298 K): δ [ppm] = 156.7 ((*C*=O)_Boc_, 81.2 ((C_q_)_Boc_), 57.0 (*C*H), 55.6 (*C*H), 44.4 (*C*H_2_), 40.8 (*C*H_2_), 32.6 (*C*H_2_), 29.0 (*C*H_2_), 28.7 ((*C*H_3_)_Boc_), 28.5 (*C*H_2_), 28.3 (*C*H_2_), 25.5 (*C*H_2_); HR-MS (ESI): (*m*/*z*) = calculated for C_14_H_27_N_2_O_2_^+^ [*M*+H]^+^: 255.2067, found: 255.2046.

#### 5-Benzyldecahydro-1*H*-pyrido[3,2-*b*]azepine ((±)-**40a**)

To a solution of mono-Boc-protected diamine (±)-**39** (74.4 mg, 0.29 mmol, 1.0 equiv) in MeOH (3 mL, 0.1 M) was added benzyl bromide (42 μL, 0.35 mmol, 1.2 equiv) and K_2_CO_3_ (48.4 mg, 0.35 mmol, 1.2 equiv). The mixture was stirred at 22 °C for 2 h. Then, the solvent was evaporated, and the residue was diluted with deionized H_2_O and extracted with EtOAc (3 × 10 mL). The organic layers were dried over Na_2_SO_4_ and filtered, and the solvent was removed in vacuo to give a yellow oil. The intermediate product was dissolved in DCM (3 mL, 0.1 M), and TFA (0.3 mL, 10 vol %) was added. The reaction was stirred at 22 °C for 2 h. Then, it was washed with NaOH (1 M, 2 × 4 mL); the organic phase was dried over Na_2_SO_4_ and filtered, and the solvent was evaporated. The crude was purified by column chromatography on silica gel (10% MeOH in DCM + 0.1% NEt_3_) to give monobenzylated diamine (±)-**40a** (56.8 mg, 0.23 mmol, 79%) as a brown oil. R_f_ = 0.20 (DCM/MeOH 9:1 + 0.1% NEt_3_); ^1^H NMR (400 MHz, MeOD-*d*_4_, 298 K): δ [ppm] = 7.33–7.35 (m, 2H; H_2_-*C*_arom._), 7.26–7.29 (m, 2H; H_2_-*C*_arom._), 7.18–7.21 (m, 1H; H-*C*_arom_), 3.89 (d, *J* = 14.1, 1H; H-*C*_benz._), 3.56 (d, *J* = 14.1, 1H; H-*C*_benz._) 3.12–3.16 (m, 1H; H-*C*(9a)), 2.74–2.80 (m, 1H; H-*C*(4a)), 2.59–2.73 (m, 4H; H_2_-*C* (2 and 6)), 2.08–2.18 (m, 1H; H-*C* (9)), 1.85–1.89 (m, 1H; H-*C* (4)), 1.68–1.80 (m, 4H; H-*C* (3, 4, 7, 8 or 9)), 1.55–1.60 (m, 1H; H-*C* (8)), 1.39–1.54 (m, 3H; H-*C* (3, 4, 7, 8 or 9)); ^13^C NMR (100 MHz, MeOD-*d*_4_, 298 K): δ [ppm] = 141.9 ((C_q_)_arom._), 129.7 ((*C*H)_arom._), 129.2 ((*C*H)_arom._), 127.8 ((*C*H)_arom._), 62.9 (C(4a)), 60.2 (*C*H_2_)_benz._), 58.7 (C(9a)), 49.2 (C (2 or 6)), 41.3 (C (2 or 6)), 30.6 (C (3, 4, 7, 8 or 9), 30.4 (C (3, 4, 7, 8 or 9), 26.7 (C (3, 4, 7, 8 or 9), 26.4 (C (3, 4, 7, 8 or 9), 26.2 (C (3, 4, 7, 8 or 9)); HR-MS (ESI): (*m*/*z*) = calculated for C_16_H_25_N_2_^+^ [*M*+H]^+^: 245.2012, found: 245.1990.

#### 1-Benzyldecahydro-1*H*-pyrido[3,2-*b*]azepine ((±)-**27a**)

To a solution of monotrifluoroacetamide-protected diamine (±)-**24** (50.0 mg, 0.20 mmol, 1.0 equiv) in MeOH (2 mL, 0.1 M) was added benzyl bromide (29 μL, 0.24 mmol, 1.2 equiv) and K_2_CO_3_ (41.5 mg, 0.30 mmol, 1.5 equiv). The mixture was stirred at 22 °C for 2 h. Then, the solvent was evaporated, and the residue was diluted with deionized H_2_O and extracted with EtOAc (3 × 10 mL). The organic layers were dried over Na_2_SO_4_ and filtered, and the solvent was removed in vacuo to give a yellow oil. The intermediate product was dissolved in a mixture of THF and dionized H_2_O (4 mL, 1:1), and LiOH (24.0 mg, 1.0 mmol, 5.0 equiv) was added. The mixture was refluxed for 24 h. Then, it was cooled to room temperature and concentrated under reduced pressure. The aqueous phase was extracted with EtOAc (3 × 10 mL). The organic layers were dried over Na_2_SO_4_ and filtered, and the solvent was removed in vacuo. The crude was purified by column chromatography on silica gel (10% MeOH in DCM + 1% NH_3_) to give monobenzylated diamine (±)-**27a** (22.2 mg, 0.09 mmol, 45%) as a yellow oil. R_f_ = 0.20 (10% MeOH in DCM + 1% NH_3_); ^1^H NMR (400 MHz, MeOD-*d*_4_, 298 K): δ [ppm] = 7.20–7.36 (m, 5H; H-*C*_arom._), 3.82 (d, *J* = 13.3, 1H; H-*C*_benz._), 3.38 (d, *J* = 13.3, 1H; H-*C*_benz._), 2.97 (quint. *J* = 4.1 Hz, 1H; H-*C*(4a)), 2.90 (dt, *J* = 14.2, 4.3 Hz, 1H; H-*C* (6)), 2.78–2.80 (m, 1H; H-*C*(9a)), 2.52–2.59 (m, 1H; H-*C* (6)), 2.43–2.49 (m, 1H; H-*C* (2)), 2.20–2.25 (m, 1H; H-*C* (2)), 1.40–1.86 (m, 9H; H-*C* (3, 4, 7, 8 and 9)), 1.22–1.29 (m, 1H; H-*C* (3, 4, 7, 8 or 9)); ^13^C NMR (100 MHz, MeOD-*d*_4_, 298 K): δ [ppm] = 140.4 ((C_q_)_arom._), 130.1 ((*C*H)_arom._), 129.2 ((*C*H)_arom._), 128.0 ((*C*H)_arom._), 64.3 (C(9a)), 59.6 (*C*H_2_)_benz._), 57.2 (C(4a)), 49.9 (C (2)), 45.8 (C (6), 33.0 (C (7, 8 or 9), 30.4 (C (3 or 4)), 25.8 (C (7, 8 or 9)), 24.5 (C (3, 4, 7, 8 or 9)), 24.3 (C (3, 4, 7, 8 or 9)); HR-MS (ESI): (*m*/*z*) = calculated for C_16_H_25_N_2_^+^ [*M*+H]^+^: 245.2012, found: 245.2006.

#### *tert*-Butyl-6-oxodecahydro-1*H*-pyrido[3,2-*b*]azepine-1-carboxylate ((±)-**41**)

To a solution of amine (±)-**25** (50.0 mg, 0.30 mmol, 1.0 equiv) in DCM (3 mL, 0.1 M) was added Boc_2_O (79 mg, 0.36 mmol, 1.2 equiv) and NEt_3_ (54 μL, 0.39 mmol, 1.3 equiv). The reaction mixture was stirred at 22 °C for 2 h. Then, the solvent was evaporated, and the crude was purified by column chromatography on silica gel (80% EtOAc in heptane) to give Boc-protected amine (±)-**41** (65 mg, 0.24 mmol, 80%) as a white solid. R_f_ = 0.21 (80% EtOAc in heptane); ^1^H NMR (400 MHz, MeOD-*d*_4_, 298 K): δ [ppm] = 4.64–4.67 (m, 1H; H-*C* (2)), 3.79 (m, 1H; H-*C*(9a)), 3.49–3.54 (m, 1H; H-*C*(4a)), 2.48–2.54 (m, 1H; H-*C* (2)), 2.28–2.31 (m, 2H; H-*C* (7)), 1.57–1.89 (m, 8H; H-*C* (3, 4, 8 and 9)), 1.46 (m, 9H; H_3_-*C*_Boc_); ^13^C NMR (100 MHz, MeOD-*d*_4_, 298 K): δ [ppm] = 173.3 (C (6)), 158.3 ((*C*=O)_Boc_, 80.4 ((C_q_)_Boc_), 60.3 (C(4a)), 50.4 (C(9a)), 43.3 (C (2)), 33.6 (C (7)), 31.1 (C (3, 4, 8 or 9)), 28.8 (C (3, 4, 8 or 9)), 27.2 (C (3, 4, 8 or 9)), 20.6 (C (3, 4, 8 or 9)), 19.9 ((*C*H_3_)_Boc_); HR-MS (ESI): (*m*/*z*) = calculated for C_14_H_25_N_2_O_3_^+^ [*M*+H]^+^: 269.1860, found: 269.1958.

#### *tert*-Butyl-decahydro-1*H*-pyrido[3,2-*b*]azepine-1-carboxylate ((±)-**42**)

A solution of lactam (±)-**41** (199 mg, 0.74 mmol, 1.0 equiv) in dry THF (7.5 mL, 0.1 M) was cooled to 0 °C, LiAlH_4_ (1 M in THF, 1.11 mL, 1.11 mmol, 1.5 equiv) was added dropwise and the reaction was stirred at 0 °C for 4 h. The reaction was then worked up using Fieser's protocol, and the crude was purified by flash column chromatography on silica gel (EtOAc) to give azepane (±)-**42** (85 mg, 0.33 mmol, 45%) as a colorless oil. R_f_ = 0.20 (EtOAc); ^1^H NMR (400 MHz, MeOD-*d*_4_, 298 K): δ [ppm] = 3.59 (s, 1H; H-*C*(9a)), 2.76–2.83 (m, 2H; H_2_-*C* (2 or 6)), 1.98–2.08 (m, 3H; H-*C*(4a and H_2_-*C* (2 or 6)), 1.69–1.78 (m, 3H; H-*C* (9 and 3, 4, 7 or 8)), 1.62–1.68 (m, 1H; H-*C* (3, 4, 7 or 8)), 1.48–1.58 (m, 4H; H-*C* (9 and 3, 4, 7 or 8)), 1.44 (m, 9H; H_3_-*C*_Boc_), 1.29–1.43 (m, 2H; H-*C* (3, 4, 7 or 8)); ^13^C NMR (100 MHz, MeOD-*d*_4_, 298 K): δ [ppm] = 157.9 ((*C*=O)_Boc_), 80.2 ((C_q_)_Boc_), 66.0 (C(4a)), 57.8 (C (2 or 6)), 57.4 (C (2 or 6)), 50.6 (C(9a)), 31.1 (C (3, 4, 7 or 8)), 29.9 (C (3, 4, 7 or 8)), 28.7 ((*C*H_3_)_Boc_), 26.5 (C (3, 4, 7 or 8)), 25.1 (C (3, 4, 7 or 8)), 21.2 (C (9)); HR-MS (ESI): (*m*/*z*) = calculated for C_14_H_27_N_2_O_2_^+^ [*M*+H]^+^: 255.2067, found: 255.2064.

#### 5-Benzyldecahydro-1*H*-pyrido[3,2-*b*]azepine ((±)-**40b**)

To a solution of mono-Boc-protected diamine (±)-**42** (85 mg, 0.33 mmol, 1.0 equiv) in MeOH (3.5 mL, 0.1 M) was added benzyl bromide (48 μL, 0.40 mmol, 1.2 equiv) and K_2_CO_3_ (55 mg, 0.40 mmol, 1.2 equiv). The mixture was stirred at 22 °C for 2 h. Then, the solvent was evaporated, and the residue was diluted with dionized H_2_O and extracted with EtOAc (3 × 10 mL). The organic layers were dried over Na_2_SO_4_ and filtered, and the solvent was removed in vacuo to give a yellow oil. The intermediate product was dissolved in DCM (3.5 mL, 0.1 M), and TFA (0.35 mL, 10 vol %) was added. The reaction was stirred at 22 °C for 2 h. Then, it was washed with NaOH (1 M, 2 × 4 mL); the organic phase was dried over Na_2_SO_4_ and filtered, and the solvent was evaporated. The crude was purified by column chromatography on silica gel (10% MeOH in DCM + 0.1% NEt_3_) to give monobenzylated diamine (±)-**40b** (86 mg, 0.25 mmol, 76%) as a brown oil. R_f_ = 0.20 (10% MeOH in DCM + 0.1% NEt_3_); ^1^H NMR (400 MHz, MeOD-*d*_4_, 298 K): δ [ppm] = 7.67–7.68 (m, 2H; H_2_-*C*_arom._), 7.53–7.54 (m, 3H; H-*C*_arom._), 4.68–4.71 (m, 1H; H-*C*_benz._), 4.53–4.56 (m, 1H; H-*C*_benz._), 4.16–4.20 (m, 1H; H-*C*(4a or 9a)), 3.93–3.96 (m, 1H; H-*C*(4a or 9a)), 3.60–3.65 (m, 1H; H-*C* (2, 3, 4, 6, 7, 8, or 9)), 3.46–3.52 (m, 1H; H_2_-*C* (2, 3, 4, 6, 7, 8, or 9)), 3.19–3.22 (m, 1H; H-*C* (2, 3, 4, 6, 7, 8, or 9)), 3.01–3.08 (m, 1H; H-*C* (2, 3, 4, 6, 7, 8, or 9)), 1.92–3.32 (m, 8H; H-*C* (2, 3, 4, 6, 7, 8, or 9)), 1.65–1.82 (m, 2H; H-*C* (2, 3, 4, 6, 7, 8, or 9)); ^13^C NMR (100 MHz, MeOD-*d*_4_, 298 K): δ [ppm] = 133.2 ((*C*H)_arom._), 131.7 ((C_q_)_arom._), 130.6 ((*C*H)_arom._), 130.0 130.6 ((*C*H)_arom._), 2X 62.3 (*C*H_2_)_benz._) and (C(4a)) or (C(9a)), 54.5 (C(4a)) or (C(9a)), 53.4 (C (2, 3, 4, 6, 7, 8, or 9), 39.4 (C (2, 3, 4, 6, 7, 8, or 9)), 2X 26.6 (C (2, 3, 4, 6, 7, 8, or 9)), 25.7 (C (2, 3, 4, 6, 7, 8, or 9), 23.3 (C (2, 3, 4, 6, 7, 8, or 9), 22.3 (C (2, 3, 4, 6, 7, 8, or 9); HR-MS (ESI): (*m*/*z*) = calculated for C_16_H_25_N_2_^+^ [*M*+H]^+^: 245.2012, found: 245.2010.

#### *tert*-Butyl-decahydroazepino[3,2-*b*]azepine-1(2*H*)-carboxylate ((±)-**43**)

To a solution of free diamine *meso*-**4b** (75.0 mg, 0.62 mmol, 1.0 equiv) in DCM (6 mL, 0.1 M) was added Boc_2_O (406 mg, 1.86 mmol, 3.0 equiv), NEt_3_ (259 μL, 1.86 mmol, 3.0 equiv), and DMAP (cat.). The reaction mixture was stirred at 22 °C for 24 h. Then, the solvent was evaporated, and the crude product was purified by column chromatography on silica gel (heptane/EtOAc 8:2) to give bis-Boc-protected diamine (65.4 mg, 0.18 mmol, 29%). *Due to the presence of Boc rotamers in the NMR measurement, no proper data could be obtained.* The intermediate product was dissolved in CHCl_3_ (7.3 mL, 0.024 mol/L), and TFA (0.73 mL, 10 vol %) was added at 0 °C. The reaction was stirred at this temperature for 90 min. Then, it was quenched by the addition of NaOH (1 M, 5.7 mL) and extracted with DCM (3 × 6 mL). The organic layers were dried over Na_2_SO_4_ and filtered, and the solvent was removed in vacuo. The crude was purified by column chromatography on silica gel (5% MeOH in DCM + 1% NH_3_) to give mono-Boc-protected diamine (±)-**43** (16.7 mg, 0.06 mmol, 10%) as a yellow oil. R_f_ = 0.20 (5% MeOH in DCM + 1% NH_3_); ^1^H NMR (400 MHz, MeOD-*d*_4_, 298 K): δ [ppm] = 3.82–3.95 (m, 1H), 3.55–3.67 (m, 2H), 2.80–3.01 (m, 4H) 1.62–1.84 (m, 11H), 1.47 (m, 9H); ^13^C NMR (100 MHz, MeOD-*d*_4_, 298 K): δ [ppm] = 157.0, 156.7, 81.2, 81.1, 73.7, 71.6, 64.4, 62.9, 62.2, 60.9, 48.4, 44.4, 36.8, 36.0, 34.3, 33.9, 29.4, 29.2, 2X 28.8, 28.3, 27.9, 27.3, 24.8, 24.5; due to the presence of Boc rotamers in the NMR measurement, structural assignment is not possible; HR-MS (ESI): (*m*/*z*) = calculated for C_15_H_29_N_2_O_2_^+^ [*M*+H]^+^: 269.2224, found: 269.2224.

#### 6-Benzyldodecahydroazepino[3,2-*b*]azepine ((±)-**44b**)

To a solution of mono-Boc protected diamine (±)-**43** (15.4 mg, 0.06 mmol 1.0 equiv) in MeOH (1 mL) was added benzyl bromide (8 μL, 0.07 mmol, 1.2 equiv) and K_2_CO_3_ (12.4 mg, 0.09 mmol, 1.5 equiv). The mixture was stirred at 22 °C for 24 h. Then, the solvent was evaporated, and the residue was diluted with dionized H_2_O and extracted with EtOAc (3 × 5 mL). The organic layers were dried over Na_2_SO_4_ and filtered, and the solvent was removed in vacuo to give a yellow oil. The intermediate product was dissolved in DCM (1 mL), and TFA (0.1 mL, 10 vol %) was added. The reaction was stirred at 22 °C for 2 h. Then, it was washed with NaOH (1 M, 2 × 2 mL); the organic phase was dried over Na_2_SO_4_ and filtered, and the solvent was evaporated. The crude was purified by column chromatography on silica gel (5% MeOH in DCM + 1% NH_3_) to give monobenzylated diamine (±)-**44b** (9.3 mg, 0.04 mmol, 67%) as a brown oil. R_f_ = 0.20 (5% MeOH in DCM + 1% NH_3_); ^1^H NMR (400 MHz, MeOD-*d*_4_, 298 K): δ [ppm] = 7.33–7.35 (m, 2H; H_2_-*C*_arom._), 7.26–7.29 (m, 2H; H_2_-*C*_arom._), 7.19–7.21 (m, 1H; H-*C*_arom_), 3.83 (d, *J* = 13.7, 1H; H-*C*_benz._), 3.69 (d, *J* = 13.7, 1H; H-*C*_benz._) 2.84–2.92 (m, 3H; H-*C*(5a and 7)), 2.73–2.79 (m, 2H; H-*C*(2 and 10a)), 2.53–2.59 (m, 1H; H-*C* (2)), 2.07–2.13 (m, 1H; H-*C* (3, 4, 5, 8, 9 or 10)), 1.97–2.05 (m, 1H; H-*C* (3, 4, 5, 8, 9 or 10)), 1.75–1.83 (m, 2H; H-*C* (3, 4, 5, 8, 9 or 10)), 1.62–1.74 (m, 4H; H-*C* (3, 4, 5, 8, 9 or 10)), 1.41–1.61 (m, 4H; H-*C* (3, 4, 5, 8, 9 or 10)); ^13^C NMR (100 MHz, MeOD-*d*_4_, 298 K): δ [ppm] = 142.1 ((C_q_)_arom._), 129.7 ((*C*H)_arom._), 129.2 ((*C*H)_arom._), 127.8 ((*C*H)_arom._), 68.4 (C(10a)), 64.9 (C(5a)), 58.1 (*C*H_2_)_benz._), 50.4 (C (2)), 48.5 (C (7)), 35.6 (C (3, 4, 5, 8, 9 or 10), 31.0 (C (3, 4, 5, 8, 9 or 10), 28.5 (C (3, 4, 5, 8, 9 or 10), 28.4 (C (3, 4, 5, 8, 9 or 10), 27.2 (C (3, 4, 5, 8, 9 or 10)), 25.9 (C (3, 4, 5, 8, 9 or 10)); HR-MS (ESI): (*m*/*z*) = calculated for C_17_H_27_N_2_^+^ [*M*+H]^+^: 259.2169, found: 259.2165.

#### 1-Benzhydryloctahydro-4H-indol-4-one ((±)-**45**)

To a solution of Boc-protected amine (±)-**8** (1.19 g, 4.97 mmol, 1.0 equiv) in DCM (50 mL, 0.1 M) was added TFA (5.0 mL, 10 vol %). The reaction was stirred at 22 °C for 2 h. Then it was evaporated to yield a colorless oil. To a solution of intermediate product in DMF (18 mL) was added Cs_2_CO_3_ (4.20 g, 12.9 mmol, 2.5 equiv) and bromodiphenylmethane (3.19 g, 12.9 mmol, 2.5 equiv). The reaction mixture was stirred at 60 °C for 24 h. Then, the solvent was evaporated, and the residue was taken up in dionized H_2_O (50 mL) and extracted with EtOAc (3 × 100 mL). The organic layers were dried over Na_2_SO_4_ and filtered, and the solvent was removed in vacuo. The crude was purified by column chromatography on silica gel (4% acetone in heptane) to give diphenylmethane protected amine (±)-**45** (117 mg, 0.38 mmol, 8%) as an orange solid. R_f_ = 0.20 (4% acetone in heptane); ^1^H NMR (400 MHz, MeOD-*d*_4_, 298 K): δ [ppm] = 7.13–7.42 (m, 10H; H-*C*_arom._), 4.82 (s, 1H; *C*H), 3.16–3.23 (m, 1H; H-*C*(7a)), 2.78–2.89 (m, 2H; H-*C*(2 and 3a)), 2.34–2.50 (m, 2H; H-*C* (2 and 5)), 2.24–2.31 (m, 1H; H-*C* (5)), 2.07–2.22 (m, 1H; H-*C* (3)), 1.74–1.93 (m, 2H; H-*C* (3 and 6)), 1.63–1.71 (m, 2H; H_2_-*C* (7)), 1.42–1.53 (m, 1H; H-*C* (6)); ^13^C NMR (100 MHz, MeOD-*d*_4_, 298 K): δ [ppm] = 215.3 (C (4)), 144.8 ((C_q_)_arom._), 143.1 ((C_q_)_arom._), 129.6 ((*C*H)_arom._), 129.3 ((*C*H)_arom._), 129.2 ((*C*H)_arom._), 129.0 ((*C*H)_arom._), 128.1 ((*C*H)_arom._), 127.9 ((*C*H)_arom._), 71.3 (*C*H), 64.5 (C(7a)), 52.8 (C(3a)), 49.7 (C (2)), 40.1 (C (5)), 26.2 (C (3)), 25.9 (C (7)), 22.1 (C (6)); HR-MS (ESI): (*m*/*z*) = calculated for C_21_H_24_NO^+^ [*M*+H]^+^: 306.1852, found: 306.1842.

#### 1-Benzhydryloctahydro-4*H*-indol-4-one ((±)-**46**)

Ketone (±)-**45** (85.5 mg, 0.28 mmol, 1.0 equiv) was dissolved in pyridine (3 mL, 0.1 M), and NH_2_OH•HCl (38.9 mg, 0.56 mmol, 2.0 equiv) was added. The mixture was stirred at 22 °C for 2 h, then the solvent was evaporated, and the residue was taken up in dionized H_2_O (5 mL) and extracted with EtOAc (3 × 10 mL). The combined organic layers were dried over Na_2_SO_4_ and filtered, and the solvent was evaporated to yield a yellow solid. To a solution of oxime isomers in pyridine (2 mL, 0.1 M) was added freshly recrystallized *p*-TsCl (64.8 mg, 0.34 mmol, 1.2 equiv), and the solution was stirred at 22 °C for 2 h. After completion, the solvent was evaporated, and the crude was taken up in dionized H_2_O (5 mL) and extracted with EtOAc (3 × 10 mL). The organic phases were dried over Na_2_SO_4_ and filtered, and the solvent was evaporated to yield a yellow sticky solid. The intermediate product was further reacted with potassium acetate (50.1 mg, 0.51 mmol, 3.0 equiv) in a mixture of EtOH (2.5 mL) and dionized H_2_O (2.5 mL). The reaction was stirred and refluxed for 16 h. After cooling to room temperature and evaporation of the EtOH, the remaining aqueous solution was adjusted to pH = 10 by the addition of aqueous NaOH solution (1 M, 0.5 mL). The aqueous phase was extracted with DCM (3 × 10 mL). The combined organic layers were dried over Na_2_SO_4_, filtered and the solvent was removed under reduced pressure. The crude product was purified by flash column chromatography on silica gel (80% EtOAc in heptane) to isolate lactam (±)-**46** (21.4 mg, 0.07 mmol, 24%) as a white solid. R_f_ = 0.20 (80% EtOAc in heptane); ^1^H NMR (400 MHz, MeOD-*d*_4_, 298 K): δ [ppm] = 7.18–7.41 (m, 10H; H-*C*_arom._), 4.81 (s, 1H; *C*H), 4.00 (q, *J* = 7.8 Hz, 1H; H-*C*(3a)), 2.89–2.96 (m, 1H; H-*C* (2)), 2.63–2.70 (m, 1H; H-*C*(8a)), 2.43–2.53 (m, 1H; H-*C* (6)), 2.06–2.23 (m, 3H; H-*C* (2, 3 and 6)), 1.67–1.80 (m, 1H; H-*C* (3)), 1.52–1.63 (m, 2H; H-*C* (7 and 8)), 1.33–1.49 (m, 2H; H-*C* (7 and 8)); ^13^C NMR (100 MHz, MeOD-*d*_4_, 298 K): δ [ppm] = 177.6 (C (5)), 144.4 ((C_q_)_arom._), 142.0 ((C_q_)_arom._), 130.2 ((*C*H)_arom._), 129.4 ((*C*H)_arom._), 129.2 ((*C*H)_arom._), 129.1 ((*C*H)_arom._), 128.2 ((*C*H)_arom._), 128.0 ((*C*H)_arom._), 72.1 (*C*H), 64.5 (C(8a)), 55.7 (C(3a)), 50.2 (C (2)), 33.3 (C (6)), 31.1 (C (3)), 26.8 (C (7)), 19.7 (C (8)); HR-MS (ESI): (*m*/*z*) = calculated for C_21_H_25_N_2_O^+^ [*M*+H]^+^: 321.1961, found: 321.1955.

#### 1-Benzhydryldecahydropyrrolo[3,2-*b*]azepine ((±)-**47**)

A solution of lactam (±)-**46** (7.7 mg, 0.02 mmol, 1.0 equiv) in dry THF (1 mL) was cooled to 0 °C, then LiAlH_4_ (1 M in THF, 60 μL, 0.06 mmol, 3.0 equiv) was added dropwise, and the reaction was stirred at 22 °C for 4 h. The reaction was then worked up using Fieser's protocol, and the crude was purified by flash column chromatography on silica gel (5% MeOH in DCM + 0.1% NEt_3_) to give azepane (±)-**47** (6.6 mg, 0.02 mmol, quant.) as a light brown oil. R_f_ = 0.20 (5% MeOH in DCM + 0.1% NEt_3_); ^1^H NMR (400 MHz, MeOD-*d*_4_, 298 K): δ [ppm] = 7.16–7.43 (m, 10H; H-*C*_arom._), 4.83 (s, 1H; *C*H), 3.50 (q, *J* = 8.3 Hz, 1H; H-*C*(3a)), 3.11–3.16 (m, 1H; H-*C* (5)), 2.81–2.89 (m, 2H; H-*C*(2 and 8a)), 2.48–2.55 (m, 1H; H-*C* (5)), 2.19–2.25 (m, 1H; H-*C* (2)), 2.04–2.10 (m, 1H; H-*C* (3)), 1.62–1.72 (m, 4H; H-*C* (3, 6, 7 and 8)), 1.48–1.61 (m, 2H; H-*C* (6 and 8)), 1.00–1.10 (m, 1H; H-*C* (7)); ^13^C NMR (100 MHz, MeOD-*d*_4_, 298 K): δ [ppm] = 144.5 ((C_q_)_arom._), 142.2 ((C_q_)_arom._), 130.1 ((*C*H)_arom._), 129.4 ((*C*H)_arom._), 129.2 ((*C*H)_arom._), 129.1 ((*C*H)_arom._), 128.1 ((*C*H)_arom._), 127.9 ((*C*H)_arom._), 71.9 (*C*H), 67.2 (C(8a)), 63.0 (C(3a)), 50.0 (C (2)), 49.5 (C (5)), 31.9 (C (3 or 6)), 31.7 (C (3 or 6)), 29.5 (C (8)), 26.2 (C (7)); HR-MS (ESI): (*m*/*z*) = calculated for C_21_H_27_N_2_^+^ [*M*+H]^+^: 307.2169, found: 307.2173.

#### 6-Methoxy-2,3,4,5-tetrahydro-1*H*-benzo[*b*]azepine (**48**)

A solution of lactam **31** (5.67 g, 29.7 mmol, 1.0 equiv) in dry THF (120 mL) was cooled to 0 °C. Then, LiAlH_4_ (1 M in THF, 267 mL, 267 mmol, 9.0 equiv) was added dropwise through a dropping funnel. The mixture was stirred at reflux for 15 h. Then, it was cooled down and worked up using Fieser’s protocol. The crude product was purified by flash column chromatography on silica gel (20% EtOAc in heptane +0.1% NEt_3_) to afford azepane **48** (4.29 g, 24.2 mmol, 81%) as a yellow solid. R_f_ = 0.16 (20% EtOAc in heptane +0.1% NEt_3_); ^1^H NMR (400 MHz, CDCl_3_, 298 K): δ [ppm] = 6.99 (t, *J* = 8.0 Hz, 1H; H-*C* (8)), 6.52 (dd, 4H, *J* = 8.1 Hz, 1H; H-*C* (7 and 9)), 3.80 (s, 3H; H_3_-*C*), 3.10 (t, *J* = 5.5 Hz, 2H; H-*C* (2)), 2.90 (t, *J* = 5.6 Hz, 2H; H-*C* (5)), 1.82–1.88 (m, 2H; H-*C* (3)), 1.60–1.65 (m, 2H; H-*C* (4)); ^13^C NMR (100 MHz, CDCl_3_, 298 K): δ [ppm] = 158.1 (C (6)), 150.6 (C(9a)), 126.6 (C (8)), 122.6 (C(5a)), 113.0 (C (7 or 9)), 104.8 (C (7 or 9)), 56.0 (*C*H_3_), 49.0 (C (2)), 31.5 (C (3)), 26.0 (C (4)), 24.8 (C (5)); HR-MS (ESI): (*m*/*z*) = calculated for C_11_H_16_NO^+^ [*M*+H]^+^: 178.1226, found: 178.1221.

#### Trifluoro-1-(6-methoxy-2,3,4,5-tetrahydro-1*H*-benzo[b]azepin-1-yl)ethanone (**49**)

To a solution of azepane **48** (4.28 g, 24.1 mmol, 1.0 equiv) in DCM (240 mL, 0.1 M) was added TFAA (4.02 mL, 28.9 mmol, 1.2 equiv) and pyridine (2.33 mL, 28.9 mmol, 1.2 equiv). The mixture was stirred at 22 °C for 2 h. The organic phase was washed with dionized H_2_O (130 mL) and brine (130 mL), dried over Na_2_SO_4_, and filtered, and the solvent was removed in vacuo to give trifluoroacetamide-protected amine **49** (6.49 g, 23.8 mmol, 99%) as a yellow solid. R_f_ = 0.24 (20% EtOAc in heptane); ^1^H NMR (400 MHz, CDCl_3_, 298 K): δ [ppm] = 7.20–7.25 (m, 1H; H-*C* (8); 7.02–7.05 (m, 1H; H-*C* (7), 6.83 (d, *J* = 7.8 Hz, 1H; H-*C* (9), 4.55–4.59 (m, 1H; H-*C* (2)), 3.85 (s, 3H; H_3_-*C*), 3.38–3.44 (m, 1H; H-*C* (5)), 2.80–2.87 (m, 1H; H-*C* (2)), 2.22–2.30 (m, 1H; H-*C* (5)), 1.90–2.01 (m, 2H; H-*C* (3 and 4)), 1.81–1.85 (m, 1H; H-*C* (3)), 1.26–1.37 (m, 1H; H-*C* (4)); ^13^C NMR (100 MHz, CDCl_3_, 298 K): δ [ppm] = 158.6 (C (6)), 156.8 (q, *J*_C–F_ = 35.3 Hz, *C*OCF_3_), 142.7 (C(9a)), 130.2 (C(5a)), 128.3 (C (8)), 120.4 (C (9)), 117.8 (q, *J*_C–F_ = 287.7 Hz, CO*C*F_3_), 112.7 (C (7)), 56.5 (*C*H_3_), 51.0 (C (2)), 29.6 (C (3)), 26.6 (C (4)), 24.8 (C (5)); ^19^F-NMR (300 MHz, CDCl_3_, 298 K): δ [ppm] = −69.7; HR-MS (ESI): (*m*/*z*) = calculated for C_13_H_15_NO_2_F_3_^+^ [*M*+H]^+^: 274.1049, found: 274.1049, C_13_H_14_NO_2_Na^+^ [*M*+Na]^+^: 296.0874, found: 296.0866.

#### Trifluoro-1-(6-hydroxy-2,3,4,5-tetrahydro-1*H*-benzo[*b*]azepin-1-yl)ethanone (**50**)

To a solution of **49** (5.73 g, 21.0 mmol, 1.0 equiv) in dry DCM (125 mL) was added BBr_3_ (1 M in DCM, 63 mL, 63 mmol, 3 equiv) at −78 °C. The mixture was warmed to 22 °C and stirred for 24 h. Then it was cooled to 0 °C and quenched by the addition of dionized H_2_O (40 mL). The precipitate was filtered, washed with dionized H_2_O, dried, and purified by flash column chromatography on silica gel (20% EtOAc in heptane to EtOAc) to yield alcohol **50** (5.07 g, 19.6 mmol, 93%) as a light brown solid. R_f_ = 0.23 (20% EtOAc in heptane); ^1^H NMR (400 MHz, MeOD-*d*_4_, 298 K): δ [ppm] = 7.04 (t, *J* = 8.0 Hz, 1H; H-*C* (8); 6.83–6.85 (m, 1H; H-*C* (7 or 9), 6.68–6.70 (m, 1H; H-*C* (7 or 9), 4.57 (dt, *J* = 13.2, 3.3 Hz, 1H; H-*C* (2)), 3.38 (dd, *J* = 13.7, 5.8 Hz, 1H; H-*C* (5)), 2.80–2.87 (m, 1H; H-*C* (2)), 2.25 (t, *J* = 9.06 Hz, 1H; H-*C* (5)), 1.89–2.01 (m, 2H; H-*C* (3 and 4)), 1.81–1.87 (m, 1H; H-*C* (3)), 1.29–1.40 (m, 1H; H-*C* (4)); ^13^C NMR (100 MHz, MeOD-*d*_4_, 298 K): δ [ppm] = 156.9 (q, *J*_C–F_ = 34.1 Hz, *C*OCF_3_), 156.4 (6), 142.9 (C(9a)), 128.3 (C(5a)), 127.9 (C (8)), 119.1 (C (7 or 9)), 117.9 (q, *J*_C–F_ = 284.6 Hz, CO*C*F_3_), 117.0 (C (7 or 9)), 51.1 (C (2)), 29.6 (C (3)), 26.8 (C (4)), 25.0 (C (5)); ^19^F/NMR (300 MHz, MeOD-*d*_4_, 298 K): δ [ppm] = −69.6; HR-MS (ESI): (*m*/*z*) = calculated for C_12_H_13_NO_2_F_3_^+^ [*M*+H]^+^: 260.0893, found: 260.0888, C_12_H_12_NO_2_F_3_Na^+^ [*M*+Na]^+^: 282.0718, found: 282.0705.

#### Trifluoro-1-(6-hydroxydecahydro-1*H*-benzo[*b*]azepin-1-yl)ethenone ((±)-**51**)

A mixture of aromatic compound (±)-**50** (5.07 g, 19.6 mmol, 1.0 equiv), Rh/C (5%, Rh on activated charcoal, 0.51 g, 10 wt % of substrate) and AcOH (2.24 mL, 39.2 mmol, 2.0 equiv) in *^i^*PrOH (50 mL) was stirred in a sealed autoclave at 70 °C under H_2_ pressure (25 bar) for 2 days. After cooling to room temperature, the reaction mixture was filtered over Celite, washed with MeOH, and evaporated to dryness to afford aliphatic bicycle (±)-**51** (5.20 g, 19.6 mmol, quant.) as a colorless oil. R_f_ = 0.23 (20% EtOAc in heptane); ^1^H NMR (400 MHz, MeOD-*d*_4_, 298 K): δ [ppm] = 4.96–5.01 (m, 0.6H), 4.45–4.49 (m, 1H), 3.90–4.03 (m, 1.7H), 3.81–3.85 (m, 1.5H), 3.69–3.78 (m, 1.6H), 3.64–3.68 (m, 0.3H), 3.42–3.50 (m, 1H), 3.26–3.28 (m, 1H), 3.23–3.24 (m, 0.3H), 1.14–2.08 (m, 35H); ^13^C NMR (100 MHz, MeOD-*d*_4_, 298 K): δ [ppm] = 158.0 (q, *J*_C–F_ = 45.9 Hz, *C*OCF_3_), 118.3 (q, *J*_C–F_ = 288.8 Hz, CO*C*F_3_), 75.2, 73.9, 73.5, 72.9, 72.4, 70.0, 2X 58.7, 57.8, 2X 54.9, 54.4, 51.5, 51.3, 46.8, 46.6, 46.1, 42.6, 2X 41.9, 2X 41.7, 41.3, 40.7, 36.7, 34.0, 32.9, 32.3, 32.1, 31.4, 30.2, 30.1, 29.5, 2X 29.3, 2X 28.5, 28.3, 27.9, 27.8, 26.7, 26.6, 26.3, 26.2, 26.1, 25.7, 25.4, 2X 25.3, 2X 25.1, 25.0, 24.8, 24.3, 23.3, 23.0, 21.8, 21.7, 21.0, 20.9, 20.6, 20.5, 19.2; due to the presence of alcohol isomers and trifluoroacetamide rotamers in the NMR measurement, structural assignment is not possible; HR-MS (ESI): (*m*/*z*) = calculated for C_12_H_19_NO_2_F_3_^+^ [*M*+H]^+^: 266.1373, found: 266.1363, C_12_H_18_NO_2_F_3_Na [*M*+Na]^+^: 288.1187, found: 288.1179.

#### *tert*-Butyl-6-hydroxydecahydro-1*H*-benzo[*b*]azepine-1-carboxylate ((±)-**52**)

Trifluoroacetamide-protected amine (±)-**51** (1.05 g, 3.96 mmol, 1.0 equiv) was dissolved in a mixture of THF and dionized H_2_O (20 mL, 1:1), and LiOH (0.47 mg, 19.8 mmol, 5.0 equiv) was added. The mixture was refluxed for 24 h. Then, it was cooled to room temperature and concentrated under reduced pressure. The aqueous phase was extracted with EtOAc (3 × 25 mL). The organic layers were dried over Na_2_SO_4_ and filtered, and the solvent was removed in vacuo to obtain a brown solid. To a solution of intermediate product in DCM (40 mL, 0.1 M) was added Boc_2_O (1.30 g, 5.94 mmol, 1.5 equiv), NEt_3_ (658 μL, 4.75 mmol, 1.2 equiv), and DMAP (cat.). The mixture was stirred at 22 °C for 24 h. Then the solvent was evaporated, and the crude was purified by flash column chromatography on silica gel (20% EtOAc in heptane) to isolate Boc-protected amine (±)-**52** (0.67 g, 2.49 mmol, 63%) as a colorless oil**.** R_f_ = 0.23 (20% EtOAc in heptane); ^1^H NMR (400 MHz, MeOD-*d*_4_, 298 K): δ [ppm] = 4.52 (br, 0.5H), 3.98–4.09 (m, 0.5H), 3.67–3.81 (m, 2H), 3.01–3.09 (m, 1H), 1.73–1.93 (m, 6H), 1.19–1.66 (m, 22H); ^13^C NMR (100 MHz, MeOD-*d*_4_, 298 K): δ [ppm] = 157.4, 80.8, 75.2, 73.4, 72.7, 70.0, 56.1, 52.5, 46.1, 42.7, 41.3, 40.3, 36.7, 36.6, 34.0, 33.0, 32.4, 31.4, 30.8, 29.7, 29.0, 28.8, 27.9, 27.4, 26.8, 26.2, 26.1, 25.3, 24.8, 23.5; due to the presence of alcohol isomers and Boc rotamers in the NMR measurement, structural assignment is not possible; HR-MS (ESI): (*m*/*z*) = calculated for C_15_H_28_NO_3_^+^ [*M*+H]^+^: 270.2064, found: 270.2064.

#### *tert*-Butyl-6-oxodecahydro-1*H*-benzo[*b*]azepine-1-carboxylate ((±)-**53**)

A solution of alcohol (±)-**52** (0.67 g, 2.49 mmol, 1.0 equiv) in DCM (25 mL, 0.1 M) was cooled to 0 °C, and DMP (1.58 g, 3.74 mmol, 1.5 equiv) was added. The reaction was stirred at 22 °C for 2 h. Then it was quenched by the addition of sat. NaHCO_3_ solution (10 mL) and Na_2_S_2_O_3_ solution (2 M, 10 mL). The water phase was washed three times with EtOAc (3 × 20 mL). The combined organic phases were washed with dionized H_2_O, dried over Na_2_SO_4_ and filtered, and the solvent was evaporated in vacuo. The crude was purified by column chromatography on silica gel (20% EtOAc in heptane) to yield ketone (±)-**53** (0.36 g, 1.35 mmol, 54%) as a yellow solid. R_f_ = 0.23 (20% EtOAc in heptane); ^1^H NMR (400 MHz, MeOD-*d*_4_, 298 K): δ [ppm] = 4.41 (quint., J = 5.1 Hz, 1H; H-*C*(9a)), 3.68–3.72 (m, 1H; H-*C* (2)), 3.00–3.04 (m, 1H; H-*C* (2)), 2.64–2.69 (m, 1H; H-*C*(9a)), 2.36–2.45 (m, 1H; H-*C* (7)), 2.25–2.33 (m, 1H; H-*C* (7)), 2.07–2.15 (m, 1H), 1.87–2.02 (m, 3H), 1.76–1.84 (m, 2H), 1.66–1.72 (m, 1H), 1.53–1.65 (m, 2H), 1.46 (s, 9H; H_3_-*C*_Boc_), 1.37–1.44 (m, 1H); ^13^C NMR (100 MHz, MeOD-*d*_4_, 298 K): δ [ppm] = 214.3 (C (6)), 157.1 ((*C*=O)_Boc_, 81.4 ((C_q_)_Boc_), 57.9 (C(9a)), 56.2 (C(5a)), 43.7 (C (2)), 39.9 (C (7)), 28.9 (*C*H_2_), 28.8 (*C*H_2_), 28.7 ((*C*H_3_)_Boc_), 28.1 (*C*H_2_), 25.1 (*C*H_2_), 22.2 (*C*H_2_); HR-MS (ESI): (*m*/*z*) = calculated for C_15_H_26_NO_3_^+^ [*M*+H]^+^: 268.1907, found: 268.1900.

#### *tert*-Butyl-(*E*)-6-(hydroxyimino)decahydro-1*H*-benzo[*b*]azepine-1-carboxylate ((±)-*E***-54**)

To a solution of ketone (±)-**53** (687 mg, 2.57 mmol, 1.0 equiv) in MeOH (10 mL) and dionized H_2_O (10 mL) was added NH_2_OH·HCl (536 mg, 7.71 mmol, 3.0 equiv) and sodium acetate (632 mg, 7.71 mmol, 5.0 equiv). The mixture was refluxed for 4 h. The reaction was allowed to cool to room temperature, and the MeOH was evaporated. The remaining aqueous phase was extracted with EtOAc (3 × 20 mL); the organic layers were dried over Na_2_SO_4_ and filtered, and the solvent was removed in vacuo. The crude product was purified by flash column chromatography on silica gel (20% EtOAc in heptane) to isolate *(E)*-oxime isomer (±)-**54** (139 mg, 0.49 mmol, 19%, ratio 1:1.3) was isolated as a colorless oil. R_f_ = 0.23 (60% EtOAc in heptane); ^1^H NMR (400 MHz, DMSO-*d*_6_, 333 K): δ [ppm] = 10.05 (s, 1H, *O*H), 4.02–4.05 (m, 1H; H-*C*(5a or 9a)), 3.62 (br, 1H), 3.00–3.08 (m, 2H), 1.75–1.99 (m, 5H), 1.62–1.70 (m, 1H), 1.48–1.53 (m, 2H), 1.41 (s, 9H; H_3_*C*-Boc), 1.17–1.19 (m, 3H); ^13^C NMR (100 MHz, DMSO-*d*_6_, 333 K): δ [ppm] = 159.2 (C (6)), 154.3 ((*C*=O)_Boc_), 78.2 ((C_q_)_Boc_), 46.7 *(CH)*, 39.8 (*C*H_2_), 2X 27.8 ((*C*H_3_)_Boc_) and (*C*H_2_), 26.0 (*C*H_2_), 24.9 (*C*H_2_), 24.0 (*C*H_2_), 22.6 (*C*H_2_), 19.9 (*C*H_2_); 1 X C*H* is hidden behind the DMSO peak, 1 *C*H is not visible; HR-MS (ESI): (*m*/*z*) = calculated for C_15_H_27_N_2_O_3_^+^ [*M*+H]^+^: 282.2016, found: 283.2013, C_15_H_26_N_2_O_3_Na [*M*+Na]^+^: 305.1841, found: 305.1832.

#### *tert*-Butyl-(*Z*)-6-(hydroxyimino)decahydro-1*H*-benzo[*b*]azepine-1-carboxylate ((±)-*Z***-54)**

(*Z*)-Oxime isomer (±)-**54** (181 mg, 0.64 mmol, 25%, ratio 1.3:1) was isolated as a white solid from the above reaction. The (*Z*)*-*oxime crystallized spontaneously and allowed to determine the *syn* ring junction by X-ray diffraction studies. R_f_ = 0.24 (60% EtOAc in heptane); ^1^H NMR (400 MHz, DMSO-*d*_6_, 333 K): δ [ppm] = 10.08 (s, 1H, *O*H), 3.99 (br, 1H; H-*C*(5a or 9a)), 3.62 (br, 1H), 3.44 (br, 1H; H-*C*(5a or 9a)), 3.04–3.10 (m, 1H), 2.00–2.12 (m, 2H), 1.84–1.98 (m, 3H), 1.76–1.80 (m, 1H), 1.57–1.66 (m, 1H), 1.45–1.52 (m, 2H), 1.41 (s, 9H; H_3_*C*-Boc), 1.29–1.34 (m, 1H), 1.16–1.27 (m, 2H); ^13^C NMR (100 MHz, DMSO-*d*_6_, 333 K): δ [ppm] = 158.6 (C (6)), 154.3 ((*C*=O)_Boc_), 78.1 ((C_q_)_Boc_), 39.7 (*C*H_2_), 2X 27.8 ((*C*H_3_)_Boc_) and (*C*H_2_), 27.7 (*C*H_2_), 25.8 (*C*H_2_), 24.9 (*C*H_2_), 24.3 (*C*H_2_), 23.8 (*C*H_2_); 2 *C*H are not visible; HR-MS (ESI): (*m*/*z*) = calculated for C_15_H_27_N_2_O_3_^+^ [*M*+H]^+^: 282.2016, found: 283.2012, C_15_H_26_N_2_O_3_Na [*M*+Na]^+^: 305.1841, found: 305.1831.

#### *tert*-Butyl-(3a*R*,8a*R*)-4-(4-chlorobenzyl)octahydropyrrolo[3,2-*b*]azepine-1(2*H*)-carboxylate ((*R,R*)*-***55**)

To a solution of orthogonally protected (*R,R*)*-***35** (43.0 mg, 0.13 mmol, 1.0 equiv) in MeOH (1.5 mL, 0.1 M) was added Pd/C (10%, Pd on activated charcoal, 4.5 mg, 10 wt % of substrate). The mixture was stirred at 22 °C under an atmosphere of hydrogen (1 atm, balloon) for 24 h. Then it was filtered over Celite and evaporated to dryness to yield a colorless oil. To a solution of intermediate product in MeOH (1.5 mL, 0.1 M) was added 4-chlorobenzaldehyde (22.5 mg, 0.16 mmol, 1.2 equiv) and NaBH_3_CN (10.1 mg, 0.16 mmol, 1.2 equiv). The mixture was refluxed for 24 h. After cooling to room temperature, it was quenched by the addition of NaOH (1 M, 2 mL). Then the solvent was concentrated under reduced pressure and the aqueous phase was extracted with EtOAc (3 × 10 mL). The organic layers were dried over Na_2_SO_4_ and filtered, and the solvent was removed in vacuo. The crude product was purified by flash chromatography on silica gel (5% EtOAc in heptane) to afford derivatized diamine (*R,R*)-**55** (4.2 mg, 0.01 mmol, 8%) as a yellow oil. R_f_ = 0.20 (5% EtOAc in heptane); ^1^H NMR (400 MHz, MeOD-*d*_4_, 298 K): δ [ppm] = 7.35–7.38 (m, 2H; H_2_-*C*_arom._), 7.29–7.31 (m, 2H; H_2_-*C*_arom._), 3.92 (d, *J* = 13.9 Hz, 1H; H-*C*_Cl.-benz._), 3.77–3.83 (m, 1H; H-*C*(8a)), 3.42–3.49 (m, 1H; H-*C* (2)), 3.35 (d, *J* = 14.0, 1H; H-*C*_Cl.-benz._), 3.17–3.27 (m, 2H; H-*C*(2 and 3a)), 2.56–2.60 (m, 1H; H-*C* (5)), 2.36–2.42 (m, 1H; H-*C* (5)), 2.21–2.26 (m, 1H; H-*C* (8)), 1.90–2.04 (m, 2H; H-*C* (3 and 8)), 1.65–1.78 (m, 2H; H-*C* (3 and 7)), 1.51–1.52 (m, 1H; H-*C* (6)), 1.46 (s, 9H; H_3_-*C*_Boc_), 1.33–1.38 (m, 2H; H-*C* (6 and 7)); ^13^C NMR (100 MHz, MeOD-*d*_4_, 298 K): δ [ppm] = 156.3 ((*C*=O)_Boc_, 140.2 ((C_q_)_arom._), 135.6 ((C_q_)_arom._), 131.4 ((*C*H)_arom._), 129.2 ((*C*H)_arom._), 80.8 ((C_q_)_Boc_), 68.0 (C(3a))_rot._, 67.5 (C(3a))_rot._, 64.1 (C(8a))_rot._, 63.7 (C(8a))_rot._, 60.5 (*C*H_2_)_Cl.-benz._), 53.6 (C (5)), 45.5 (C (2))_rot._, 44.9 (C (2))_rot._, 32.4 (C (6)), 31.1 (C (3 or 8))_rot._, 30.4 (C (3 or 8))_rot._, 30.3 (C (3 or 8))_rot._, 28.8 ((*C*H_3_)_Boc_), 28.1 (C (7))_rot._, 28.0 (C (7))_rot._; HR-MS (ESI): (*m*/*z*) = calculated for C_20_H_30_N_2_O_2_Cl^+^ [*M*+H]^+^: 365.1990, found: 365.1981.

#### (3a*R*,8a*R*)-4-(4-Chlorobenzyl)decahydropyrrolo[3,2-*b*]azepine ((*R,R*)-**58**)

To a solution of Boc-protected amine (*R,R*)-**55** (4.2 mg, 0.01 mmol, 1.0 equiv) in DCM (1 mL) was added TFA (0.1 mL, 10 vol %). The reaction was stirred at 22 °C for 2 h. Then, the solvent was evaporated, and the crude was purified by flash chromatography on silica gel (5%–10% MeOH in DCM + 1% NH_3_) and treated with TFA to afford derivatized diamine (*R,R*)-**58** (4.9 mg, 0.01 mmol, quant.) as a TFA salt and yellow solid. R_f_ = 0.20 (10% MeOH in DCM + 1% NH_3_); ^1^H NMR (400 MHz, MeOD-*d*_4_, 298 K): δ [ppm] = 7.36–7.38 (m, 2H; H_2_-*C*_arom._), 7.31–7.33 (m, 2H; H_2_-*C*_arom._), 3.90 (d, *J* = 13.9 Hz, 1H; H-*C*_Cl.-benz._), 3.72–3.77 (m, 1H; H-*C*(8a)), 3.41–3.50 (m, 3H; H-*C*_Cl.-benz._ and H-*C*(2 and 3a), 3.15–3.24 (m, 1H; H-*C* (2)), 2.71–2.77 (m, 1H; H-*C* (5)), 2.34–2.44 (m, 2H; H-*C* (3 and 5)), 2.17–2.27 (m, 1H; H-*C* (8)), 1.96–2.06 (m, 1H; H-*C* (3)), 1.78–1.89 (m, 2H; H-*C* (7 and 8)), 1.54–1.58 (m, 1H; H-*C* (6)), 1.42–1.50 (m, 2H; H-*C* (6 and 7)); ^13^C NMR (100 MHz, MeOD-*d*_4_, 298 K): δ [ppm] = 139.8 ((C_q_)_arom._), 133.8 ((C_q_)_arom._), 131.3 ((*C*H)_arom._), 129.4 ((*C*H)_arom._), 66.9 (C(3a)), 64.5 (C(8a)), 60.0 (*C*H_2_)_Cl.-benz._)), 54.6 (C (5)), 44.5 (C (2)), 31.3 (C (6)), 31.0 (C (3)), 29.3 (C (8)), 26.8 (C (7)); HR-MS (ESI): (*m*/*z*) = calculated for C_15_H_22_N_2_Cl^+^ [*M*+H]^+^: 265.1466, found: 265.1463.

#### *tert*-Butyl (3a*R*,8a*R*)-4-(3-chlorobenzyl)octahydropyrrolo[3,2-*b*]azepine-1(2*H*)-carboxylate ((*R,R*)-**56**)

To a solution of orthogonally protected (*R,R*)*-***35** (90 mg, 0.27 mmol, 1.0 equiv) in MeOH (2.7 mL, 0.1 M) was added Pd/C (10%, Pd on activated charcoal, 10 mg, 10 wt % of substrate). The mixture was stirred at 22 °C under an atmosphere of hydrogen (1 atm, balloon) for 24 h. Then it was filtered over Celite and evaporated to dryness to yield a colorless oil. To a solution of intermediate product in MeOH (2.5 mL, 0.1 M) was added 3-chlorobenzaldehyde (58 mg, 0.41 mmol, 1.5 equiv), NaBH_3_CN (26 mg, 0.41 mmol, 1.5 equiv), and AcOH (23 μL, 0.38 mmol, 1.5 equiv). The mixture was refluxed for 24 h. After cooling to room temperature, it was quenched by the addition of NaOH (1 M, 4 mL). Then, the solvent was concentrated under reduced pressure, and the aqueous phase was extracted with EtOAc (3 × 20 mL). The organic layers were dried over Na_2_SO_4_ and filtered, and the solvent was removed in vacuo. The crude product was purified by flash chromatography on silica gel (10% EtOAc in heptane) to afford derivatized diamine (*R,R*)-**56** (56 mg, 0.15 mmol, 56%) as a colorless oil. R_f_ = 0.22 (5% EtOAc in heptane); ^1^H NMR (400 MHz, MeOD-*d*_4_, 298 K): δ [ppm] = 7.41 (s, 1H; H-*C*_arom._), 7.27–7.29 (m, 2H; 2X H-*C*_arom._), 7.21–7.24 (m, 1H; H-*C*_arom._), 3.94 (d, *J* = 14.1 Hz, 1H; H-*C*_benz._), 3.78–3.84 (m, 1H; H-*C*(8a)), 3.42–3.48 (m, 1H; H-*C* (3)), 3.36 (d, *J* = 14.1, 1H; H-*C*_benz._), 3.16–3.28 (m, 2H; H-*C*(3 and 3a)), 2.55–2.59 (m, 1H; H-*C* (5)), 2.44–2.41 (m, 1H; H-*C* (5)), 2.21–2.23 (m, 1H; H-*C* (2)), 1.90–2.01 (m, 2H; H-*C* (2 and 8)), 1.69–1.76 (m, 2H; H-*C* (7 and 8)), 1.52–1.53 (m, 1H; H-*C* (6)), 1.46 (s, 9H; H_3_-*C*_Boc_), 1.30–1.42 (m, 2H; H-*C* (6 and 7)); ^13^C NMR (100 MHz, MeOD-*d*_4_, 298 K): δ [ppm] = 156.1 ((*C*=O)_Boc_, 144.0 ((C_q_)_arom._), 135.2 ((C_q_)_arom._), 130.7 ((*C*H)_arom._), 129.7 ((*C*H)_arom._), 128.2 ((*C*H)_arom._), 128.0 ((*C*H)_arom._), 80.8 ((C_q_)_Boc_), 68.0 (C(3a))_rot._, 67.5 (C(3a))_rot._, 64.1 (C(8a))_rot._, 63.7 (C(8a))_rot._, 60.8 (*C*H_2_)_benz._), 53.8 (C (5)), 45.4 (C (3))_rot._, 44.9 (C (3))_rot._, 32.4 (C (6)), 31.1 (C (2 or 8)), 30.4 (C (2 or 8))_rot._, 30.3 (C (2 or 8))_rot._, 28.8 ((*C*H_3_)_Boc_), 28.0 (C (7))_rot._, 27.9 (C (7))_rot._; HR-MS (ESI): (*m*/*z*) = calculated for C_20_H_30_N_2_O_2_Cl^+^ [*M*+H]^+^: 365.1990, found: 365.1983.

#### (3a*R*,8a*R*)-4-(3-chlorobenzyl)decahydropyrrolo[3,2-*b*]azepine ((*R,R*)-**59**)

To a solution of Boc-protected amine (*R,R*)-**56** (38 mg, 0.10 mmol, 1.0 equiv) in DCM (1 mL) was added TFA (0.1 mL, 10 vol %). The reaction was stirred at 22 °C for 2 h. Then, the solvent was evaporated, and the crude was purified by flash chromatography on silica gel (5%–10% MeOH in DCM + 1% NH_3_) to afford derivatized diamine (*R,R*)-**59** (19.9 mg, 0.08 mmol, 80%) as an orange oil. R_f_ = 0.25 (10% MeOH in DCM + 1% NH_3_); ^1^H NMR (400 MHz, MeOD-*d*_4_, 298 K): δ [ppm] = 7.41 (s, 1H; H-*C*_arom._), 7.28–7.31 (m, 2H; H-*C*_arom._), 7.21–7.26 (m, 1H; H-*C*_arom._), 3.90 (d, *J* = 14.1 Hz, 1H; H-*C*_benz._), 3.33–3.43 (m, 3H; H-*C*_benz._ and H-*C*(3a and 8a)), 3.17–3.23 (m, 1H; H-*C* (2), 2.88–2.95 (m, 1H; H-*C* (2)), 2.66–2.71 (m, 1H; H-*C* (5)), 2.32–2.38 (m, 1H; H-*C* (5)), 2.20–2.27 (m, 1H; H-*C* (3)), 2.05–2.15 (m, 1H; H-*C* (8)), 1.78–1.89 (m, 2H; H-*C* (3 and 7)), 1.66–1.71 (m, 1H; H-*C* (8)), 1.51–1.57 (m, 1H; H-*C* (6)), 1.35–1.49 (m, 2H; H-*C* (6 and 7)); ^13^C NMR (100 MHz, MeOD-*d*_4_, 298 K): δ [ppm] = 144.0 ((C_q_)_arom._), 135.2 ((C_q_)_arom._), 130.8 ((*C*H)_arom._), 129.6 ((*C*H)_arom._), 128.0 ((*C*H)_arom._), 128.0 ((*C*H)_arom._), 68.7 (C(3a)), 64.5 (C(8a)), 60.5 (*C*H_2_)_benz._)), 54.9 (C (5)), 45.0 (C (2)), 32.5 (C (3)), 31.7 (C (6)), 31.2 (C (8)), 27.3 (C (7)); HR-MS (ESI): (*m*/*z*) = calculated for C_15_H_22_N_2_Cl^+^ [*M*+H]^+^: 265.1466, found: 265.1472.

#### *tert*-Butyl-(3a*R*,8a*R*)-4-(2-chlorobenzyl)octahydropyrrolo[3,2-*b*]azepine-1(2*H*)-carboxylate ((*R,R*)-**57**)

To a solution of orthogonally protected (*R,R*)*-***35** (90.0 mg, 0.27 mmol, 1.0 equiv) in MeOH (2.7 mL, 0.1 M) was added Pd/C (10%, Pd on activated charcoal, 10 mg, 10 wt % of substrate). The mixture was stirred at 22 °C under an atmosphere of hydrogen (1 atm, balloon) for 24 h. Then it was filtered over Celite and evaporated to dryness to yield a colorless oil. To a solution of intermediate product in MeOH (2.5 mL, 0.1 M) was added 2-chlorobenzaldehyde (58 mg, 0.41 mmol, 1.5 equiv), NaBH_3_CN (26 mg, 0.41 mmol, 1.5 equiv), and AcOH (23 μL, 0.38 mmol, 1.5 equiv). The mixture was refluxed for 24 h. After cooling to room temperature, it was quenched by the addition of NaOH (1 M, 4 mL). Then, the solvent was concentrated under reduced pressure, and the aqueous phase was extracted with EtOAc (3 × 20 mL). The organic layers were dried over Na_2_SO_4_ and filtered, and the solvent was removed in vacuo. The crude product was purified by flash chromatography on silica gel (10% EtOAc in heptane) to afford derivatized diamine (*R,R*)-**57** (56 mg, 0.15 mmol, 56%) as a colorless oil. R_f_ = 0.22 (5% EtOAc in heptane); ^1^H NMR (400 MHz, MeOD-*d*_4_, 298 K): δ [ppm] = 7.62–7.64 (m, 1H; H-*C*_arom._), 7.33–7.36 (m, 1H; H-*C*_arom._), 7.26–7.30 (m, 1H; H-*C*_arom._), 7.19–7.23 (m, 1H; H-*C*_arom._), 3.90 (d, *J* = 14.6 Hz, 1H; H-*C*_benz._), 3.80–3.88 (m, 1H; H-*C*(8a)), 3.66 (d, *J* = 14.6, 1H; H-*C*_benz._), 3.41–3.47 (m, 1H; H-*C* (3)), 3.22–3.26 (m, 2H; H-*C*(3 and 3a)), 2.44–2.58 (m, 2H; H-*C* (5)), 2.21–2.24 (m, 1H; H-*C* (2)), 1.93–2.05 (m, 2H; H-*C* (2 and 8)), 1.62–1.75 (m, 2H; H-*C* (7 and 8)), 1.51–1.55 (m, 1H; H-*C* (6)), 1.47 (s, 9H; H_3_-*C*_Boc_), 1.35–1.44 (m, 2H; H-*C* (6 and 7)); ^13^C NMR (100 MHz, MeOD-*d*_4_, 298 K): δ [ppm] = 156.2 ((*C*=O)_Boc_, 138.7 ((C_q_)_arom._), 135.0 ((C_q_)_arom._), 132.2 ((*C*H)_arom._), 130.4 ((*C*H)_arom._), 129.2 ((*C*H)_arom._), 127.7 ((*C*H)_arom._), 80.8 ((C_q_)_Boc_), 68.5 (C(3a))_rot._, 68.0 (C(3a))_rot._, 64.1 (C(8a))_rot._, 63.7 (C(8a))_rot._, 58.2 (*C*H_2_)_benz._), 53.8 (C (5)), 45.5 (C (3))_rot._, 44.9 (C (3))_rot._, 32.3 (C (6)), 31.1 (C (2 or 8))_rot._, 30.9 (C (2 or 8))_rot._, 30.4 (C (2 or 8))_rot._, 30.1 (C (2 or 8))_rot._, 28.8 ((*C*H_3_)_Boc_), 28.0 (C (7))_rot._, 27.9 (C (7))_rot._; HR-MS (ESI): (*m*/*z*) = calculated for C_20_H_30_N_2_O_2_Cl^+^ [*M*+H]^+^: 365.1990, found: 365.1984.

#### (3a*R*,8a*R*)-4-(2-Chlorobenzyl)decahydropyrrolo[3,2-*b*]azepine ((*R,R*)-**60**)

To a solution of Boc-protected amine (*R,R*)-**57** (46 mg, 0.13 mmol, 1.0 equiv) in DCM (1.3 mL) was added TFA (0.13 mL, 10 vol %). The reaction was stirred at 22 °C for 2 h. Then, the solvent was evaporated, and the crude was purified by flash chromatography on silica gel (5%–10% MeOH in DCM + 1% NH_3_) to afford derivatized diamine (*R,R*)-**60** (16.2 mg, 0.06 mmol, 46%) as an orange oil. R_f_ = 0.20 (10% MeOH in DCM + 1% NH_3_); ^1^H NMR (400 MHz, MeOD-*d*_4_, 298 K): δ [ppm] = 7.60–7.62 (m, 1H; H-*C*_arom._), 7.33–7.35 (dd, *J* = 7.8, 1.3 Hz, 1H; H-*C*_arom._), 7.26–7.30 (m, 1H; H-*C*_arom._), 7.19–7.23 (m, 1H; H-*C*_arom._), 3.89 (d, *J* = 14.6 Hz, 1H; H-*C*_benz._), 3.67 (d, *J* = 14.6 Hz, 1H; H-*C*_benz._), 3.33–3.40 (m, 2H; H-*C*(3a and 8a)), 3.14–3.19 (m, 1H; H-*C* (2)), 2.85–2.92 (m, 1H; H-*C* (2)), 2.64–2.69 (m, 1H; H-*C* (5)), 2.37–2.43 (m, 1H; H-*C* (5)), 2.17–2.24 (m, 1H; H-*C* (3)), 2.05–2.14 (m, 1H; H-*C* (8)), 1.84–1.92 (m, 1H; H-*C* (3)), 1.76–1.82 (m, 1H; H-*C* (6)), 1.65–1.70 (m, 1H; H-*C* (7)), 1.35–1.56 (m, 3H; H-*C* (6, 7 and 8)); ^13^C NMR (100 MHz, MeOD-*d*_4_, 298 K): δ [ppm] = 138.8 ((C_q_)_arom._), 134.9 ((C_q_)_arom._), 132.0 ((*C*H)_arom._), 130.4 ((*C*H)_arom._), 129.3 ((*C*H)_arom._), 127.8 ((*C*H)_arom._), 69.2 (C(3a or 8a)), 64.5 (C(3a or 8a)), 58.0 (*C*H_2_)_benz._)), 54.9 (C (5)), 45.1 (C (2)), 32.4 (C (3)), 31.6 (C (7 or 8)), 31.4 (C (7 or 8)), 27.2 (C (6)); HR-MS (ESI): (*m*/*z*) = calculated for C_15_H_22_N_2_Cl^+^ [*M*+H]^+^: 265.1466, found: 265.1472.

#### (3a*R*,8a*R*)-4-(3-Bromobenzyl)decahydropyrrolo[3,2-*b*]azepine ((*R,R*)-**61**)

To a solution of mono-Boc protected amine (*R,R*)-**11** (16.0 mg, 0.07 mmol, 1.0 equiv) in MeOH (1 mL, 0.1 M) was added 3-bromobenzaldehyde (13 μL, 0.11 mmol, 1.5 equiv), NaBH_3_CN (6.9 mg, 0.11 mmol, 1.5 equiv), and AcOH (6 μL, 0.11 mmol, 1.5 equiv). The mixture was refluxed for 24 h. After cooling to room temperature, it was quenched by the addition of NaOH (1 M, 1 mL). Then, the solvent was concentrated under reduced pressure, and the aqueous phase was extracted with EtOAc (3 × 10 mL). The organic layers were dried over Na_2_SO_4_, filtered and and the solvent was removed in vacuo. The intermediate product was dissolved in DCM (1 mL), and TFA (0.1 mL, 10 vol %) was added. The reaction was stirred at 22 °C for 2 h. Then, the solvent was evaporated, and the crude was purified by flash chromatography on silica gel (5%–10% MeOH in DCM + 1% NH_3_) and treated with TFA to afford derivatized diamine (*R,R*)-**61** as double TFA salt (11.2 mg, 0.02 mmol, 29%) and yellowish oil. R_f_ = 0.20 (5% MeOH in DCM + 1% NH_3_); ^1^H NMR (400 MHz, MeOD-*d*_4_, 298 K): δ [ppm] = 7.57 (s, 1H; H-*C*_arom._), 7.40 (m, 1H; H-*C*_arom._), 7.34 (m, 1H; H-*C*_arom._), 7.24 (t, *J* = 7.8 Hz, 1H; H-*C*_arom._), 3.93 (d, *J* = 14.1 Hz, 1H; H-*C*_benz._), 3.71–3.77 (m, 1H; H-*C*(8a)), 3.40–3.51 (m, 3H; H-*C*(3a, 5 and H-*C*_benz._)), 3.15–3.22 (m, 1H; H-*C* (5)), 2.71–2.77 (m, 1H; H-*C* (3)), 2.36–2.44 (m, 2H; H-*C* (3 and 6)), 2.17–2.27 (m, 1H; H-*C* (8)), 1.97–2.05 (m, 1H; H-*C* (6)), 1.79–1.88 (m, 2H; H-*C* (7 and 8)), 1.57–1.61 (m, 1H; H-*C* (2)), 1.42–1.52 (m, 2H; H-*C* (2 and 7)); ^13^C NMR (100 MHz, MeOD-*d*_4_, 298 K): δ [ppm] = 143.8 ((C_q_)_arom._), 132.6 ((*C*H)_arom._), 2X 131.2 ((2X *C*H)_arom._), 128.5 ((*C*H)_arom._), 123.4 ((C_q_)_arom._), 66.9 (C(3a)), 64.5 (C(8a)), 60.1 (*C*H_2_)_benz._)), 54.7 (C (3)), 44.5 (C (5)), 31.3 (C (2 or 6)), 31.1 (C (2 or 6)), 29.4 (C (8)), 26.8 (C (7)); ^19^F-NMR (100 MHz, MeOD-*d*_4_, 298 K): δ [ppm] = −77.0; HR-MS (ESI): (*m*/*z*) = calculated for C_15_H_22_N_2_Br^+^ [*M*+H]^+^: 309.0961, found: 309.0959.

#### (3a*R*,8a*R*)-4-(2,3-Dichlorobenzyl)decahydropyrrolo[3,2-*b*]azepine ((*R,R*)-**62**)

To a solution of mono-Boc protected amine (*R,R*)-**11** (16.0 mg, 0.07 mmol, 1.0 equiv) in MeOH (1 mL, 0.1 M) was added 2,3-dichlorobenzaldehyde (19 mg, 0.11 mmol, 1.5 equiv), NaBH_3_CN (6.9 mg, 0.11 mmol, 1.5 equiv), and AcOH (6 μL, 0.11 mmol, 1.5 equiv). The mixture was refluxed for 24 h. After cooling to room temperature, it was quenched by the addition of NaOH (1 M, 1 mL). Then, the solvent was concentrated under reduced pressure, and the aqueous phase was extracted with EtOAc (3 × 10 mL). The organic layers were dried over Na_2_SO_4_ and filtered, and the solvent was removed in vacuo. The intermediate product was dissolved in DCM (1 mL), and TFA (0.1 mL, 10 vol %) was added. The reaction was stirred at 22 °C for 2 h. Then, the solvent was evaporated, and the crude was purified by flash chromatography on silica gel (5%–10% MeOH in DCM + 1% NH_3_) and treated with TFA to afford derivatized diamine (*R,R*)-**62** as double TFA salt (9.3 mg, 0.02 mmol, 29%) and yellowish oil. R_f_ = 0.20 (5% MeOH in DCM + 1% NH_3_); ^1^H NMR (400 MHz, MeOD-*d*_4_, 298 K): δ [ppm] = 7.36–7.37 (m, 2H; H-*C*_arom._), 7.32–7.33 (m, 1H; H-*C*_arom._), 3.91 (d, *J* = 14.4 Hz, 1H; H-*C*_benz._), 3.73–3.79 (m, 1H; H-*C*(8a)), 3.41–3.53 (m, 3H; H-*C*(2, 3a and H-*C*_benz._)), 3.16–3.23 (m, 1H; H-*C* (2)), 2.69–2.74 (m, 1H; H-*C* (5)), 2.37–2.47 (m, 2H; H-*C* (3 and 5)), 2.17–2.26 (m, 1H; H-*C* (8)), 1.96–2.05 (m, 1H; H-*C* (3), 1.81–1.90 (m, 2H; H-*C* (7 and 8)), 1.58–1.62 (m, 1H; H-*C* (6)), 1.44–1.53 (m, 2H; H-*C* (6 and 7)); ^13^C NMR (100 MHz, MeOD-*d*_4_, 298 K): δ [ppm] = 145.4 ((C_q_)_arom._), 2X 136.1 ((C_q_)_arom._), 3X 128.1 ((3X *C*H)_arom._), 66.8 (C(3a)), 64.4 (C(8a)), 59.6 (*C*H_2_)_benz._)), 55.0 (C (5)), 44.5 (C (2)), 31.3 (C (3 or 6)), 31.0 (C (3 or 6)), 29.3 (C (8)), 26.7 (C (7)); ^19^F-NMR (100 MHz, MeOD-*d*_4_, 298 K): δ [ppm] = −77.0; HR-MS (ESI): (*m*/*z*) = calculated for C_15_H_21_N_2_Cl_2_^+^ [*M*+H]^+^: 299.1075, found: 299.1076.

#### (3a*R*,8a*R*)-4-(3,5-Dichlorobenzyl)decahydropyrrolo[3,2-*b*]azepine ((*R,R*)-**63**)

To a solution of mono-Boc protected amine (*R,R*)-**11** (16.0 mg, 0.07 mmol, 1.0 equiv) in MeOH (1 mL, 0.1 M) was added 3,5-dichlorobenzaldehyde (19 mg, 0.11 mmol, 1.5 equiv), NaBH_3_CN (6.9 mg, 0.11 mmol, 1.5 equiv), and AcOH (6 μL, 0.11 mmol, 1.5 equiv). The mixture was refluxed for 24 h. After cooling to room temperature, it was quenched by the addition of NaOH (1 M, 1 mL). Then, the solvent was concentrated under reduced pressure, and the aqueous phase was extracted with EtOAc (3 × 10 mL). The organic layers were dried over Na_2_SO_4_ and filtered, and the solvent was removed in vacuo. The intermediate product was dissolved in DCM (1 mL), and TFA (0.1 mL, 10 vol %) was added. The reaction was stirred at 22 °C for 2 h. Then, the solvent was evaporated, and the crude was purified by flash chromatography on silica gel (5%–10% MeOH in DCM + 1% NH_3_) and treated with TFA to afford derivatized diamine (*R,R*)-**63** as double TFA salt (9.0 mg, 0.02 mmol, 29%) and yellowish oil. R_f_ = 0.20 (5% MeOH in DCM + 1% NH_3_); ^1^H NMR (400 MHz, MeOD-*d*_4_, 298 K): δ [ppm] = 7.54–7.57 (m, 1H; H-*C*_arom._), 7.44–7.46 (m, 1H; H-*C*_arom._), 7.29 (t, *J* = 7.9 Hz, 1H; H-*C*_arom._), 3.96 (d, *J* = 14.7 Hz, 1H; H-*C*_benz._), 3.73–3.80 (m, 2H; H-*C*_benz._ and H-*C*(8a)), 3.55–3.61 (m, 1H; H-*C*(3a)), 3.41–3.46 (m, 1H; H-*C* (2)), 3.15–3.23 (m, 1H; H-*C* (2)), 2.70–2.75 (m, 1H; H-*C* (5)), 2.45–2.52 (m, 1H; H-*C* (5)), 2.37–2.42 (m, 1H; H-*C* (3)), 2.18–2.24 (m, 1H; H-*C* (8)), 2.03–2.10 (m, 1H; H-*C* (3)), 1.83–1.88 (m, 2H; H-*C* (7 and 8)), 1.57–1.60 (m, 1H; H-*C* (6)), 1.44–1.50 (m, 2H; H-*C* (6 and 7)); ^13^C NMR (100 MHz, MeOD-*d*_4_, 298 K): δ [ppm] = 141.0 ((C_q_)_arom._), 134.1 ((C_q_)_arom._), 133.0 ((C_q_)_arom._), 2X 130.2 ((*C*H)_arom._), 128.5 ((*C*H)_arom._), 67.1 (C(3a)), 64.4 (C(8a)), 58.3 (*C*H_2_)_benz._)), 54.8 (C (5)), 44.5 (C (2)), 31.0 (C (3 or 6)), 30.6 (C (3 or 6)), 29.2 (C (8), 26.6 (C (7)); ^19^F-NMR (100 MHz, MeOD-*d*_4_, 298 K): δ [ppm] = −77.0; HR-MS (ESI): (*m*/*z*) = calculated for C_15_H_21_N_2_Cl_2_^+^ [*M*+H]^+^: 299.1076, found: 299.1074.

### Purity of Compounds

All compounds are >95% pure by HPLC analysis (Table S1). Analytical RP-HPLC was performed with an Ultimate 3000 Rapid Separation LC–MS System (DAD-3000RS diode array detector) using an Acclaim RSLC 120 C18 column (2.2 μm, 120 Å, 3 × 50 mm, flow 1.2 mL/min) from Dionex. Data recording and processing was done with Dionex Chromeleon Management System Version 6.80 (analytical RP-HPLC). All RP-HPLC were using HPLC-grade acetonitrile and Milli-Q deionized water. The elution solutions were A) Milli-Q deionized water containing 0.05% TFA; D) Milli-Q deionized water/acetonitrile (10:90, v/v) containing 0.05% TFA.

### Safety Statement

With this statement we confirm that no unexpected or unusually high safety hazards were encountered.

### Biological Assays and Pharmacological Experiments

#### Monoamine Uptake and Secretion Assays in Cultured PC12 Rat Adrenal Gland (Phaeochromocytoma) Cells

##### Uptake Assay

PC12 (CRL-1721) cells were cultured in Dulbecco’s modified Eagle medium (DMEM) complete, supplemented with 10% fetal bovine serum (FBS). Cells were seeded in a poly-d-lysine coated 24-well plate at a density of 200,000 cells per well and placed in the cell incubator at 5% CO_2_ and 37 °C for 48 h to achieve full confluence. For the uptake assay, the 24-well plate was maintained in the cell incubator. An uptake assay buffer was prepared as follows: 130 mM NaCl, 1.5 mM KCl, 1.25 mM CaCl_2_, 2 mM NaH_2_PO_4_, 1.5 mM MgCl_2_, 20 mM glucose, 25 mM HEPES, 0.1 mM EDTA, and 0.5% BSA, pH 7.2. Cells were preincubated with either dimethyl sulfoxide (DMSO), atomoxetine (Merck, Germany), venlafaxine and amoxapine (both Medchem Express, USA distributed by Lucerna Chem AG, Switzerland), (*R,R*)-**1a** and (*S,S*)-**1a**, for 30 min. Then, the cells were incubated for 2 h with the same treatments, as well as a monoamine mix containing 1 μM of each of the following monoamines: norepinephrine, dopamine, and serotonin. The assay buffer on the cells was collected and snap-frozen to be later analyzed with LC-ESI-MS/MS. *Secretion assay:* The cells were seeded in a poly-d-lysine coated poly-d-lysine coated 24-well plate at a density of 200,000 cells per well and incubated in 5% CO_2_ at 37 °C for 24 h to achieve near-full confluence. Then cells were incubated with DMEM complete with 10% Fetal Bovine Serum (FBS), containing either DMSO or 10 μM (*R,R*)-**1a**, for 24 h. For the secretion assay, PC12 cells were maintained at a constant temperature of 37 °C using a thermomixer. Cells were stimulated with basal secretion buffer consisting of 110 mM NaCl, 4.7 mM KCl, 2.5 mM CaCl_2_, 1.2 mM NaH_2_PO_4_, 1.2 mM MgCl_2_, 11 mM glucose, and 15 mM Hepes, pH 7.4, for 15 min. A high K^+^ buffer consisting of 85 mM NaCl, 59 mM KCl, 2.5 mM CaCl_2_, 1.2 mM NaH_2_PO_4_, 1.2 mM MgCl_2,_ 11 mM glucose, and 15 mM Hepes, pH 7.4 to induce secretion. After 10 min of incubation buffer was collected and snap frozen to be later analyzed with LC-ESI-MS/MS.

#### LC-ESI-MS/MS for Neurotransmitter Quantification

The targeted MRM-based method for the quantification of neurotransmitters was performed as described before.^79^ For the analysis, a hybrid triple quadrupole 4000 QTRAP mass spectrometer (AB Sciex Concord, ON, Canada) with a Shimadzu UFLC (Shimadzu Corporation, Kyoto, Japan) and with a cooled autosampler was used. The sample temperature was maintained at 4 °C in the autosampler prior to analysis. The LC column used was Imtakt Intrada amino acid WAA34, 100 × 3 mm; 3 μm, maintained at 35 °C. The system was operated in positive mode. The mobile phases were 100 mM ammonium formate in water (mobile phase A) and 95:5:0.3 acetonitrile:water:formic acid (mobile phase B). The gradient at a flow rate of 1.00 mL/min was as follows: 0 min—92% B, 3 min—88% B, 6.4 min—70% B, 6.5–10 min—0% B, 10.1–12.9 min—92% B. Chromatograms were generated utilizing the fixed retention time configuration. MS peaks were quantified using a calibration curve. All chemicals and reagents used for LC-ESI-MS/MS were obtained from Sigma-Aldrich and were of the purest analytical HPLC grade.

##### MRM LC-ESI-MS/MS Method to Quantify (*R,R*)-**1a**

A hybrid triple quadrupole 4000 QTRAP mass spectrometer (AB Sciex Concord, Ontario, Canada) was used with a Shimadzu UFLC (Shimadzu Corporation, Kyoto, Japan) with a cooled autosampler. The system was used in a negative-ion mode and a Turbo ion-spray with gas1, gas2, and curtain gas pressures set at 50, 50, and 40 psi, respectively. The source was heated at 650 °C. Quantitation was performed with MRM (multiple reaction-monitoring) mode. MRM conditions were optimized for all analytes and internal standards (IS). As IS (*R,R*)-**60** (2-chloro) C_15_H_21_ClN_2_·2HCl MW: 337.72, MW 264.14 (base) was used.

Mass spectrometry parameters were optimized by direct infusion. The sample temperature was maintained at 4 °C in the autosampler prior to analysis. The LC column was a Waters X-Select HSS T3 2.5 μm C18 2.1 × 100 mm, maintained at 60 °C. The mobile phases were 5 mM ammonium formate in water +0.1%FA, (mobile phase A) and 5 mM ammonium formate in methanol +0.1%FA (mobile phase B). The flow rate is 0.6 mL/min with the gradient indicated in Table S3. Peaks were integrated, and the Analyst software version 1.6.2 (AB Sciex Concord, Ontario, Canada) was used for quantification. Identification of compounds in samples was confirmed by comparison of precursor and product ion *m*/*z* values and LC retention times with standards. The multiple reaction monitoring (MRM) transitions in Table S4 were monitored for quantification of the analytes. The calibration curve for (*R,R*)-**1a** is shown in Figure S15.

#### Mice Experiments

The animal study was conducted in compliance with the regulations of the Swiss authorities. The study protocol was reviewed and approved by the Veterinäramt Kanton Bern (approval number BE70/2024), ensuring adherence to ethical guidelines for animal research.

#### Evaluation of Pharmacokinetic Profiles and Biodistribution

C57BL/6JRJ mice (6 weeks old, 2 males and 2 females) were supplied by Janvier Laboratories and kept under standard environmental conditions (24 ± 2 °C; light–dark cycle of 12:12 h) with food and water ad libitum. Mice were allowed to acclimate to the animal facility for 7 days. Then, mice were habituated to handling for 7 days, which was done by taking them out of the cage using a tunnel and placing them in the hands of the experimenter for a few seconds before returning to the cage. For the pharmacokinetic profiles, blood microsamples (<20 μL) were taken from the tail-veil at the following time points after administration: 5 min, 15 min, 30 min, 1 h, 1.5 h, 2 h, 4 h, 8 h, and 24 h. Blood samples were taken by placing each mouse on the grip of a cage (not the home-cage) and covering them on top using a small cup (7 × 8 × 8 cm) with a cut-out semicircle (1 × 1.5 cm) to allow easy access to the tail of the mouse. Then, the tail was swabbed with a cotton pad impregnated with 70% ethanol for disinfection, a puncture was done using a needle (24G), and after applying a slight pressure, the blood was collected using 20 μL end-to-end-capillaries with EDTA. The needle puncture was only necessary for the first and the 24 h samples.

(*R,R*)-**1a** dose was calculated considering the free salt molecular weight and formulated in 0.9% NaCl (saline) using a 5 mL/kg administration volume. The pharmacokinetic profile was evaluated first using intravenous administration. For this, the mouse’s tail was warmed up for 1–2 min in a water bath at 37–38 °C, and the administration was performed on a tail vein using a fine needle (27 or 30G). After 1 week of washout, the pharmacokinetic profile was evaluated after oral administration using a feeding probe (20G, 30 mm).

After 1 week of wash-out, tissue biodistribution was evaluated after oral administration. For this, mice were administered orally using a feeding probe (20G, 30 mm), and euthanasia was carried out by decapitation 30 min after administration. Before euthanasia, anesthesia was induced by placing each mouse in an airtight induction box and delivering isoflurane at 0.3–0.6 L/min (4%) for 3 min; 100% oxygen was used as carrier gas to prevent hypoxia. After decapitation, blood was collected from the trunk using EDTA tubes, an aliquot of whole blood was taken, and the rest was spun down to separate the plasma. Brain and peripheral tissues were harvested, immediately snap-frozen with liquid nitrogen, and stored at −80 °C until processing. Biodistribution was evaluated 30 min after administration because *C*_max_ values on blood were similar between 15 and 30 min after oral administration.

#### Behavioral Mouse Experiments

Adult male and female C57Bl6/J mice (8–12 weeks old; 15–18 g body weight) were supplied by Janvier Laboratories and kept under standard environmental conditions (24 ± 2 °C; light–dark cycle of 12:12 h) with food and water ad libitum. Fresh bedding and environmental enrichment were provided weekly. Following 1 week of acclimatization at the local experimental facility, mice were handled daily for 10 days by the same experimenter (2 min sessions/mouse). In the last 2 days of handling, animals were trained in the Rotarod task. In these training sessions, each mouse was examined for a maximum of 5 min, or until managing to stay on an operating rotarod, set at 4 rpm for 2 min.^80^ All animals examined in the acute and chronic cohorts completed the Rotarod training successfully.

#### Acutely Treated Cohort

Following handling and training, 12 week-old male mice in the acutely treated cohort, were administered either a dose of the compound (*n* = 7), or vehicle (0.5% DMSO, *n* = 6) intraperitoneally, in a 1% body weight volume. Animals were treated in two sessions, separated by a week of rest (no treatment or behavioral testing). During the treatment session, injected animals were placed in a separate cage for 40 min. Immediately thereafter, animals were examined in the Rotarod task (approximately 5 min in total) using the PanLab 76–0770 (Harvard Apparatus). In animals that failed to complete the task, the mean latency of three consecutive attempts carried out over the 5 min was calculated. Following 5 min of rest, mice were placed in the light/dark box arena, illuminated at 65/0 lx,^81^ where activity was monitored using an appropriate high-speed infrared camera and video analysis software (EthoVision 13).

Activity was recorded over 5 min, and analysis was conducted for individual parameters in the respective compartments of the arena, as previously described.^81^ Following an additional 5 min of rest, body temperature was recorded using a rectal probe. At the end of the second 20 min behavioral session, animals were deeply anesthetized and euthanized using saline reperfusion, 1 h following drug administration. Brain tissue was extracted, and individual brain regions were dissected in ice-cold saline. The tissue was briefly dried and snap-frozen using liquid nitrogen. Midbrain and brainstem tissue were weighted and processed for LC-ESI-MS/MS analysis as previously described.^82^

#### Chronically Treated Mouse Cohort

A separate cohort of 8 week-old male (*n* = 10) and female (*n* = 10) mice was used to study the effects of chronic drug administration on behavior. Following handling (10 days) and rotarod training (2 days), the cohort was administered either a 0.5 mg/kg dose of the compound, or vehicle (5 females and 5 females/group), daily, for 5 consecutive days of each week, over 4 weeks (28 days). Throughout the duration of the study, mice were provided with their regular light/dark cycle, food and water ad libitum, and weekly fresh cages. Weight was monitored every 2 days.

Core body temperature was examined on the fifth day of each study week using a rectal probe. Prior to behavioral examination, injected animals were briefly transferred to a fresh cage (40 min). Body temperature was examined weekly. On days 17 and 19, animals in the treatment group received a 1.0 mg/kg dose instead of their daily dose. Similarly, on days 24 and 26, animals in the treatment group received a 10 mg/kg dose. In separate sessions, animals were examined in the Rotarod and light/dark box paradigms, as well as the tail suspension test (TST).^83^

The time spent immobile was recorded over a single 2 min trial by an additional experimenter that was blind to the treatment group. Mice were examined using the rotarod test on days 3, 10, 17, and 25. The light/dark box test was conducted on days 9 and 24. TSTs were carried out on days 5, 19, and 26. Core body temperature was additionally examined prior to the TSTs on days 19 and 26. At the start of the fifth week (following 48 h of rest), mice were administered a 0.5 mg/kg dose of the compound, or vehicle, prior to tissue collection. Mice were sacrificed under deep isoflurane anesthesia and cardiac reperfusion, as above. All sacrifices and tissue collection were similarly performed 1 h following drug administration.

## Supporting Information Available

24 monocyclic and bicyclic ring systems consisting of 5-, 6-, or 7-membered rings extracted from GDB-4c (Figure S1); supporting synthesis schemes (Schemes S1–S3), general methods for chemical synthesis; ^1^H/ ^13^C NMR spectra, chiral HPLC, and analytical purity (Table S1); X-ray crystal deposition of representative compounds (Figure S2); PPB2 target prediction for fused azepanes (Table S2 and Figures S3–S10); biochemical assay procedures and details of biological assays (Figures S11–S14); and related analytical methods (Tables S3 and S4 and Figure S15) (PDF)
Molecular string files of all compounds (CSV)

## References

[ref1] Bohacek R. S.; McMartin C.; Guida W. C. The Art and Practice of Structure-Based Drug Design: A Molecular Modeling Perspective. Med. Res. Rev. 1996, 16 (1), 3–50. 10.1002/(SICI)1098-1128(199601)16:1<3::AID-MED1>3.0.CO;2-6. 8788213

[ref2] Reymond J.; Ruddigkeit L.; Blum L.; van Deursen R. The Enumeration of Chemical Space. WIREs Comput. Mol. Sci. 2012, 2 (5), 717–733. 10.1002/wcms.1104.

[ref3] Mullard A. The Drug-Maker’s Guide to the Galaxy. Nature 2017, 549 (7673), 445–447. 10.1038/549445a. 28959982

[ref4] Hoffmann T.; Gastreich M. The next Level in Chemical Space Navigation: Going Far beyond Enumerable Compound Libraries. Drug Discovery Today 2019, 24 (5), 1148–1156. 10.1016/j.drudis.2019.02.013. 30851414

[ref5] Warr W. A.; Nicklaus M. C.; Nicolaou C. A.; Rarey M. Exploration of Ultralarge Compound Collections for Drug Discovery. J. Chem. Inf. Model. 2022, 62 (9), 2021–2034. 10.1021/acs.jcim.2c00224. 35421301

[ref6] Grygorenko O. O.; Radchenko D. S.; Dziuba I.; Chuprina A.; Gubina K. E.; Moroz Y. S. Generating Multibillion Chemical Space of Readily Accessible Screening Compounds. iScience 2020, 23 (11), 101681 10.1016/j.isci.2020.101681. 33145486 PMC7593547

[ref7] Sadybekov A. V.; Katritch V. Computational Approaches Streamlining Drug Discovery. Nature 2023, 616 (7958), 673–685. 10.1038/s41586-023-05905-z. 37100941

[ref8] Dickson P.; Kodadek T. Chemical Composition of DNA-Encoded Libraries, Past Present and Future. Organic & Biomolecular Chemistry 2019, 17 (19), 4676–4688. 10.1039/C9OB00581A. 31017595 PMC6520149

[ref9] Gironda-Martínez A.; Donckele E. J.; Samain F.; Neri D. DNA-Encoded Chemical Libraries: A Comprehensive Review with Succesful Stories and Future Challenges. ACS Pharmacol. Transl. Sci. 2021, 4 (4), 1265–1279. 10.1021/acsptsci.1c00118. 34423264 PMC8369695

[ref10] Peterson A. A.; Liu D. R. Small-Molecule Discovery through DNA-Encoded Libraries. Nat. Rev. Drug Discovery 2023, 22, 699–722. 10.1038/s41573-023-00713-6. 37328653 PMC10924799

[ref11] Dockerill M.; Winssinger N. DNA-Encoded Libraries: Towards Harnessing Their Full Power with Darwinian Evolution. Angew. Chem. 2023, 135 (9), e202215542 10.1002/ange.202215542. 36458812

[ref12] Lipinski C. A.; Lombardo F.; Dominy B. W.; Feeney P. J. Experimental and Computational Approaches to Estimate Solubility and Permeability in Drug Discovery and Development Settings. Adv. Drug Delivery Rev. 1997, 23 (1), 3–25. 10.1016/S0169-409X(96)00423-1. 11259830

[ref13] Fink T.; Bruggesser H.; Reymond J. L. Virtual Exploration of the Small-Molecule Chemical Universe below 160 Da. Angew. Chem., Int. Ed. Engl. 2005, 44 (10), 1504–1508. 10.1002/anie.200462457. 15674983

[ref14] Fink T.; Reymond J. L. Virtual Exploration of the Chemical Universe up to 11 Atoms of C, N, O, F: Assembly of 26.4 Million Structures (110.9 Million Stereoisomers) and Analysis for New Ring Systems, Stereochemistry, Physicochemical Properties, Compound Classes, and Drug Discovery. J. Chem. Inf. Model. 2007, 47 (2), 342–353. 10.1021/ci600423u. 17260980

[ref15] Blum L. C.; Reymond J.-L. 970 Million Druglike Small Molecules for Virtual Screening in the Chemical Universe Database GDB-13. J. Am. Chem. Soc. 2009, 131 (25), 8732–8733. 10.1021/ja902302h. 19505099

[ref16] Ruddigkeit L.; van Deursen R.; Blum L. C.; Reymond J. L. Enumeration of 166 Billion Organic Small Molecules in the Chemical Universe Database GDB-17. J. Chem. Inf. Model. 2012, 52 (11), 2864–2875. 10.1021/ci300415d. 23088335

[ref17] Reymond J.-L.; Ruddigkeit L.; Blum L.; van Deursen R. The Enumeration of Chemical Space. WIREs Computational Molecular Science 2012, 2 (5), 717–733. 10.1002/wcms.1104.

[ref18] Meier K.; Bühlmann S.; Arús-Pous J.; Reymond J.-L. The Generated Databases (GDBs) as a Source of 3D-Shaped Building Blocks for Use in Medicinal Chemistry and Drug Discovery. CHIMIA 2020, 74 (4), 241–241. 10.2533/chimia.2020.241. 32331540

[ref19] Buehler Y.; Reymond J.-L. Molecular Framework Analysis of the Generated Database GDB-13s. J. Chem. Inf. Model. 2023, 63 (2), 484–492. 10.1021/acs.jcim.2c01107. 36533982 PMC9875802

[ref20] Buehler Y.; Reymond J.-L. Expanding Bioactive Fragment Space with the Generated Database GDB-13s. J. Chem. Inf. Model. 2023, 63, 6239 10.1021/acs.jcim.3c01096. 37722101 PMC10598793

[ref21] Rankovic Z. CNS Drug Design: Balancing Physicochemical Properties for Optimal Brain Exposure. J. Med. Chem. 2015, 58 (6), 2584–2608. 10.1021/jm501535r. 25494650

[ref22] Meier K.; Arús-Pous J.; Reymond J.-L. A Potent and Selective Janus Kinase Inhibitor with a Chiral 3D-Shaped Triquinazine Ring System from Chemical Space. Angew. Chem., Int. Ed. Engl. 2021, 60 (4), 2074–2077. 10.1002/anie.202012049. 32986914

[ref23] Bizzini L. D.; Müntener T.; Häussinger D.; Neuburger M.; Mayor M. Synthesis of Trinorbornane. Chem. Commun. 2017, 53 (83), 11399–11402. 10.1039/C7CC06273G. 28975933

[ref24] Delarue Bizzini L.; Bürgi T.; Mayor M. The Enantiomers of Trinorbornane and Derivatives Thereof. Helv. Chim. Acta 2020, 103 (3), e2000019 10.1002/hlca.202000019.

[ref25] Rebhan L.; Buehler Y.; Reymond J.-L. Diamonds in Chemical Space: The Synthesis of Brexazine. Helv. Chim. Acta 2025, 108 (1), e202400175 10.1002/hlca.202400175.

[ref26] Carreira E. M.; Fessard T. C. Four-Membered Ring-Containing Spirocycles: Synthetic Strategies and Opportunities. Chem. Rev. 2014, 114 (16), 8257–8322. 10.1021/cr500127b. 25003801

[ref27] Shire B. R.; Anderson E. A. Conquering the Synthesis and Functionalization of Bicyclo[1.1.1]Pentanes. JACS Au 2023, 3 (6), 1539–1553. 10.1021/jacsau.3c00014. 37388694 PMC10301682

[ref28] Kirichok A. A.; Tkachuk H.; Kozyriev Y.; Shablykin O.; Datsenko O.; Granat D.; Yegorova T.; Bas Y. P.; Semirenko V.; Pishel I.; Kubyshkin V.; Lesyk D.; Klymenko-Ulianov O.; Mykhailiuk P. K. 1-Azaspiro[3.3]Heptane as a Bioisostere of Piperidine**. Angew. Chem., Int. Ed. Engl. 2023, 62 (51), e202311583 10.1002/anie.202311583. 37819253

[ref29] Levterov V. V.; Panasiuk Y.; Sahun K.; Stashkevych O.; Badlo V.; Shablykin O.; Sadkova I.; Bortnichuk L.; Klymenko-Ulianov O.; Holota Y.; Lachmann L.; Borysko P.; Horbatok K.; Bodenchuk I.; Bas Y.; Dudenko D.; Mykhailiuk P. K. 2-Oxabicyclo[2.2.2]Octane as a New Bioisostere of the Phenyl Ring. Nat. Commun. 2023, 14 (1), 5608 10.1038/s41467-023-41298-3. 37783681 PMC10545790

[ref30] Dibchak D.; Snisarenko M.; Mishuk A.; Shablykin O.; Bortnichuk L.; Klymenko-Ulianov O.; Kheylik Y.; Sadkova I. V.; Rzepa H. S.; Mykhailiuk P. K. General Synthesis of 3-Azabicyclo[3.1.1]Heptanes and Evaluation of Their Properties as Saturated Isosteres**. Angew. Chem., Int. Ed. Engl. 2023, 62 (39), e202304246 10.1002/anie.202304246. 37232421

[ref31] Tsien J.; Hu C.; Merchant R. R.; Qin T. Three-Dimensional Saturated C(Sp3)-Rich Bioisosteres for Benzene. Nat. Rev. Chem. 2024, 8, 605–627. 10.1038/s41570-024-00623-0. 38982260 PMC11823177

[ref32] Schreiber S. L. Target-Oriented and Diversity-Oriented Organic Synthesis in Drug Discovery. Science 2000, 287 (5460), 1964–1969. 10.1126/science.287.5460.1964. 10720315

[ref33] Kidd S. L.; Osberger T. J.; Mateu N.; Sore H. F.; Spring D. R. Recent Applications of Diversity-Oriented Synthesis Toward Novel, 3-Dimensional Fragment Collections. Front. Chem. 2018, 6, 460 10.3389/fchem.2018.00460. 30386766 PMC6198038

[ref34] Mayol-Llinàs J.; Farnaby W.; Nelson A. Modular Synthesis of Thirty Lead-like Scaffolds Suitable for CNS Drug Discovery. Chem. Commun. 2017, 53 (91), 12345–12348. 10.1039/C7CC06078E. 29099137

[ref35] Karageorgis G.; Liver S.; Nelson A. Activity-Directed Synthesis: A Flexible Approach for Lead Generation. ChemMedChem. 2020, 15 (19), 1776–1782. 10.1002/cmdc.202000524. 32734671 PMC7589241

[ref36] Grygorenko O. O.; Radchenko D. S.; Volochnyuk D. M.; Tolmachev A. A.; Komarov I. V. Bicyclic Conformationally Restricted Diamines. Chem. Rev. 2011, 111 (9), 5506–5568. 10.1021/cr100352k. 21711015

[ref37] Meanwell N. A.; Loiseleur O. Applications of Isosteres of Piperazine in the Design of Biologically Active Compounds: Part 1. J. Agric. Food Chem. 2022, 70 (36), 10942–10971. 10.1021/acs.jafc.2c00726. 35675050

[ref38] Visini R.; Arus-Pous J.; Awale M.; Reymond J. L. Virtual Exploration of the Ring Systems Chemical Universe. J. Chem. Inf. Model. 2017, 57, 2707–2718. 10.1021/acs.jcim.7b00457. 29019686

[ref39] Thakkar A.; Kogej T.; Reymond J.-L.; Engkvist O.; Bjerrum E. J. Datasets and Their Influence on the Development of Computer Assisted Synthesis Planning Tools in the Pharmaceutical Domain. Chem. Sci. 2020, 11 (1), 154–168. 10.1039/C9SC04944D. 32110367 PMC7012039

[ref40] Genheden S.; Thakkar A.; Chadimová V.; Reymond J.-L.; Engkvist O.; Bjerrum E. AiZynthFinder: A Fast, Robust and Flexible Open-Source Software for Retrosynthetic Planning. J. Cheminf. 2020, 12 (1), 70 10.1186/s13321-020-00472-1. PMC7672904 33292482

[ref41] Thakkar A.; Selmi N.; Reymond J.-L.; Engkvist O.; Bjerrum E. J. “Ring Breaker”: Neural Network Driven Synthesis Prediction of the Ring System Chemical Space. J. Med. Chem. 2020, 63 (16), 8791–8808. 10.1021/acs.jmedchem.9b01919. 32352286

[ref42] Probst D.; Reymond J.-L. Visualization of Very Large High-Dimensional Data Sets as Minimum Spanning Trees. J. Cheminf. 2020, 12 (1), 12 10.1186/s13321-020-0416-x. PMC7015965 33431043

[ref43] Higgins T. F.; Winkler J. D. Synthesis and Applications of the C2-Symmetrical Diamine 2,7-Diazabicyclo[4.4.1]Undecane. J. Org. Chem. 2020, 85 (11), 7424–7432. 10.1021/acs.joc.0c00829. 32353240

[ref44] Mendez D.; Gaulton A.; Bento A. P.; Chambers J.; De Veij M.; Félix E.; Magariños M. P.; Mosquera J. F.; Mutowo P.; Nowotka M.; Gordillo-Marañón M.; Hunter F.; Junco L.; Mugumbate G.; Rodriguez-Lopez M.; Atkinson F.; Bosc N.; Radoux C. J.; Segura-Cabrera A.; Hersey A.; Leach A. R. ChEMBL: Towards Direct Deposition of Bioassay Data. Nucleic Acids Res. 2019, 47 (D1), D930–D940. 10.1093/nar/gky1075. 30398643 PMC6323927

[ref45] Lagunin A.; Stepanchikova A.; Filimonov D.; Poroikov V. PASS: Prediction of Activity Spectra for Biologically Active Substances. Bioinformatics 2000, 16 (8), 747–748. 10.1093/bioinformatics/16.8.747. 11099264

[ref46] Keiser M. J.; Roth B. L.; Armbruster B. N.; Ernsberger P.; Irwin J. J.; Shoichet B. K. Relating Protein Pharmacology by Ligand Chemistry. Nat. Biotechnol. 2007, 25 (2), 197–206. 10.1038/nbt1284. 17287757

[ref47] Awale M.; Reymond J. L. The Polypharmacology Browser: A Web-Based Multi-Fingerprint Target Prediction Tool Using ChEMBL Bioactivity Data. J. Cheminf. 2017, 9, 11 10.1186/s13321-017-0199-x. PMC5319934 28270862

[ref48] Mayr A.; Klambauer G.; Unterthiner T.; Steijaert M.; Wegner J. K.; Ceulemans H.; Clevert D.-A.; Hochreiter S. Large-Scale Comparison of Machine Learning Methods for Drug Target Prediction on ChEMBL. Chem. Sci. 2018, 9 (24), 5441–5451. 10.1039/C8SC00148K. 30155234 PMC6011237

[ref49] Awale M.; Reymond J. L. Web-Based Tools for Polypharmacology Prediction. Methods Mol. Biol. 2019, 1888, 255–272. 10.1007/978-1-4939-8891-4_15. 30519952

[ref50] Awale M.; Reymond J.-L. Polypharmacology Browser PPB2: Target Prediction Combining Nearest Neighbors with Machine Learning. J. Chem. Inf. Model. 2019, 59 (1), 10–17. 10.1021/acs.jcim.8b00524. 30558418

[ref51] Lavecchia A.; Cerchia C. In Silico Methods to Address Polypharmacology: Current Status, Applications and Future Perspectives. Drug Discovery Today 2016, 21 (2), 288–298. 10.1016/j.drudis.2015.12.007. 26743596

[ref52] Poirier M.; Awale M.; Roelli M. A.; Giuffredi G. T.; Ruddigkeit L.; Evensen L.; Stooss A.; Calarco S.; Lorens J. B.; Charles R.-P.; Reymond J.-L. Identifying Lysophosphatidic Acid Acyltransferase β (LPAAT-β) as the Target of a Nanomolar Angiogenesis Inhibitor from a Phenotypic Screen Using the Polypharmacology Browser PPB2. ChemMedChem. 2019, 14 (2), 224–236. 10.1002/cmdc.201800554. 30520265

[ref53] Morgan H. L. The Generation of a Unique Machine Description for Chemical Structures-A Technique Developed at Chemical Abstracts Service. J. Chem. Doc. 1965, 5 (2), 107–113. 10.1021/c160017a018.

[ref54] Rogers D.; Hahn M. Extended-Connectivity Fingerprints. J. Chem. Inf. Model. 2010, 50 (5), 742–754. 10.1021/ci100050t. 20426451

[ref55] Awale M.; Reymond J. L. Atom Pair 2D-Fingerprints Perceive 3D-Molecular Shape and Pharmacophores for Very Fast Virtual Screening of ZINC and GDB-17. J. Chem. Inf. Model. 2014, 54, 1892–1897. 10.1021/ci500232g. 24988038

[ref56] Chu U. B.; Ruoho A. E. Biochemical Pharmacology of the Sigma-1 Receptor. Mol. Pharmacol. 2016, 89 (1), 142–153. 10.1124/mol.115.101170. 26560551

[ref57] Westerink R. H. S.; Ewing A. G. The PC12 Cell as Model for Neurosecretion. Acta Physiol. 2008, 192 (2), 273–285. 10.1111/j.1748-1716.2007.01805.x. PMC2663028 18005394

[ref58] Figlewicz D. P.; Bentson K.; Ocrant I. The Effect of Insulin on Norepinephrine Uptake by PC12 Cells. Brain Res. Bull. 1993, 32 (4), 425–431. 10.1016/0361-9230(93)90210-3. 8221132

[ref59] Reis-Silva T. M.; Sandini T. M.; Calefi A. S.; Orlando B. C. G.; Moreira N.; Lima A. P. N.; Florio J. C.; Queiroz-Hazarbassanov N. G. T.; Bernardi M. M. Stress Resilience Evidenced by Grooming Behaviour and Dopamine Levels in Male Mice Selected for High and Low Immobility Using the Tail Suspension Test. Eur. J. Neurosci. 2019, 50 (6), 2942–2954. 10.1111/ejn.14409. 30888692

[ref60] Nagai Y.; Kisaka Y.; Nomura K.; Nishitani N.; Andoh C.; Koda M.; Kawai H.; Seiriki K.; Nagayasu K.; Kasai A.; Shirakawa H.; Nakazawa T.; Hashimoto H.; Kaneko S. Dorsal Raphe Serotonergic Neurons Preferentially Reactivate Dorsal Dentate Gyrus Cell Ensembles Associated with Positive Experience. Cell Rep. 2023, 42 (3), 112149 10.1016/j.celrep.2023.112149. 36821440

[ref61] Dunham N. W.; Miya T. S. A Note on a Simple Apparatus for Detecting Neurological Deficit in Rats and Mice. J. Am. Pharm. Assoc. Am. Pharm. Assoc. 1957, 46 (3), 208–209. 10.1002/jps.3030460322. 13502156

[ref62] Takao K.; Miyakawa T. Light/Dark Transition Test for Mice. J. Visualized Exp. 2006, 1, 104 10.3791/104. PMC2504462 18704188

[ref63] Cryan J. F.; Mombereau C.; Vassout A. The Tail Suspension Test as a Model for Assessing Antidepressant Activity: Review of Pharmacological and Genetic Studies in Mice. Neurosci. Biobehav. Rev. 2005, 29 (4–5), 571–625. 10.1016/j.neubiorev.2005.03.009. 15890404

[ref64] Fritze S.; Spanagel R.; Noori H. R. Adaptive Dynamics of the 5-HT Systems Following Chronic Administration of Selective Serotonin Reuptake Inhibitors: A Meta-Analysis. J. Neurochem. 2017, 142 (5), 747–755. 10.1111/jnc.14114. 28653748

[ref65] Gabrielsen M.; Kurczab R.; Siwek A.; Wolak M.; Ravna A. W.; Kristiansen K.; Kufareva I.; Abagyan R.; Nowak G.; Chilmonczyk Z.; Sylte I.; Bojarski A. J. Identification of Novel Serotonin Transporter Compounds by Virtual Screening. J. Chem. Inf. Model. 2014, 54 (3), 933–943. 10.1021/ci400742s. 24521202 PMC3982395

[ref66] Aggarwal S.; Mortensen O. V. Discovery and Development of Monoamine Transporter Ligands. In Drug Development in Psychiatry; Macaluso M.; Preskorn S. H.; Shelton R. C. Eds.; Springer International Publishing: Cham, 2023; pp 101–129. 10.1007/978-3-031-21054-9_4. PMC10074400 36928847

[ref67] Kawai H.; Bouchekioua Y.; Nishitani N.; Niitani K.; Izumi S.; Morishita H.; Andoh C.; Nagai Y.; Koda M.; Hagiwara M.; Toda K.; Shirakawa H.; Nagayasu K.; Ohmura Y.; Kondo M.; Kaneda K.; Yoshioka M.; Kaneko S. Median Raphe Serotonergic Neurons Projecting to the Interpeduncular Nucleus Control Preference and Aversion. Nat. Commun. 2022, 13 (1), 7708 10.1038/s41467-022-35346-7. 36550097 PMC9780347

[ref68] Gertsch J.; Chicca A. CNS Drug Discovery in Academia: Where Basic Research Meets Innovation. ChemBioChem. 2024, 25 (19), e202400397 10.1002/cbic.202400397. 38958639

[ref69] Davies H. M. L.; Manning J. R. C-H Activation as a Strategic Reaction: Enantioselective Synthesis of 4-Substituted Indoles. J. Am. Chem. Soc. 2006, 128, 1060–1061. 10.1021/ja057768+. 16433506

[ref70] Vranesic I.; Ofner S.; Flor P. J.; Bilbe G.; Bouhelal R.; Enz A.; Desrayaud S.; McAllister K.; Kuhn R.; Gasparini F. AFQ056/Mavoglurant, a Novel Clinically Effective mGluR5 Antagonist: Identification, SAR and Pharmacological Characterization. Bioorg. Med. Chem. 2014, 22, 5790–5803. 10.1016/j.bmc.2014.09.033. 25316499

[ref71] Cho H.; Murakami K.; Nakanishi H.; Isoshima H.; Hayakawa K.; Uchida I. REGIOSELECTIVE SYNTHESIS OF SEVERAL HETEROCYCLIC FUSED AZEPANES USING DIISOBUTYLALUMINUM HYDRIDE. Heterocycles 1998, 48 (5), 919–927. 10.3987/COM-98-8104.

[ref72] Fisher M. H.; Mrozik H.; Schoen W. R.; Shih T. L.; Wyvratt M. J. Heterocyclic-Fused Lactams Promote Release of Growth Hormone. US5606054A, 1997

[ref73] Patel S.; Hamilton G. Inhibitors of rip1 kinase and methods of use thereof. US20180170927A1, 2018

[ref74] Jössang-Yanagida A.; Gansser C. Tetrahydropyridoazepines and Tetrahydropyridoazepinones from the Corresponding Dihydroquinolinones. J. Heterocyclic Chem. 1978, 15, 249–251. 10.1002/jhet.5570150213. 33830

[ref75] Martínez L. R.; Gustavo Avila Zarraga J.; Duran M. E.; Ramírez Apam M. T.; Cañas R. Synthesis of Novel Furo, Thieno, and Benzazetoazepines and Evaluation of Their Cytotoxicity. Bioorg. Med. Chem. Lett. 2002, 12, 1675–1677. 10.1016/S0960-894X(02)00232-9. 12039588

[ref76] Delgado A.; Garcia J. M.; Mauleon D.; Minguillon C.; Subirats J. R.; Feliz M.; Lopez F.; Velasco D. Synthesis and Conformational Analysis of 2-Amino-1,2,3,4-Tetrahydro-1-Naphthalenols. Can. J. Chem. 1988, 66 (3), 517–527. 10.1139/v88-088.

[ref77] Franke A.; Lenke D.; Gries J.; Lehmann H. D. Aminopropanol Derivatives of 6-Hydroxy-2,3,4,5-Tetrahydro-1H-1-Benzazepin-2-One and Pharmaceutical Formulations Containing the Said Compounds. US4340595A, 1982

[ref78] Han R.-B.; Shao Y.-P.; Wu H.-F.; Zhang D.; Piao F.-Y. Synthesis and Anticonvulsant Evaluation of Some New 1,3,4,5-Tetrahydro-6-Alkoxy-2H-1-Benzazepin-2-One Derivatives. Med. Chem. Res. 2014, 23 (6), 2810–2820. 10.1007/s00044-013-0871-2.

[ref79] Morozova V.; Pellegata D.; Charles R.-P.; Gertsch J. Carboxylesterase 1-Mediated Endocannabinoid Metabolism in Skin: Role in Melanoma Progression in BRafV600E/Pten–/– Mice. Cancer & Metabolism 2025, 13 (1), 8 10.1186/s40170-025-00378-2. 39934865 PMC11817774

[ref80] Deacon R. M. J. Measuring Motor Coordination in Mice. JoVE 2013, 75, e2609 10.3791/2609-v. PMC3724562 23748408

[ref81] Campos-Cardoso R.; Godoy L. D.; Lazarini-Lopes W.; Novaes L. S.; dos Santos N. B.; Perfetti J. G.; Garcia-Cairasco N.; Munhoz C. D.; Padovan C. M. Exploring the Light/Dark Box Test: Protocols and Implications for Neuroscience Research. J. Neurosci. Methods 2023, 384, 109748 10.1016/j.jneumeth.2022.109748. 36410541

[ref82] Kowalczyk J.; Budzyńska B.; Kurach Ł.; Pellegata D.; El Sayed N. S.; Gertsch J.; Skalicka-Woźniak K. Neuropsychopharmacological Profiling of Scoparone in Mice. Sci. Rep. 2022, 12 (1), 822 10.1038/s41598-021-04741-3. 35039558 PMC8764054

[ref83] Can A.; Dao D. T.; Terrillion C. E.; Piantadosi S. C.; Bhat S.; Gould T. D. The Tail Suspension Test. J. Visualized Exp. 2012, 59, 3769 10.3791/3769-v. PMC3353516 22315011

