# Correction to “Exploring Simple Drug Scaffolds from the GDB Chemical Space Reveals a Chiral Bicyclic Azepane with Potent Neuropharmacology”

**DOI:** 10.1021/acs.jmedchem.6c01144

**Published:** 2026-04-21

**Authors:** Aline Carrel, Adonis Yiannakas, Jaap-Jan Roukens, Ines Reynoso-Moreno, Markus Orsi, Amol Thakkar, Josep Arus-Pous, Daniele Pellegata, Jürg Gertsch, Jean-Louis Reymond

Figure 6 was corrected to match the data provided in the Supporting Information by removing data for a cpd (*R,R*)-**64**, which does not appear in the publication.

**Figure 6:**
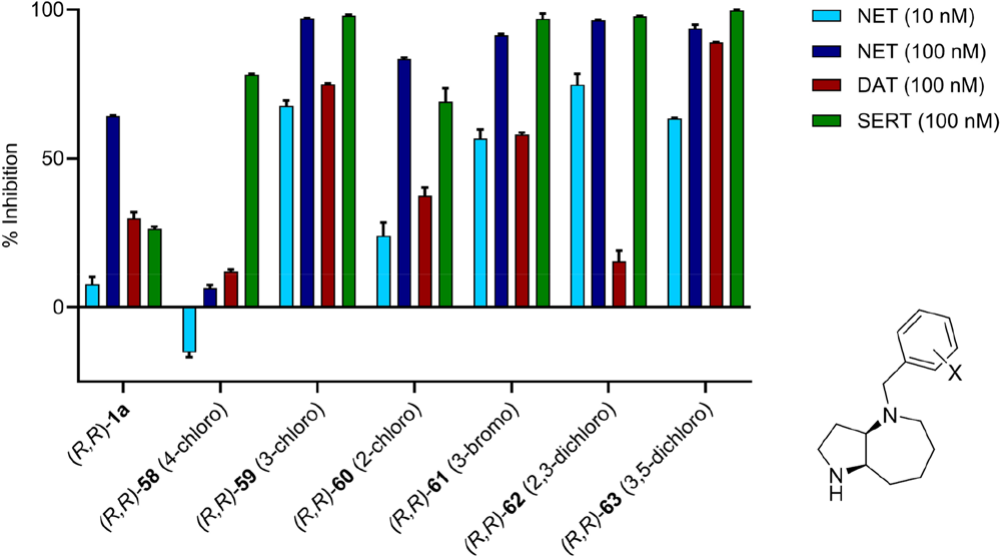
Preliminary structure–activity relationship (SAR) data of halogenated analogues of (*R*,*R*)-**1a**. Bar plot showing the comparative inhibitory activity on monoamine transporters NET, DAT, and SERT. Data are shown as mean of two measurements each performed in triplicates. The experiments were conducted by Eurofins Cerep SA (France) using a radioactive ligand displacement assay.

