# Supplementary material for: Exploring Simple Drug Scaffolds from the Generated Database Chemical Space Reveals a Chiral Bicyclic Azepane with Potent Neuropharmacology
Source: J Med Chem. 2025 Apr 24;68(9):9176–201. doi: 10.1021/acs.jmedchem.4c02549 (PMC12067442; doi:10.1021/acs.jmedchem.4c02549)
Supplement: Supplementary file 1 — jm4c02549_si_001.pdf [file jm4c02549_si_001.pdf]

Supporting Information

Exploring simple drug scaffolds from the GDB chemical space reveals a chiral bicyclic azepane with potent neuropharmacology

Aline Carrel,[a)] Adonis Yiannakas,[b)#] Jaap-Jan Roukens,[b)#] Ines Reynoso-Moreno[b], Markus Orsi,[a)] Amol Thakkar,[a)] Josep Arus-Pous,[a)] Daniele Pellegata[b], Jürg Gertsch,[b)*] and Jean-Louis Reymond [a)*]

[a)] *Department of Chemistry, Biochemistry and Pharmaceutical Sciences, University of Bern, Freiestrasse 3, 3012 Bern, Switzerland,*

[b)] *Institute of Biochemistry and Molecular Medicine, University of Bern, Gertrud-Woker Strasse 5, 3012 Bern, Switzerland*

E-Mail: jean-louis.reymond@unibe.ch

E-Mail: juerg.gertsch@unibe.ch

[#]These authors contributed equally

Table of contents

1 CHEMICAL SYNTHESIS S4

1.1 SUPPORTING FIGURES S4
1.1.1 FIGURE S1 .... S4
1.2 SUPPORTING SCHEMES FOR SYNTHESIS S5
1.2.1 SCHEME S1 .... S5
1.2.2 SCHEME S2 .... S6
1.2.3 SCHEME S3 .... S7
1.3 METHODS S8
1.3.1 GENERAL S8

2 ^{1}H- AND ^{13}C-NMR SPECTRA S10

3 CHIRAL HPLC S80

3.1 CHEMICAL PURITY S81

4 ANALYTICAL PURITY FOR TESTED COMPOUNDS S83

4.1 TABLE S1 .... S83

5 X-RAY CRYSTAL DEPOSITION S84

5.1 FIGURE S2 .... S84

6 PPB2 TARGET PREDICTION FOR FUSED AZEPANES S86

6.1 TABLE S2 .... S86
6.2 FIGURE S3 .... S87
6.3 FIGURE S4 .... S88
6.4 FIGURE S5 .... S89
6.5 FIGURE S6 .... S90
6.6 FIGURE S7 .... S91
6.7 FIGURE S8 .... S92
6.8 FIGURE S9 .... S93
6.9 FIGURE S10 .... S94

8 BIOCHEMICAL ASSAY PROCEDURES S95

9 BIOLOGICAL ASSAYS S98

9.1 FIGURE S11 .... S98
9.2 FIGURE S12 .... S99
9.3 FIGURE S13 .... S100

9.4 FIGURE S14 .... S101
9.5 TABLE S3 .... S101
9.6 TABLE S4 .... S102
9.7 FIGURE S15 .... S102

10 REFERENCES S103

1 Chemical Synthesis

1.1 Supporting Figures

1.1.1 Figure S1

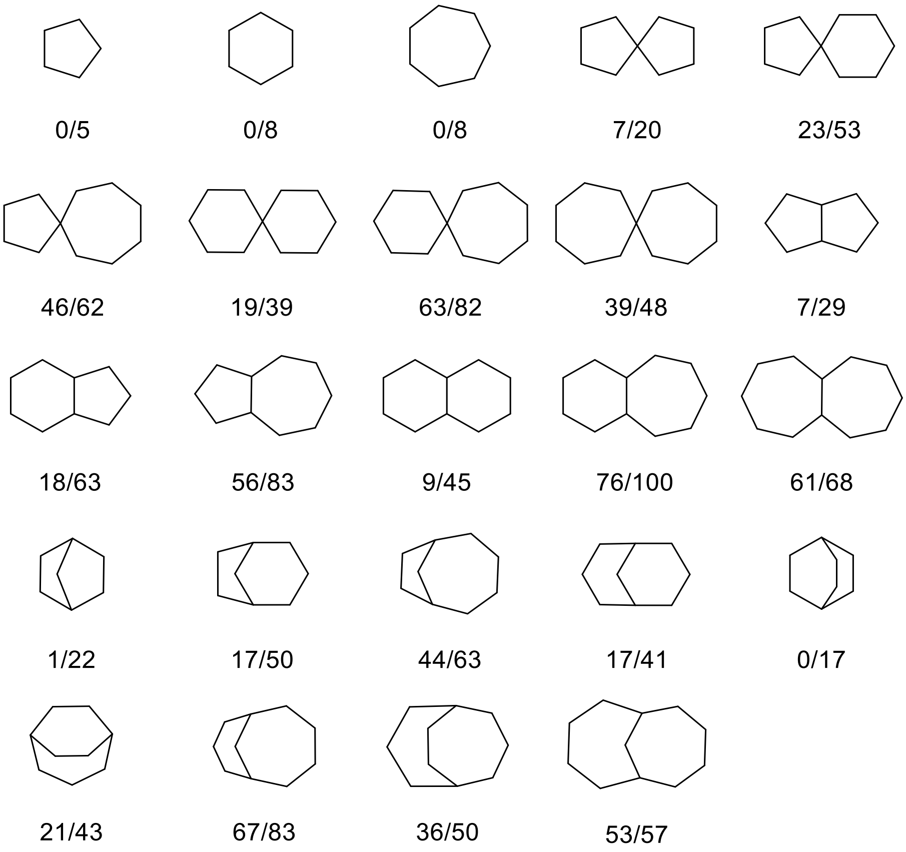

Figure S1. The 24 monocyclic and bicyclic ring systems consisting of 5- , 6- or 7-membered rings extracted from GDB-4c. The number of novel/enumerated amine or diamine scaffolds is indicated below each ring system. Scaffolds not occurring in PubChem were labeled as novel.

1.2 Supporting Schemes for Synthesis

1.2.1 Scheme S1

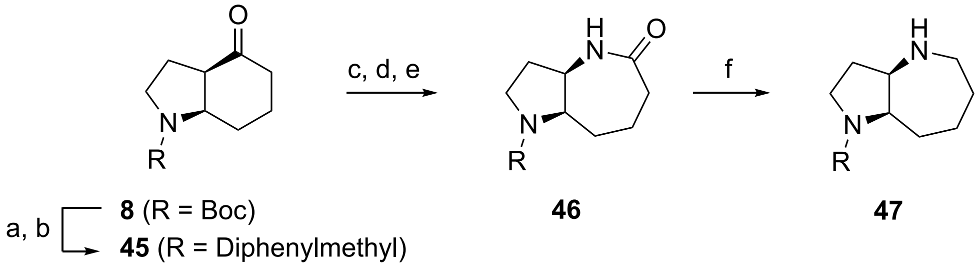

Scheme S1. Synthesis of (5,7)-*cis* fused diamine with diphenylmethyl protecting group. Conditions: a) TFA, DCM, 22 °C, 2 h; b) Bromodiphenylmethane, Cs_2CO_3, DMF, 60 °C, 24 h, 8% over two steps; c) NH_2OH•HCl, pyr, 22 °C, 2 h; d) *p*-TsCl, pyr, 22 °C, 2 h; e) KOAc, EtOH/H_2O, 100 °C, 16 h, 24% over three steps; f) $LiAlH_4$, THF, 0 °C then 22 °C, 4 h, quant.

1.2.2 Scheme S2

A)

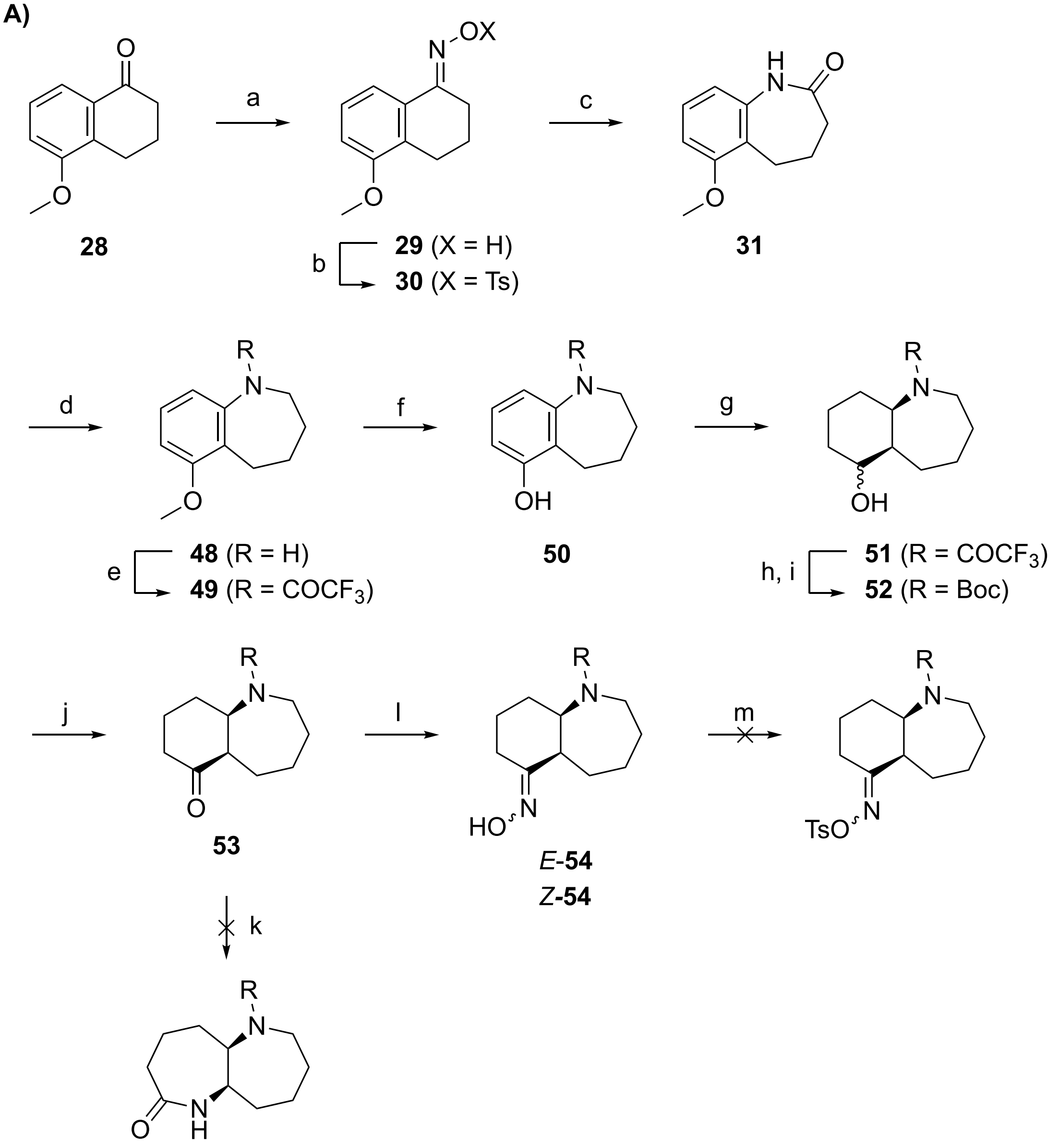

B)

side product formation:

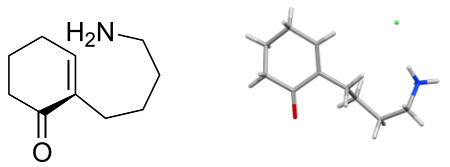

Scheme S2. Synthesis towards (7,7)-*cis*-fused diamine. **A)** Conditions: a) $NH_2OH{\bullet}HCl$, NaOH, EtOH, reflux, 2 h, quant.; b) *p*-TsCl, pyr, acetone/H_2O, 22 °C, 24 h, quant.; c) AcOH, H_2O, 70 °C, 24 h, 75%; d) $LiAlH_4$, THF, 0 °C then reflux, 15 h, 81%; e) TFAA, pyr, DCM, 22 °C, 2 h 99%; f) BBr_3, DCM, -78 °C to 22 °C, 24 h, 93%; g) Rh/C, H_2 (20 bar), AcOH, iPrOH, 70 °C, 2 d, quant.; h) LiOH, THF/H_2O, reflux, 24 h; i) Boc_2O, NEt_3, DMAP, DCM, 22 °C, 24 h, 63% over two steps; j) DMP, DCM, 22 °C, 2 h, 54%; k) H_2SO_4, NaN_3, $CHCl_3$, 0 °C to 22 °C, 24 h; l) $NH_2OH{\bullet}HCl$, NaOAc, MeOH/H_2O, 70 °C, 4 h, 44%, *E*:*Z* 1.3:1. m) *p*-TsCl, pyr, acetone/H_2O, 22 °C, 24 h; **B)** During oxidation and oxime formation a ring-opening side product was

observed, which most probably contributed to lower yields in these steps. Crystallization at a later stage proved the open ring conformation.

1.2.3 Scheme S3

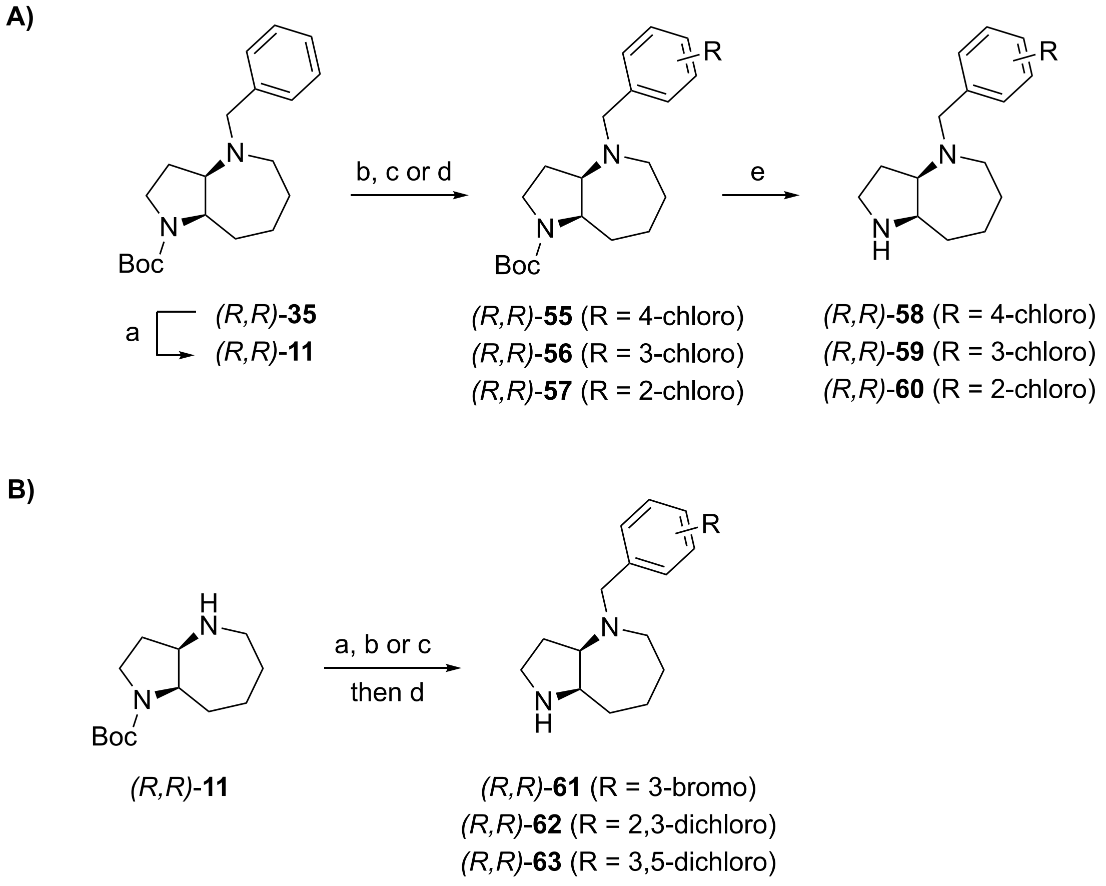

Scheme S3. Synthesis of halogenated derivatives of hit compound (*R,R*)-**1a**. **A)** Conditions: a) Pd/C, H_2 (1 bar), MeOH, 22 °C, 24 h; b) 4-chlorobenzaldehyde, $NaBH_3CN$, MeOH, reflux, 24 h, 8% over two steps; c) 3-chlorobenzaldehyde, $NaBH_3CN$, AcOH, MeOH, reflux, 24 h, 56% over two steps; d) 2-chlorobenzaldehyde, $NaBH_3CN$, AcOH, MeOH, reflux, 24 h, 56% over two steps; e) TFA, DCM, 22 °C, 2 h, (*R,R*)-**58** quant., (*R,R*)-**59**: 80%, (*R,R*)-**60**: 46%;

B) Conditions: a) 3-bromobenzaldehyde, $NaBH_3CN$, AcOH, MeOH, reflux, 24 h; b) 2,3-dichlorobenzaldehyde, $NaBH_3CN$, AcOH, MeOH, reflux, 24 h; d) 3,5-dichlorobenzaldehyde, $NaBH_3CN$, AcOH, MeOH, reflux, 24 h; e) TFA, DCM, 22 °C, 2 h, 29% over two steps for (*R,R*)-**61**, (*R,R*)-**62**, (*R,R*)-**63**.

1.3 Methods

1.3.1 General

Reagents. Commercially available chemicals were purchased from commercial suppliers: Sigma Aldrich, Fluorochem, Alfa Aesar, Combi-Blocks, Toronto Research Chemicals, Apollo Scientific and others. They were used without further purification unless otherwise stated.

Solvents. Dry solvents for reaction (DCM, THF, toluene) were obtained by an in-house dry solvent system (filtration over alumina under a positive pressure of argon). Other dry solvents (DMF, pyridine) were purchased form commercial sources. Solvents used for non-anhydrous reactions, extractions and chromatography (acetic acid, acetone, $CHCl_3$, CH_3CN, DCM, EtOAc, EtOH, heptane, ^{i}PrOH, MeOH, THF) were bought in technical quality and distilled before use or bought in HPLC grade from commercial suppliers.

Reactions. If necessary, reactions were performed using standard Schlenk techniques in flame-dried flasks under a positive pressure of argon. Reactions were monitored by thin-layer chromatography (TLC) or by analytical liquid chromatography mass spectrometry (LCMS).

Thin-layer chromatography (TLC). TLC was conducted on pre-coated aluminum sheets (0.2 mm silica gel 60 with fluorescent indicator, ALUGRAM® Xtra SIL G/UV_{254} by Macherey-Nagel). Visualization was performed by either UV light (254 nm) or by staining with potassium permanganate solution or ninhydrin solution.

Column chromatography. Adsorption column chromatography was carried out with SiO_2 (pore size: 60 Å, 230-400 mesh particle size by Sigma Aldrich) and distilled technical solvents.

NMR spectra (^{1}H, ^{13}C). NMR spectra were recorded at 22 °C unless otherwise stated. Chemical shifts (δ) are reported in ppm relative to the signal of tetramethylsilane (TMS) and residual solvent signals in ^{1}H and ^{13}C NMR spectra were used as internal reference. Coupling constants (J) are given in Hz. The apparent resonance multiplicity is described as s (singlet), d (doublet), t (triplet), q (quartet), quint. (quintet), m (multiplet), or combinations thereof and broad signals are indicated as br (broad). Atom to peak assignment was performed through standard 2D NMR techniques such as COSY, HSQC, HMBC, and NOESY. ^{1}H and ^{13}C -NMR spectra were measured either on a Bruker Avance III 300 spectrometer (at 300 MHz and 75

MHz, respectively) or on a Bruker Avance II 400 spectrometer (at 400 MHz and 101 MHz, respectively).

High-resolution mass spectrometry (HR-MS). HR-MS analyses were performed by the mass spectrometry service of the department of chemistry and biochemistry at the University of Bern on an LTQ Orbitrap XL with nano ESI (Thermo).

Single Crystal X-ray diffraction. Single crystal X-ray diffractions were measured by the XRD-service of the University of Bern and the experiments are carried out using Oxford Diffraction (now Agilent) SuperNova, equipped with Mo micro-source and Oxford cryosystem 700 for low/high temperature measurements.

Recrystallization of *p*-TsCl. Upon prolonged standing the chemical develops impurities of *p*-toluenesulfonic acid and HCl. Therefore, tosyl chloride was purified by dissolving 10 g in $CHCl_3$ (25 mL), filtering and diluting with petroleum ether (125 mL) to precipitate impurities. The solution was clarified with charcoal, filtered and concentrated to yield 8 g of white crystals.

Fieser work-up. The following procedure was used to work-up reactions with x g of $LiAlH_4$.

1. Dilute with THF and cool to 0°C
2. Slowly add x mL deion. H_2O
3. Add x mL aq. NaOH (15%)
4. Add 3x mL deion. H_2O
5. Warm to room temperature and stir 15 min
6. Add some anhydrous $MgSO_4$
7. Stir 15 min and filter to remove salts

HCl and TFA salt formation. The HCl and TFA salts were obtained by treatment of the dried purified diamines with HCl in MeOH or TFA in MeOH (3M solutions), 3 eq. per amine. The solvent was evaporated and the procedure was repeated once.

2 ^{1}H- and ^{13}C-NMR spectra

<u>*tert*-butyl 4-oxo-4,5,6,7-tetrahydro-1H-indole-1-carboxylate (**6**)</u>

^{1}H-NMR

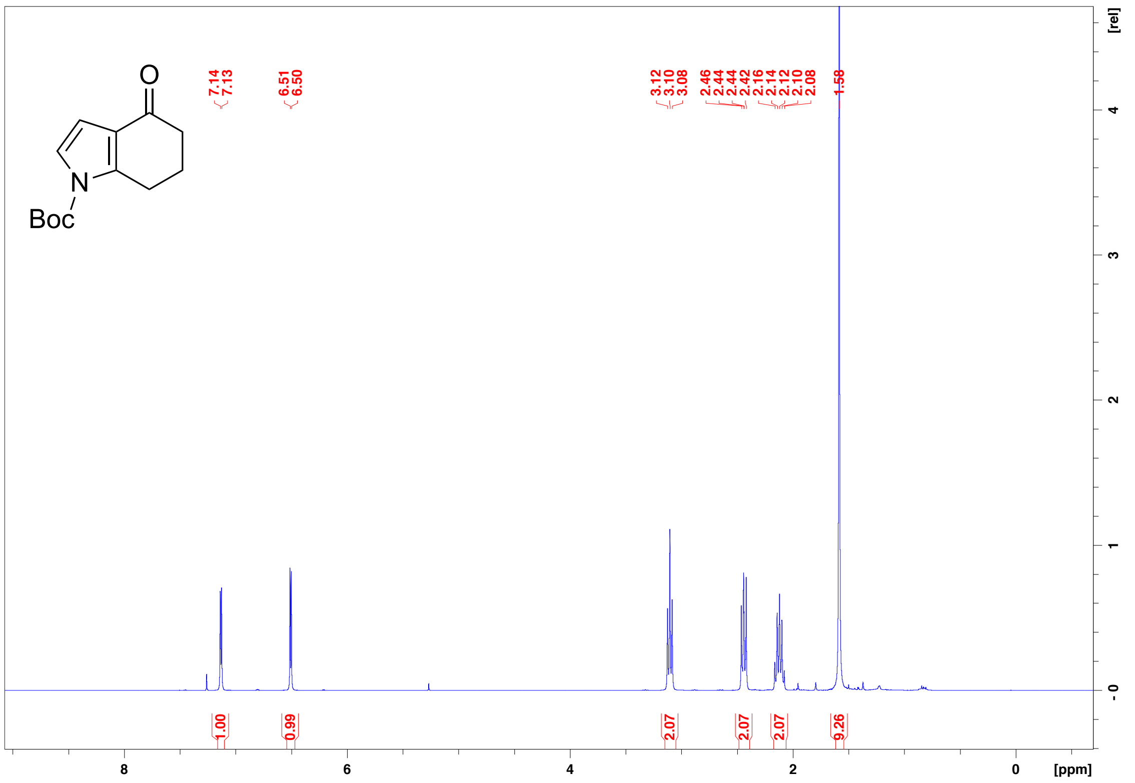

<u>*tert*-butyl-4-hydroxyoctahydro-1H-indole-1-carboxylate ((±)-**7**)</u>

^{1}H-NMR

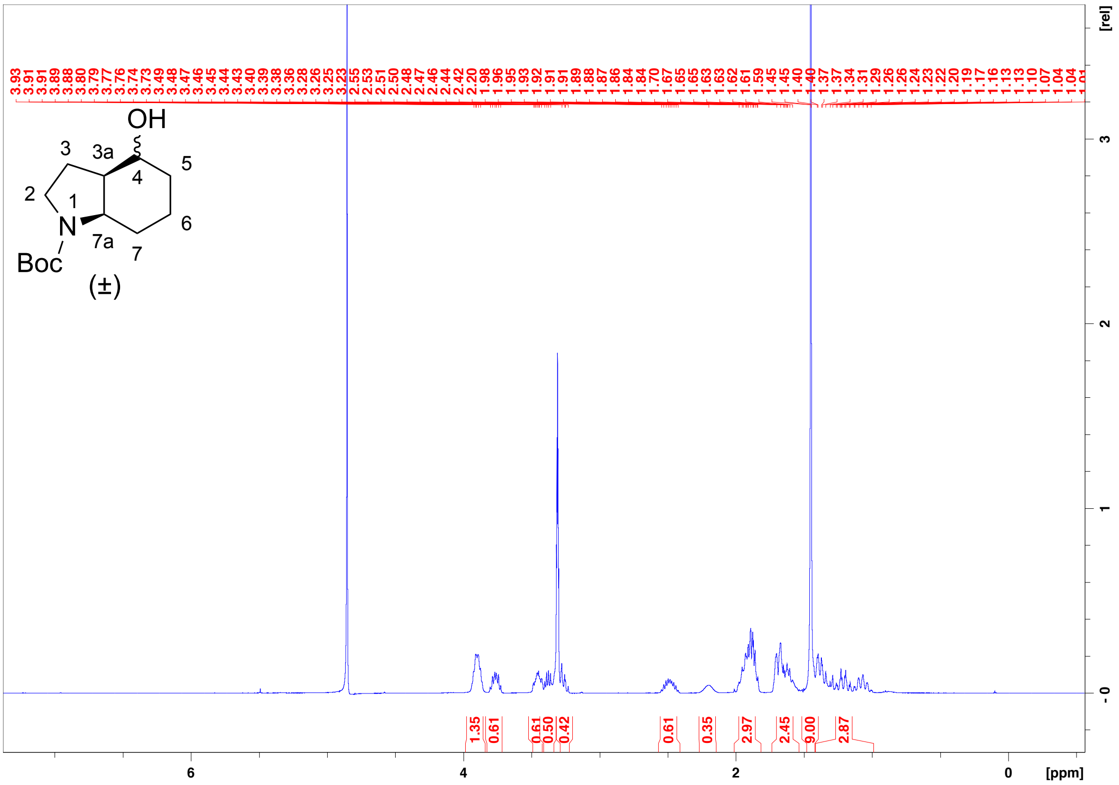

^{13}C-NMR

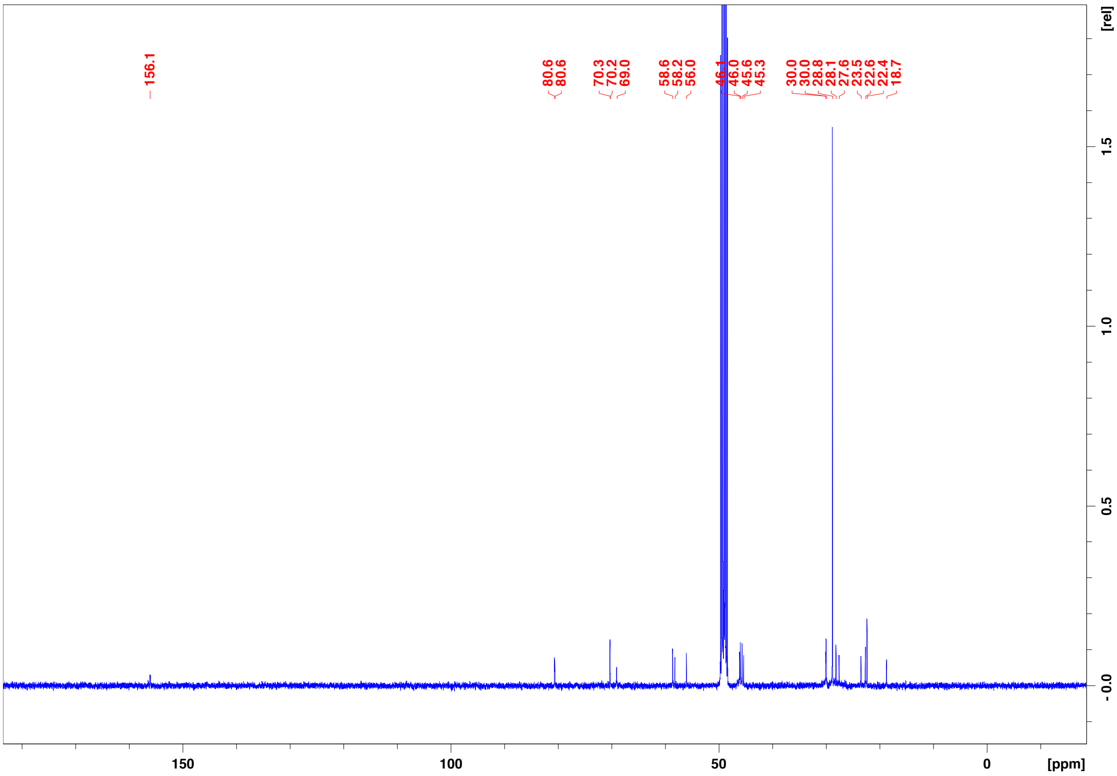

tert-butyl-4-oxooctahydro-1H-indole-1-carboxylate ((±)-**8**)

^{1}H-NMR

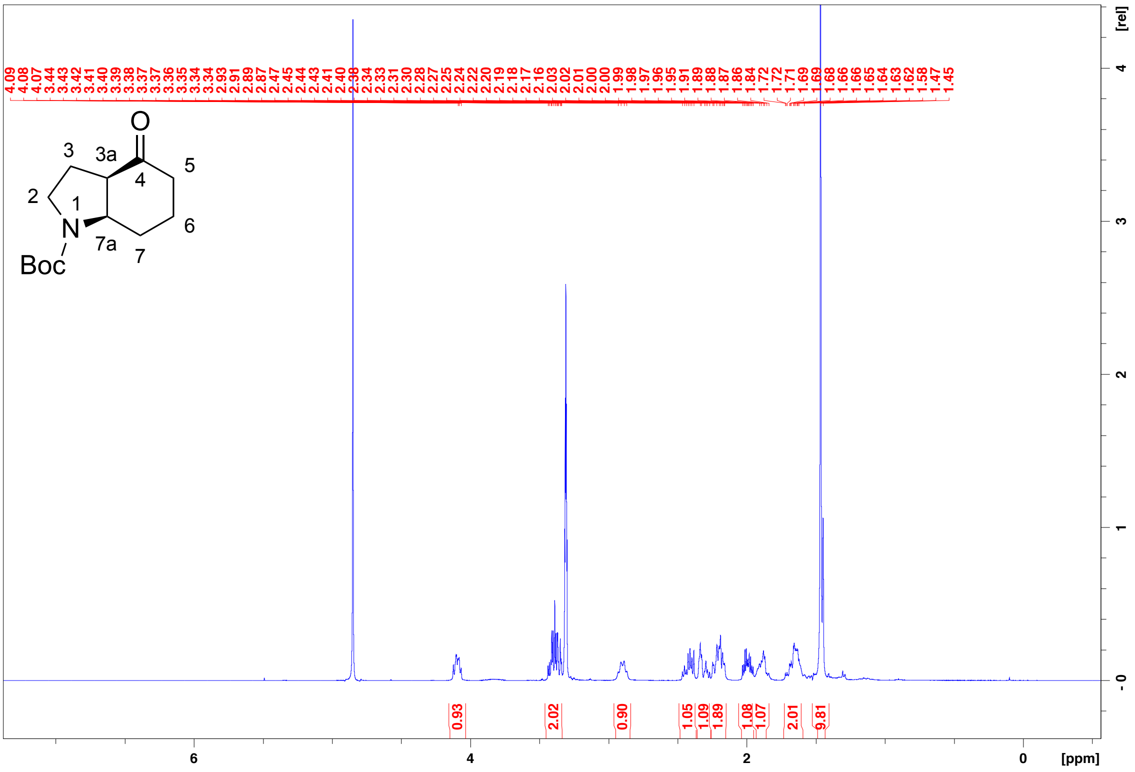

^{13}C-NMR

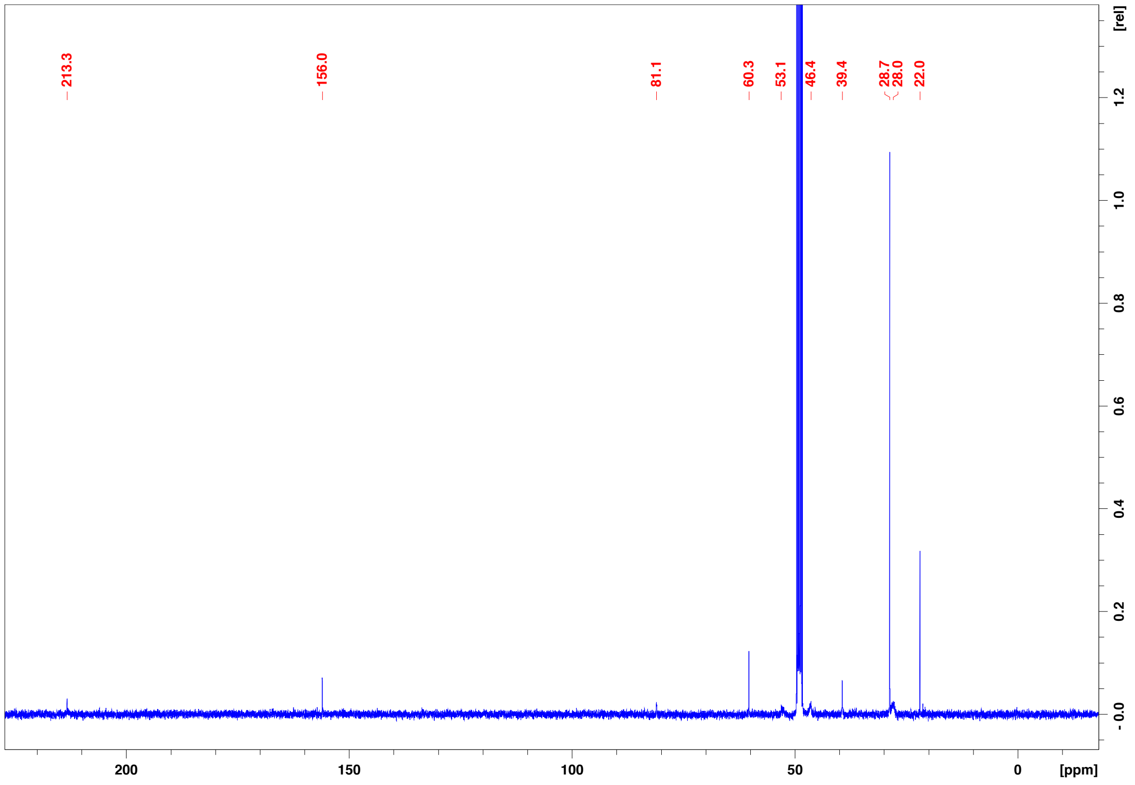

tert-butyl-5-oxooctahydropyrrolo[3,2-b]azepine-1(2H)-carboxylate ((±)-**10**)

^{1}H-NMR

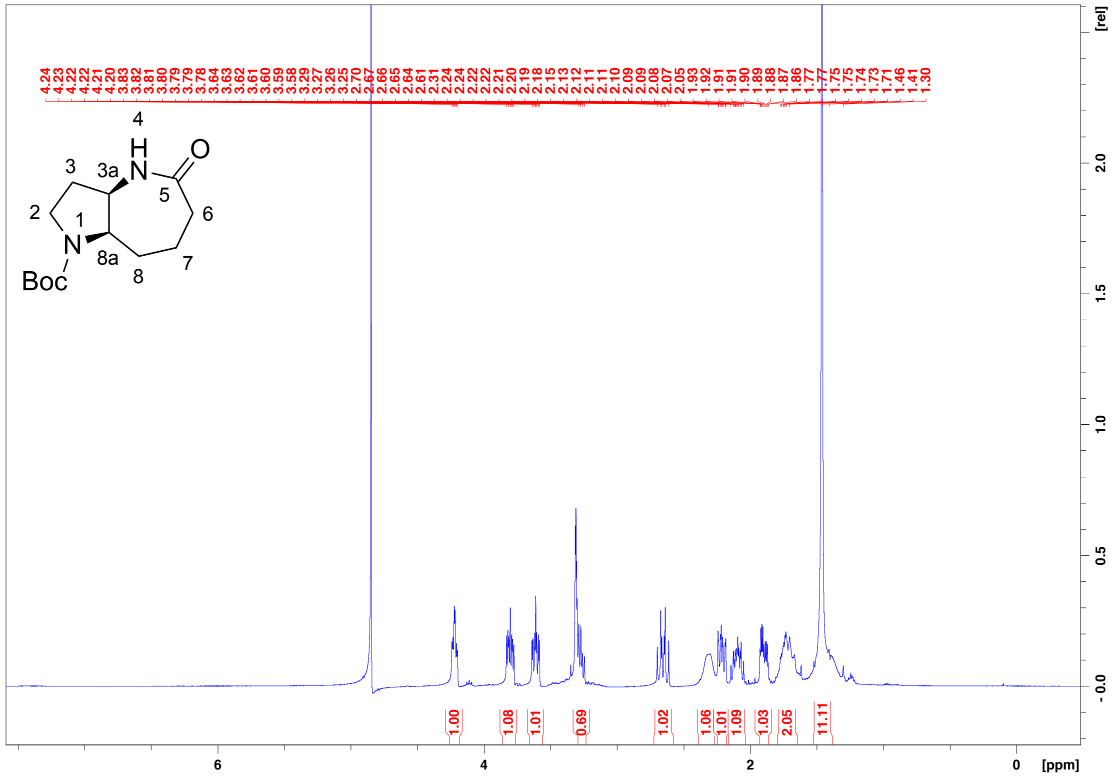

^{13}C-NMR

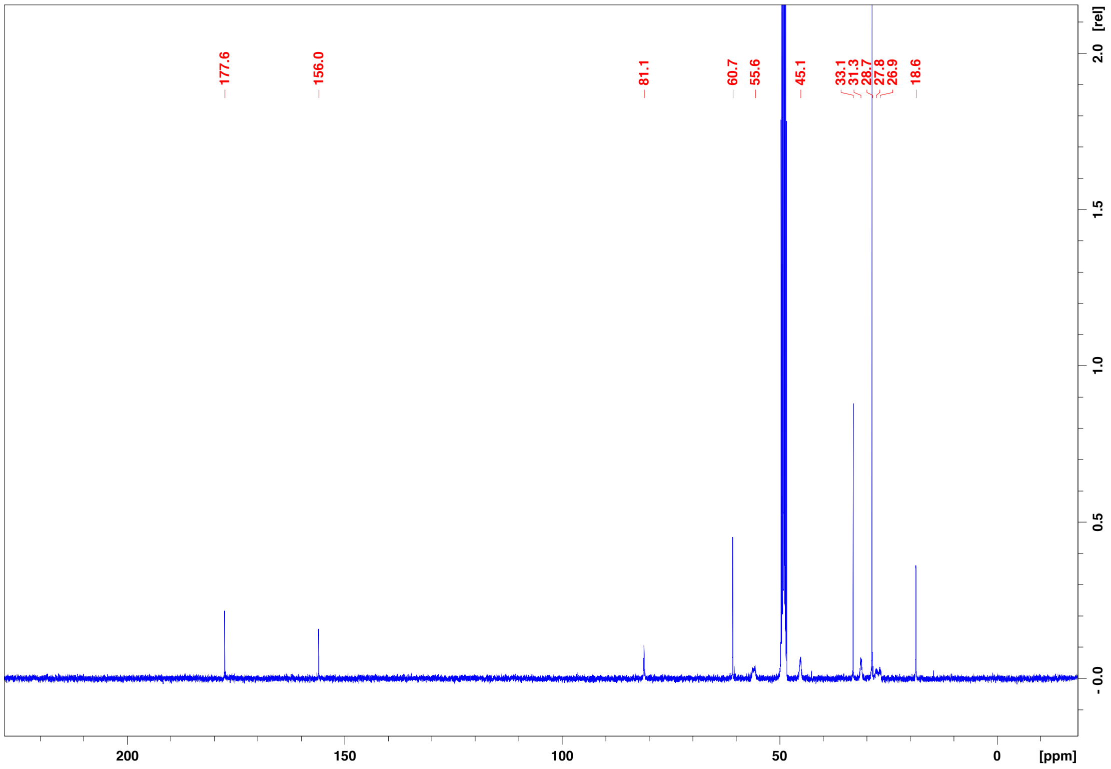

tert-butyl (3a*S*,8a*S*)-4-oxooctahydropyrrolo[3,2-c]azepine-1(2H)-carboxylate ((±)-**9**)

^{1}H-NMR

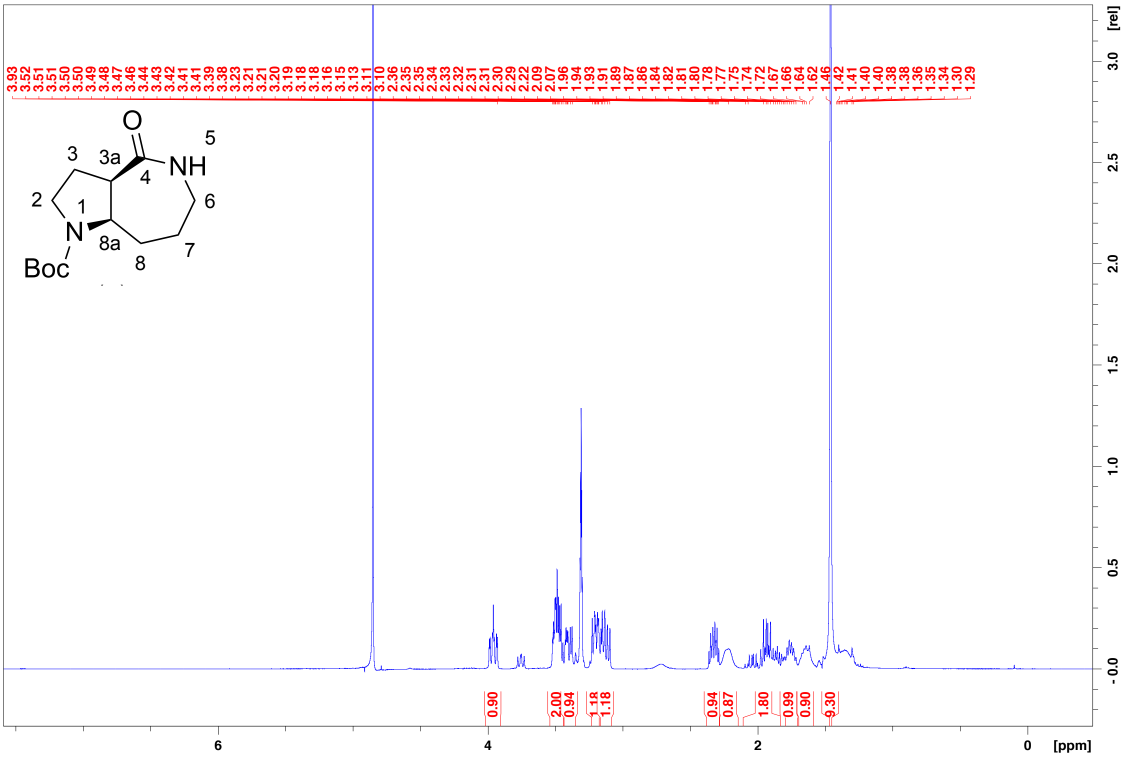

^{13}C-NMR

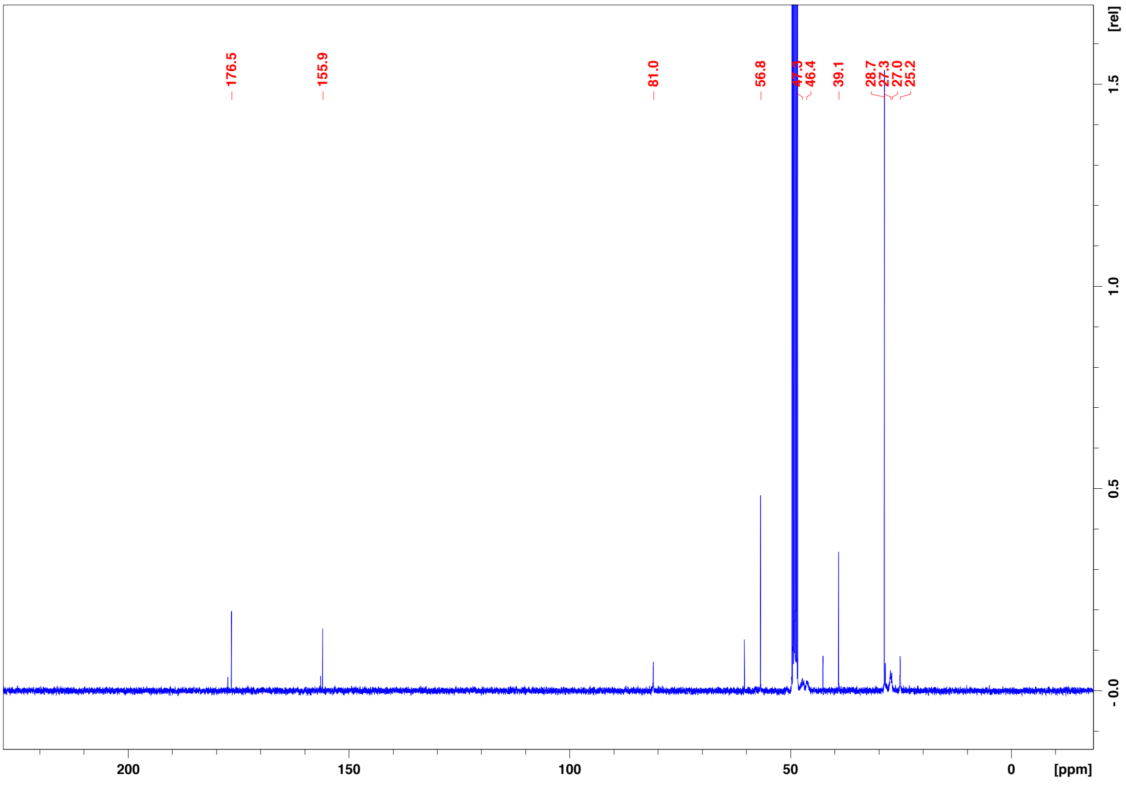

tert-butyl-octahydropyrrolo[3,2-b]azepine-1(2H)-carboxylate ((±)-**11**)

^{1}H-NMR

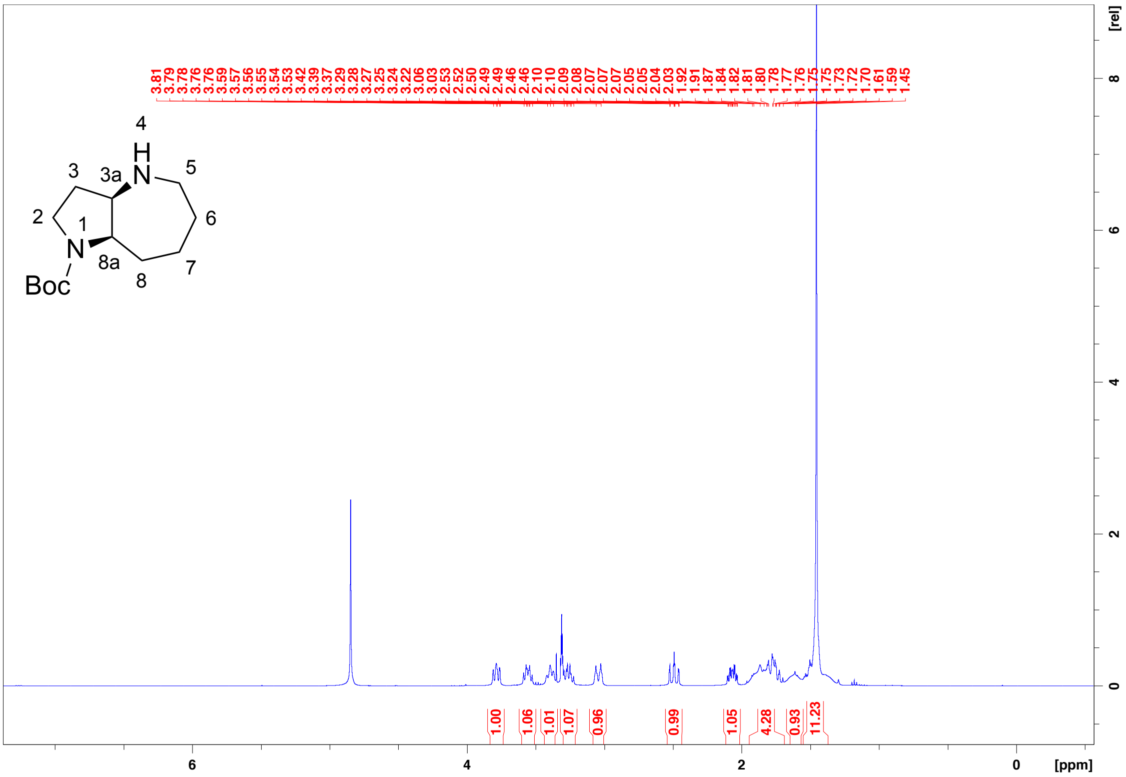

^{13}C-NMR

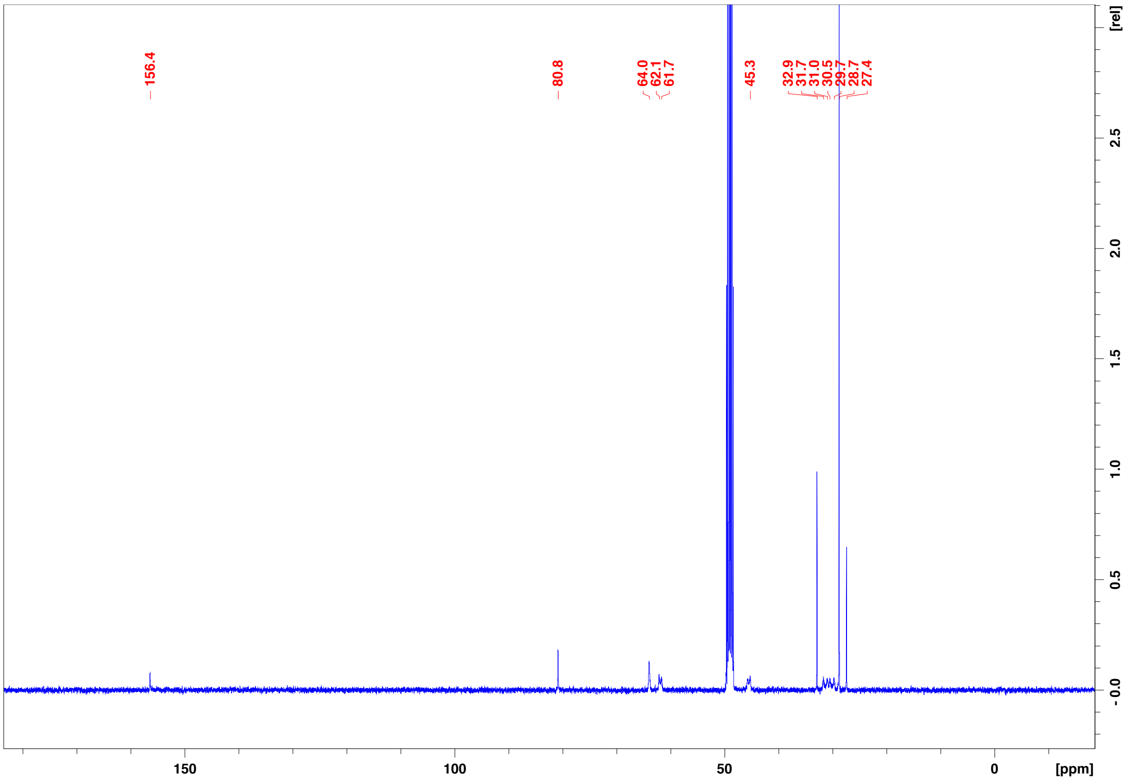

<u>Decahydropyrrolo[3,2-b]azepine ((±)-**2a**)</u>

^{1}H-NMR

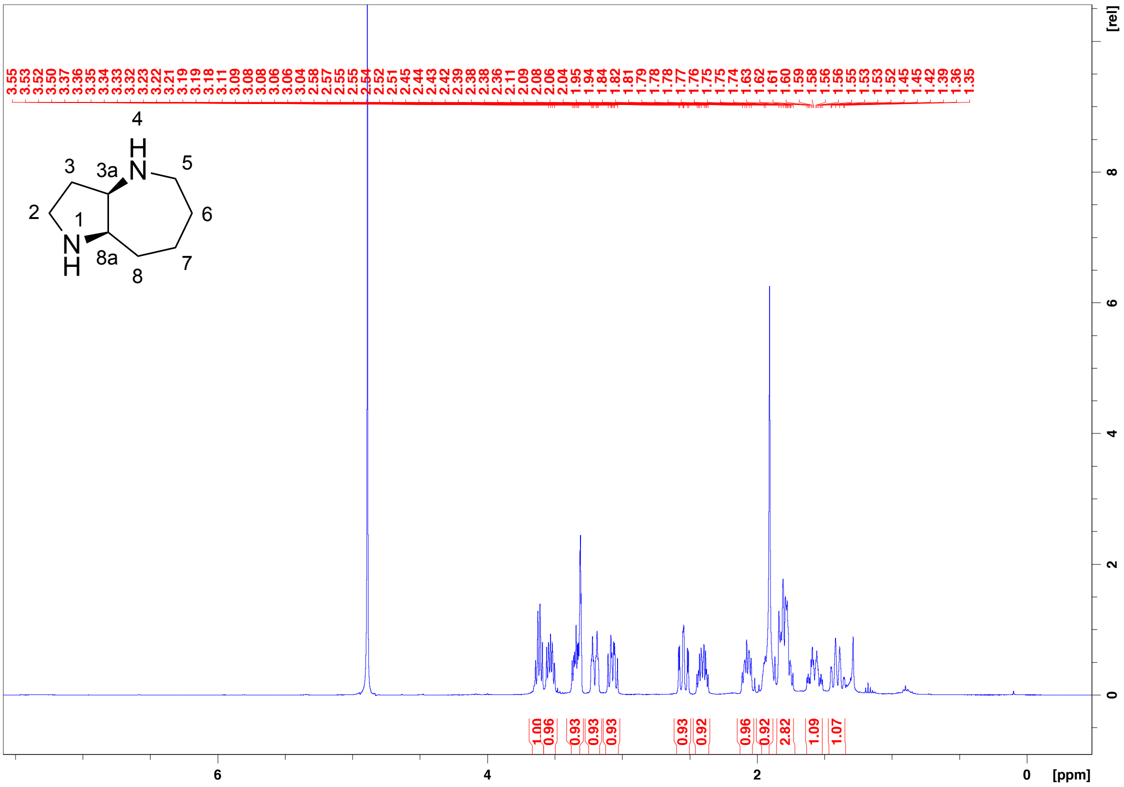

^{13}C-NMR

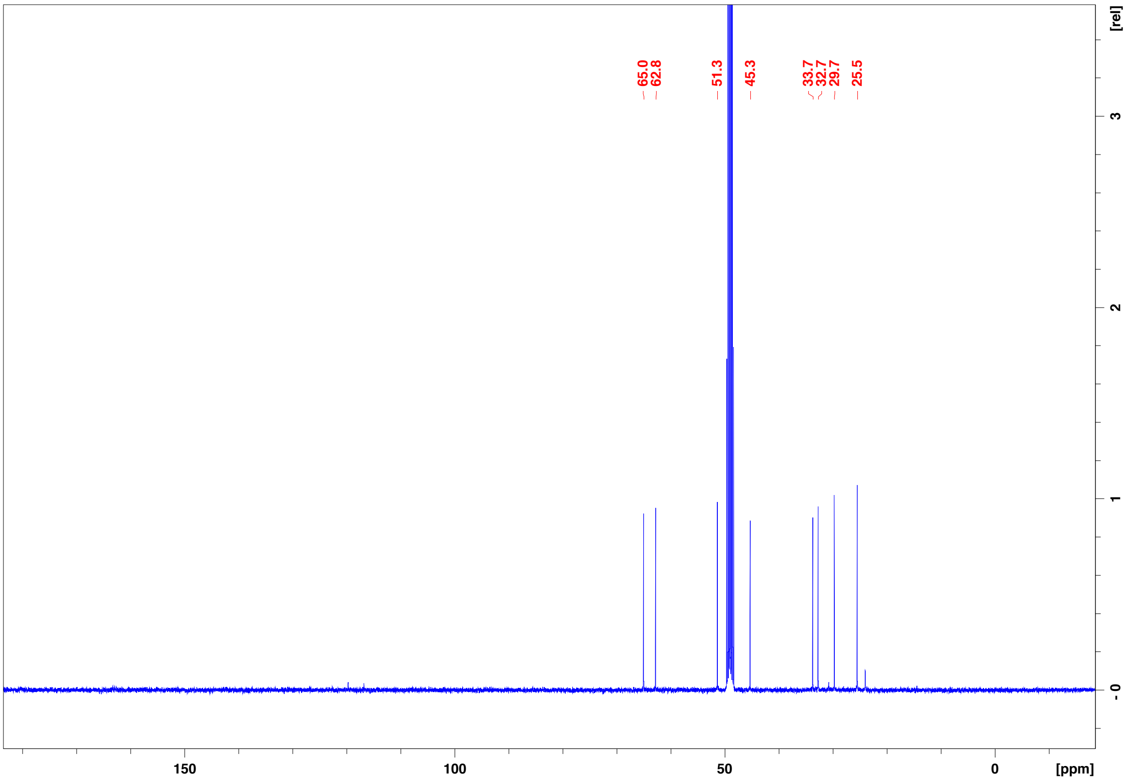

(E)-1,5,6,7-tetrahydro-4H-indol-4-one oxime (E-**12**)

^{1}H-NMR

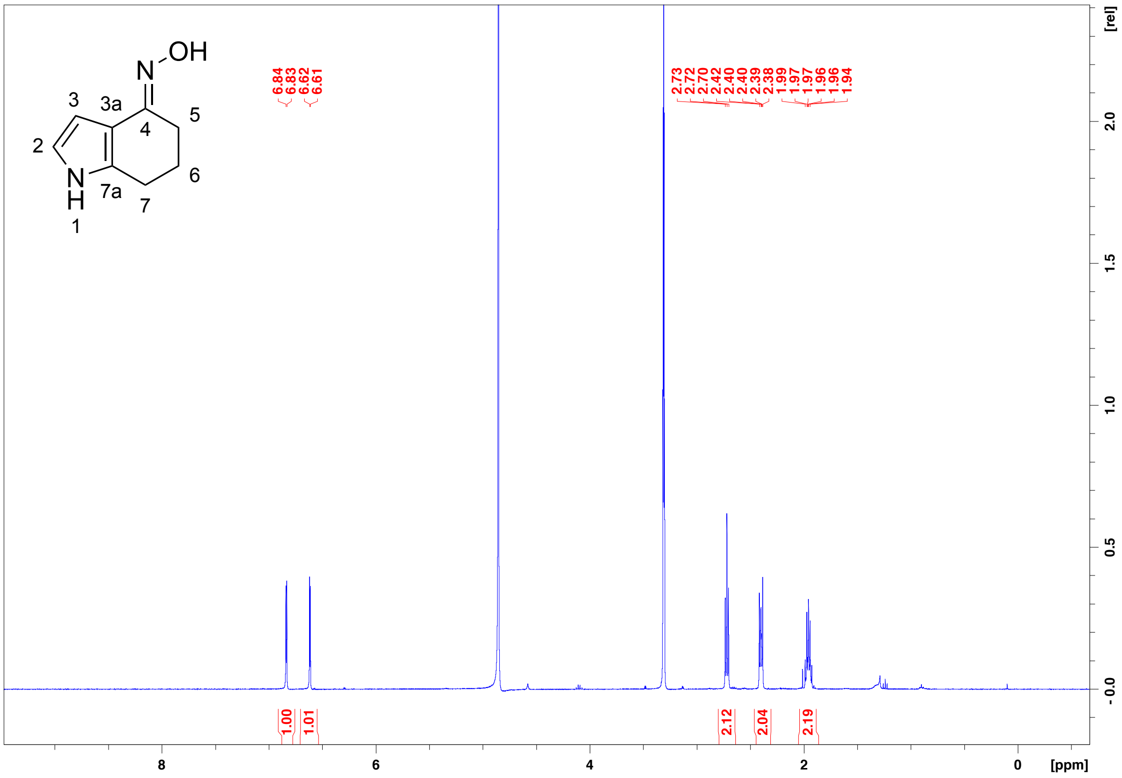

^{13}C-NMR

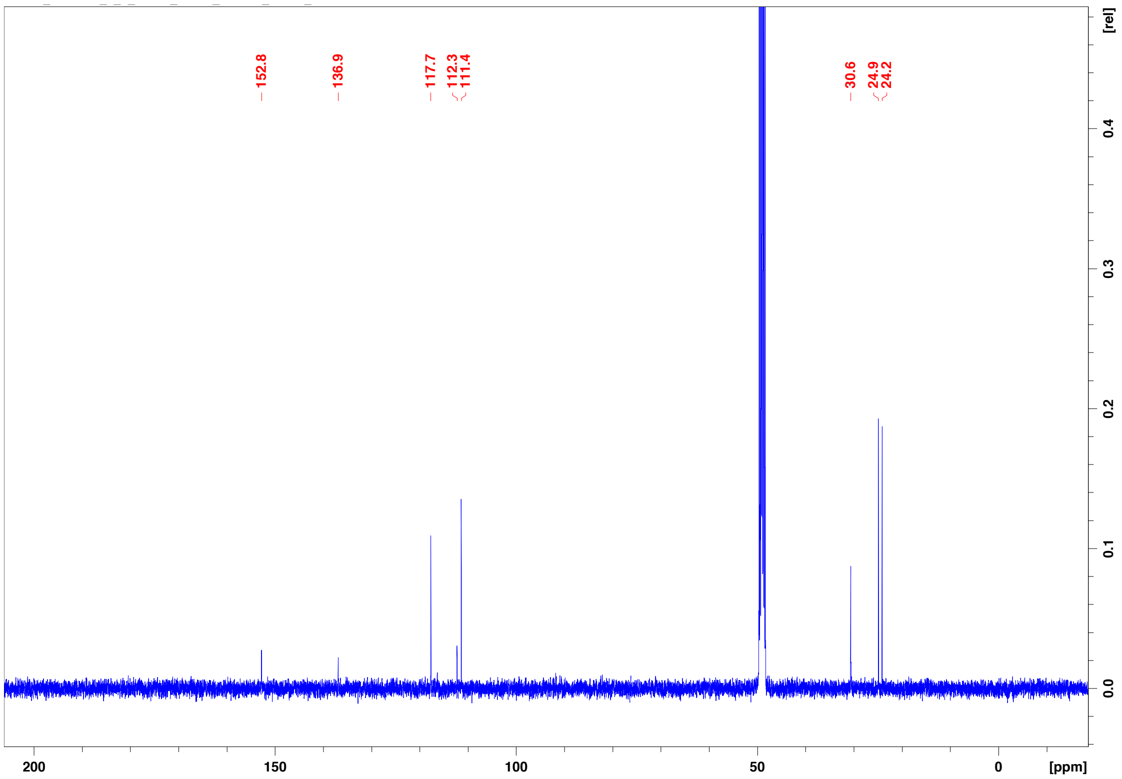

(Z)-1,5,6,7-tetrahydro-4H-indol-4-one oxime (Z-**12**)

^{1}H-NMR

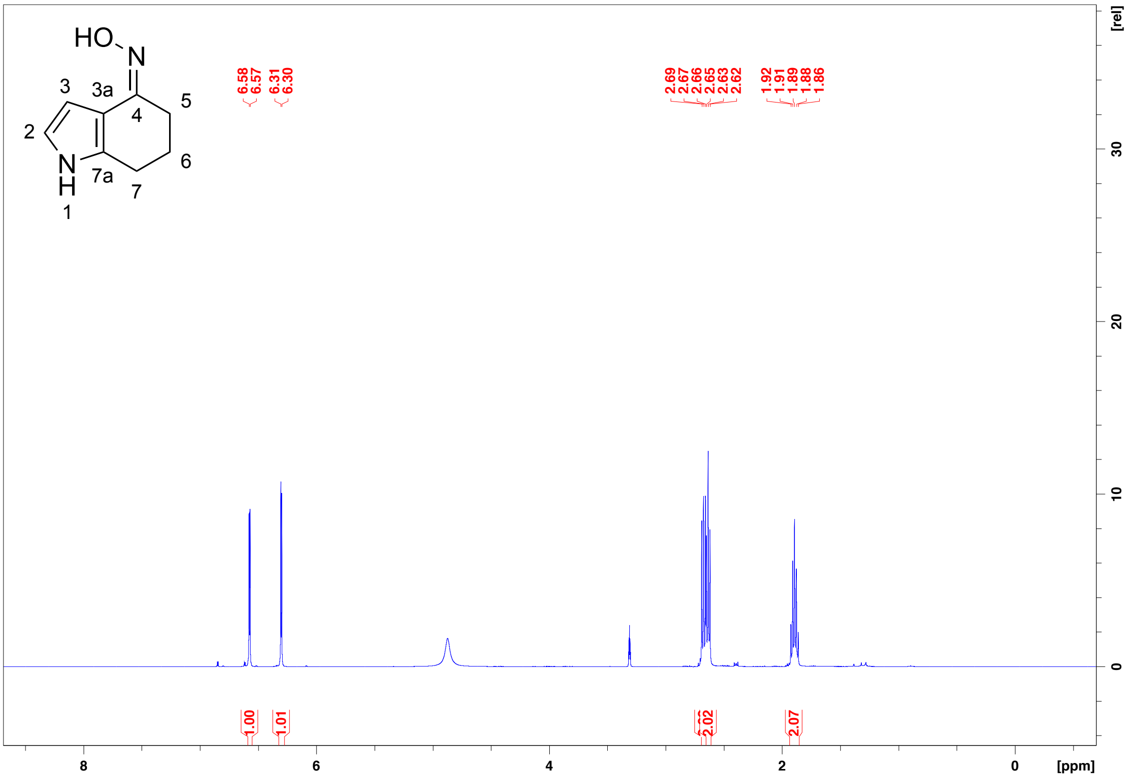

^{13}C-NMR

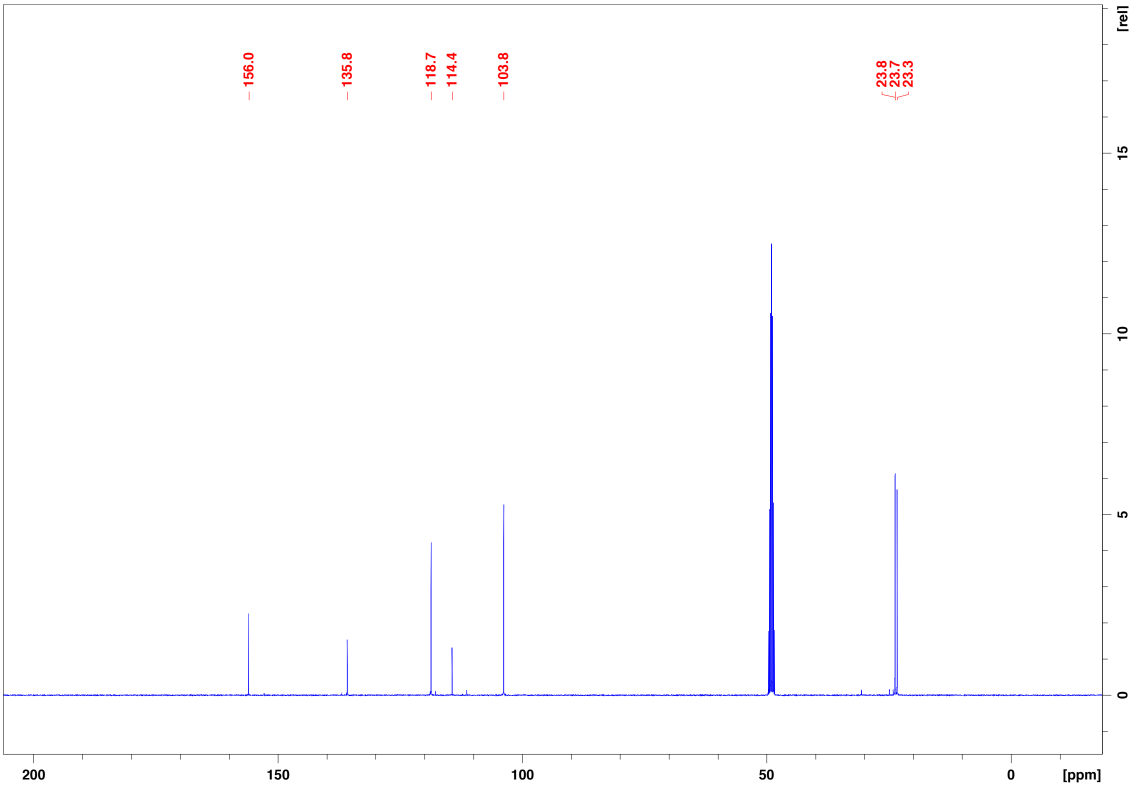

(E)-1,5,6,7-tetrahydro-4H-indol-4-one *O*-tosyl oxime (**13**)

^{1}H-NMR

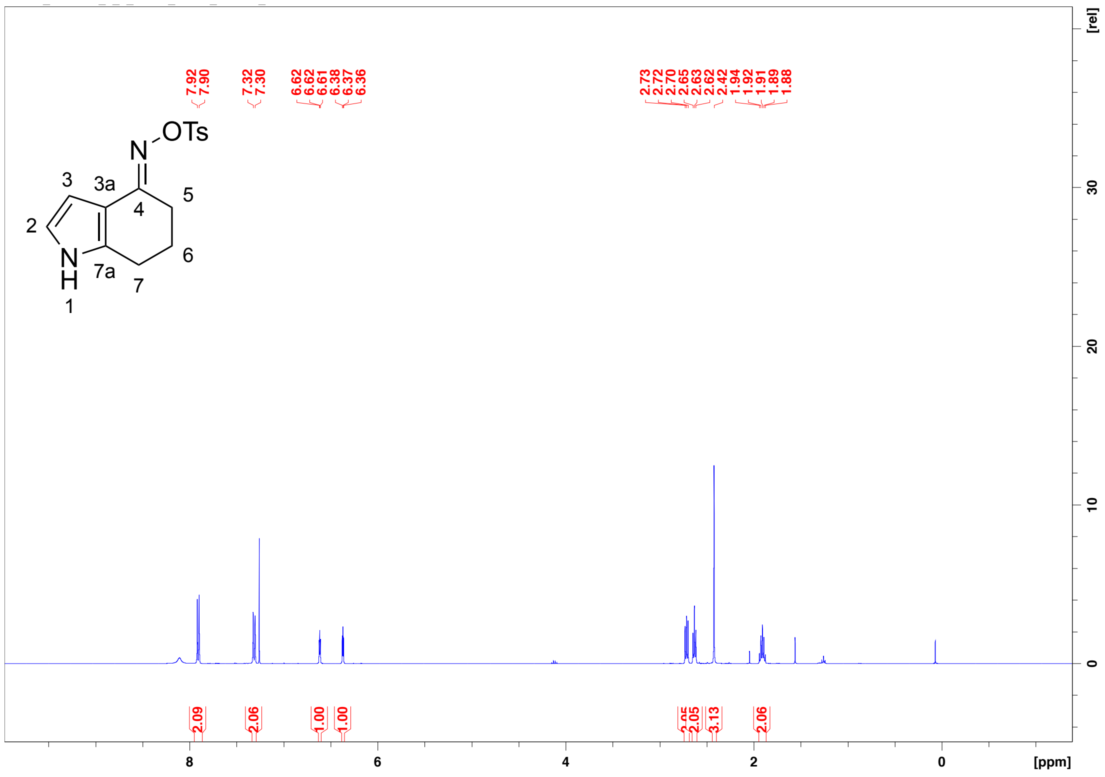

^{13}C-NMR

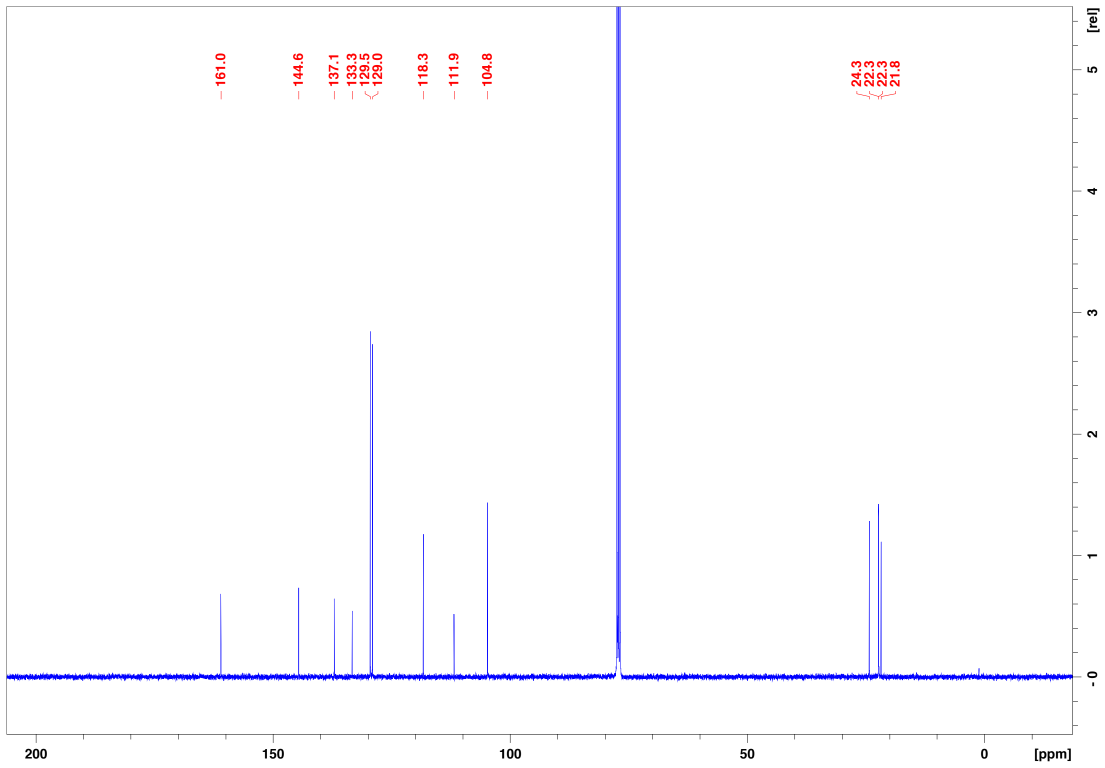

4,6,7,8-tetrahydropyrrolo[3,2-b]azepin-5(1H)-one (**14**)

^{1}H-NMR

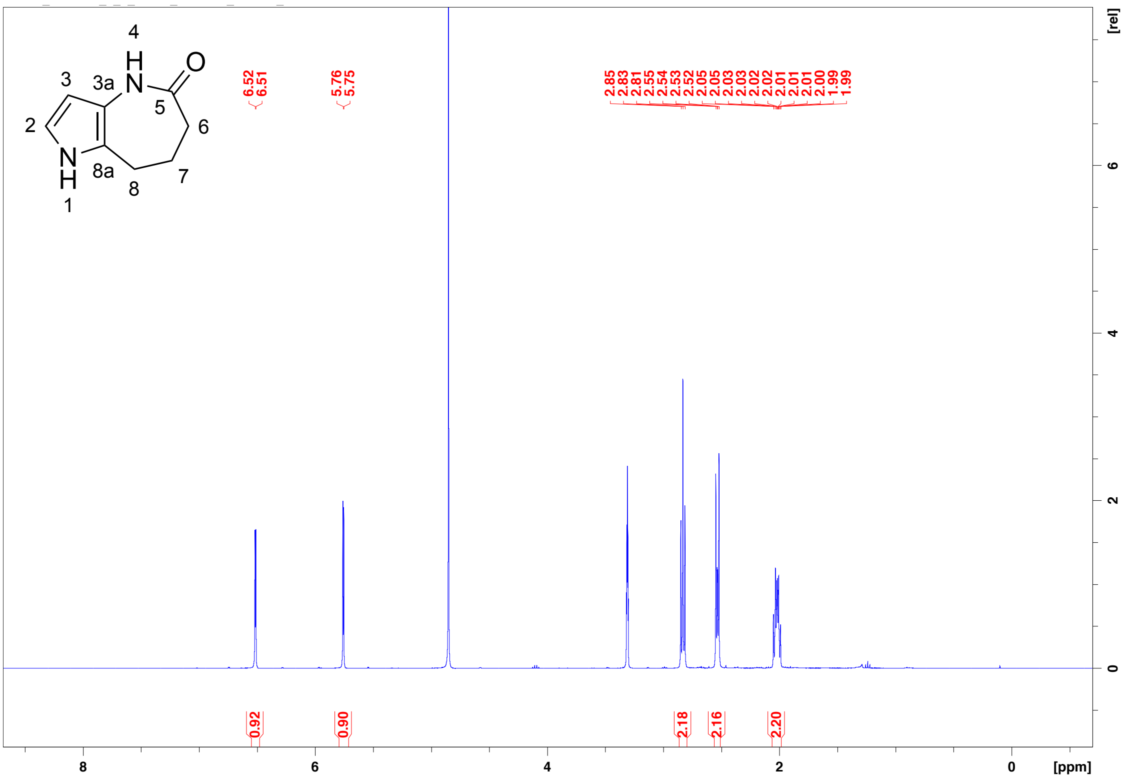

^{13}C-NMR

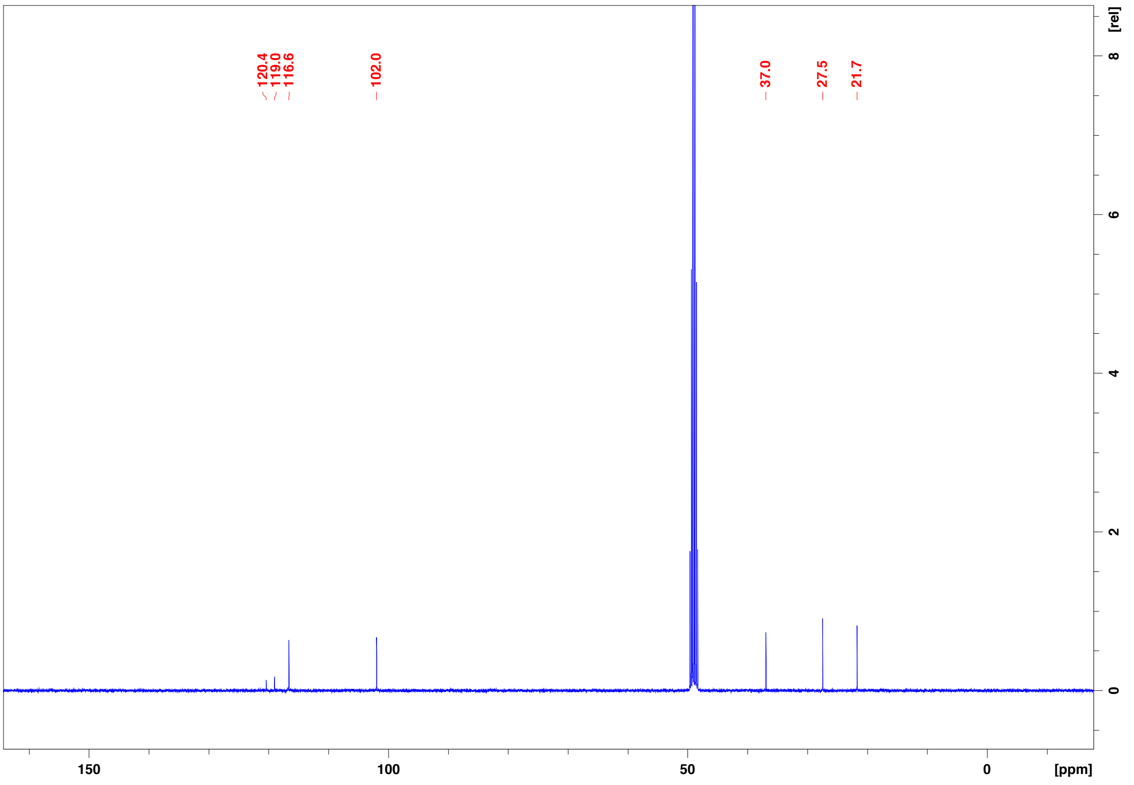

Octahydropyrrolo[3,2-b]azepin-5(1H)-one ((±)-**15**)

^{1}H-NMR

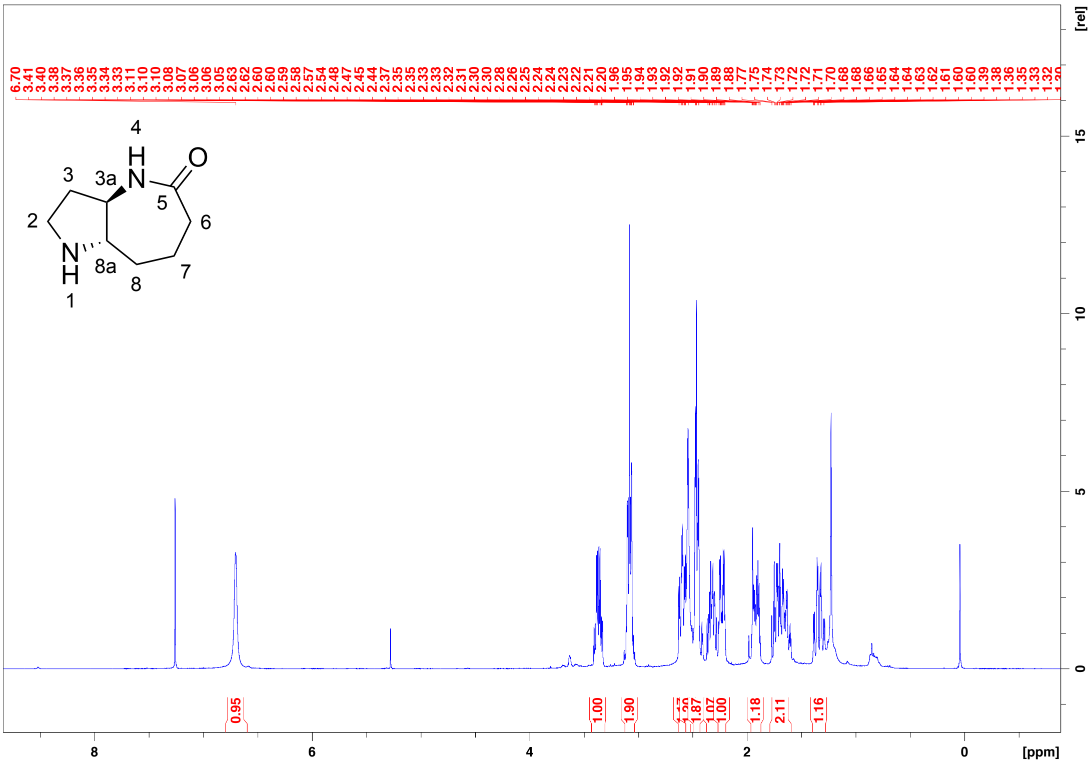

^{13}C-NMR

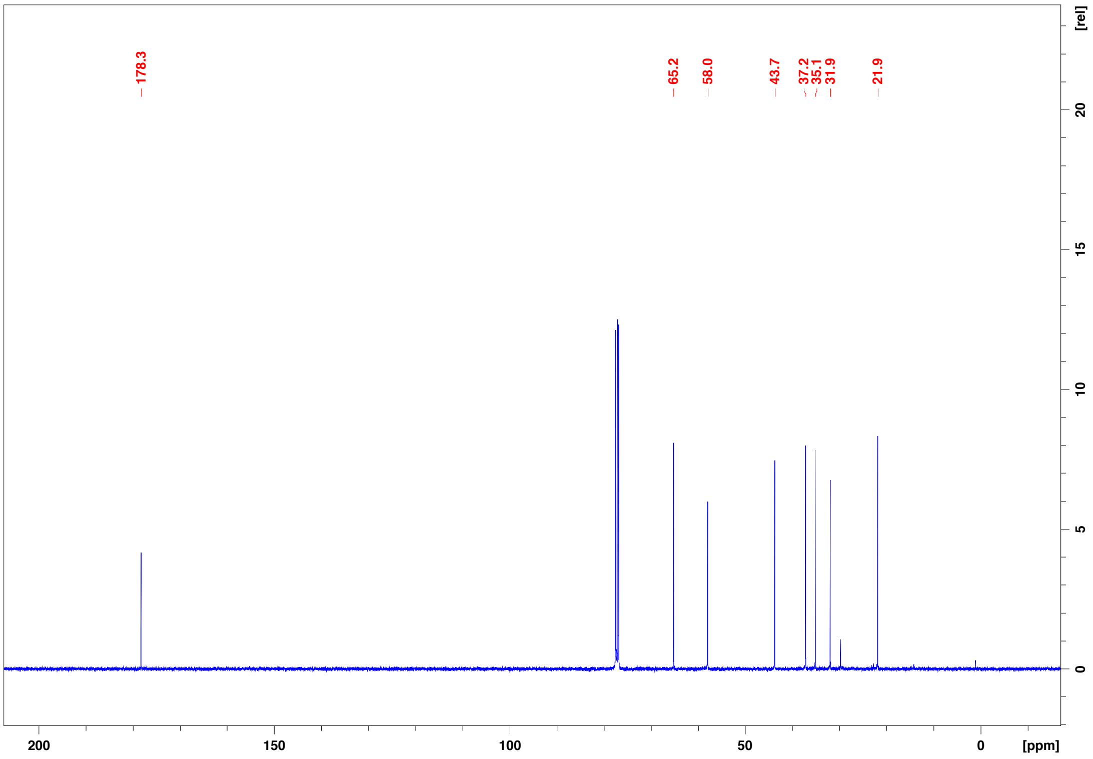

1-benzyloctahydropyrrolo[3,2-b]azepin-5(1H)-one ((±)-**16**)

^{1}H-NMR

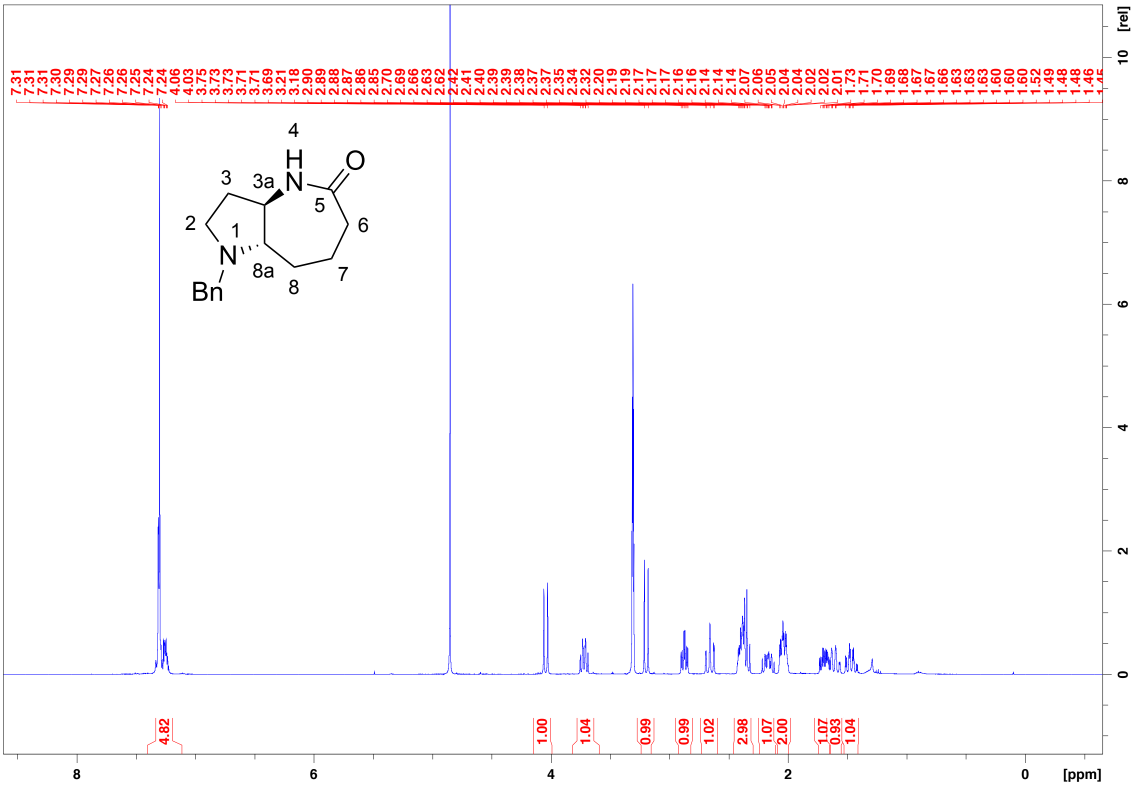

^{13}C-NMR

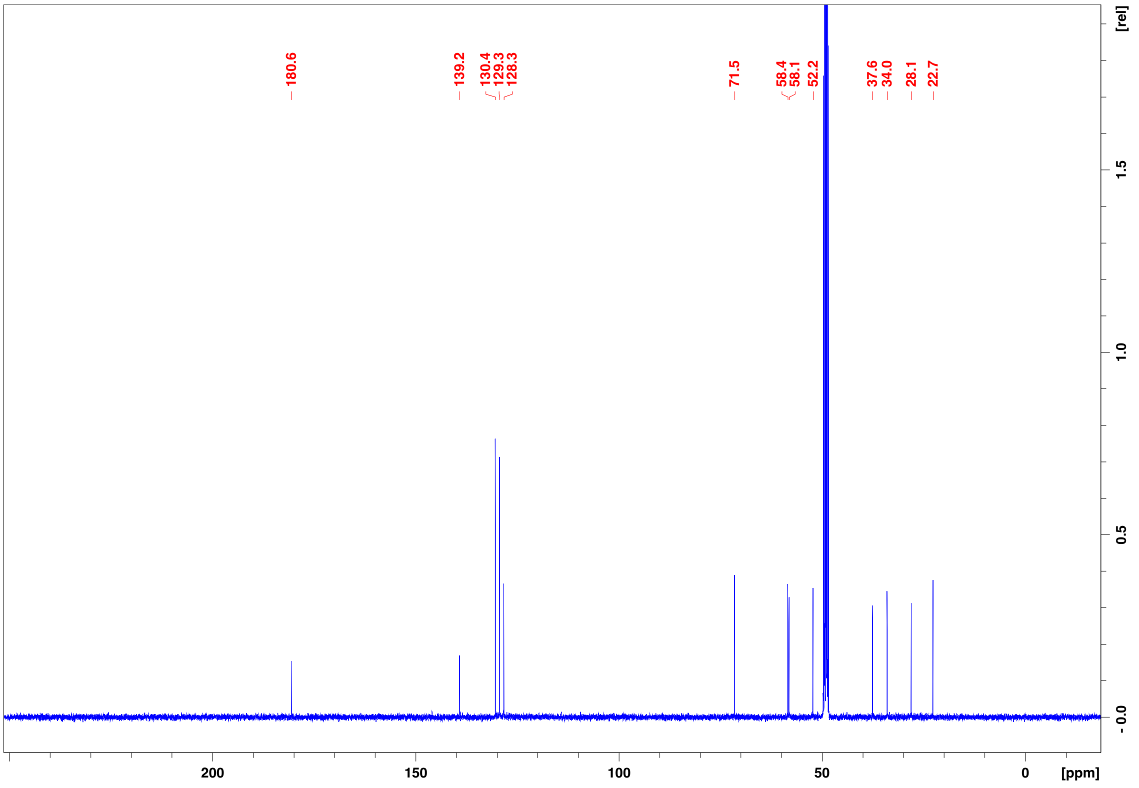

1-benzyldecahydropyrrolo[3,2-b]azepine ((±)-**17b**)

^{1}H-NMR

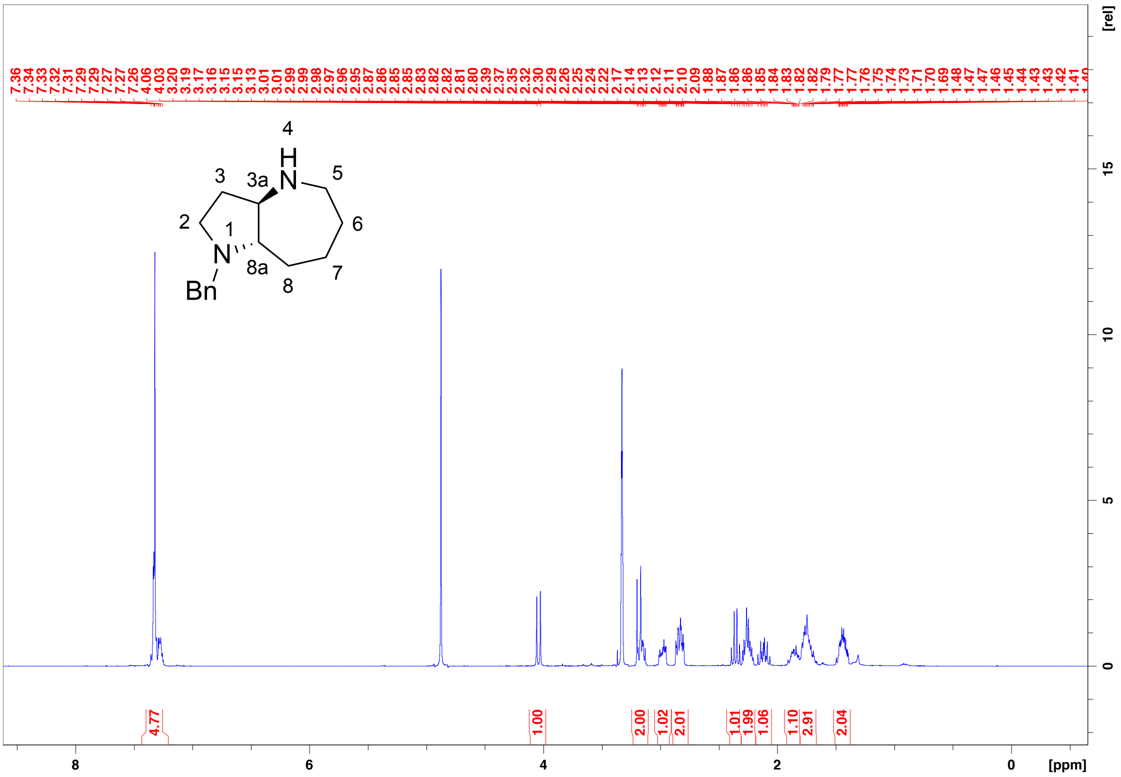

^{13}C-NMR

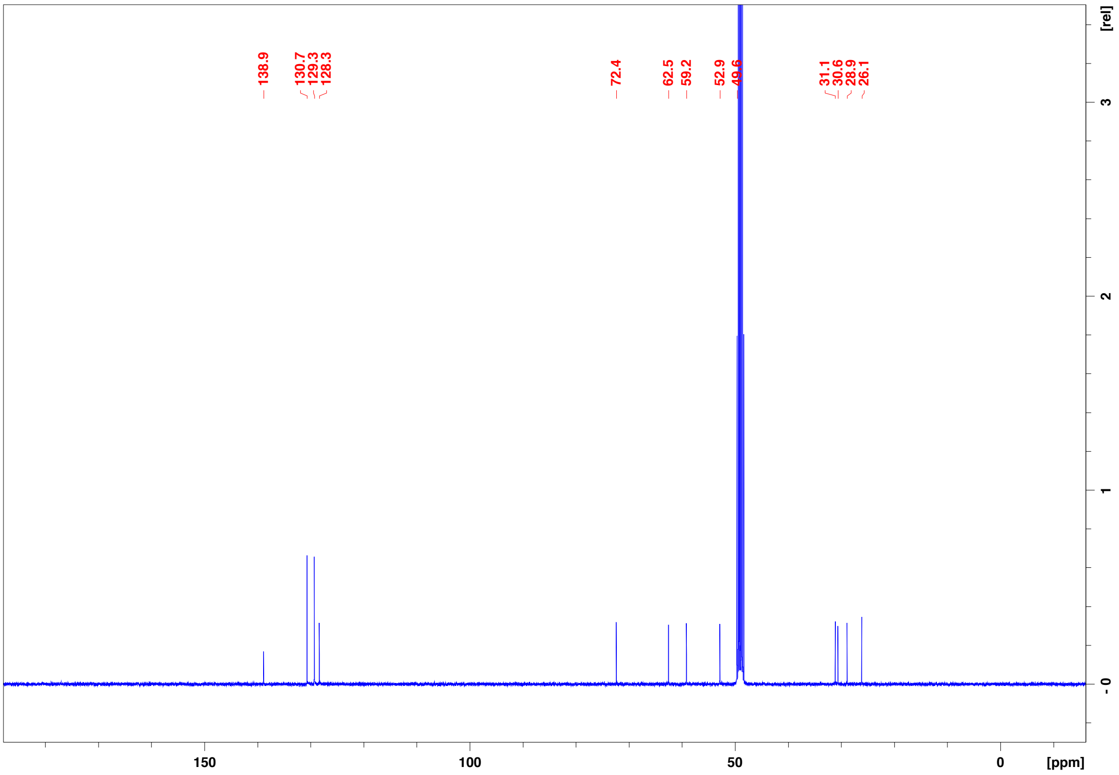

Decahydropyrrolo[3,2-b]azepine ((±)-**2b**)

^{1}H-NMR

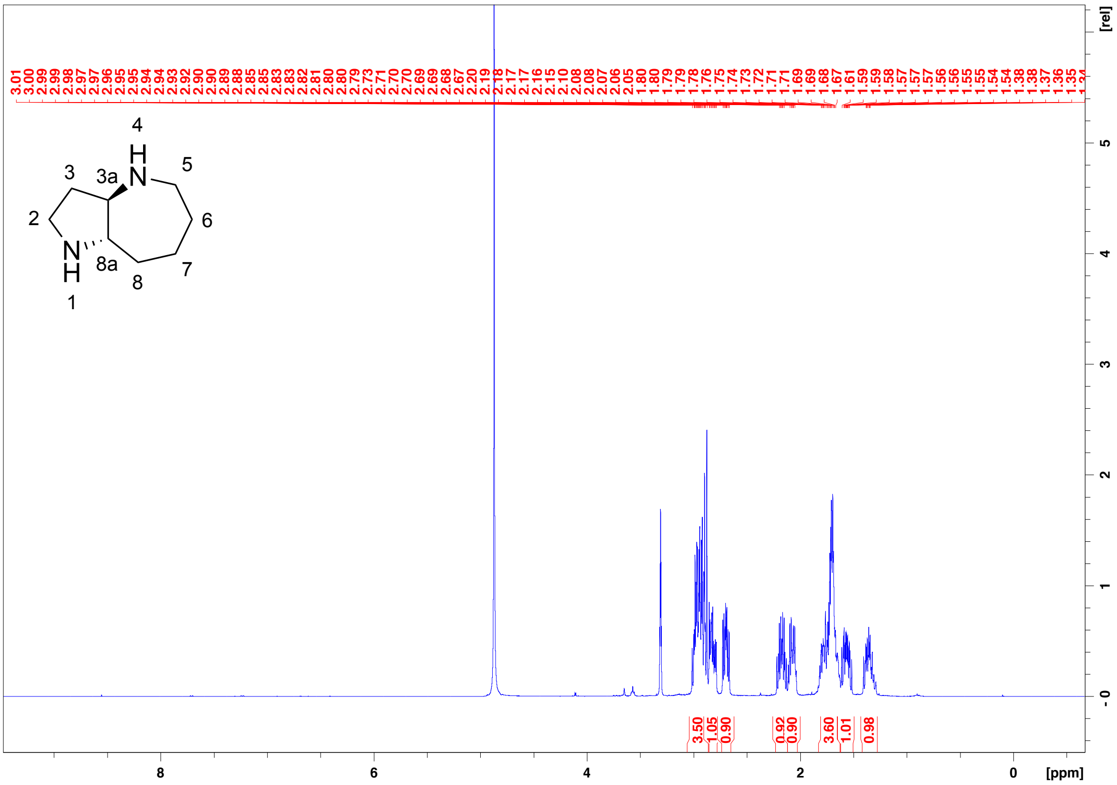

^{13}C-NMR

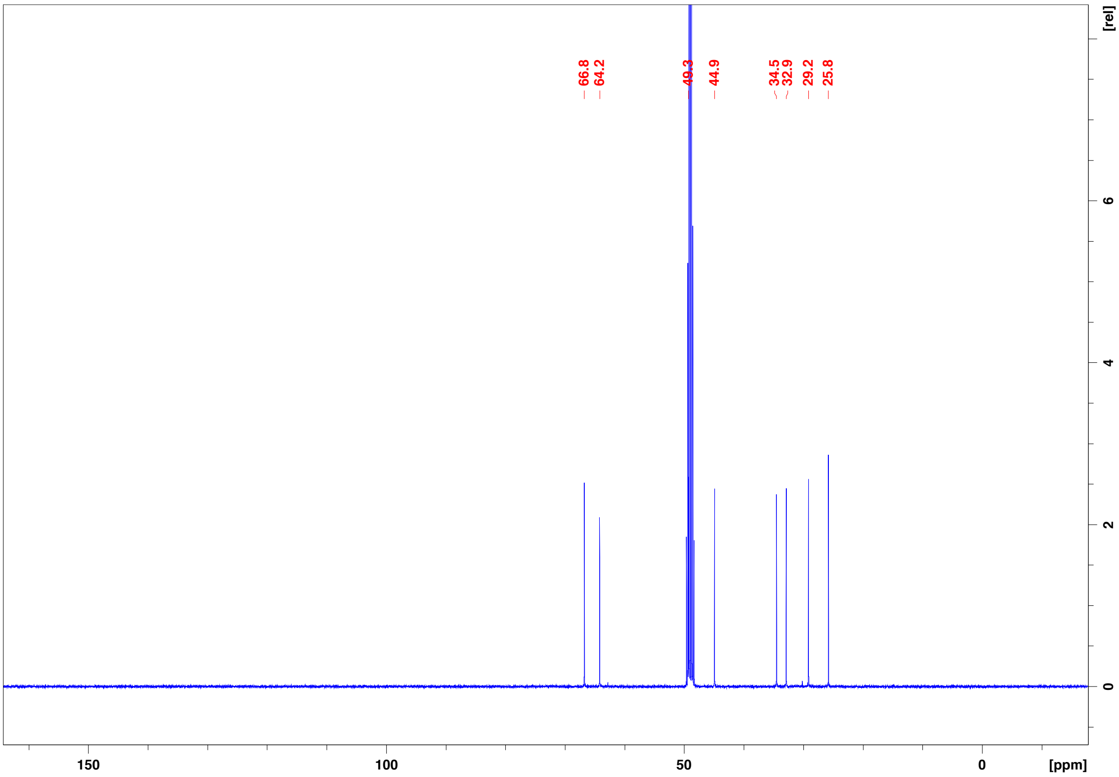

(*E*)-7,8-dihydroquinolin-5(6H)-one oxime (**19**)

^{1}H-NMR

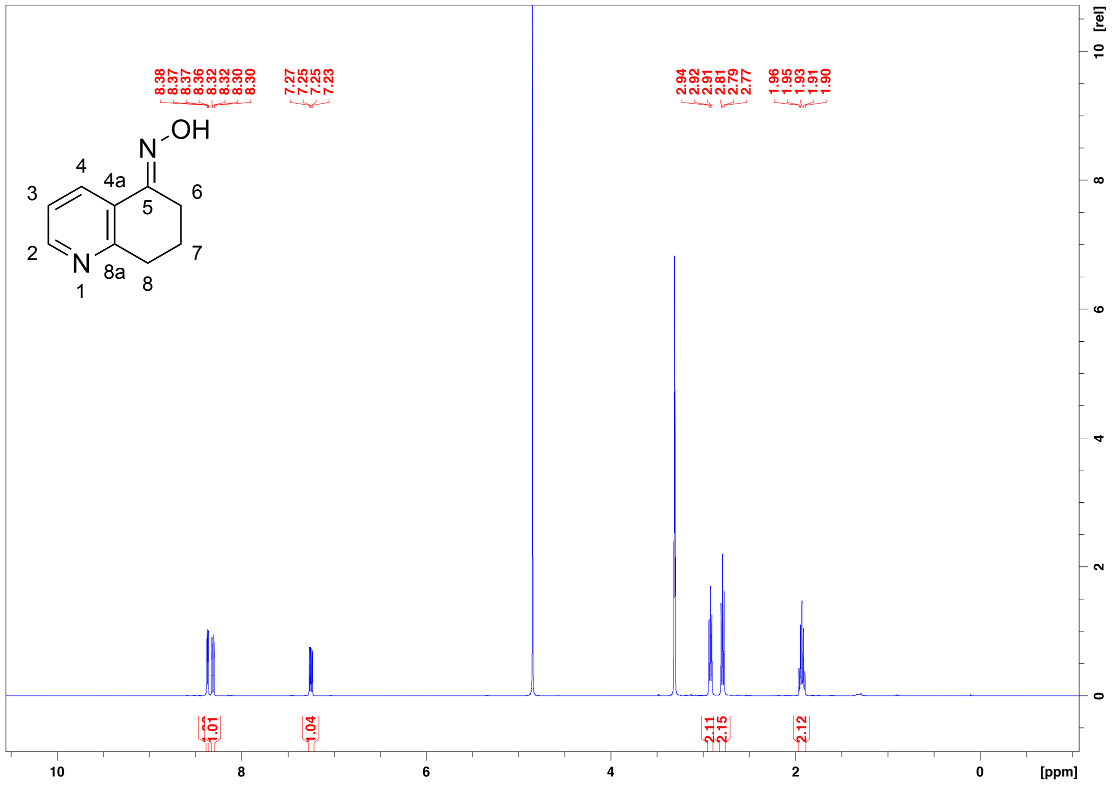

^{13}C-NMR

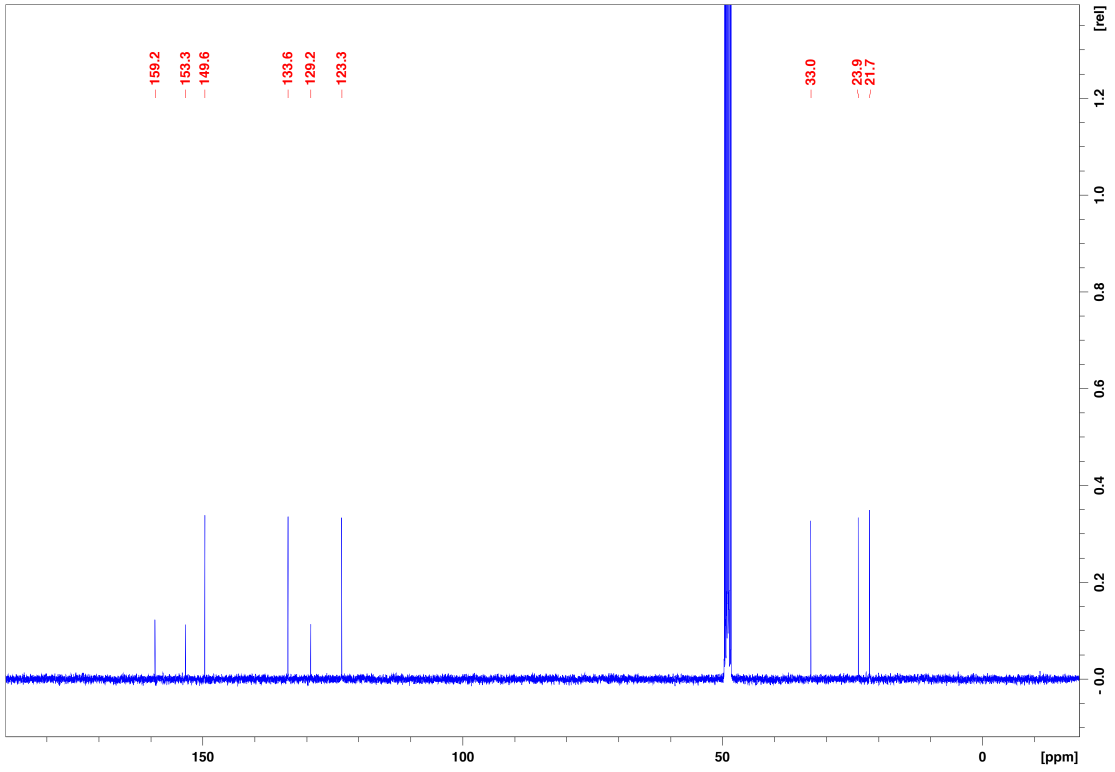

(*E*)-7,8-dihydroquinolin-5(6H)-one *O*-tosyl oxime (**20**)

^{1}H-NMR

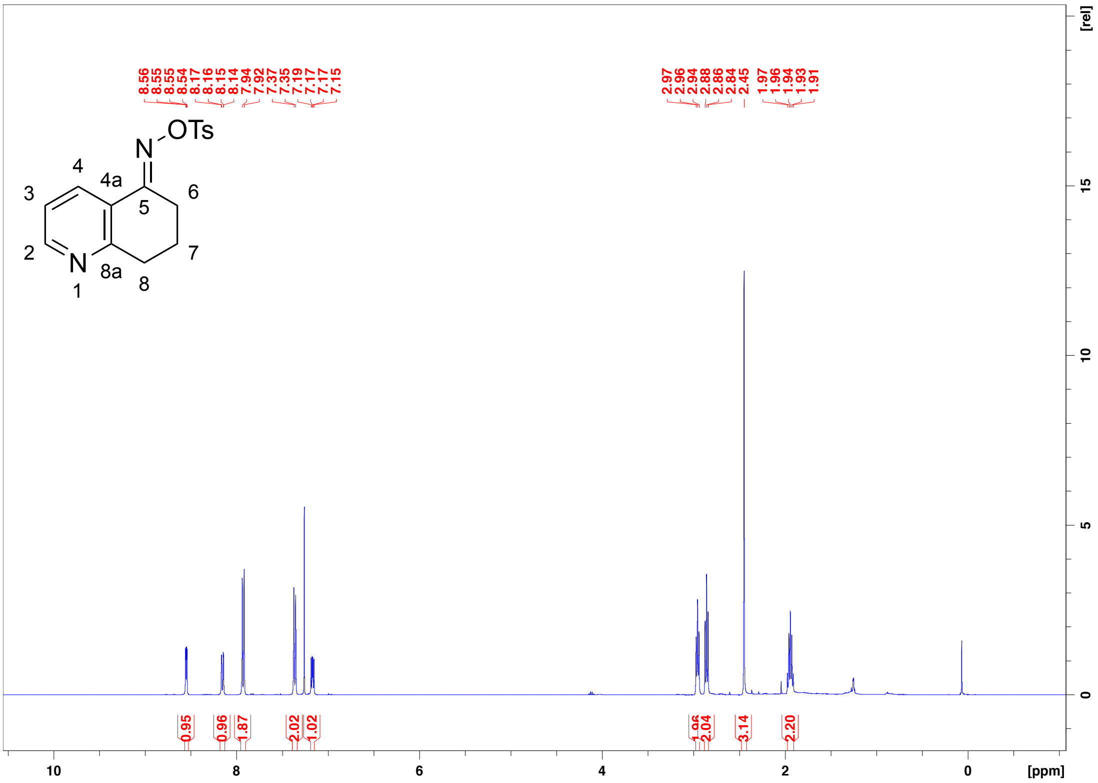

^{13}C-NMR

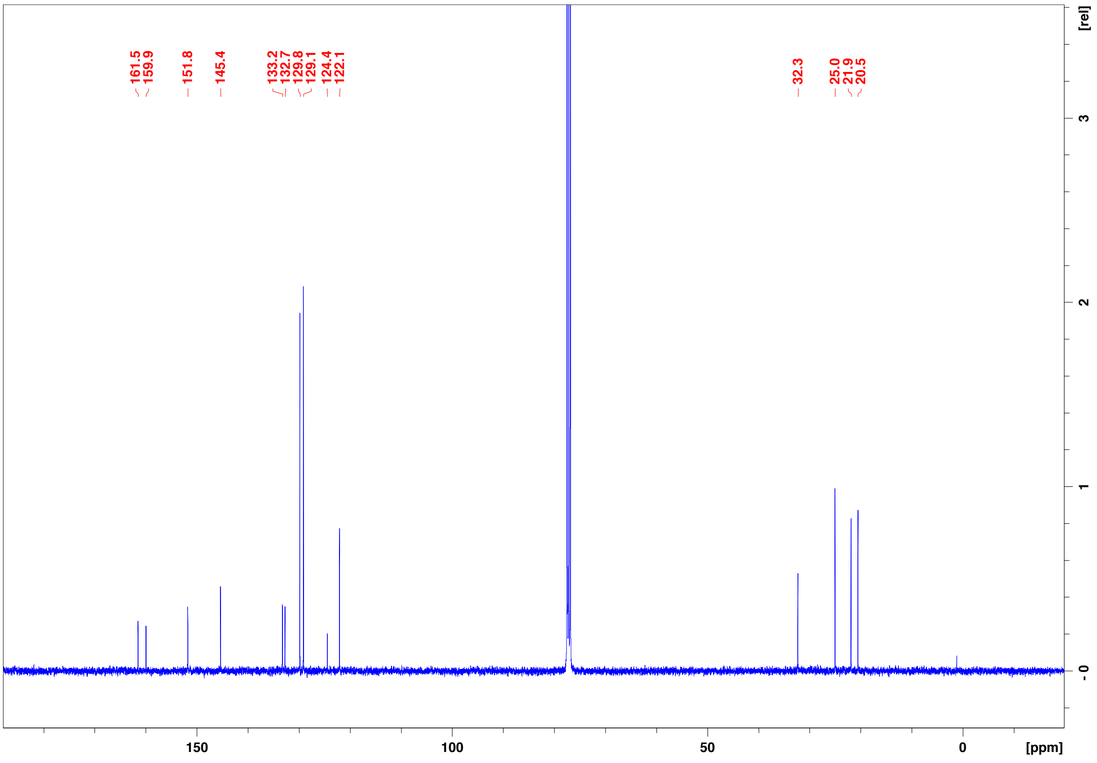

5,7,8,9-tetrahydro-6H-pyrido[3,2-*b*]azepin-6-one (**21**)

^{1}H-NMR

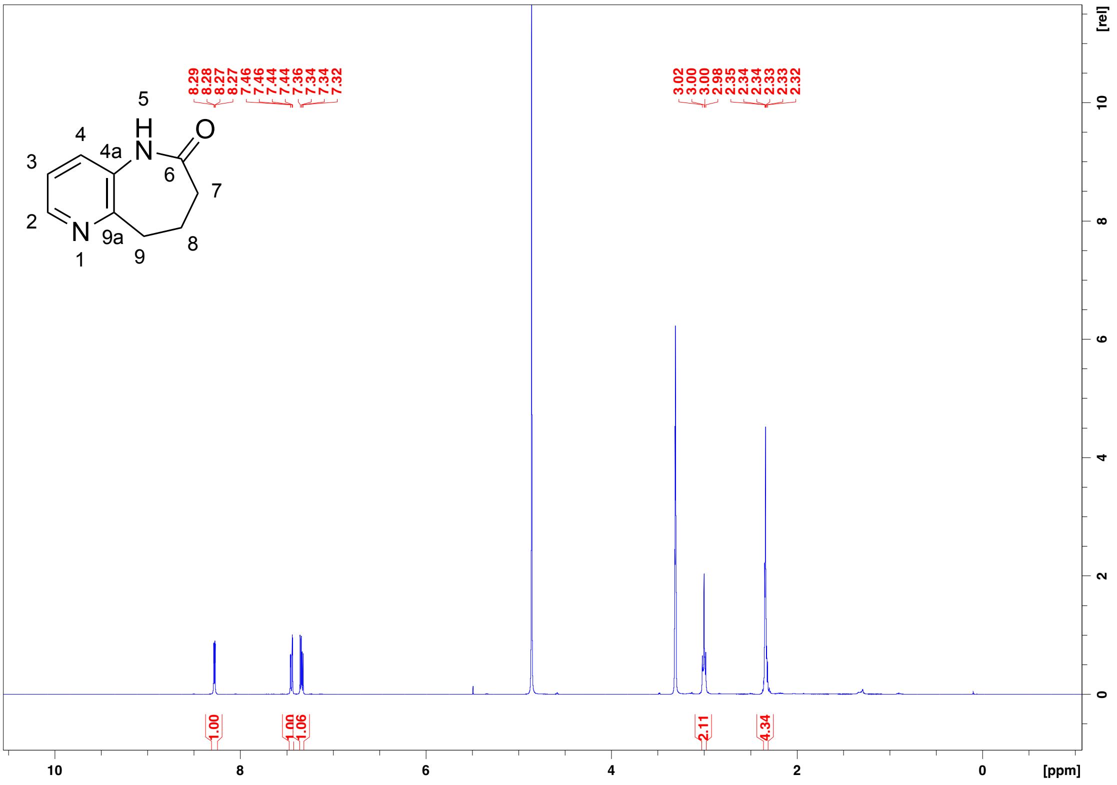

^{13}C-NMR

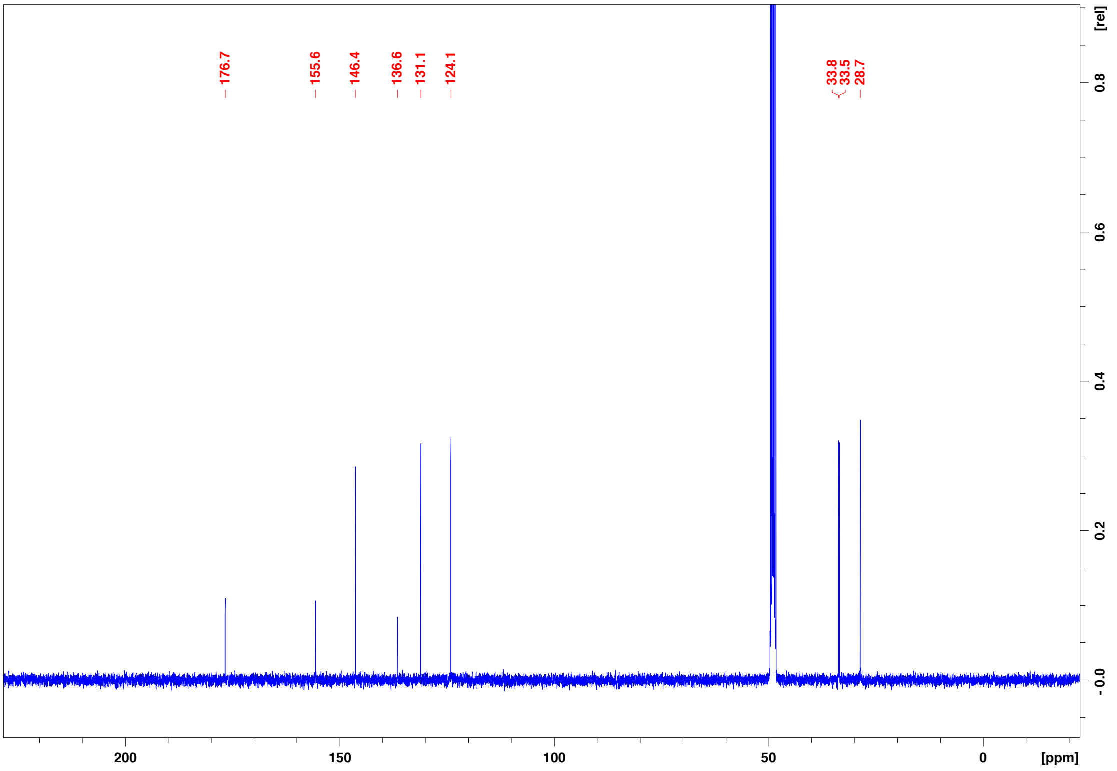

6,7,8,9-tetrahydro-5H-pyrido[3,2-*b*]azepine (**22**)

^{1}H-NMR

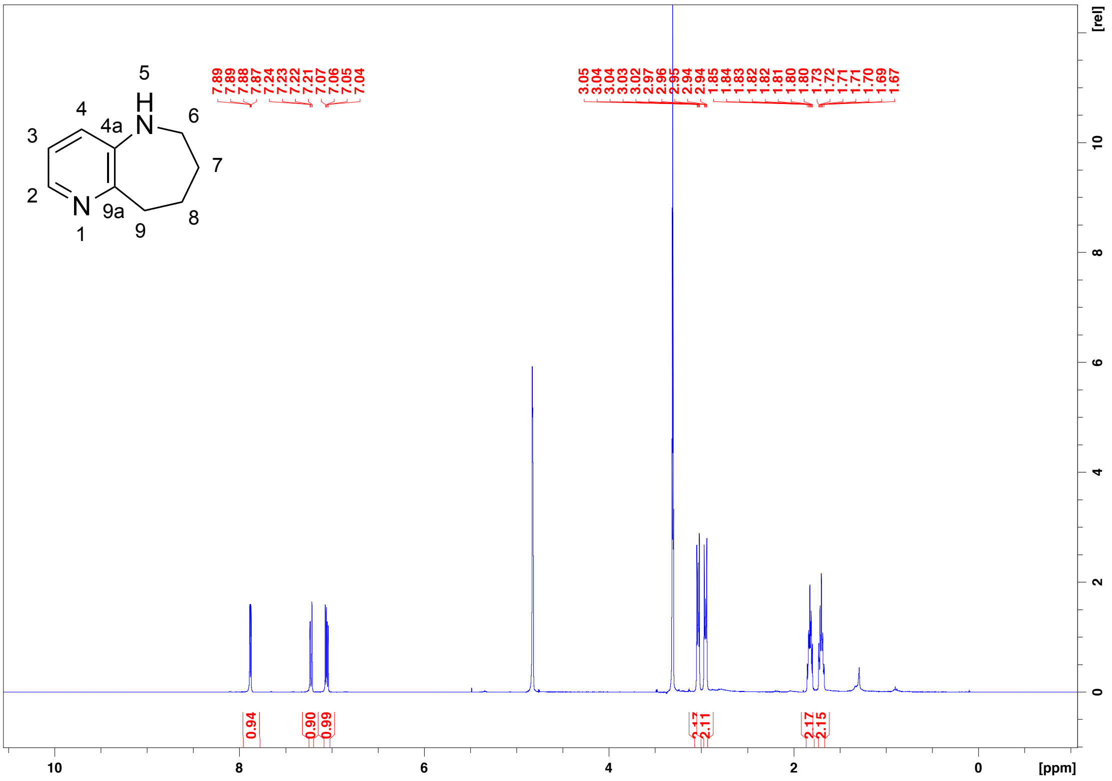

^{13}C-NMR

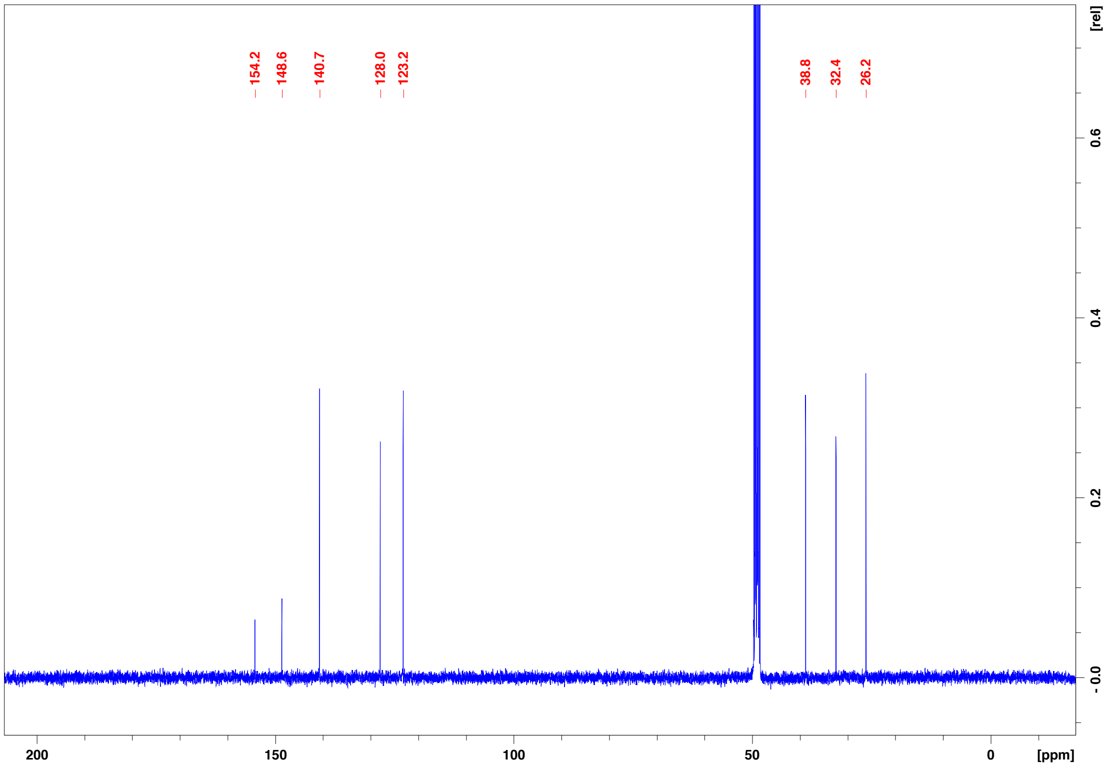

trifluoro-1-(6,7,8,9-tetrahydro-5H-pyrido[3,2-b]azepin-5-yl)ethanone (**23**)

^{1}H-NMR

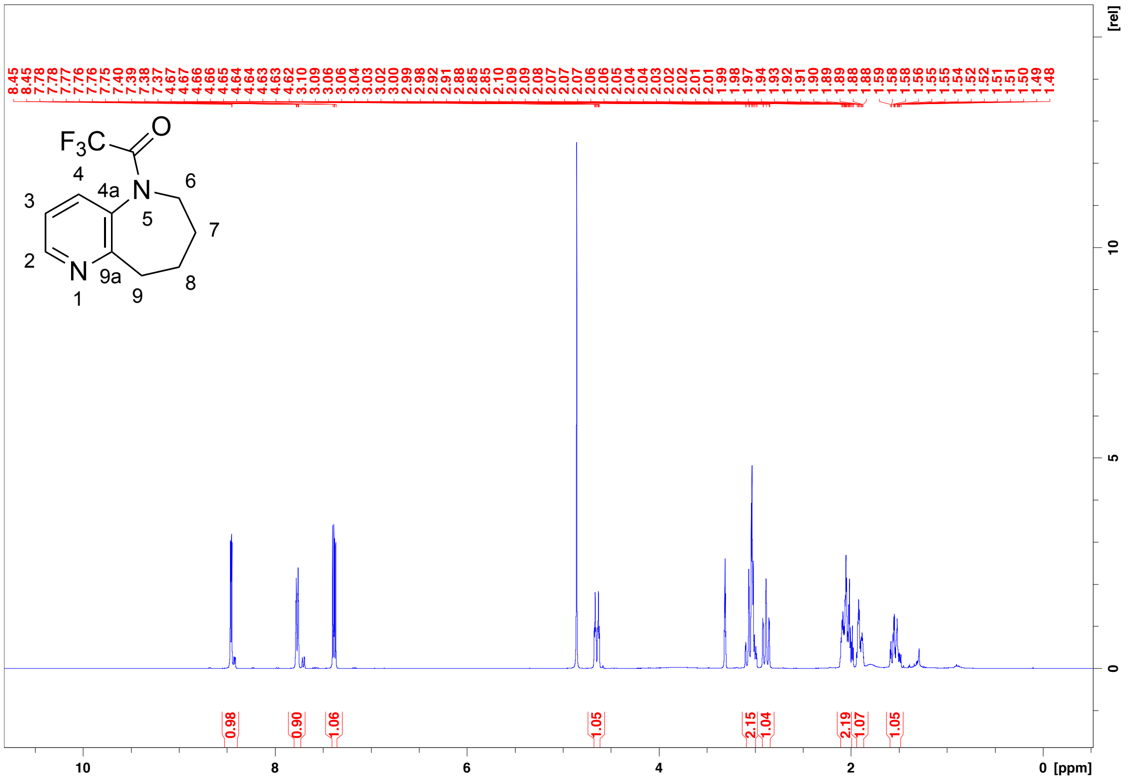

^{13}C-NMR

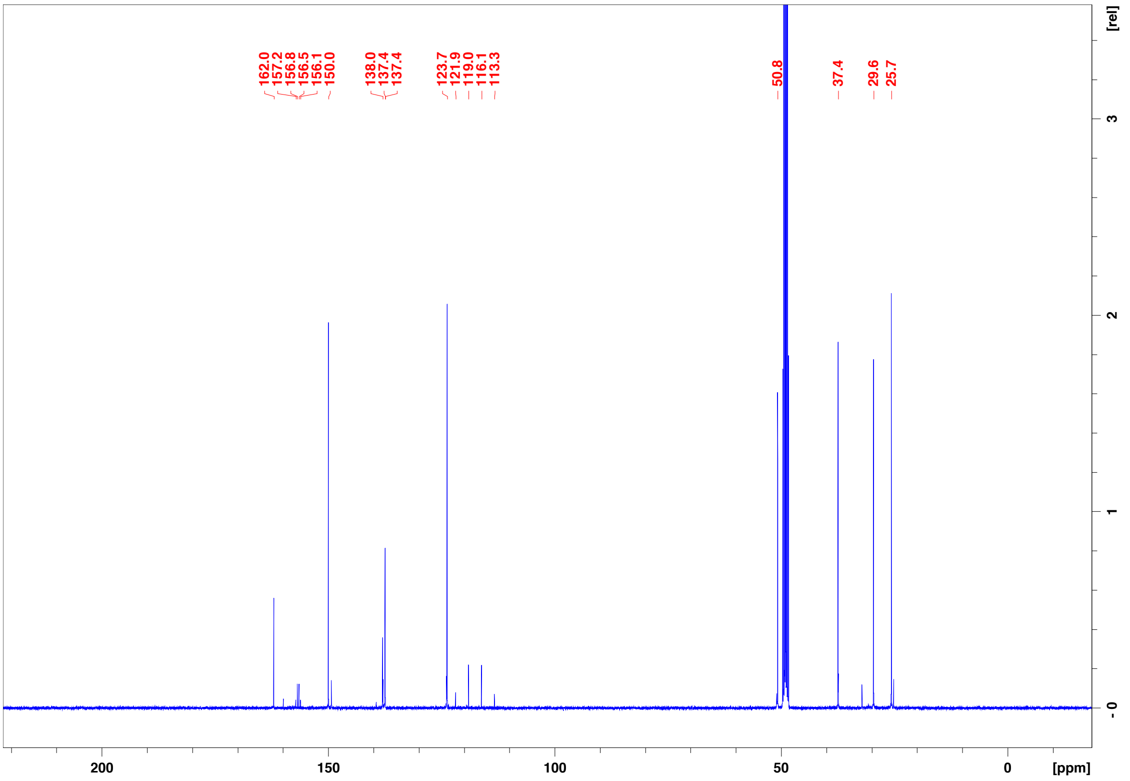

<u>1-(decahydro-5H-pyrido[3,2-b]azepin-5-yl)-trifluoroethanone ((±)-**24**)</u>

^{1}H-NMR

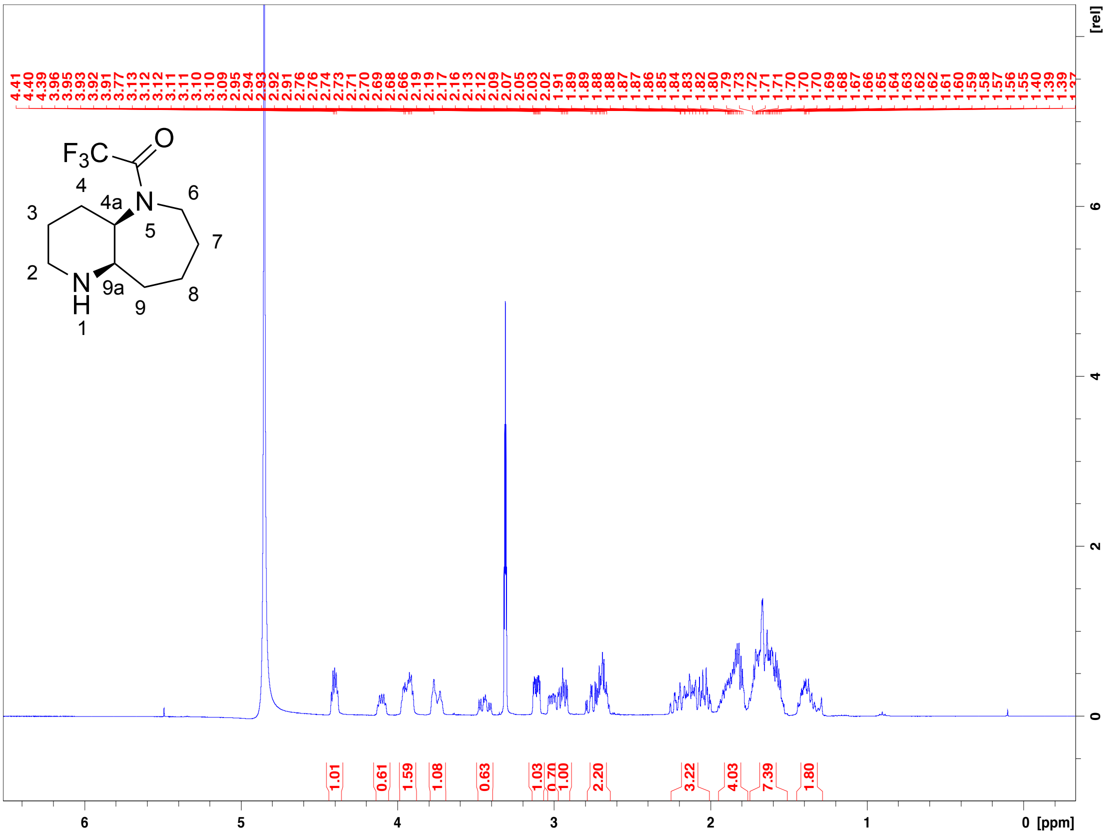

^{13}C-NMR

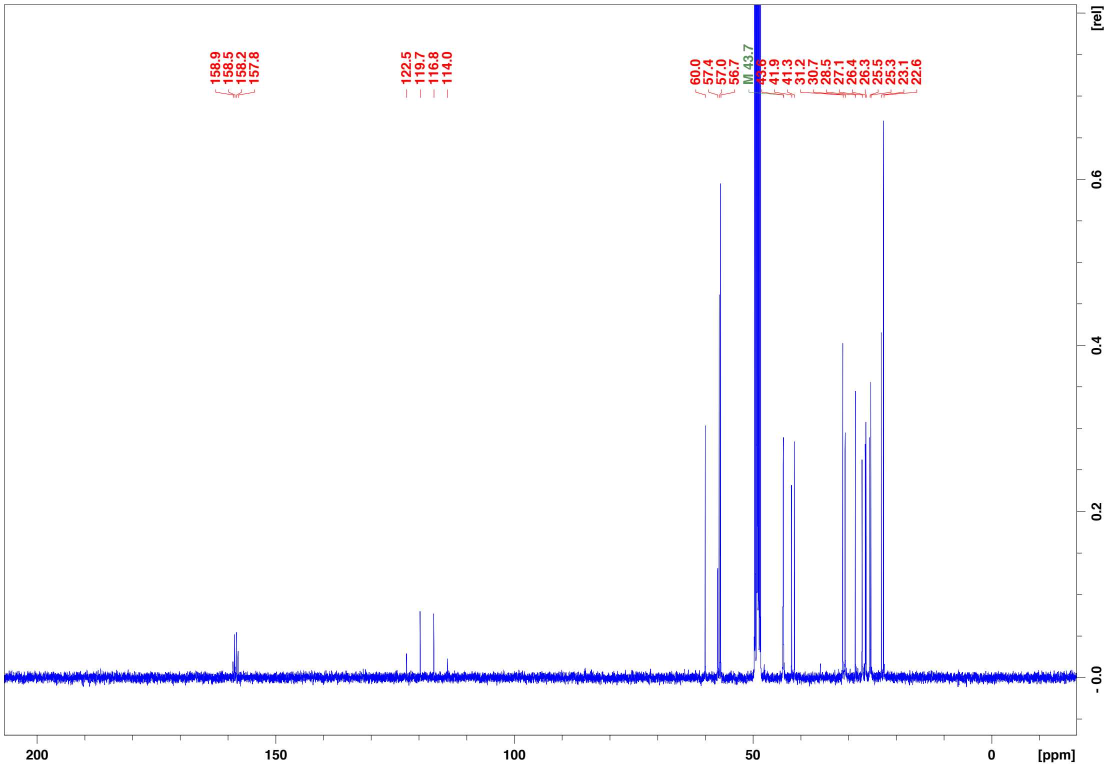

Decahydro-1H-pyrido[3,2-*b*]azepine ((±)-**3a**)

^{1}H-NMR

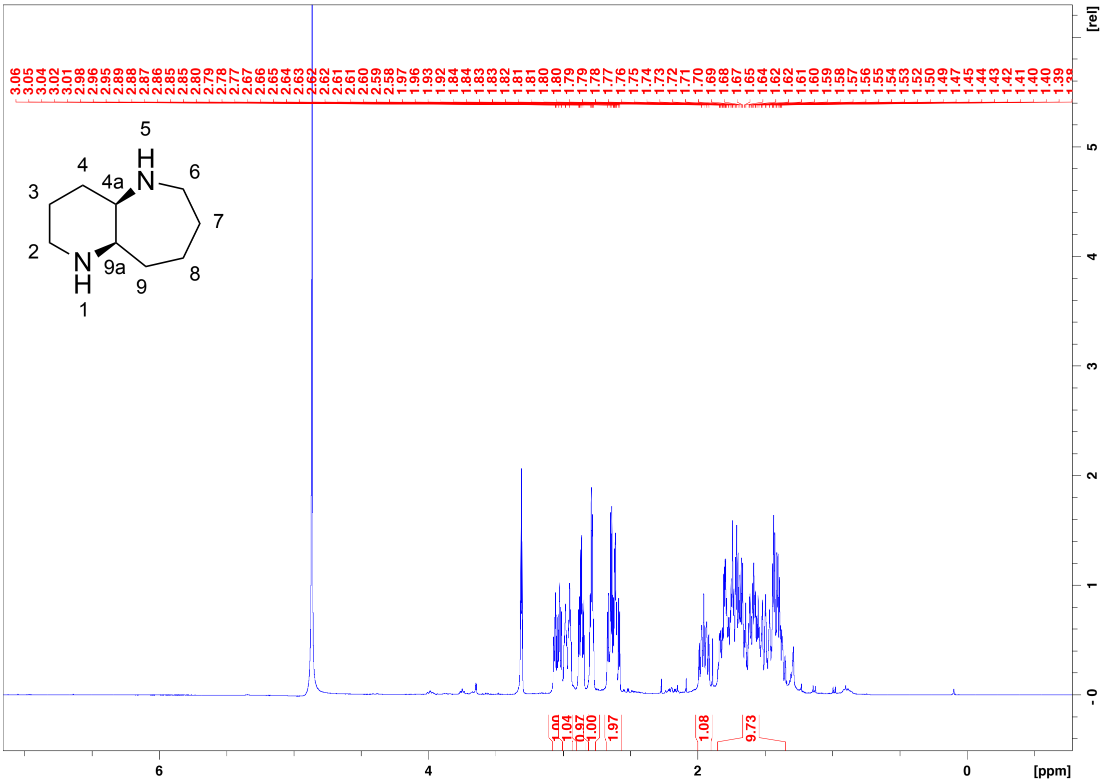

^{13}C-NMR

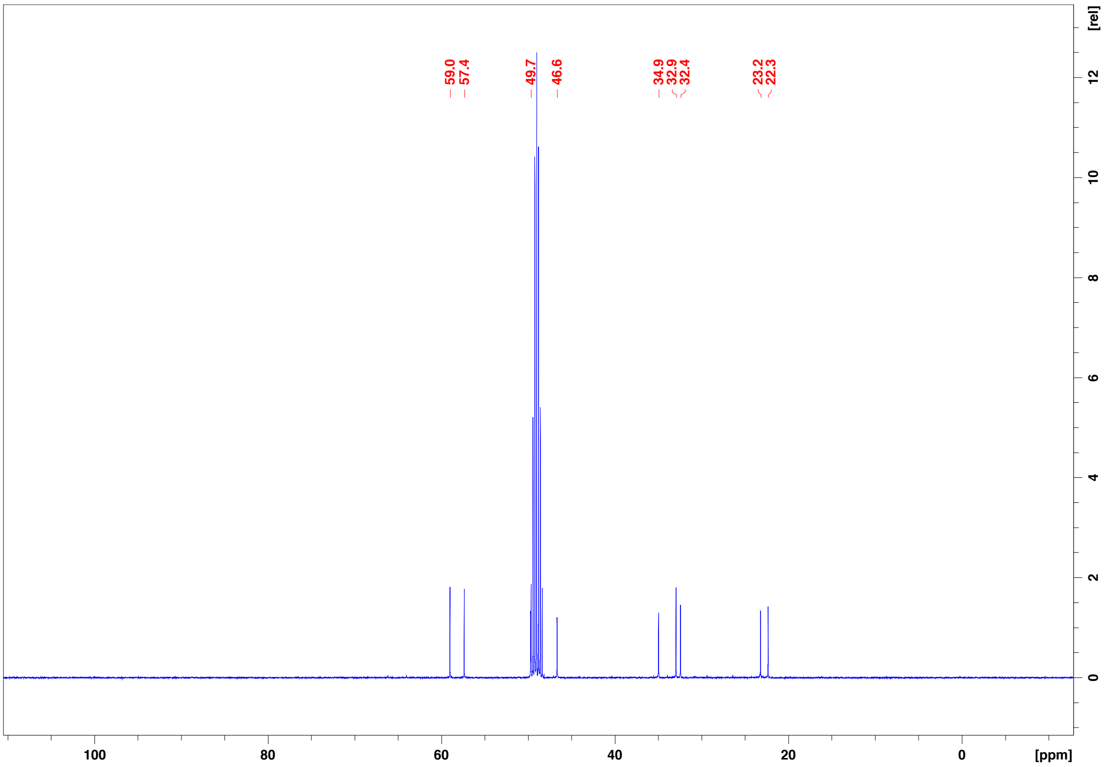

decahydro-6*H*-pyrido[3,2-b]azepin-6-one ((±)-**25**)

^{1}H-NMR

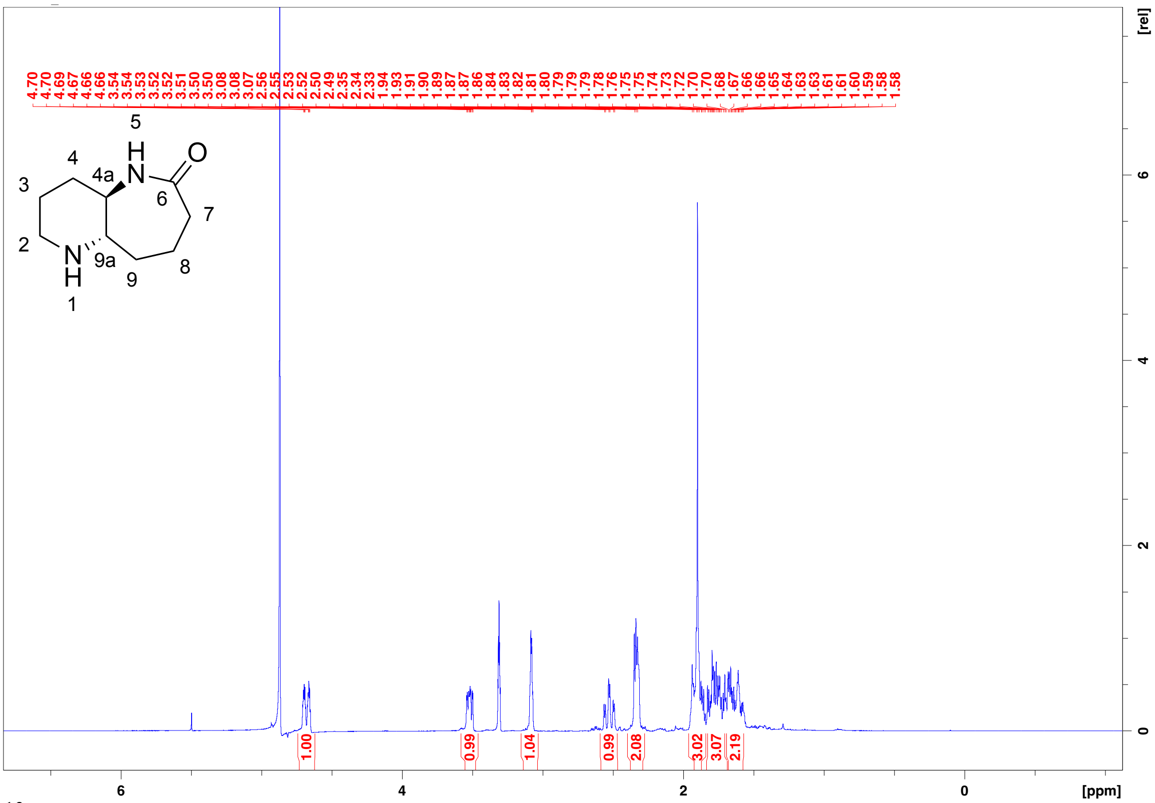

^{13}C-NMR

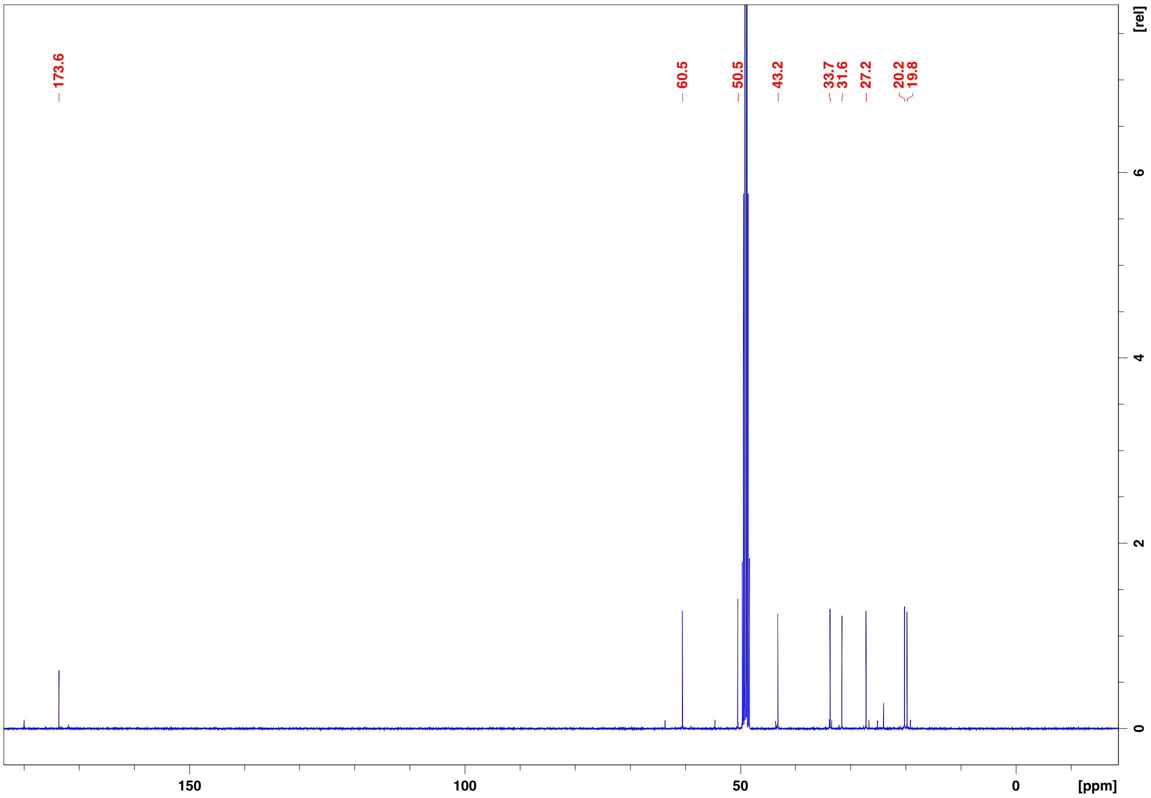

1-benzyldecahydro-6*H*-pyrido[3,2-b]azepin-6-one ((±)-**26**)

^{1}H-NMR

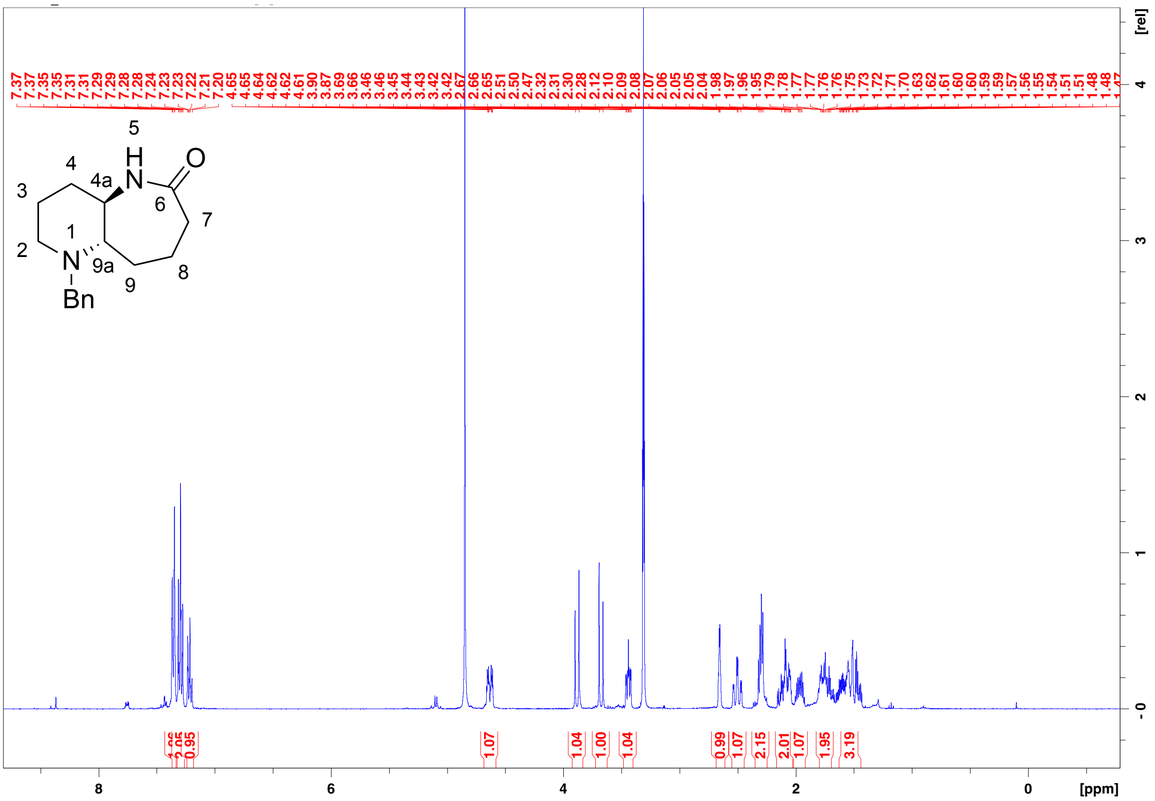

^{13}C-NMR

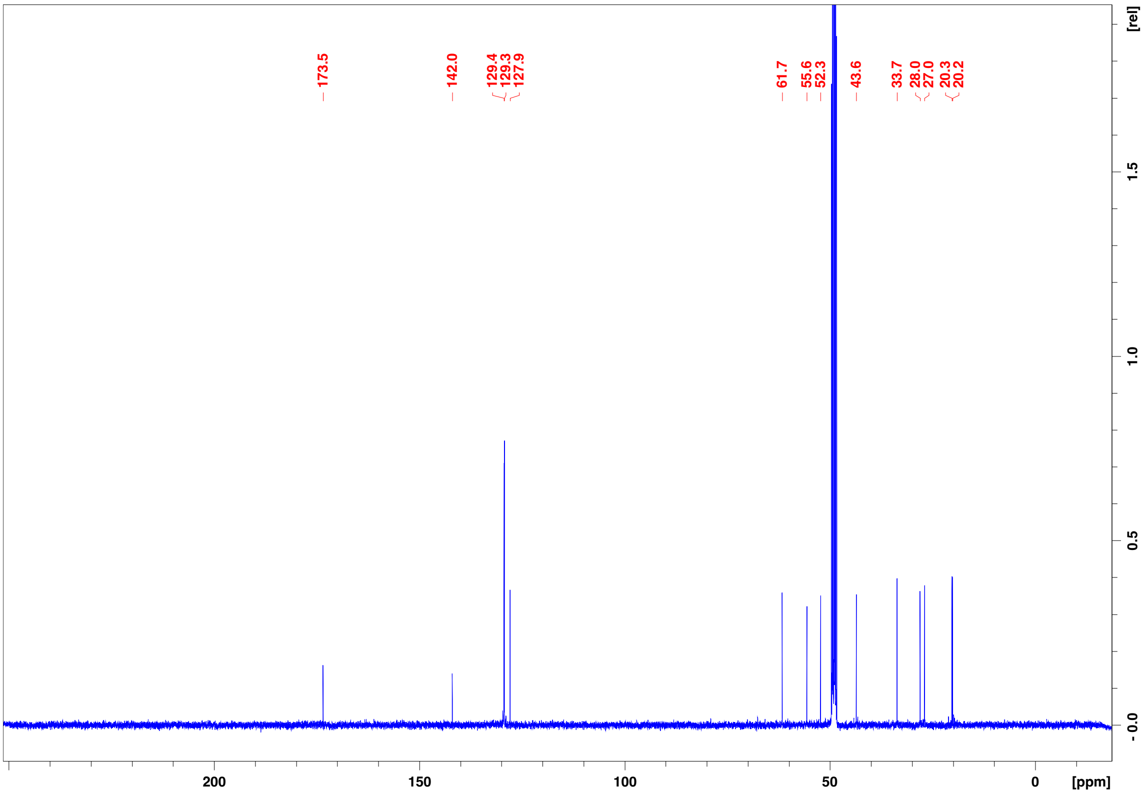

1-benzyldecahydro-1*H*-pyrido[3,2-b]azepine ((±)-**27b**)

^{1}H-NMR

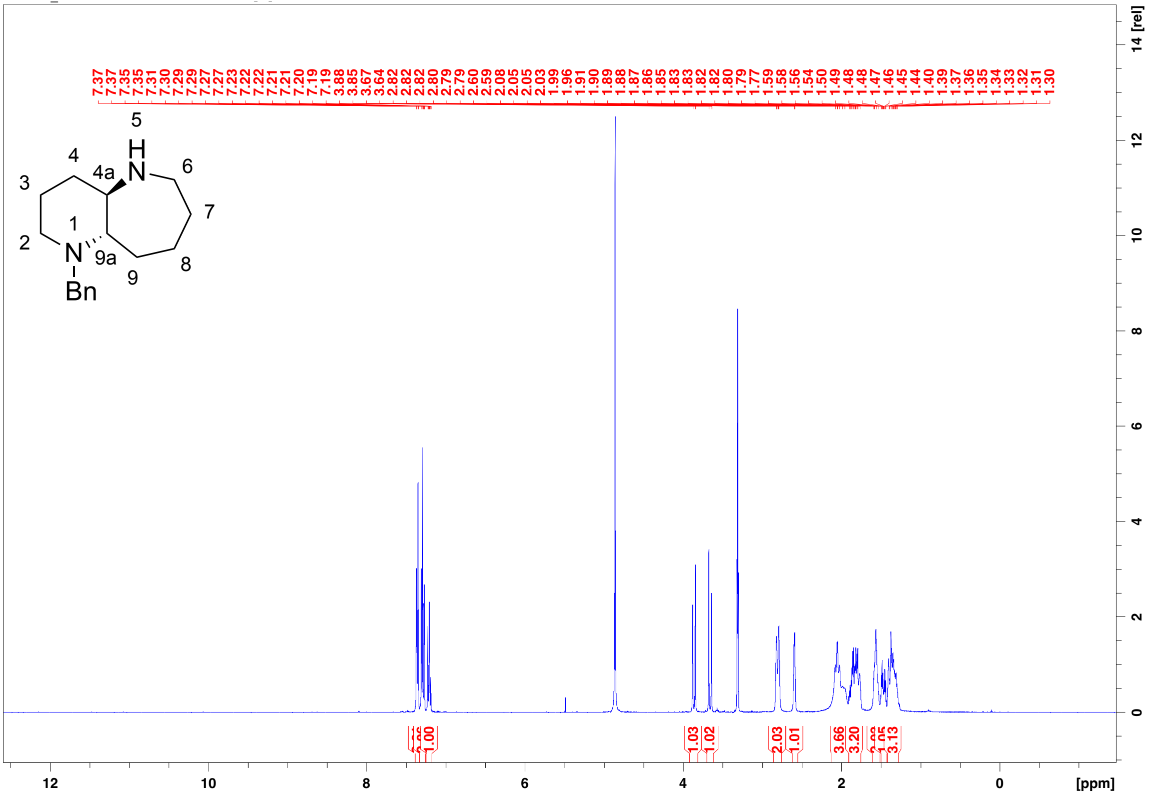

^{13}C-NMR

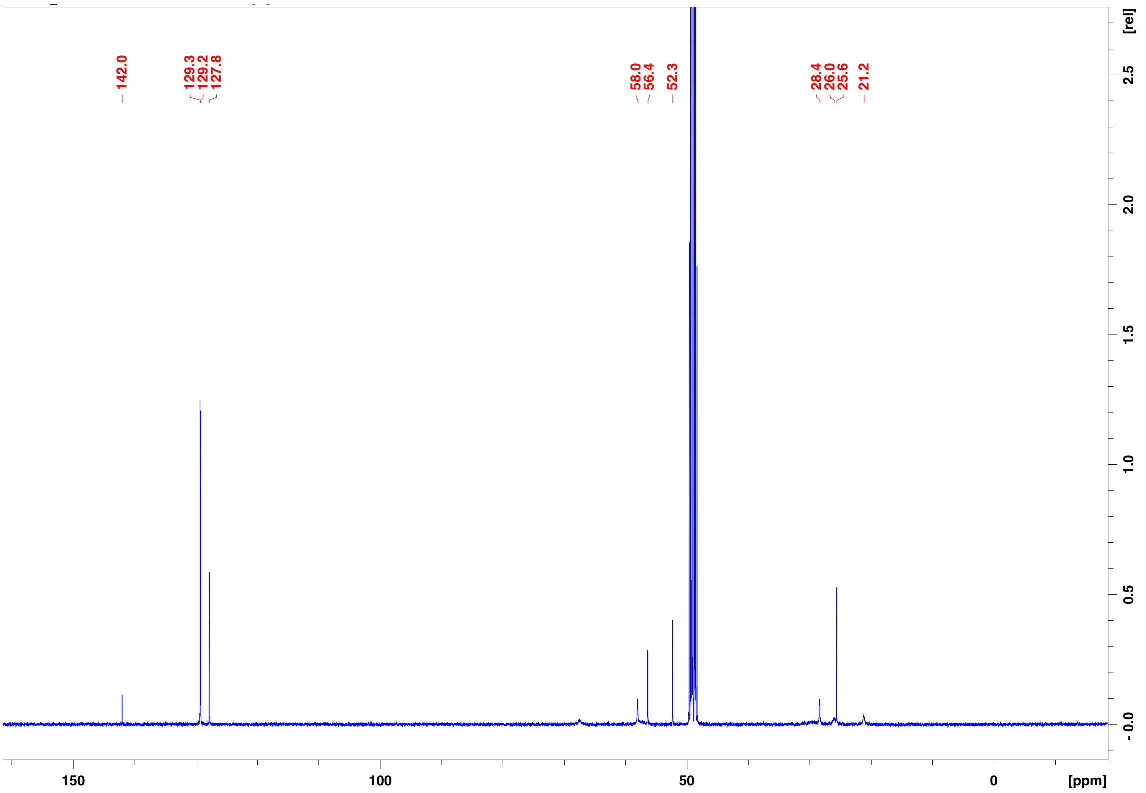

Decahydro-1H-pyrido[3,2-b]azepine ((±)-**3b**)

^{1}H-NMR

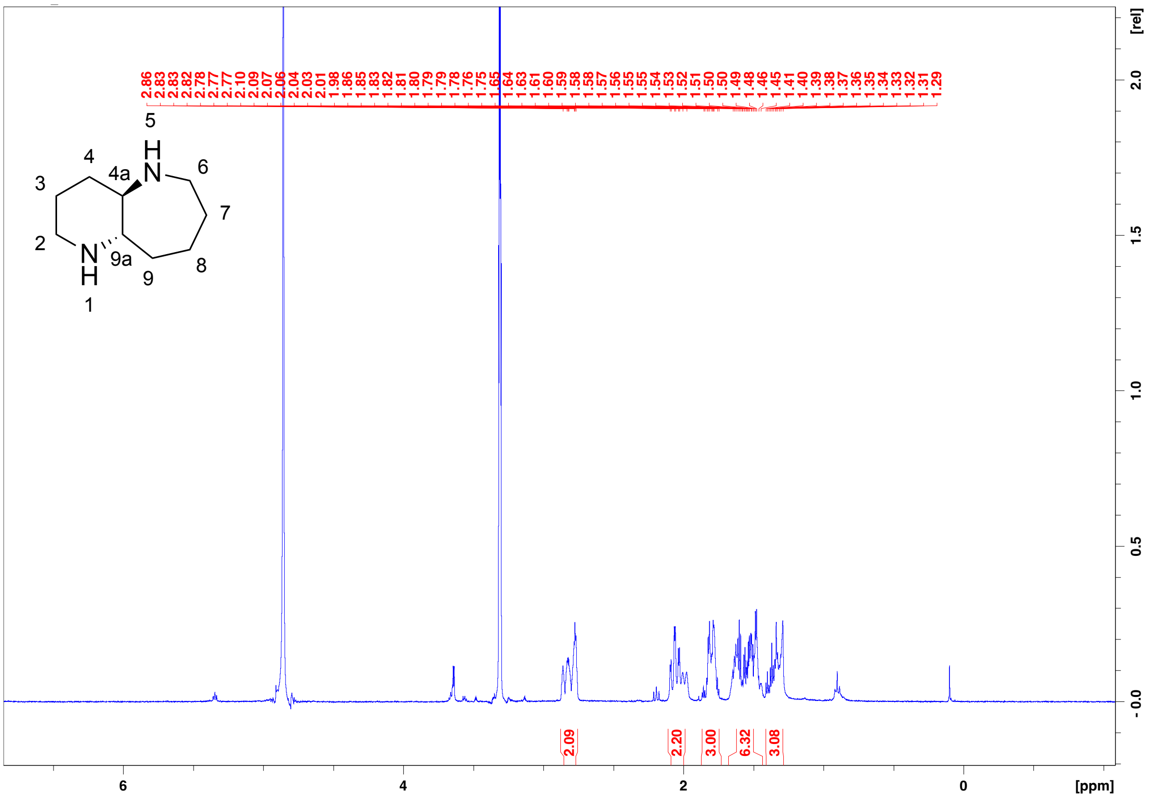

^{13}C-NMR

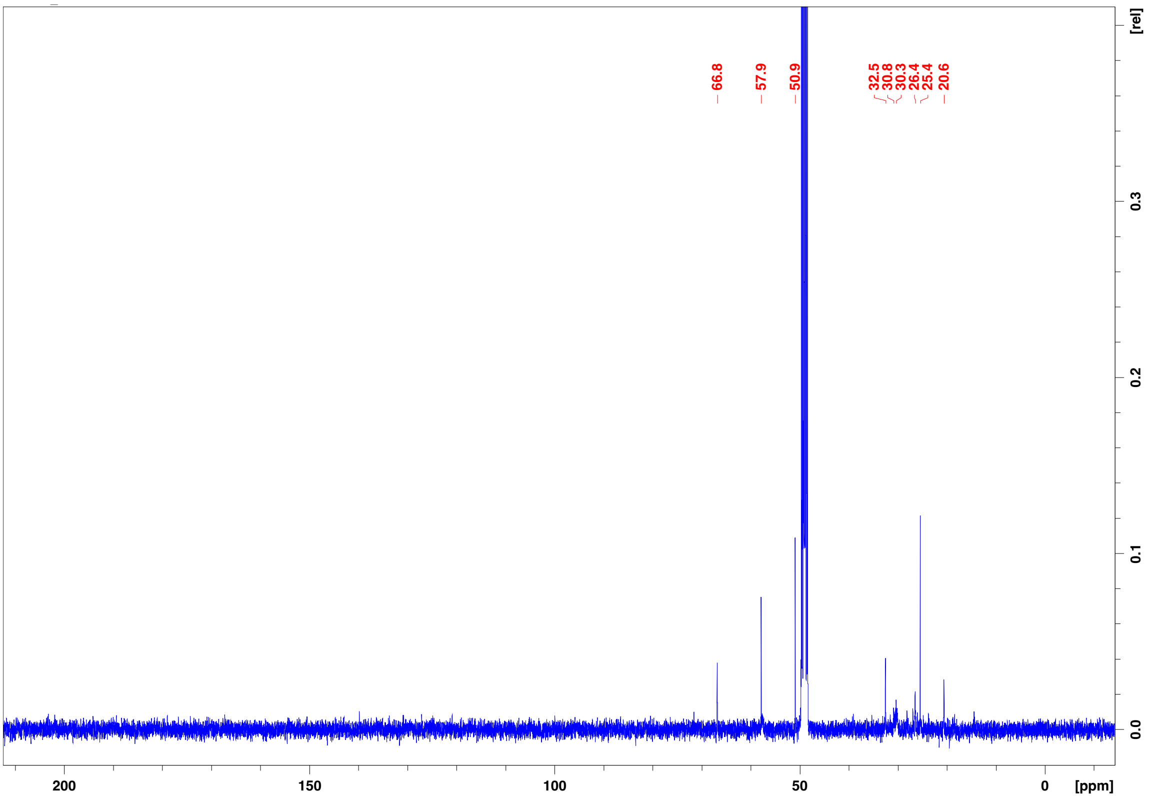

(*E*)-5-methoxy-3,4-dihydronaphthalen-1(2H)-one oxime (**29**)

^{1}H-NMR

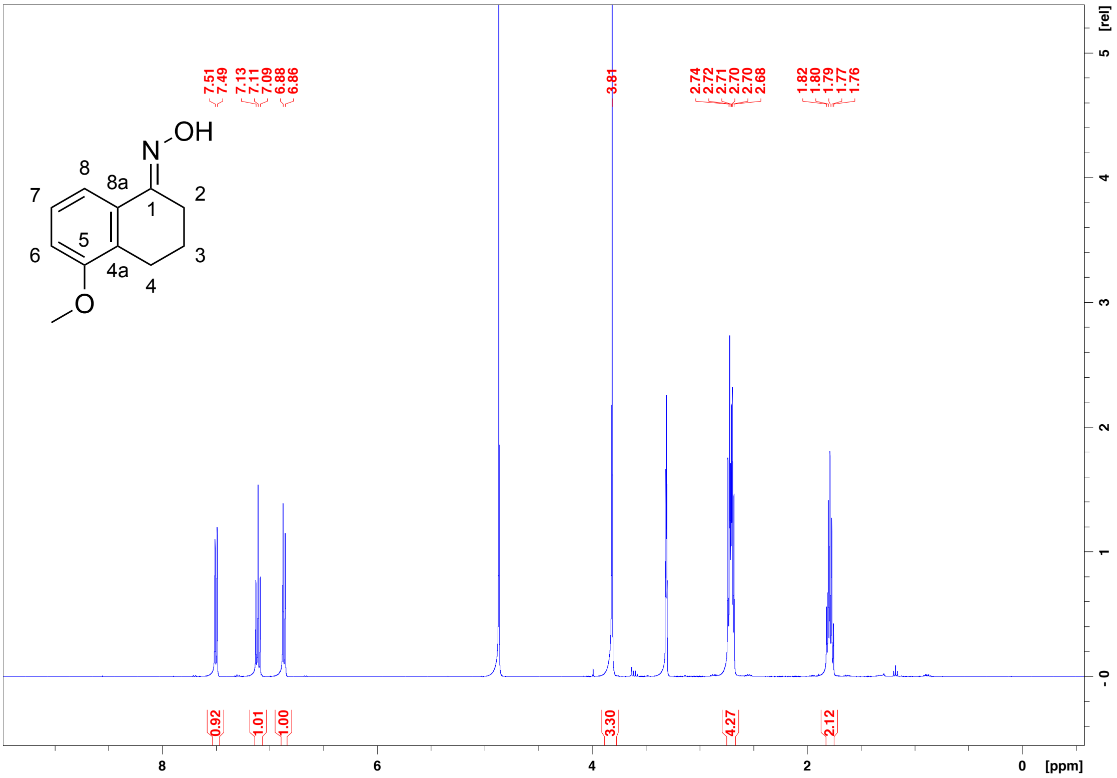

^{13}C-NMR

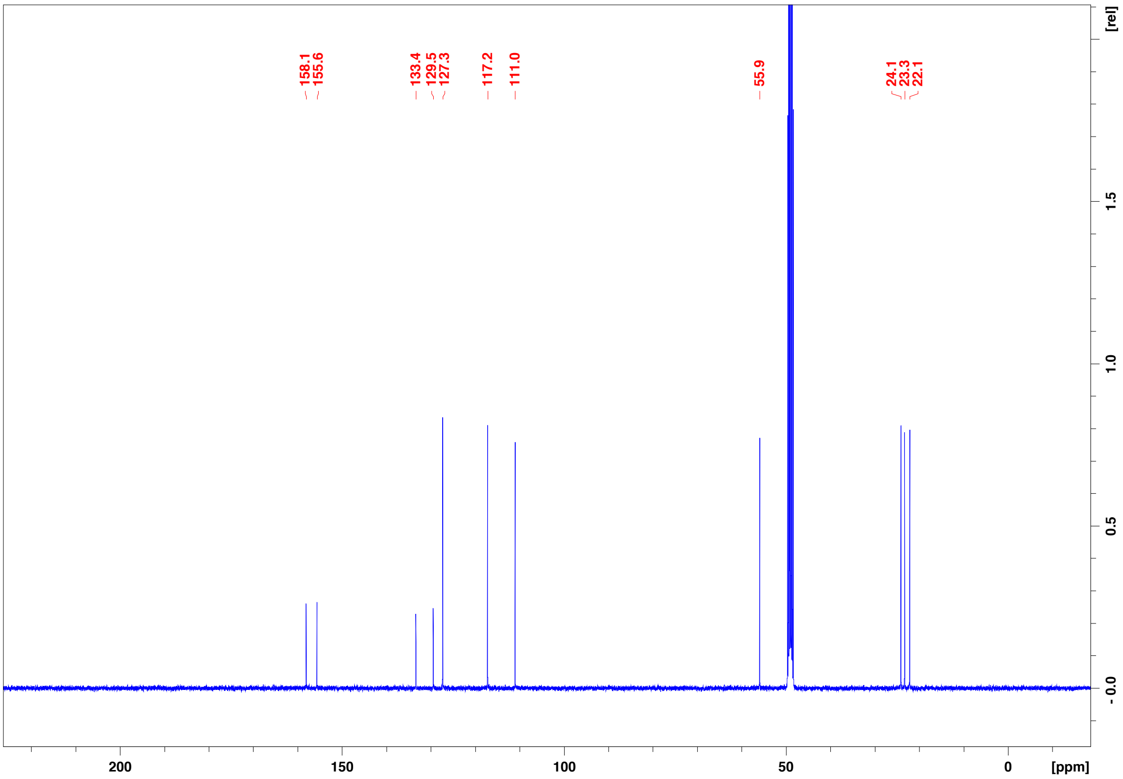

(*E*)-5-methoxy-3,4-dihydronaphthalen-1(2H)-one *O*-tosyl oxime (**30**)

^{1}H-NMR

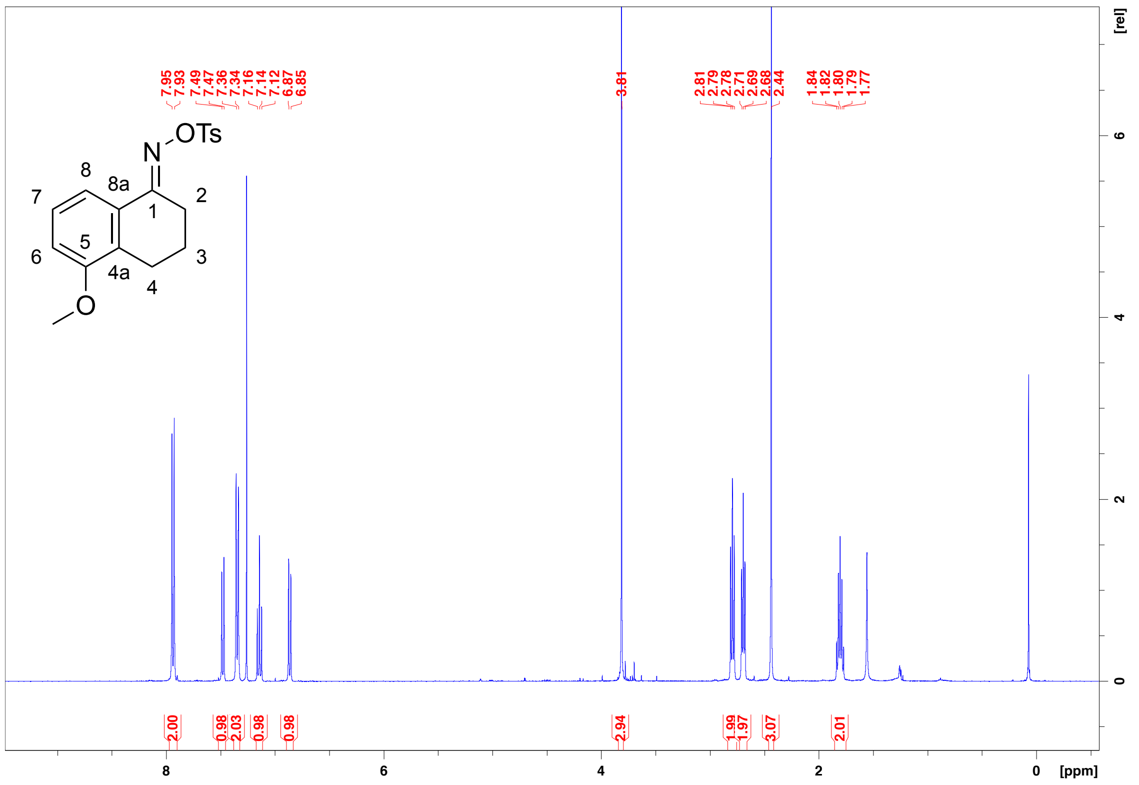

^{13}C-NMR

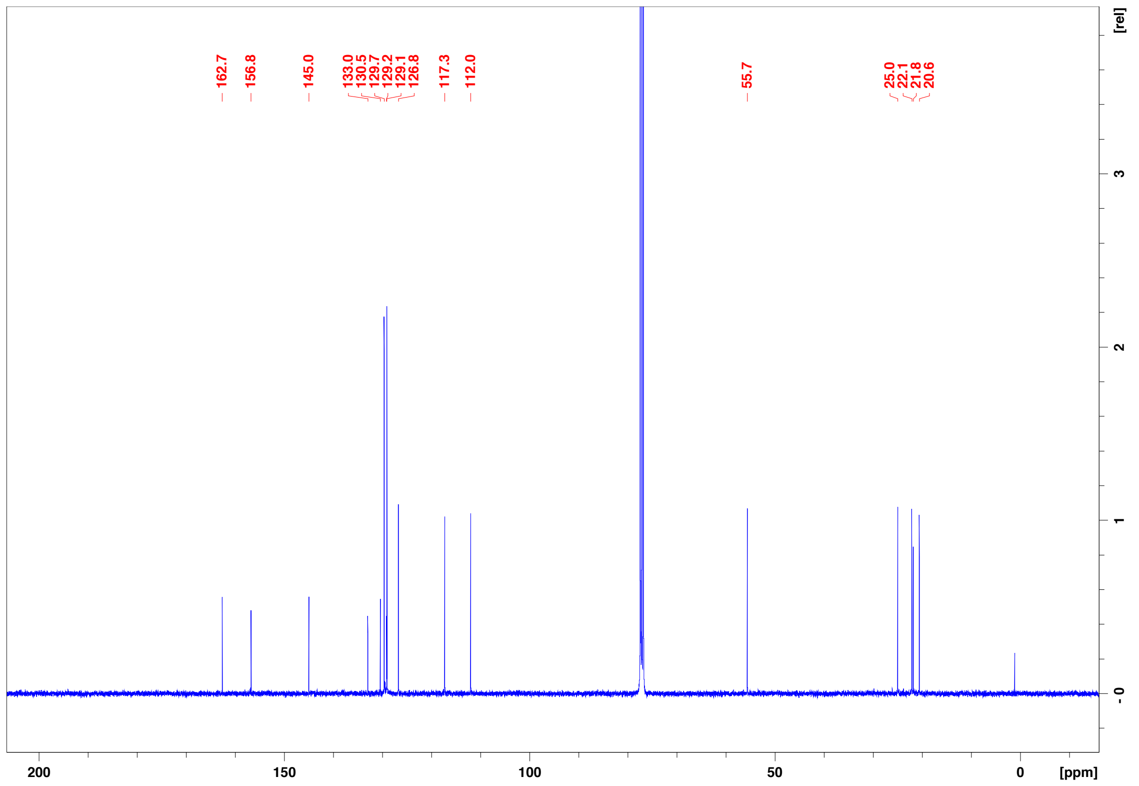

6-methoxy-1,3,4,5-tetrahydro-2H-benzo[b]azepin-2-one (**31**)

^{1}H-NMR

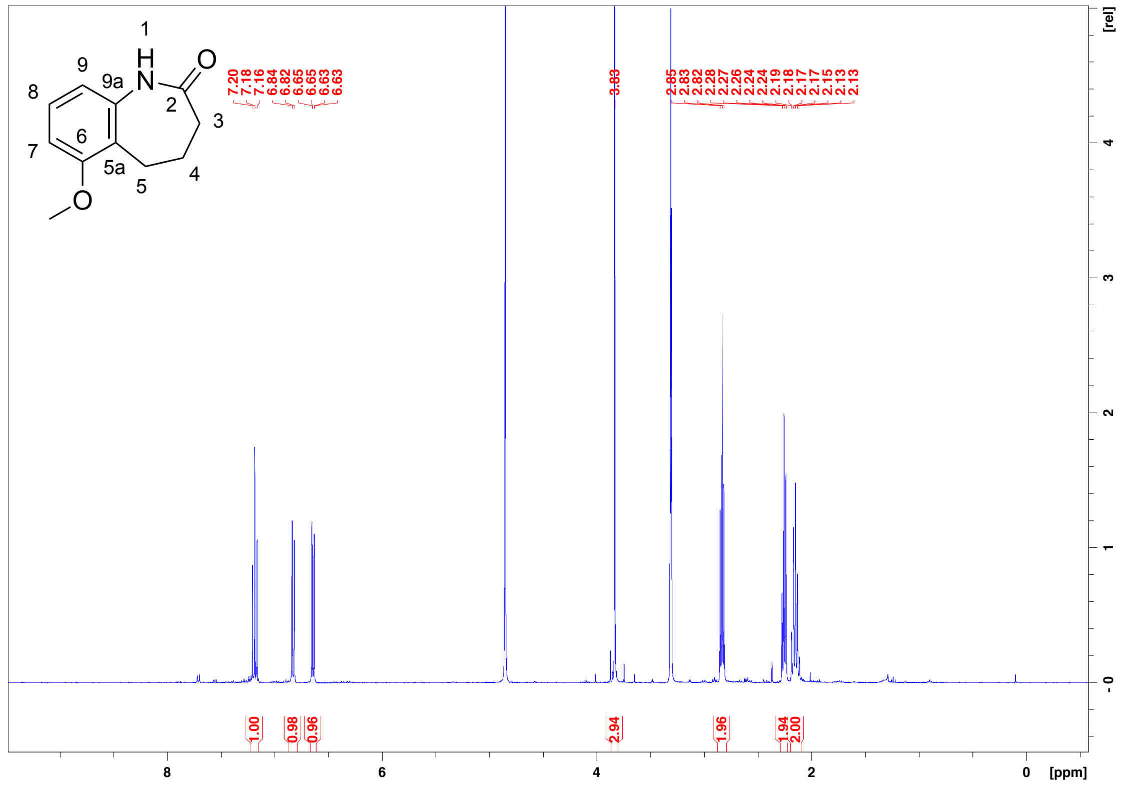

^{13}C-NMR

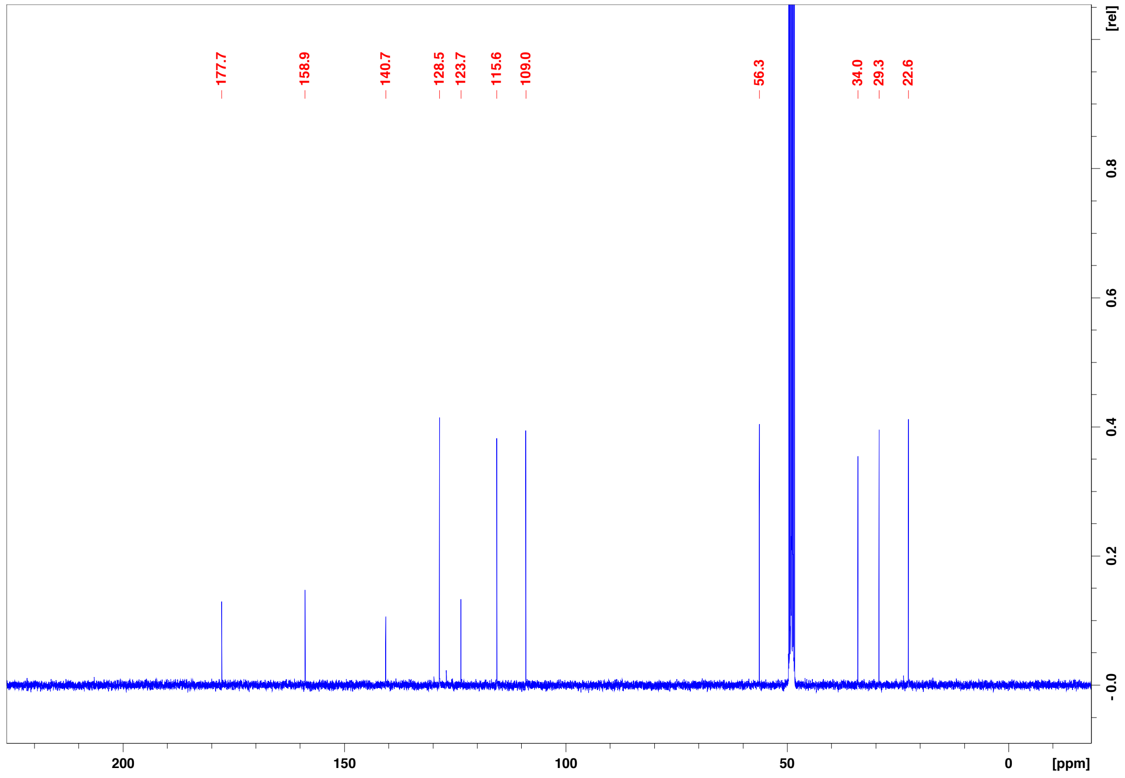

6-hydroxy-1,3,4,5-tetrahydro-2H-benzo[b]azepin-2-one (**32**)

^{1}H-NMR

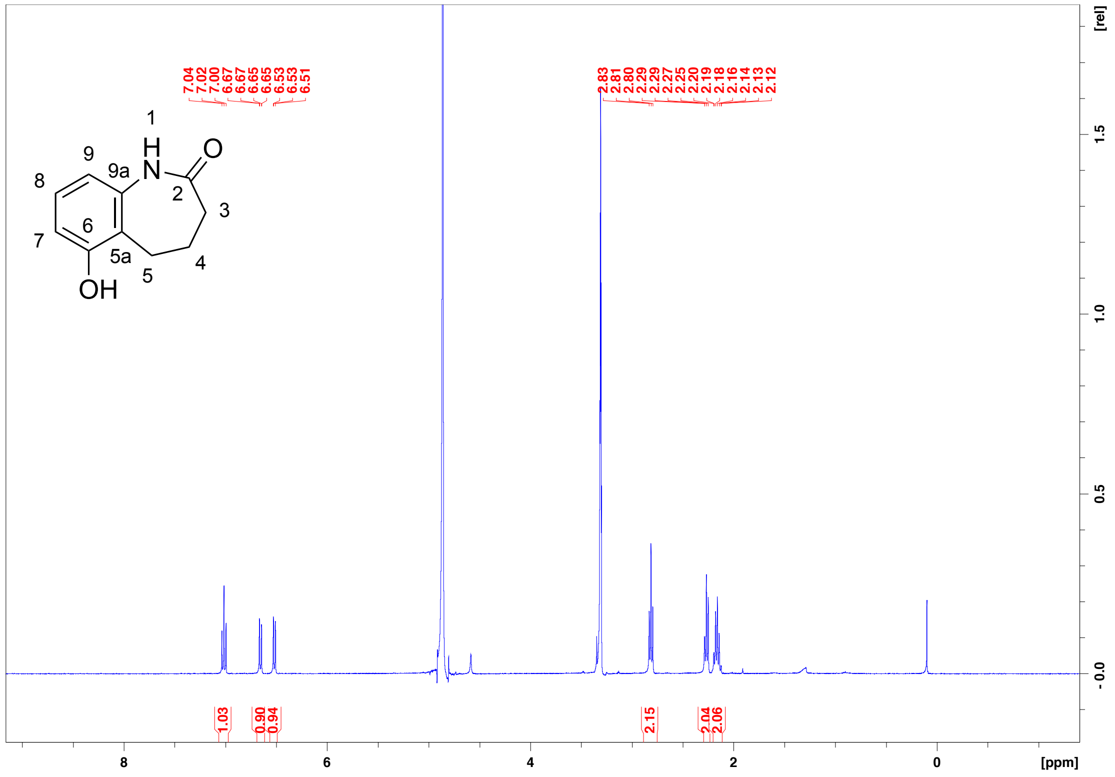

^{13}C-NMR

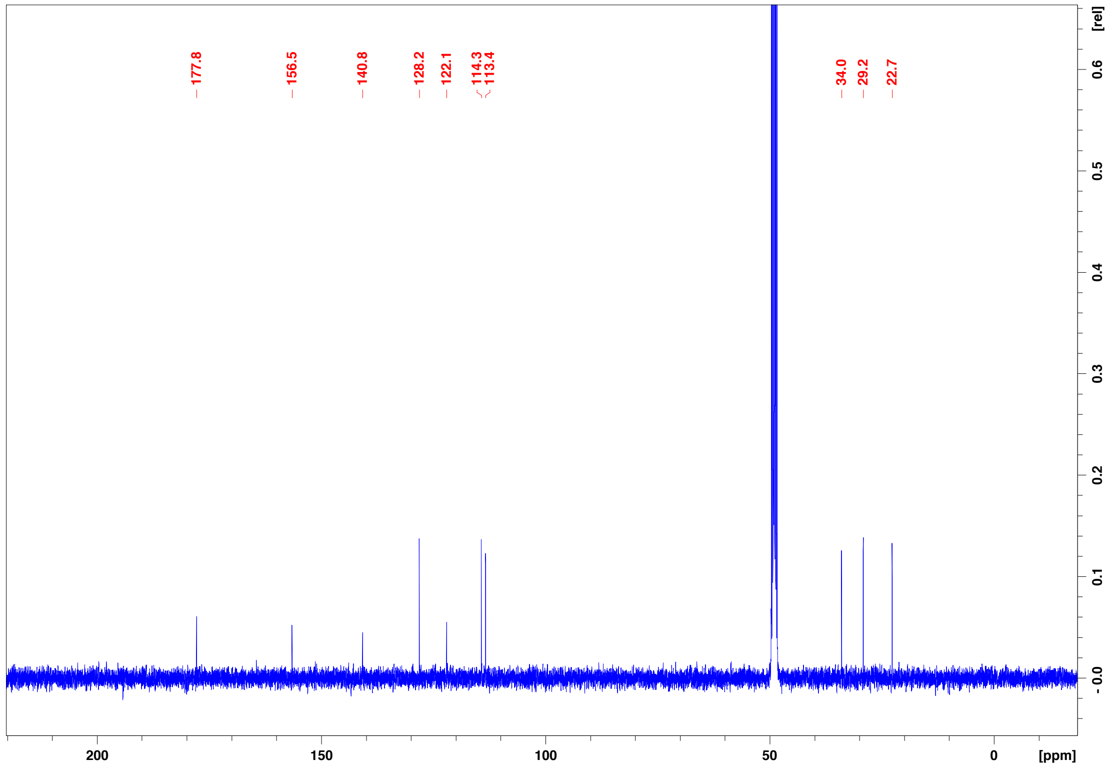

6-hydroxydecahydro-2*H*-benzo[b]azepin-2-one ((±)-**33**)

^{1}H-NMR

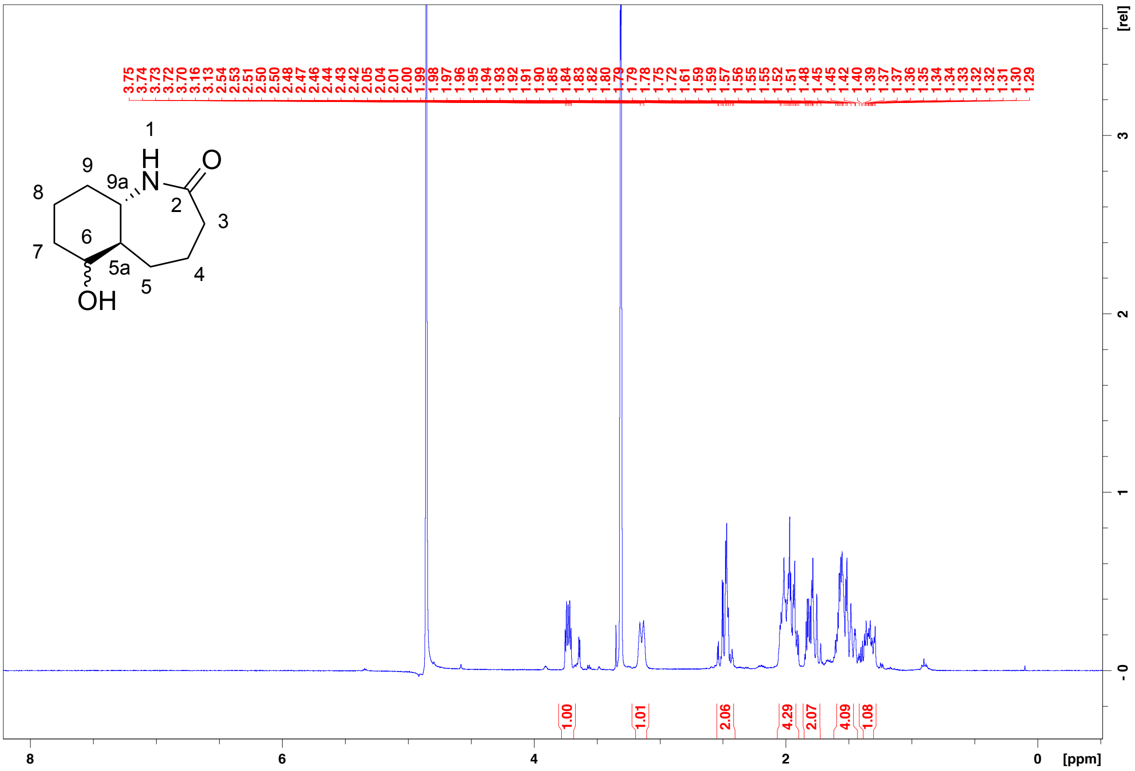

^{13}C-NMR

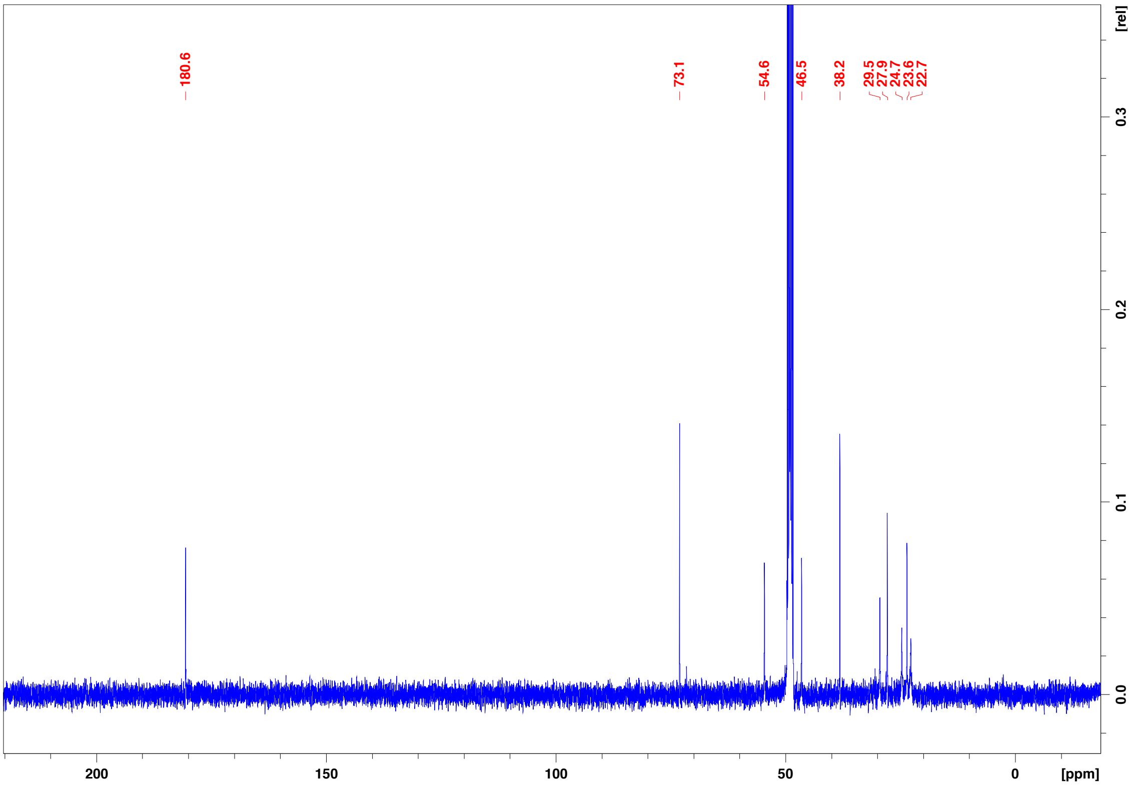

octahydro-1*H*-benzo[b]azepine-2,6-dione ((±)-**34**)

^{1}H-NMR

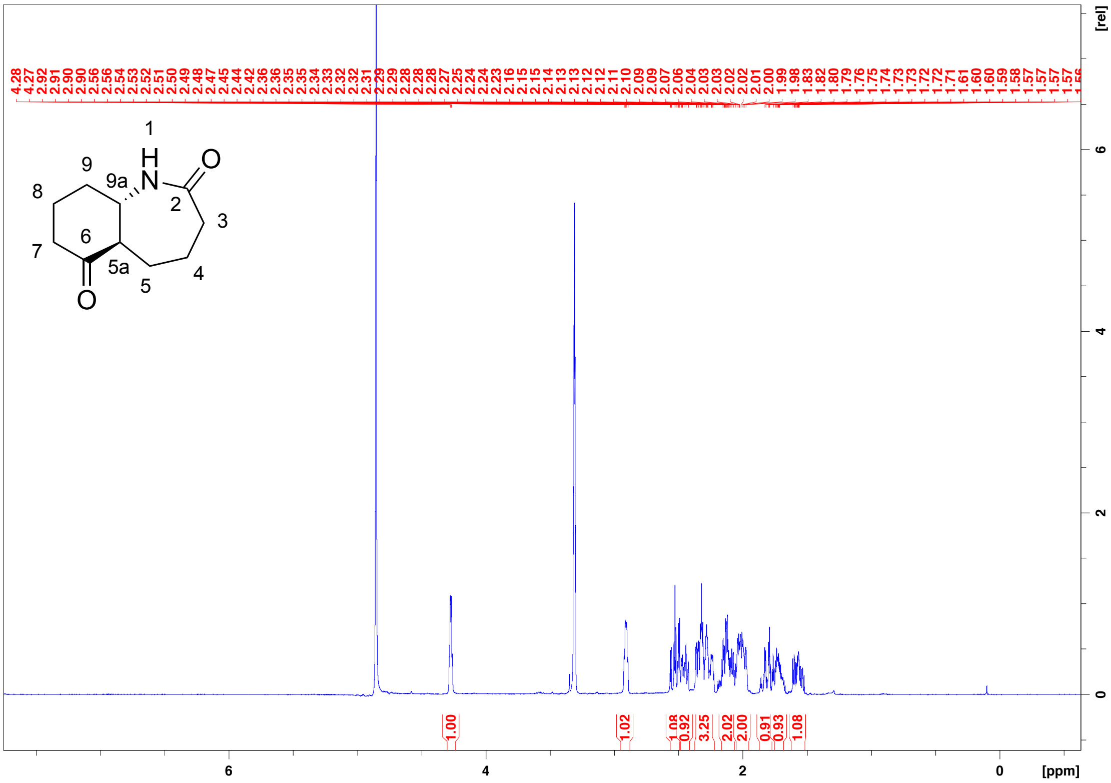

^{13}C-NMR

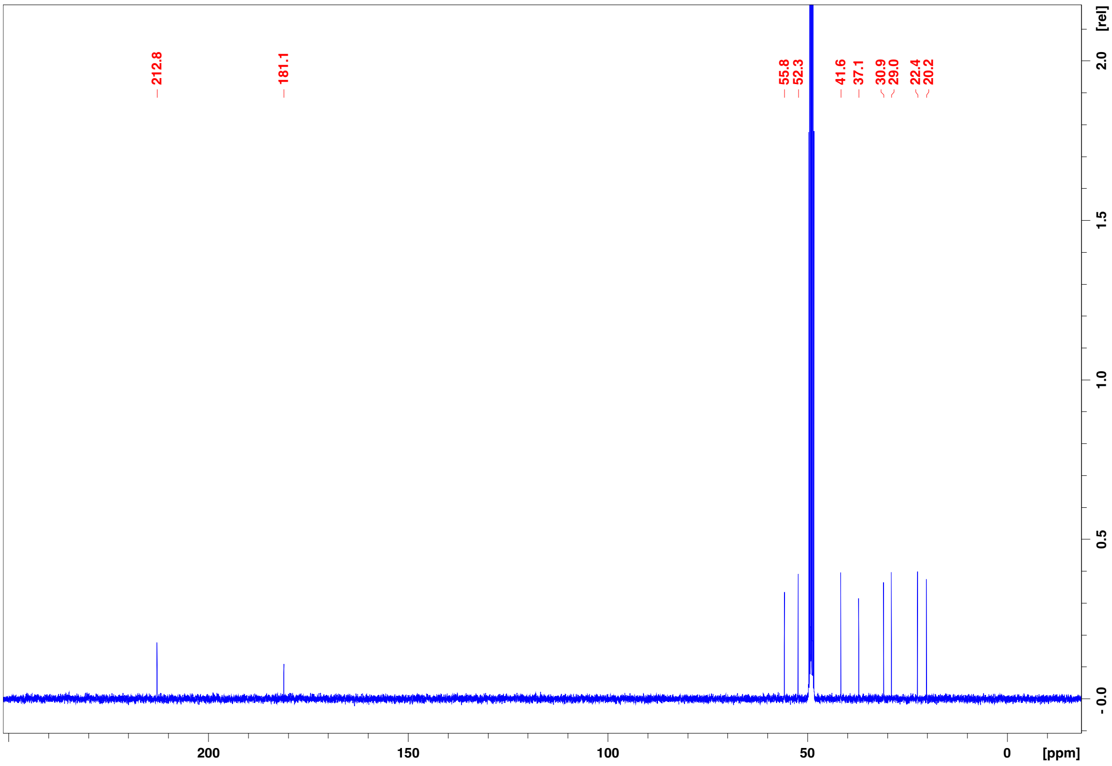

dodecahydroazepino[3,2-b]azepine (*meso*-**4b**)

^{1}H-NMR

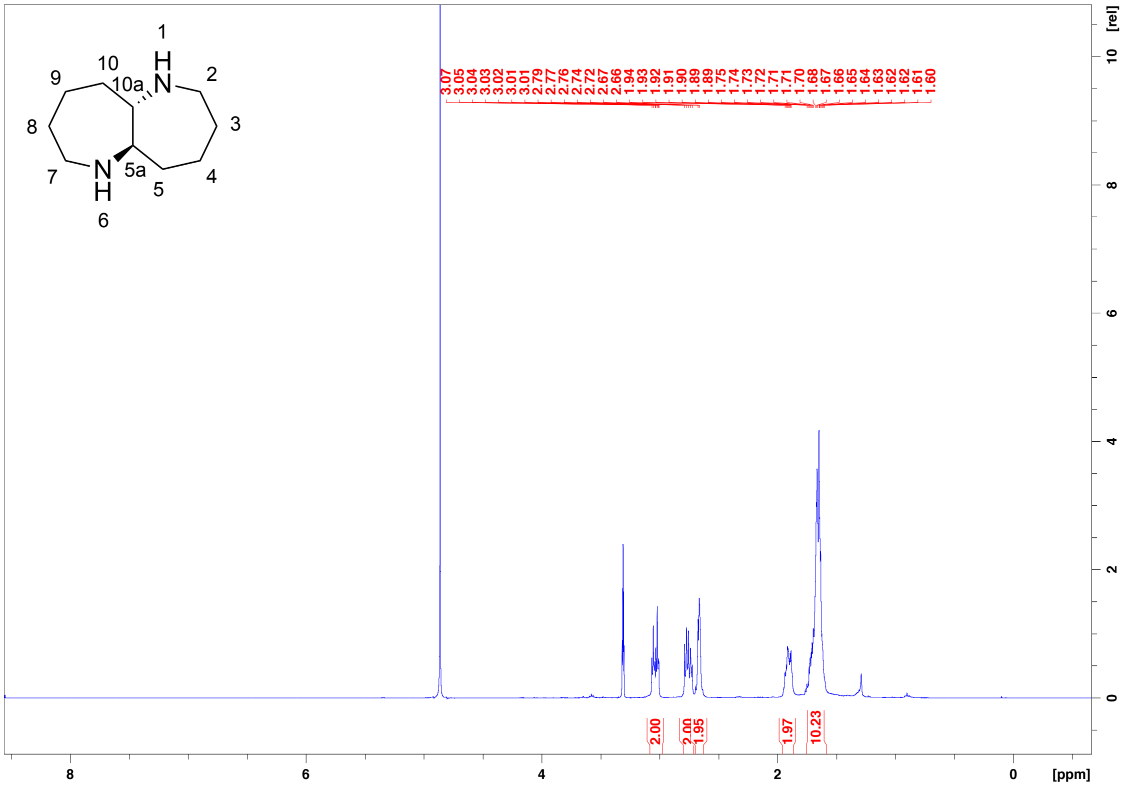

^{13}C-NMR

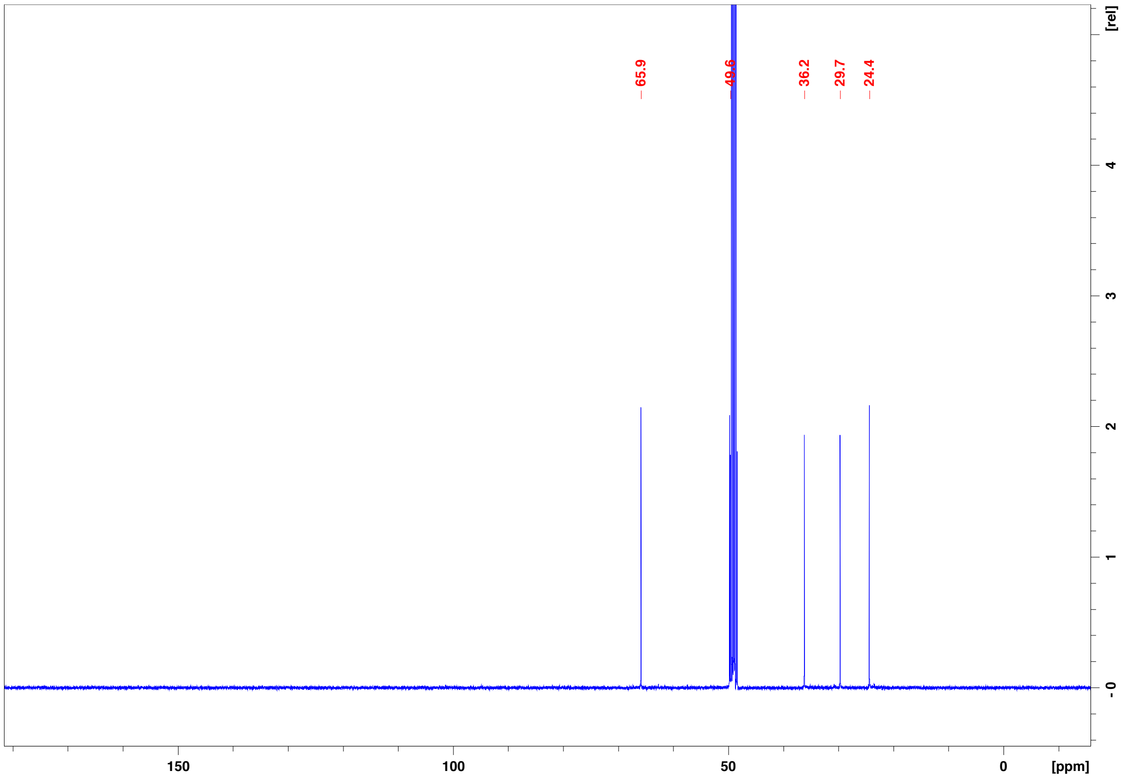

tert-butyl-4-benzyloctahydropyrrolo[3,2-b]azepine-1(2H)-carboxylate ((±)-**35**)

^{1}H-NMR

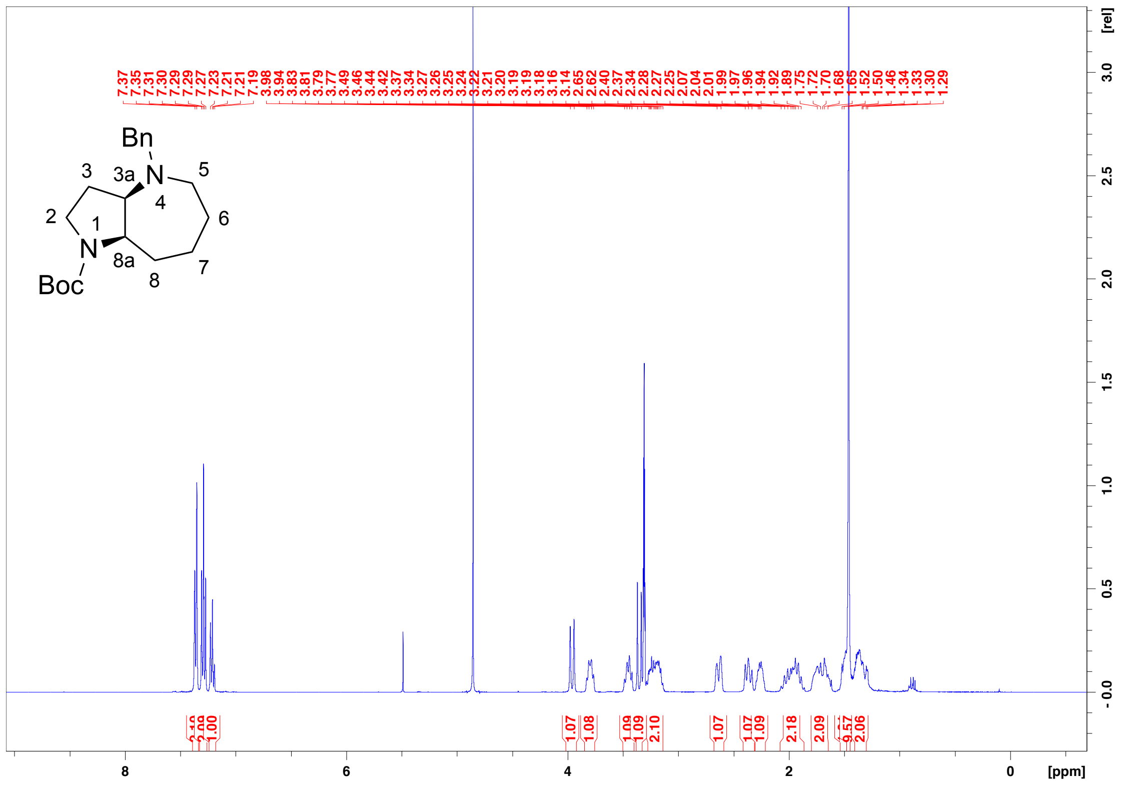

^{13}C-NMR

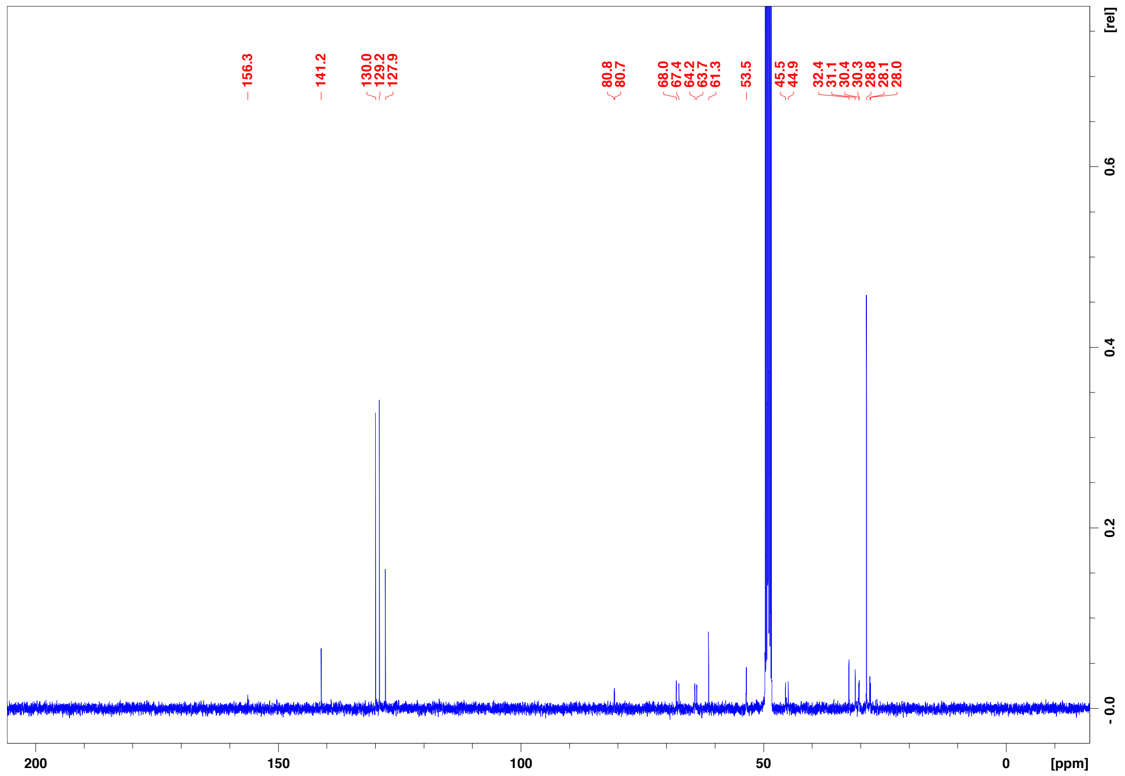

tert-butyl-(3a*R*,8a*R*)-4-benzyloctahydropyrrolo[3,2-b]azepine-1(2H)-carboxylate (*(R,R)*-**35**)

^{1}H-NMR

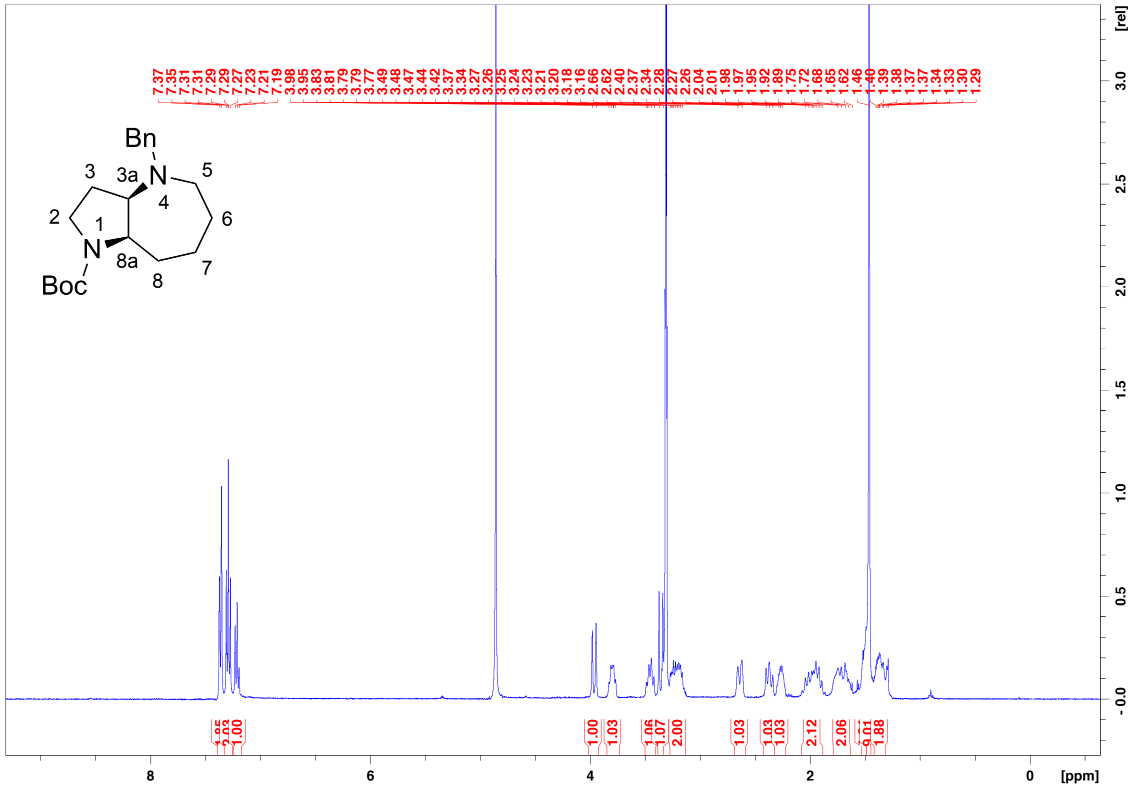

tert-butyl-(3a*S*,8a*S*)-4-benzyloctahydropyrrolo[3,2-b]azepine-1(2H)-carboxylate (*(S,S)*-**35**)

^{1}H-NMR

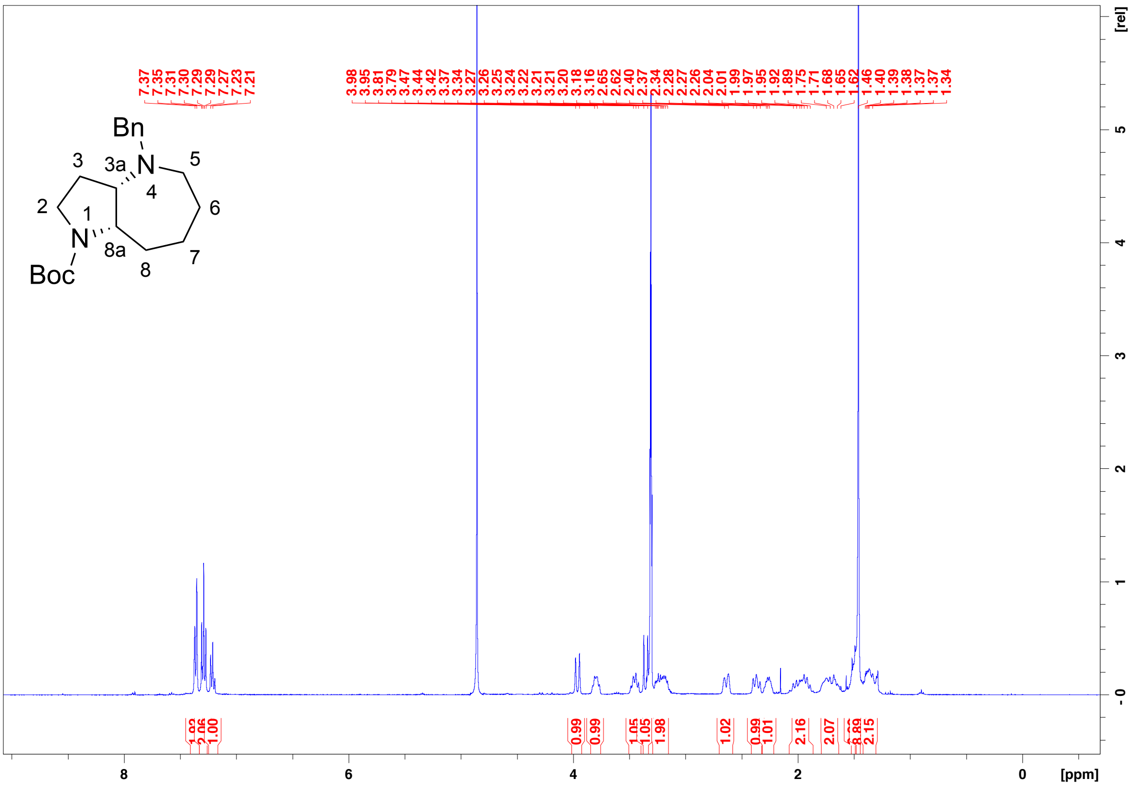

4-benzyldecahydropyrrolo[3,2-b]azepine ((±)-**1a**)

^{1}H-NMR

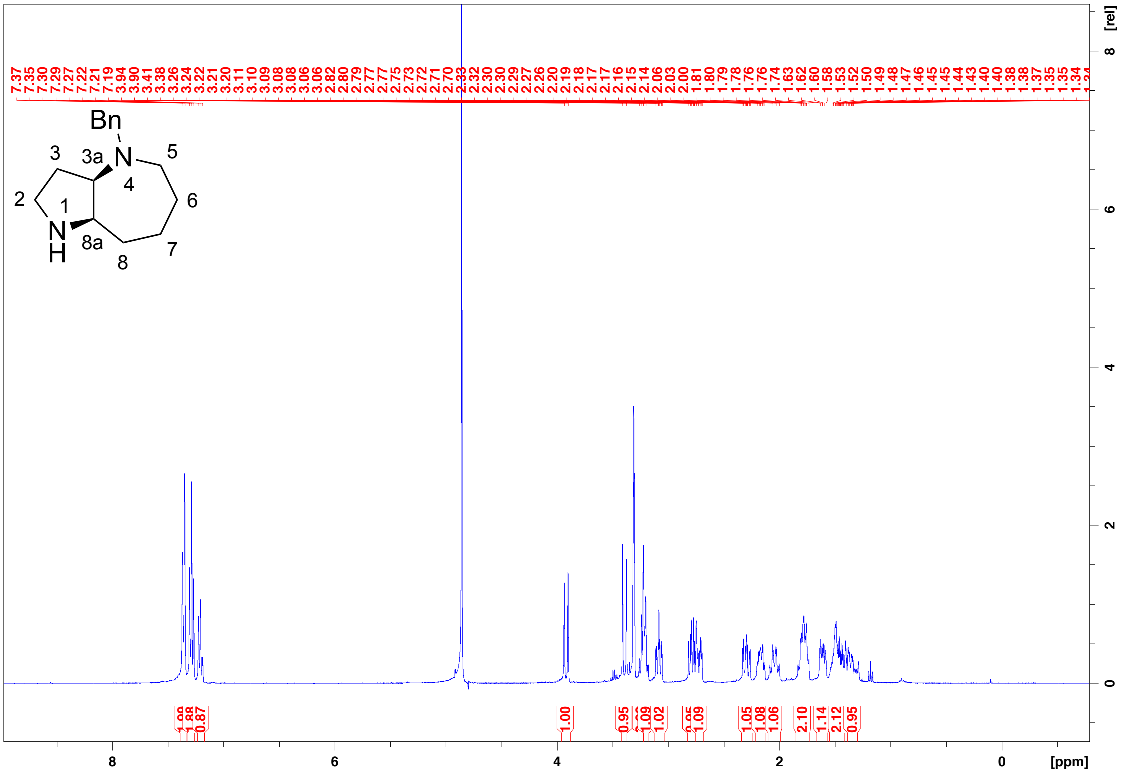

^{13}C-NMR

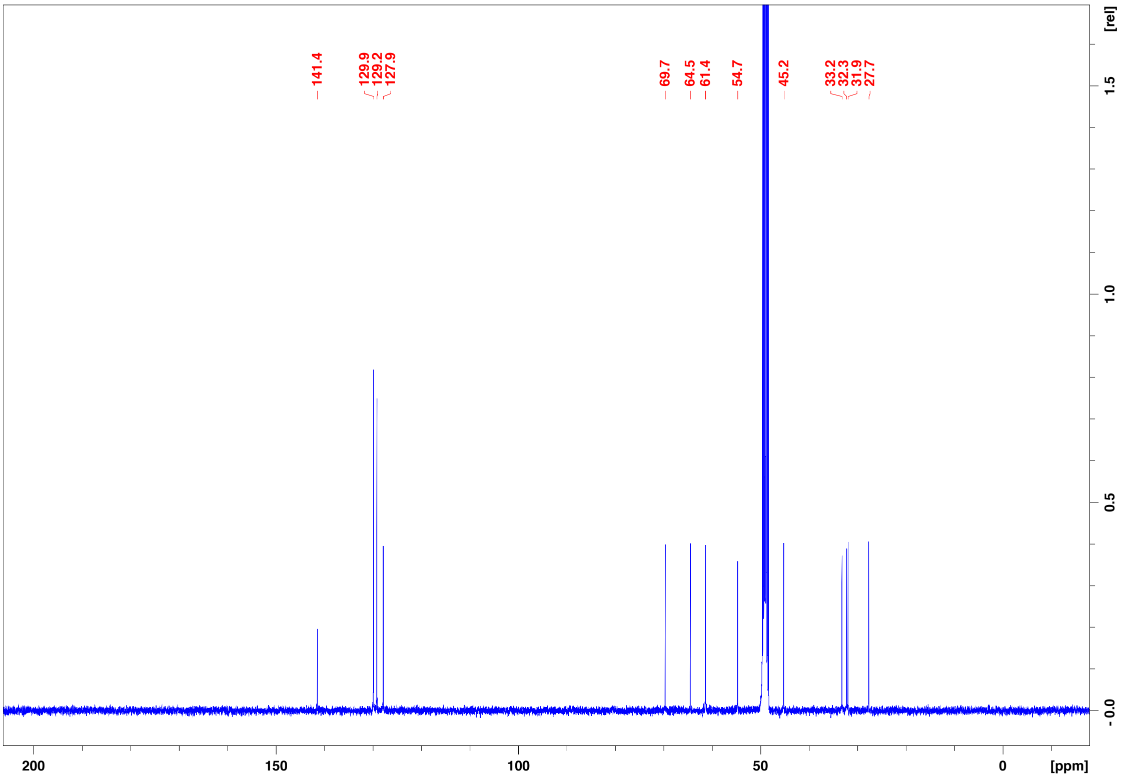

(3a*R*,8a*R*)-4-benzyldecahydropyrrolo[3,2-b]azepine ((*R,R*)-**1a**)

^{1}H-NMR

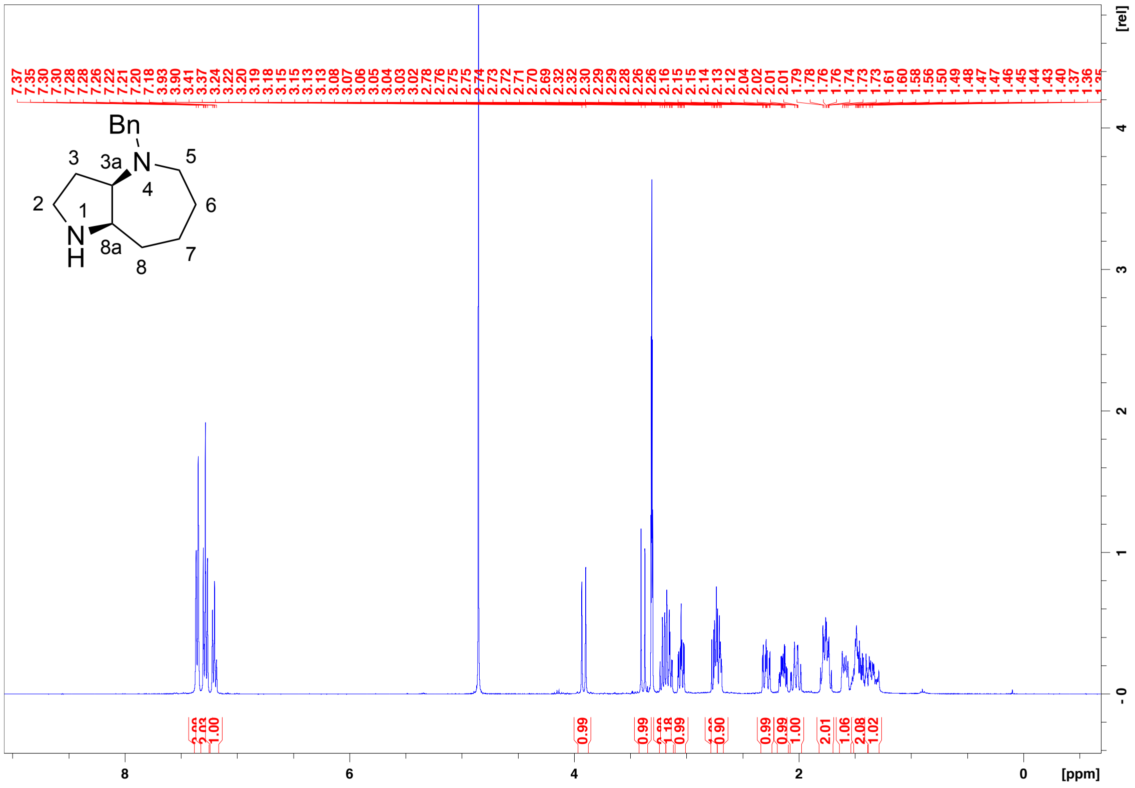

(3a*S*,8a*S*)-4-benzyldecahydropyrrolo[3,2-b]azepine ((*S,S*)-**1a**)

^{1}H-NMR

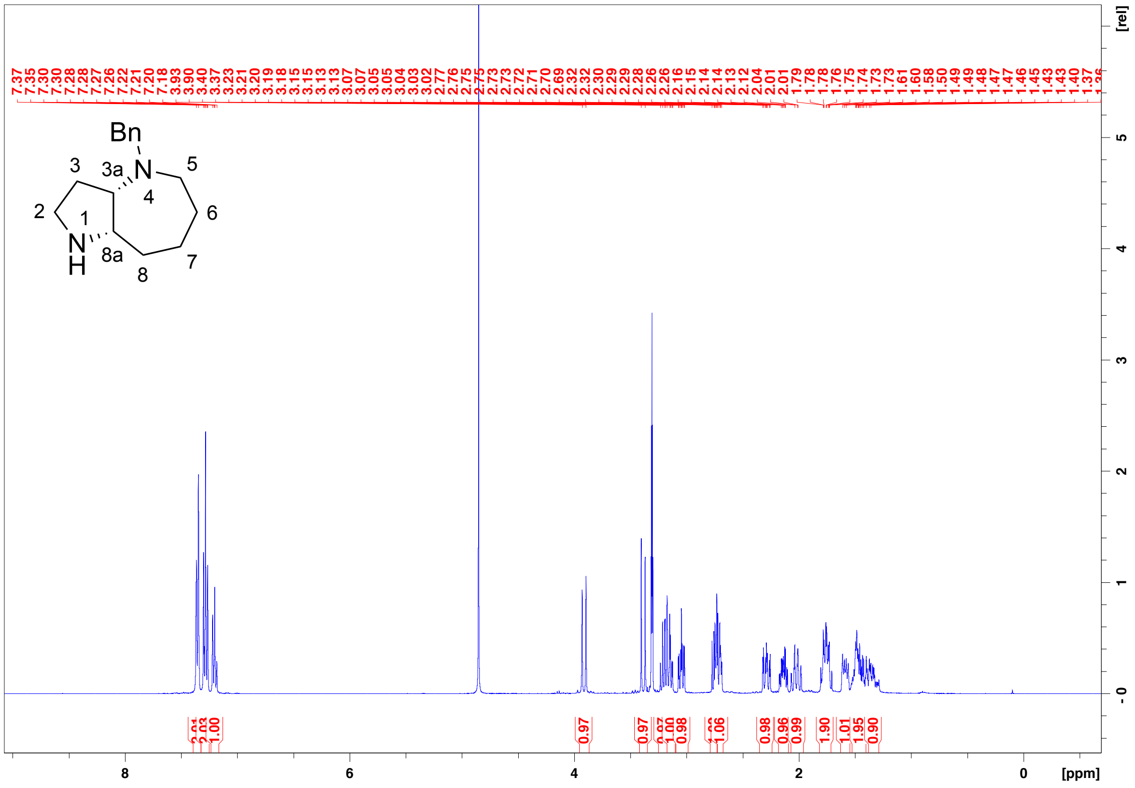

Trifluoro-1-octahydropyrrolo[3,2-b]azepin-4(1H)-yl)ethanone ((±)-**36**)

^{1}H-NMR

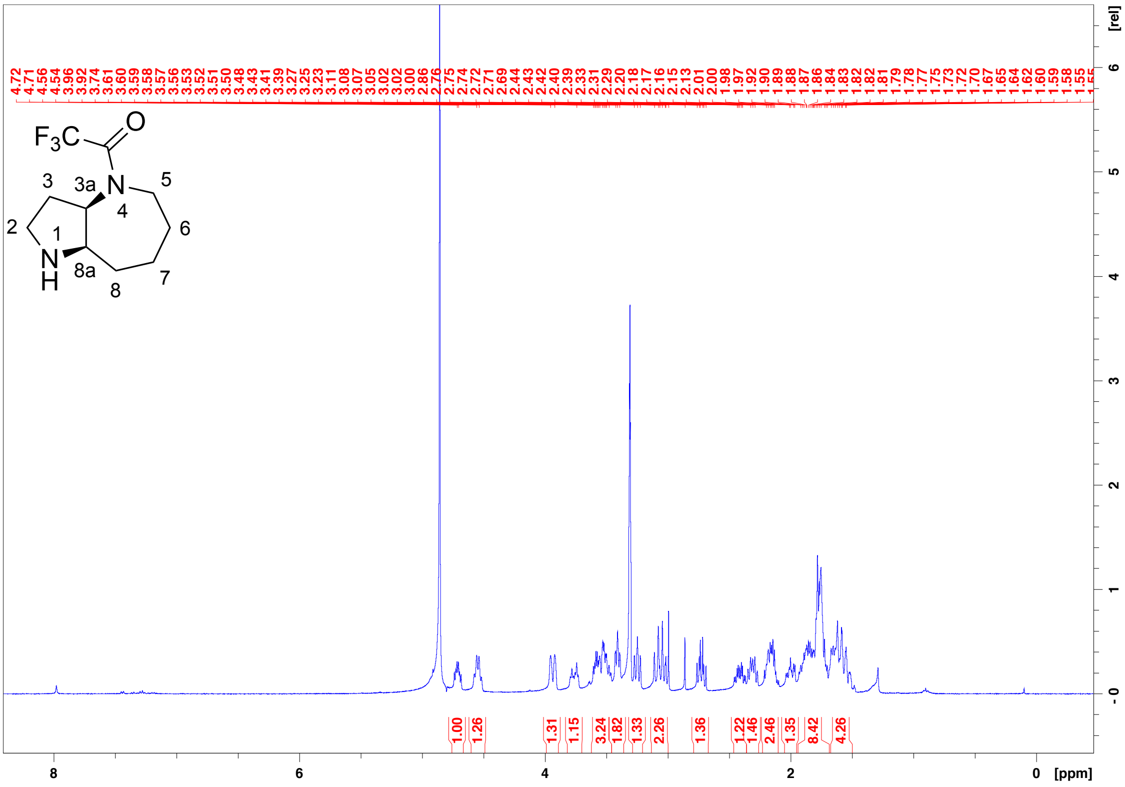

^{13}C-NMR

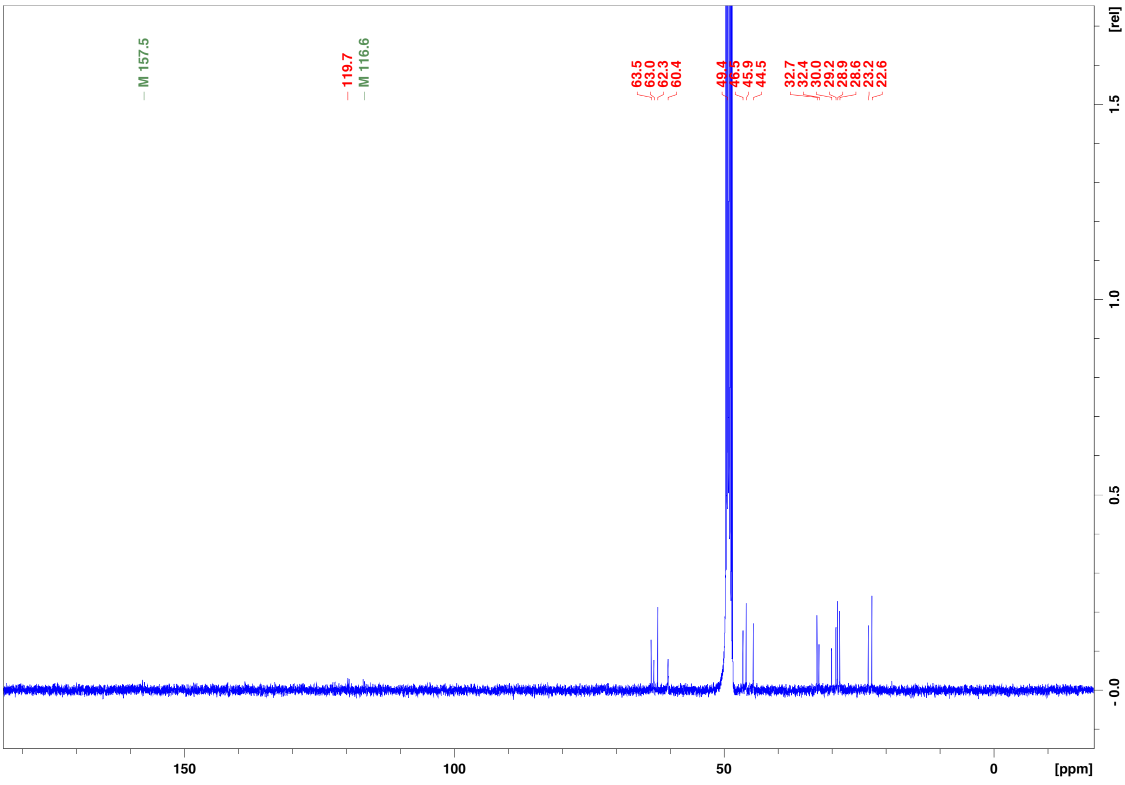

1-benzyldecahydropyrrolo[3,2-b]azepine ((±)-**17a**)

^{1}H-NMR

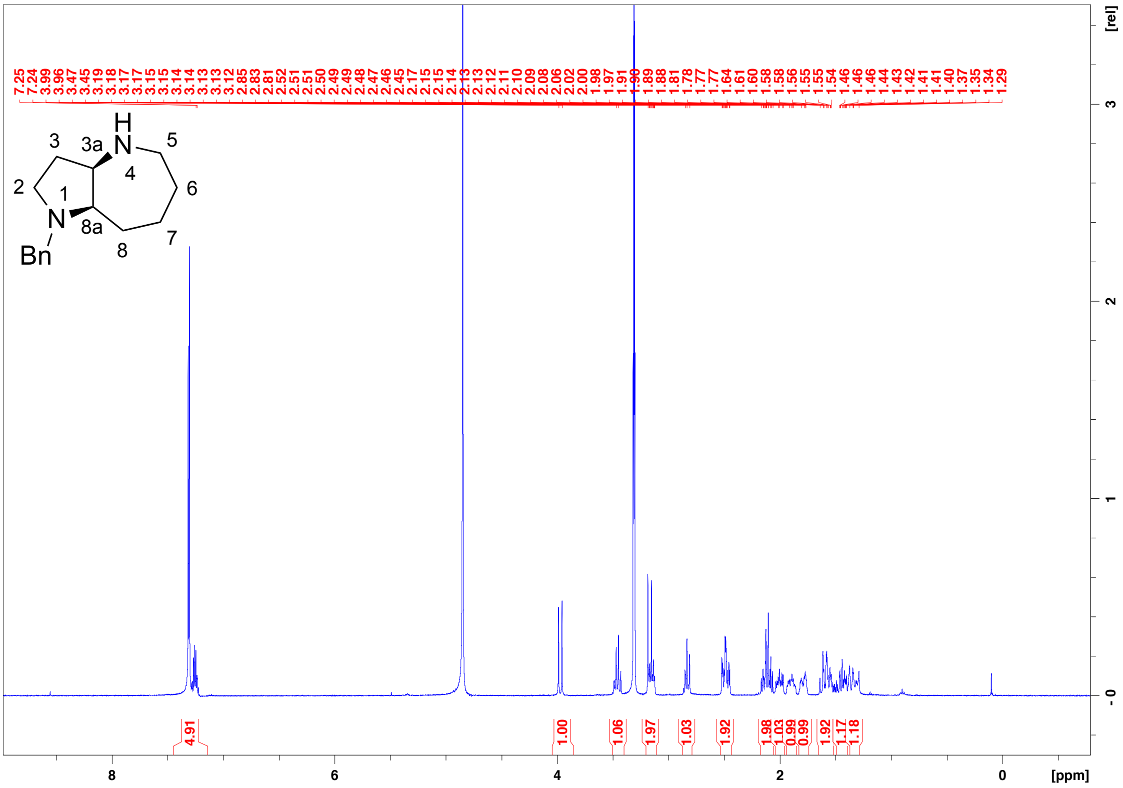

^{13}C-NMR

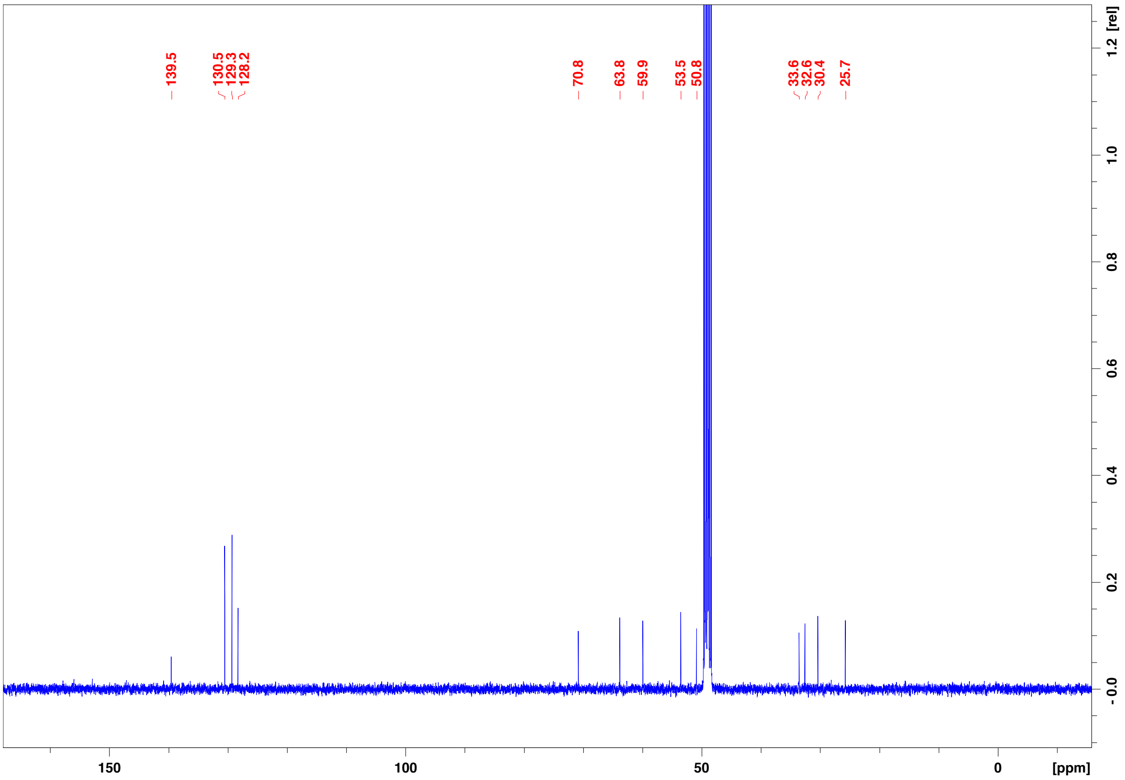

tert-butyl-5-oxooctahydropyrrolo[3,2-b]azepine-1(2H)-carboxylate ((±)-**37**)

^{1}H-NMR

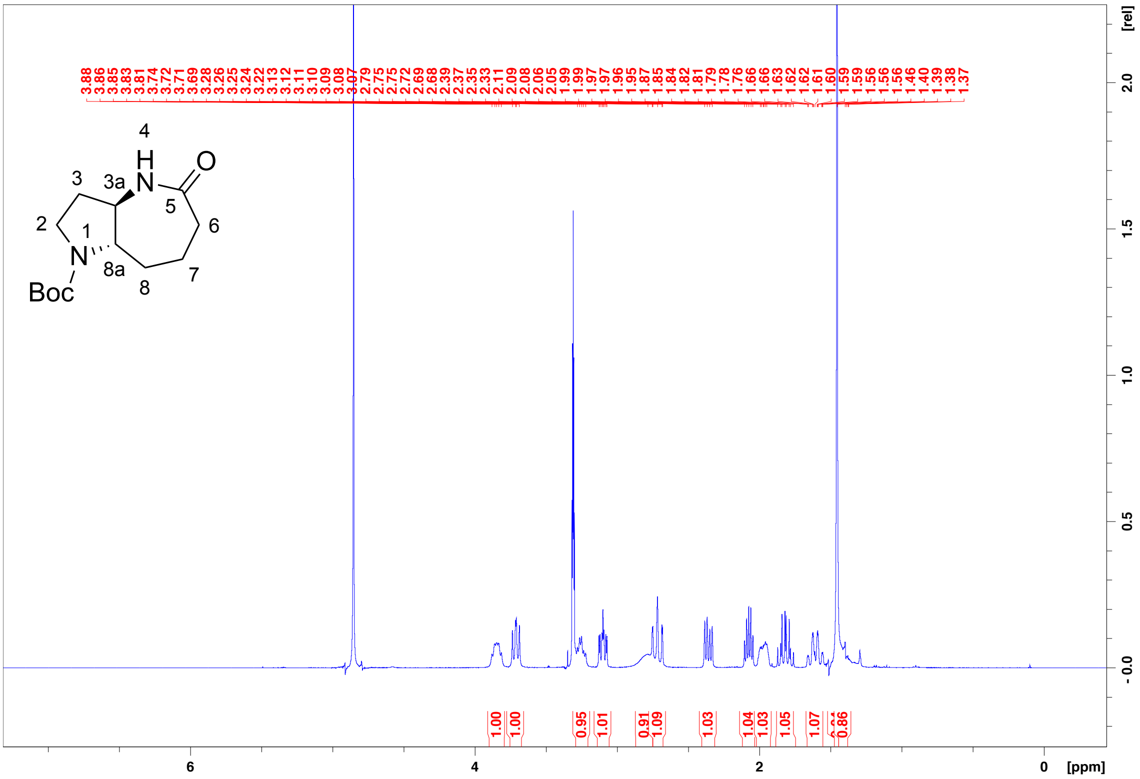

^{13}C-NMR

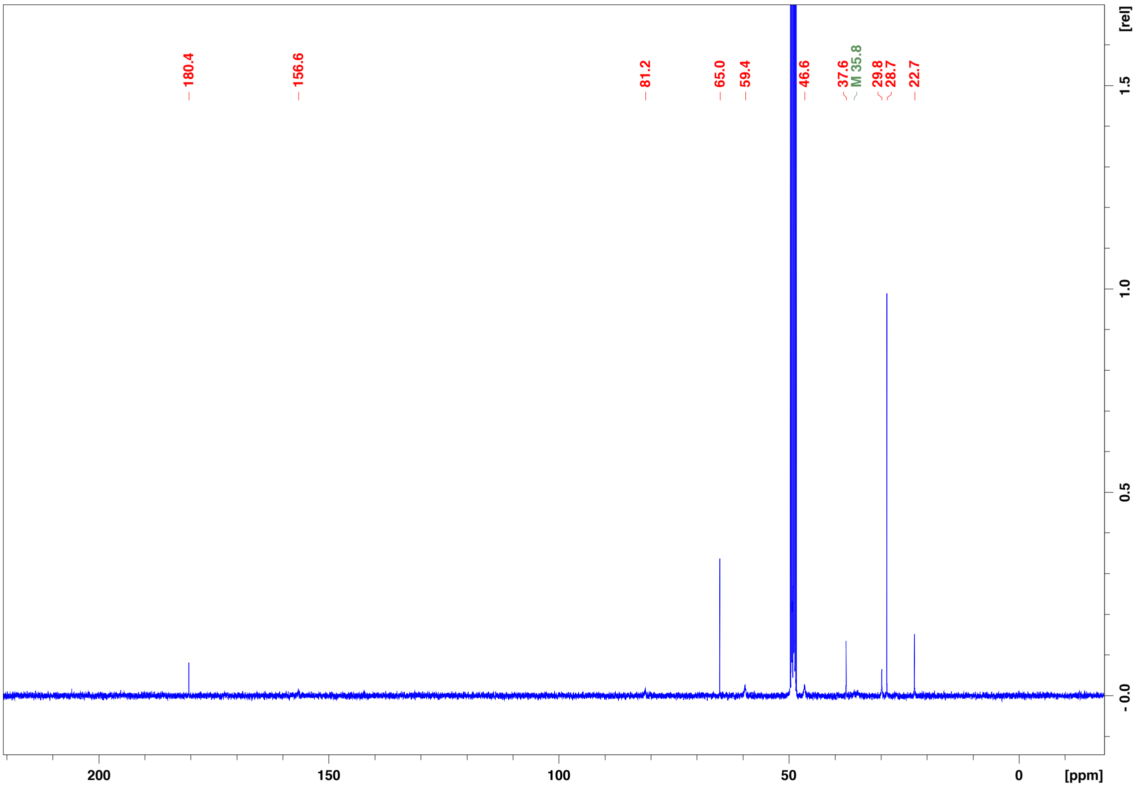

<u>*tert*-butyl-octahydropyrrolo[3,2-b]azepine-1(2H)-carboxylate ((±)-**38**)</u>

^{1}H-NMR

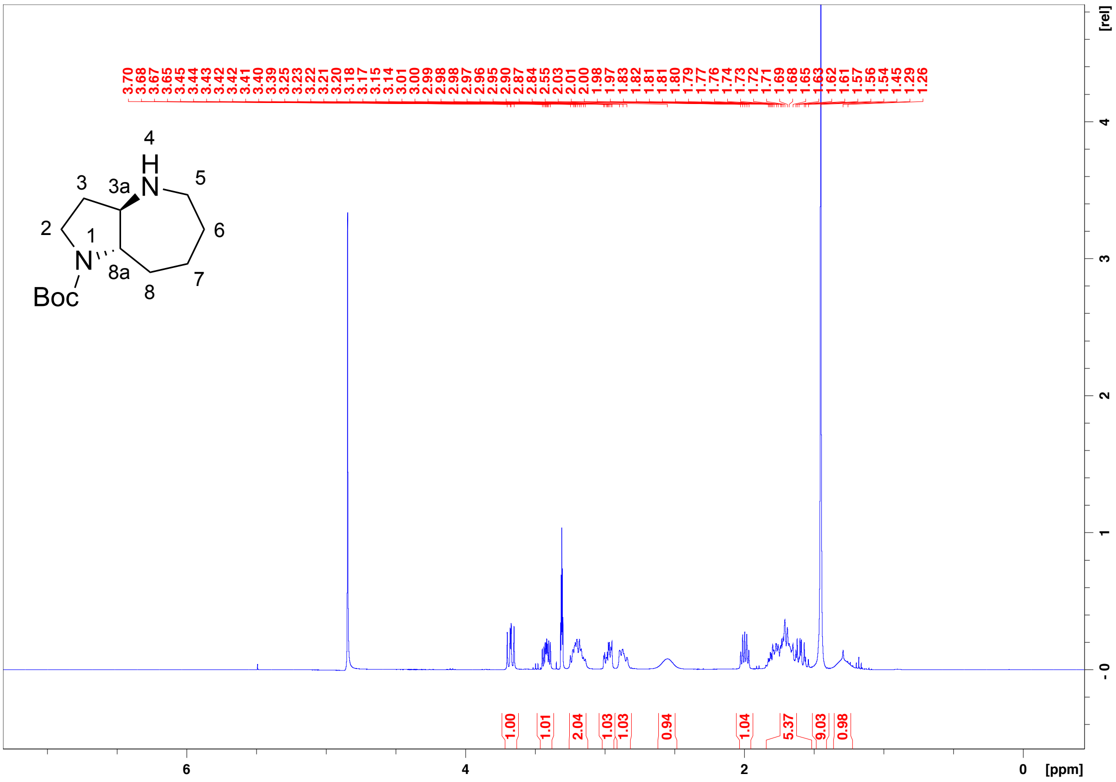

^{13}C-NMR

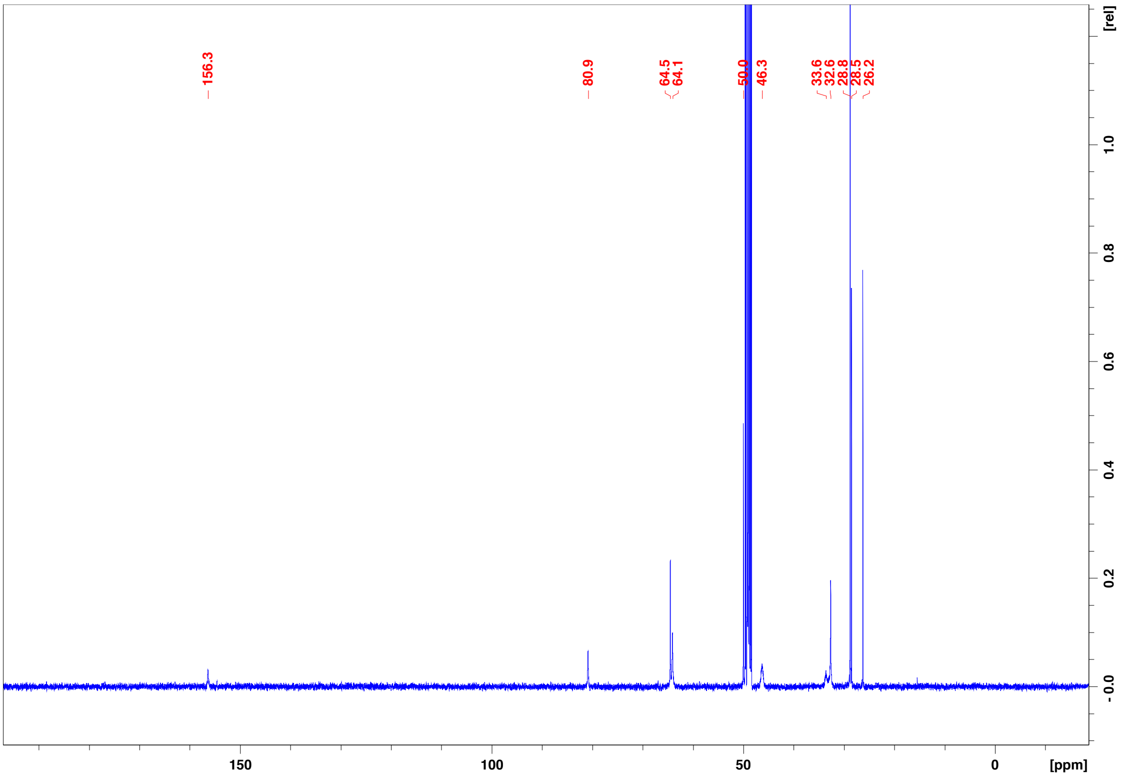

4-benzyldecahydropyrrolo[3,2-b]azepine ((±)-**1b**)

^{1}H-NMR

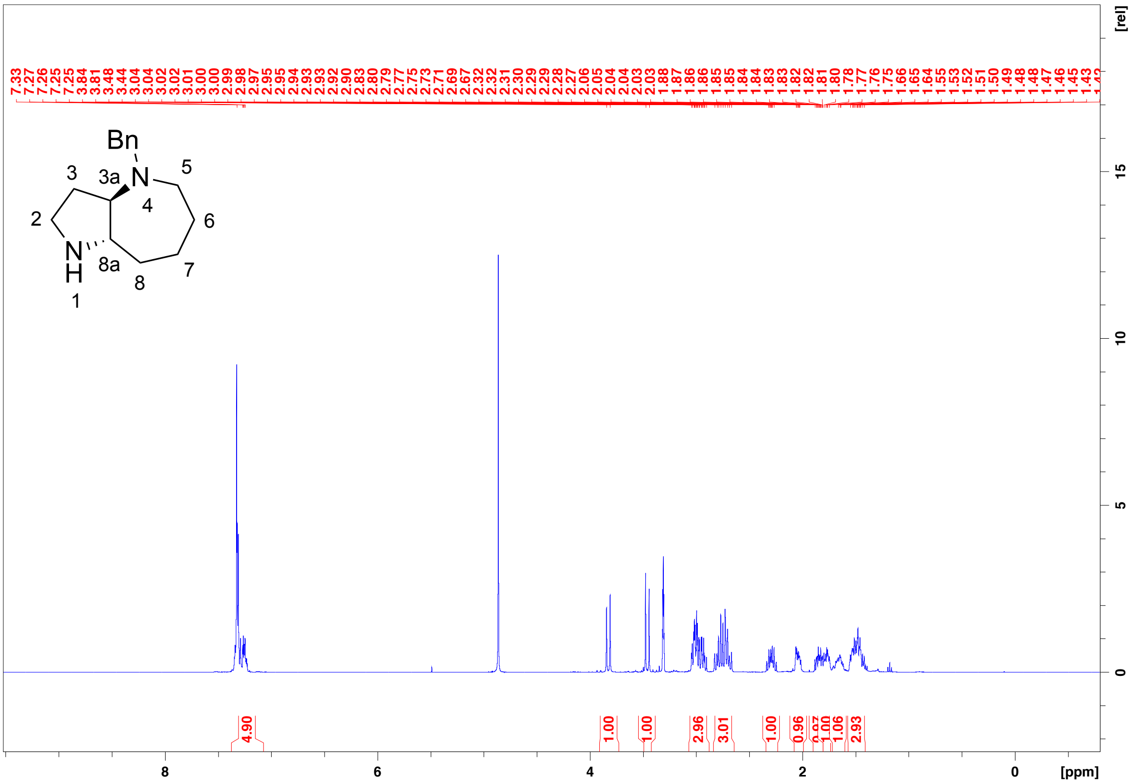

^{13}C-NMR

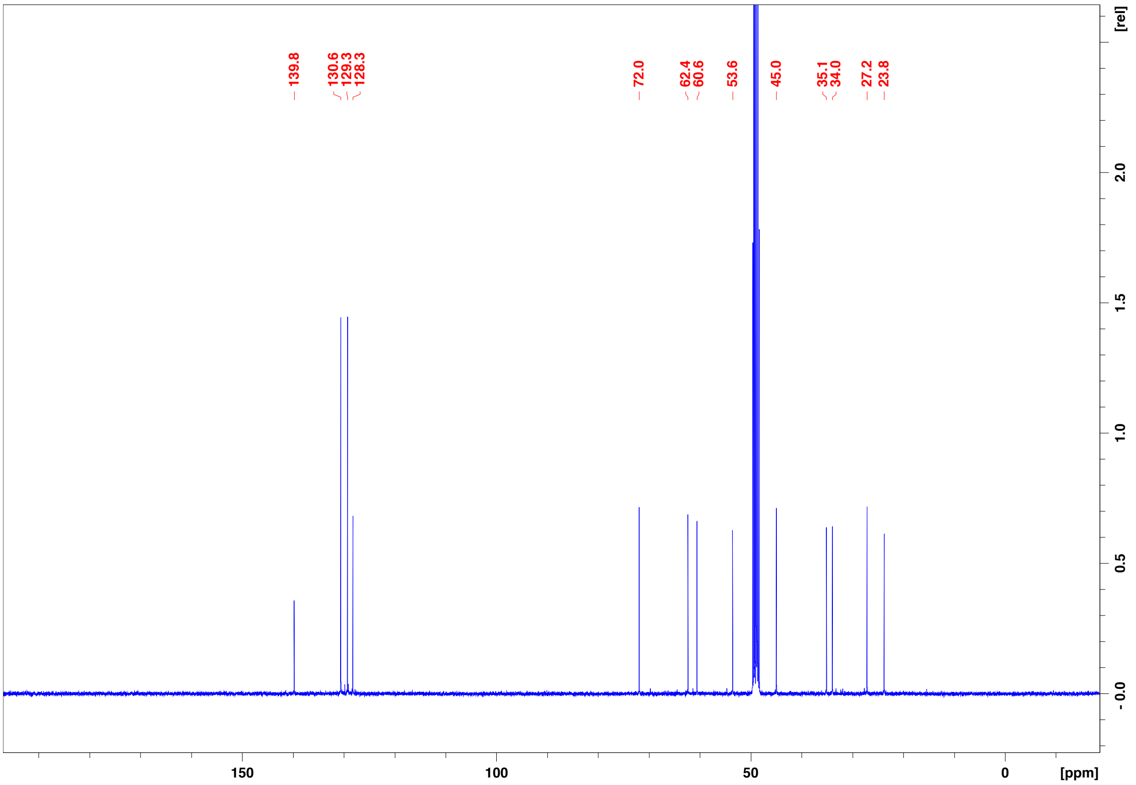

tert-butyl-decahydro-1H-pyrido[3,2-b]azepine-1-carboxylate ((±)-**39**)

^{1}H-NMR

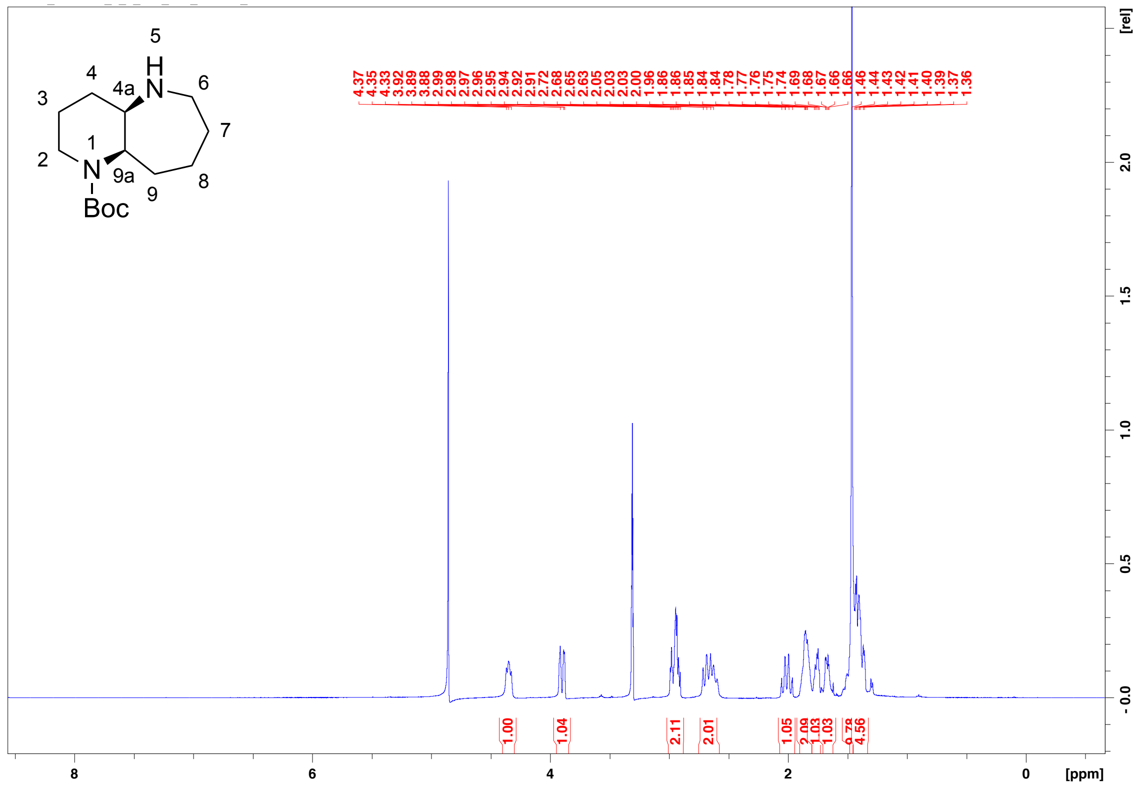

^{13}C-NMR

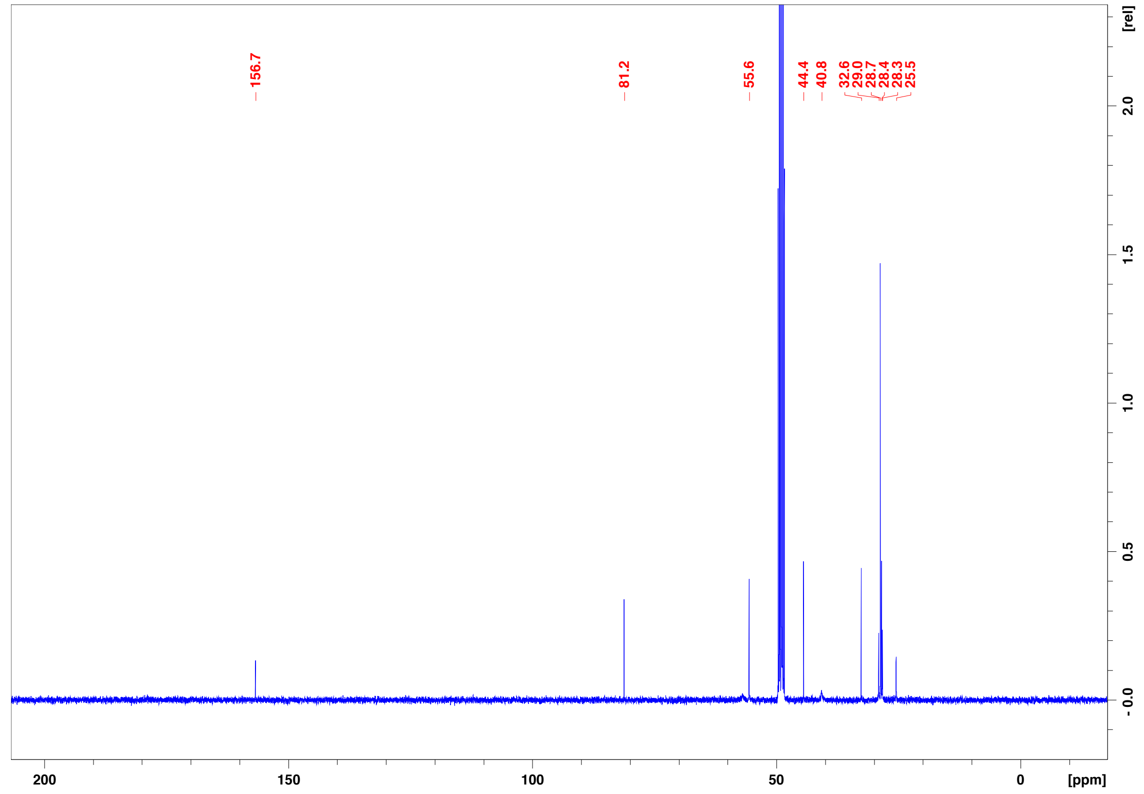

5-benzyldecahydro-1H-pyrido[3,2-b]azepine ((±)-**40a**)

^{1}H-NMR

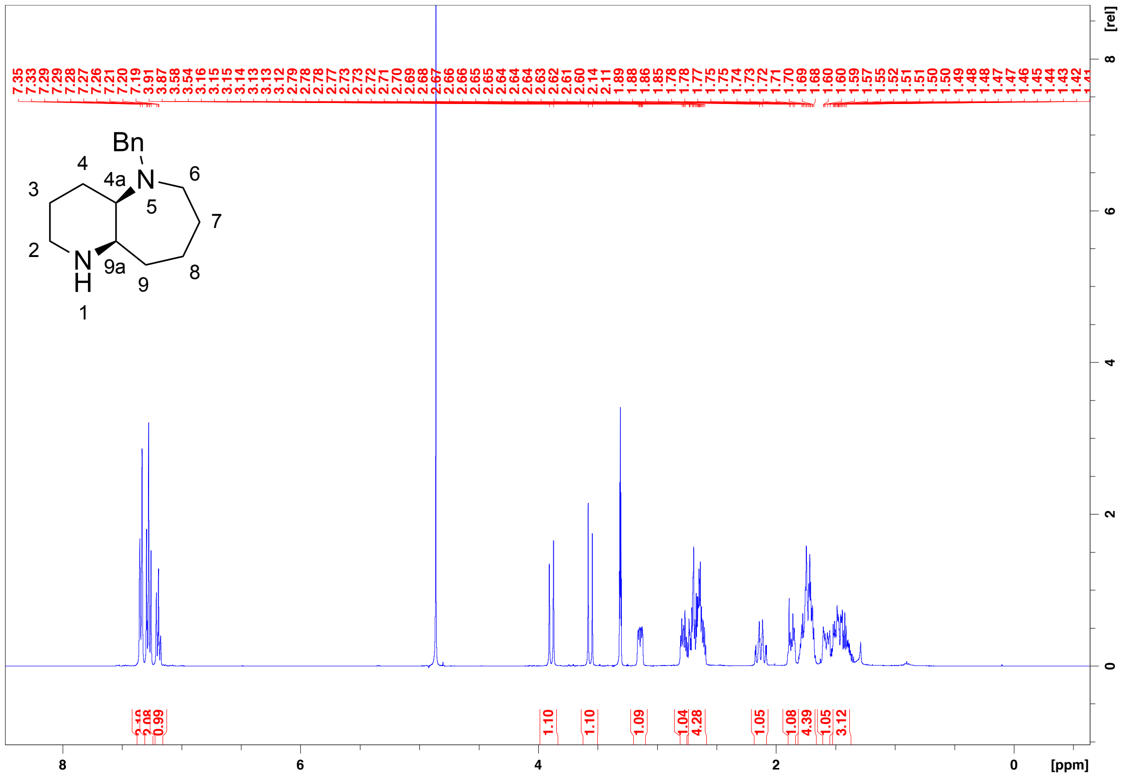

^{13}C-NMR

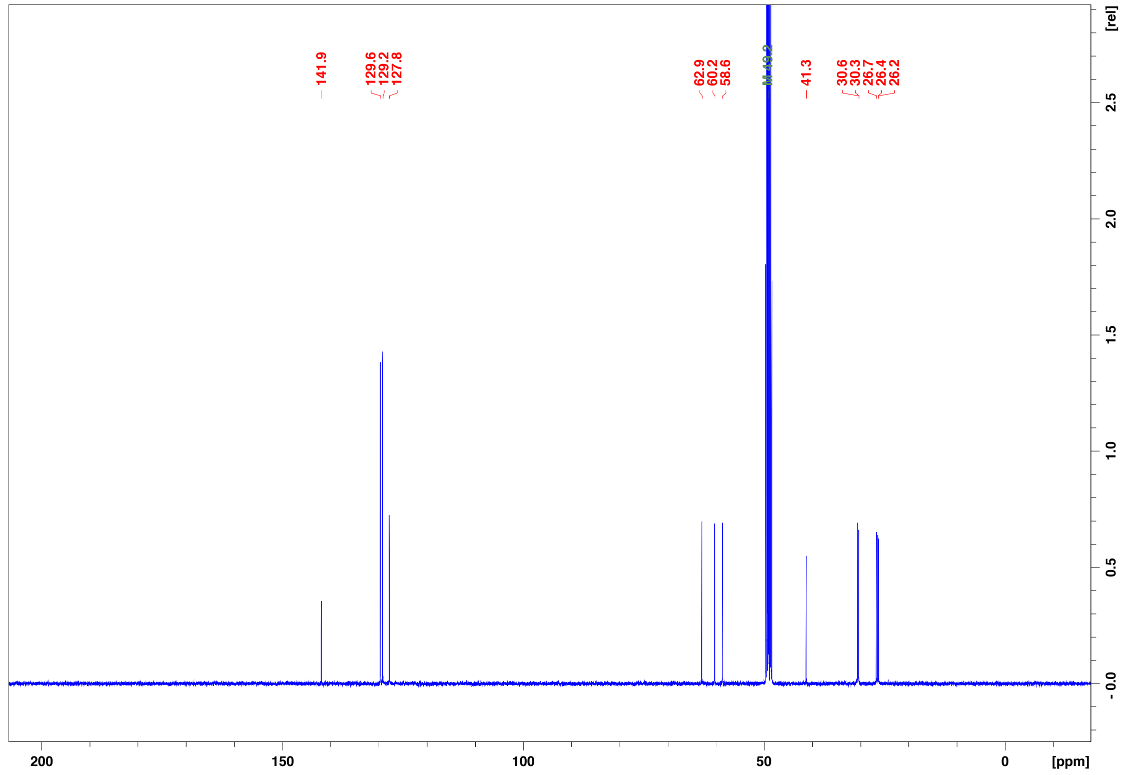

<u>1-benzyldecahydro-1H-pyrido[3,2-b]azepine ((±)-**27a**)</u>

^{1}H-NMR

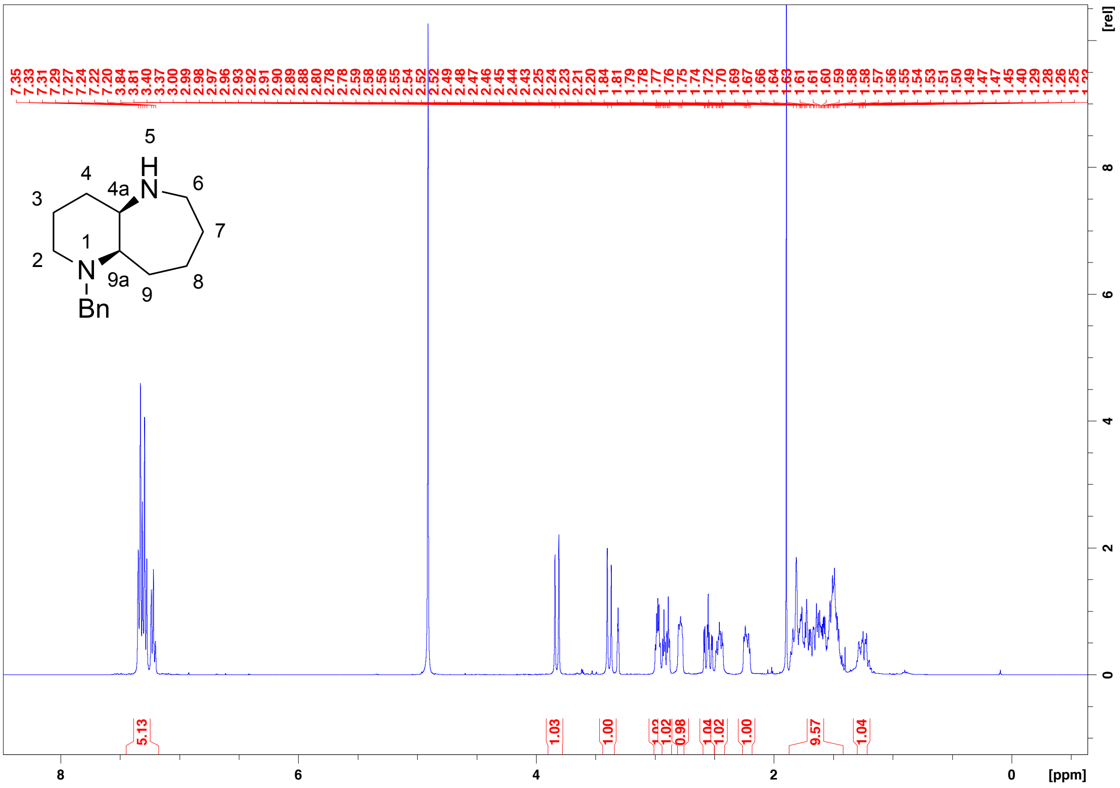

^{13}C-NMR

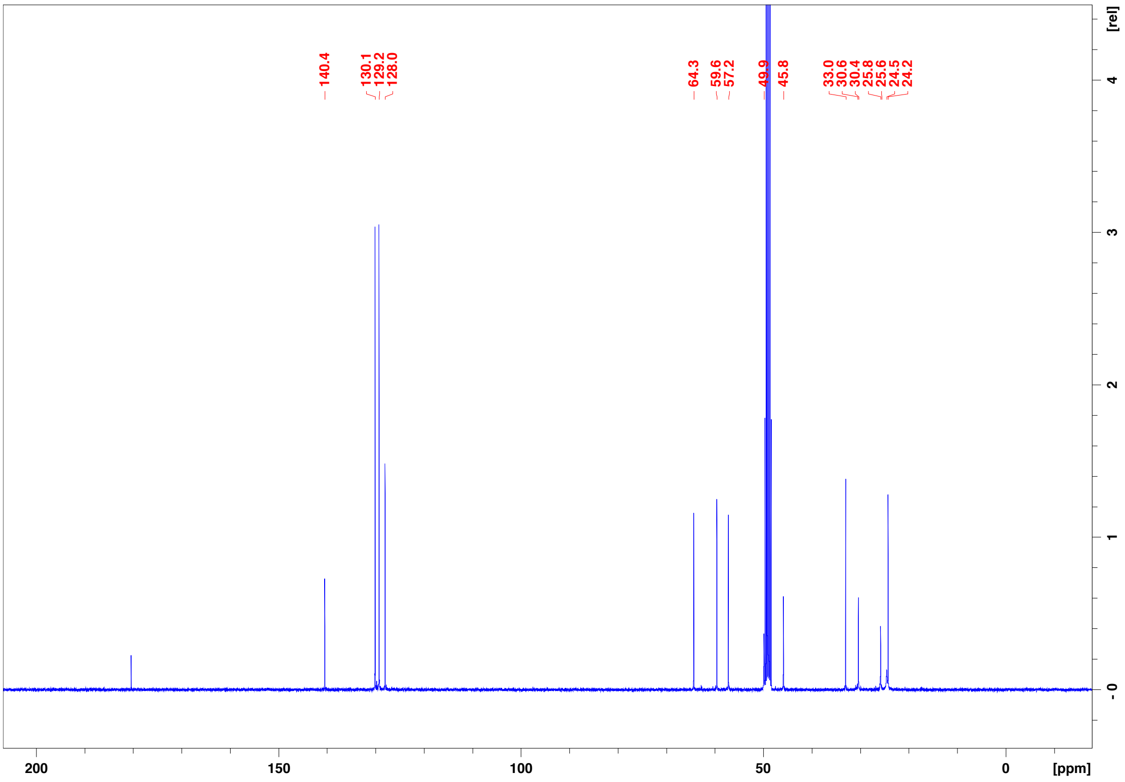

tert-butyl-6-oxodecahydro-1H-pyrido[3,2-b]azepine-1-carboxylate ((±)-**41**)

^{1}H-NMR

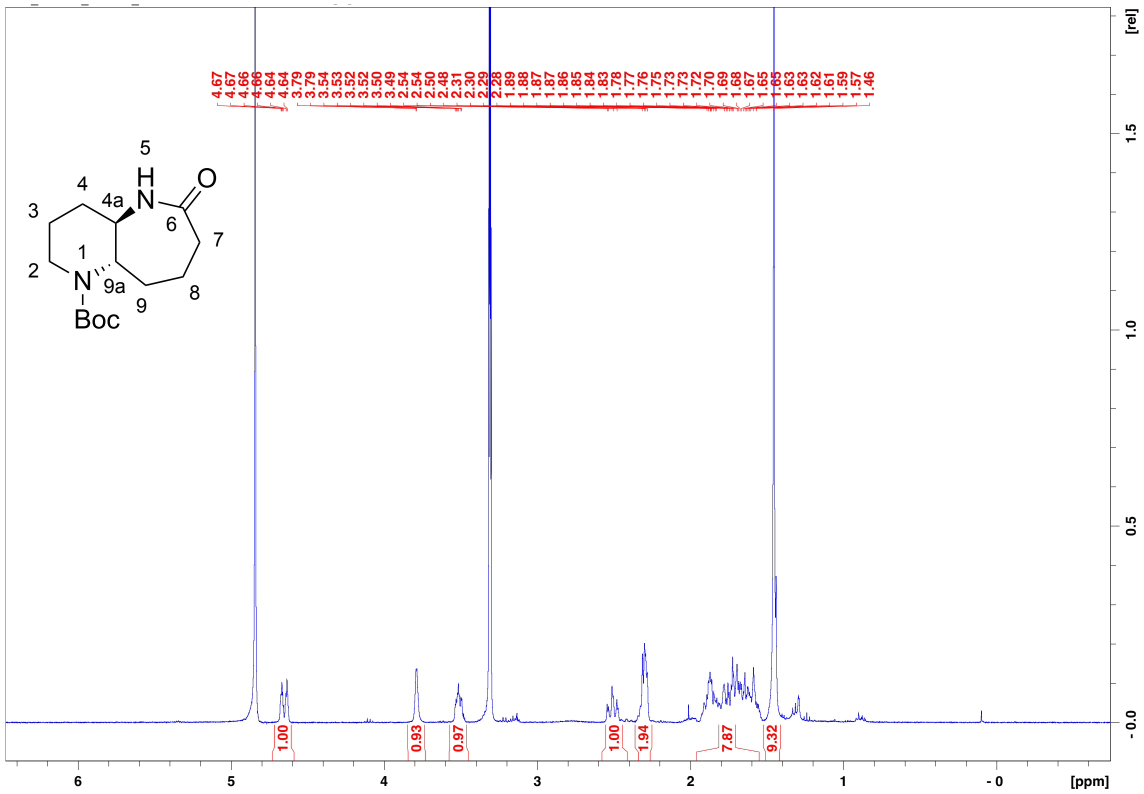

^{13}C-NMR

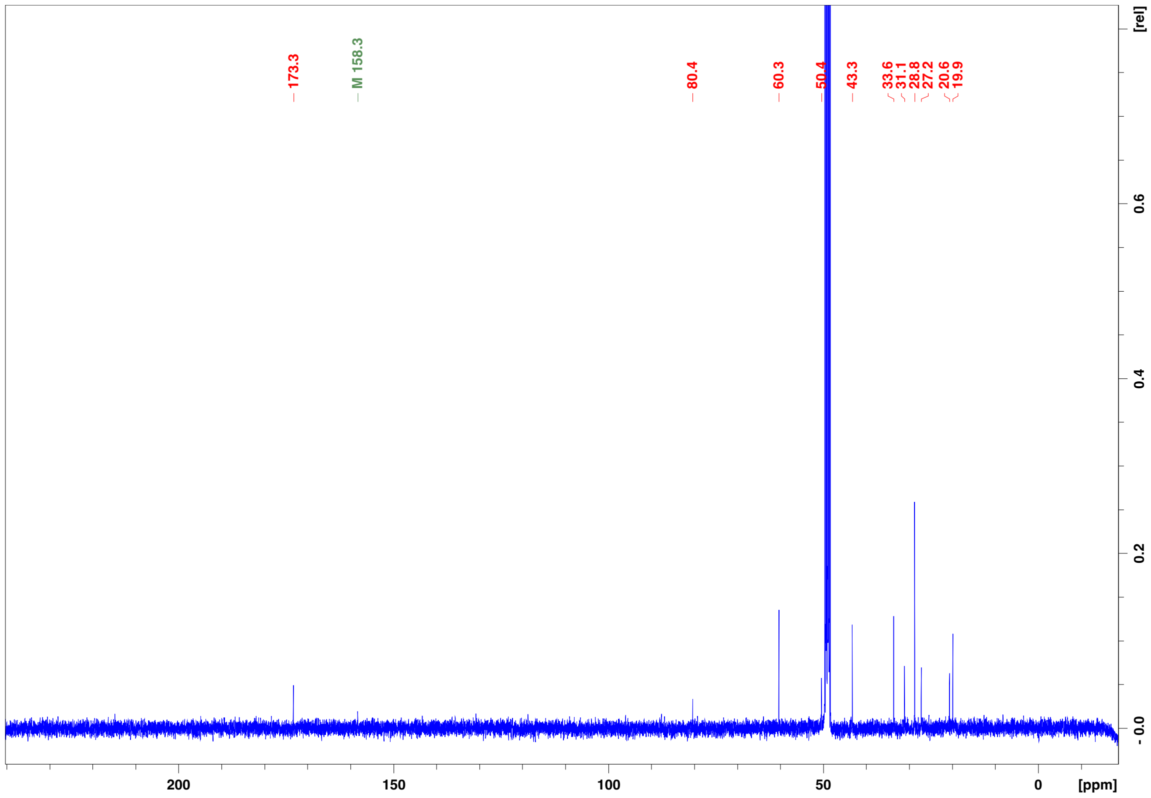

tert-butyl-decahydro-1H-pyrido[3,2-b]azepine-1-carboxylate ((±)-**42**)

^{1}H-NMR

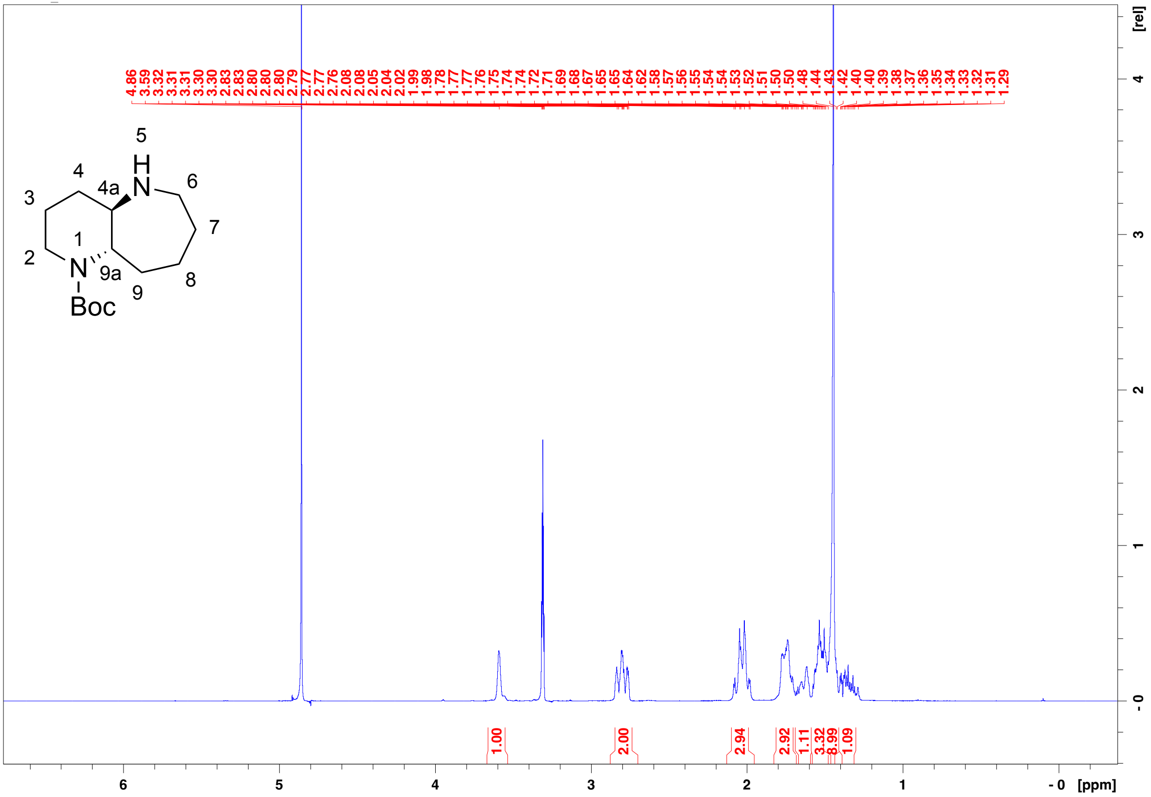

^{13}C-NMR

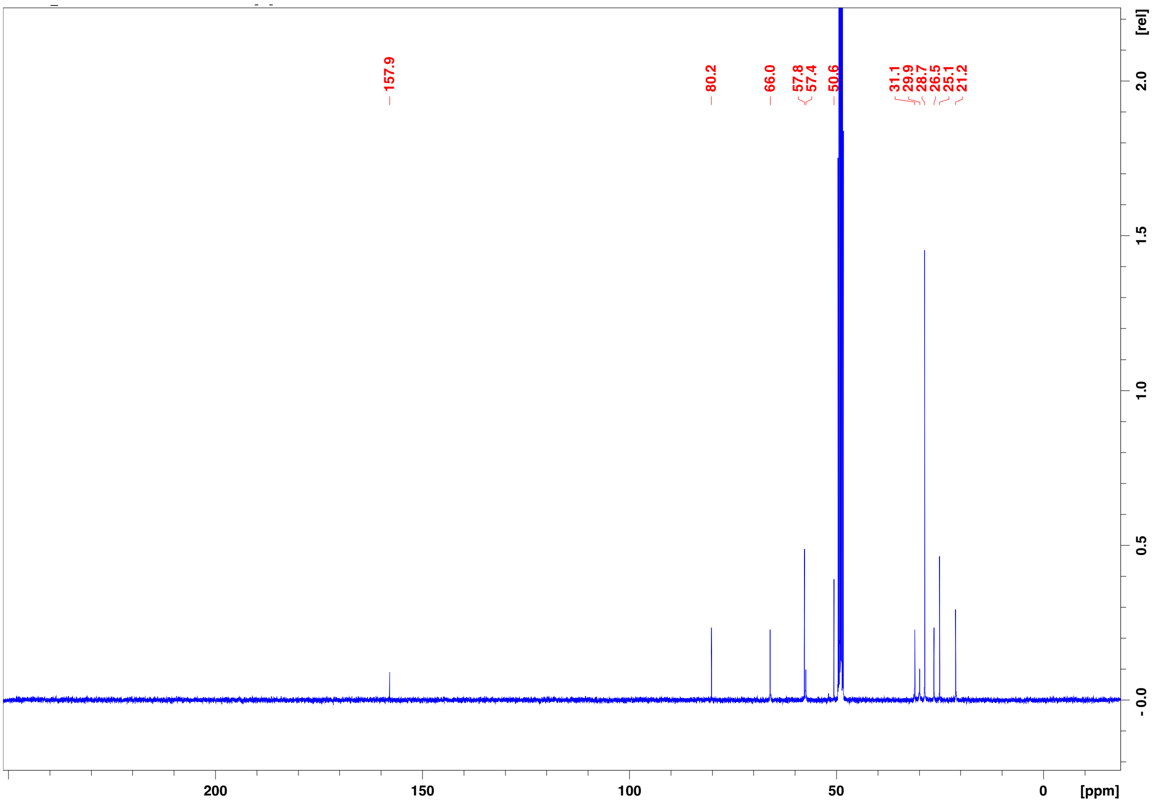

5-benzyldecahydro-1H-pyrido[3,2-b]azepine ((±)-**40b**)

^{1}H-NMR

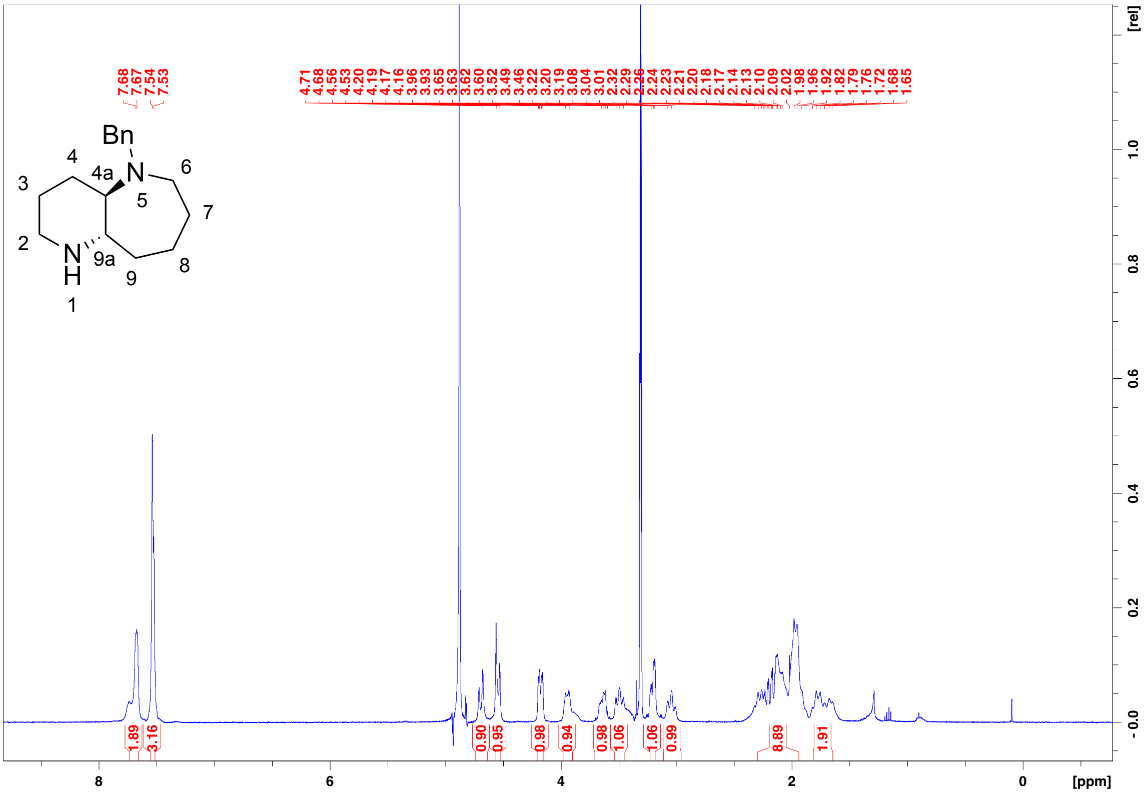

^{13}C-NMR

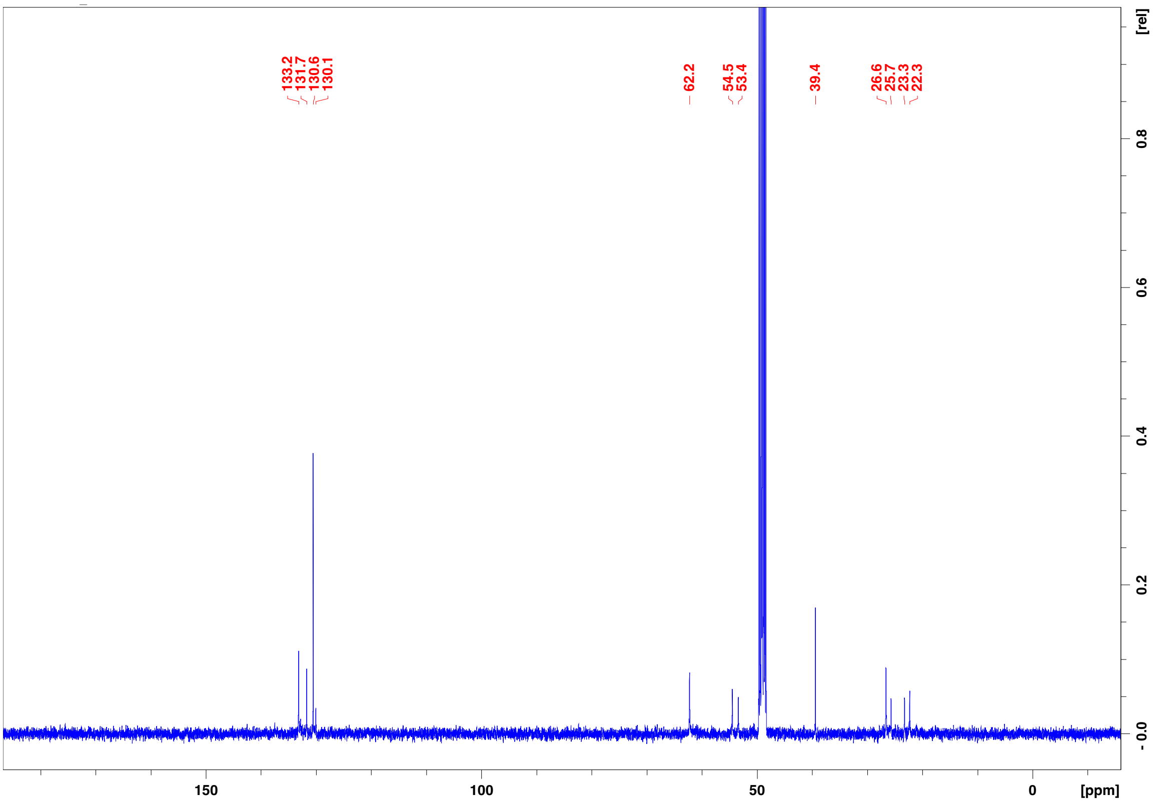

tert-butyl-decahydroazepino[3,2-*b*]azepine-1(2H)-carboxylate ((±)-**43**)

^{1}H-NMR

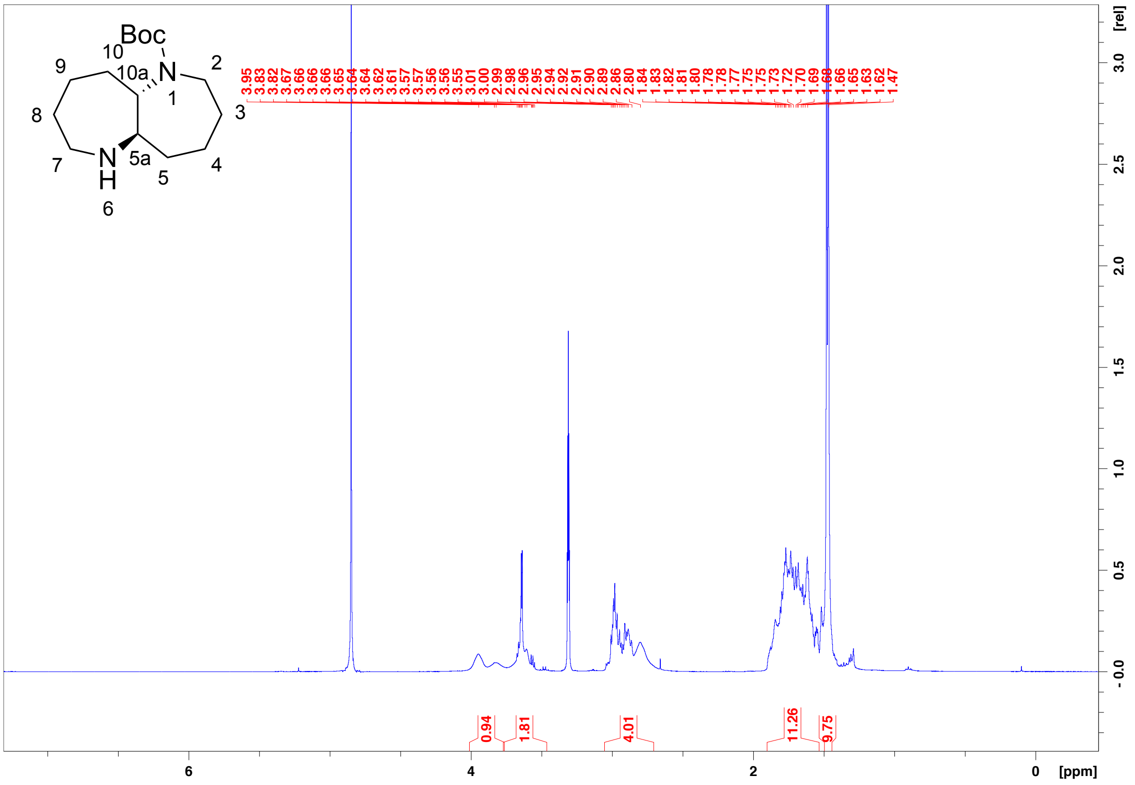

^{13}C-NMR

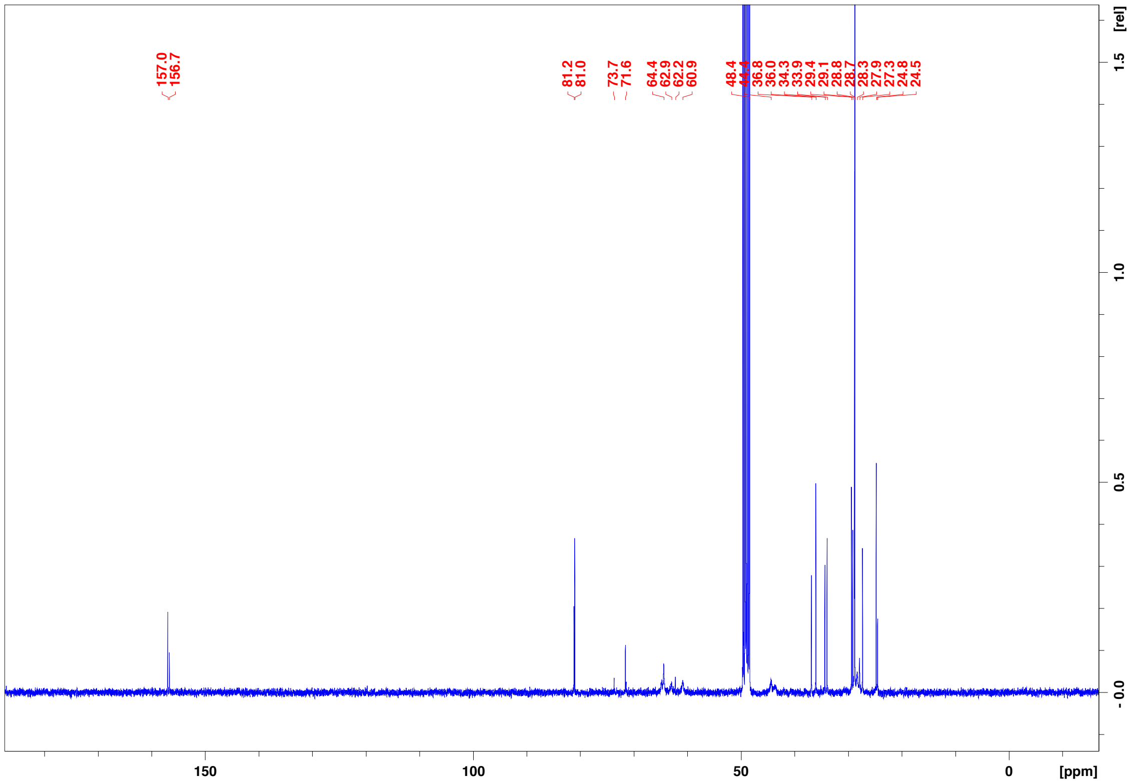

6-benzyldodecahydroazepino[3,2-b]azepine ((±)-**44b**)

^{1}H-NMR

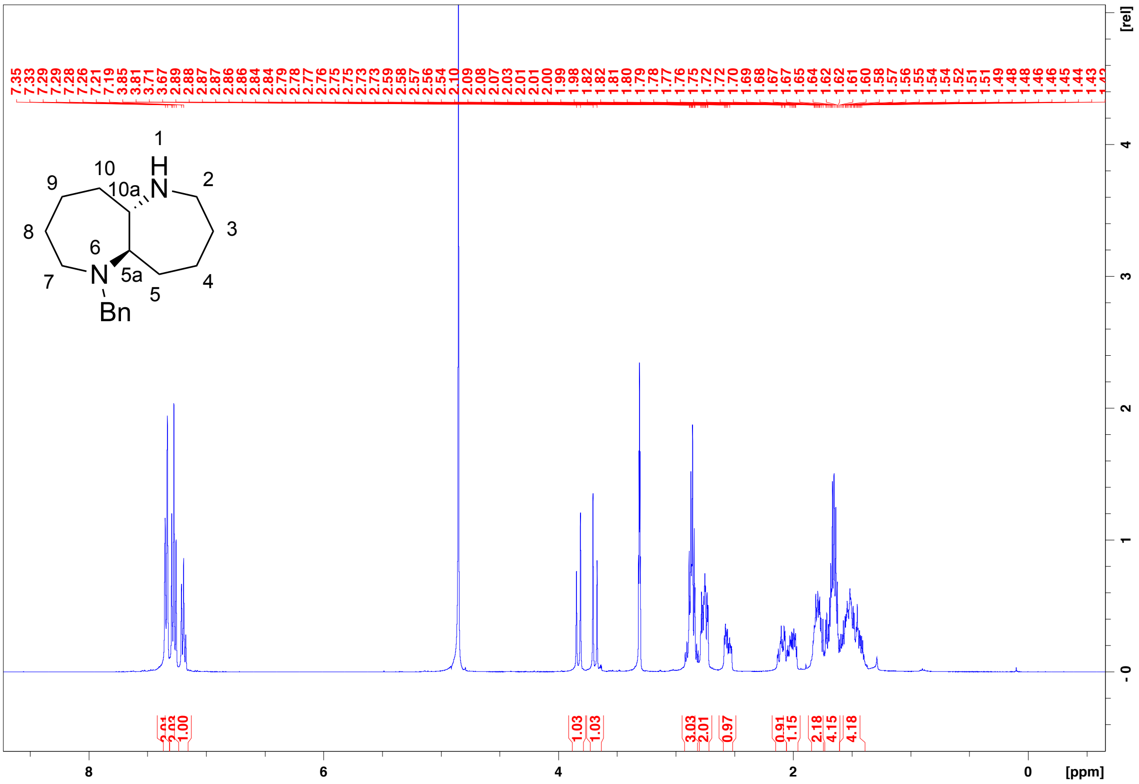

^{13}C-NMR

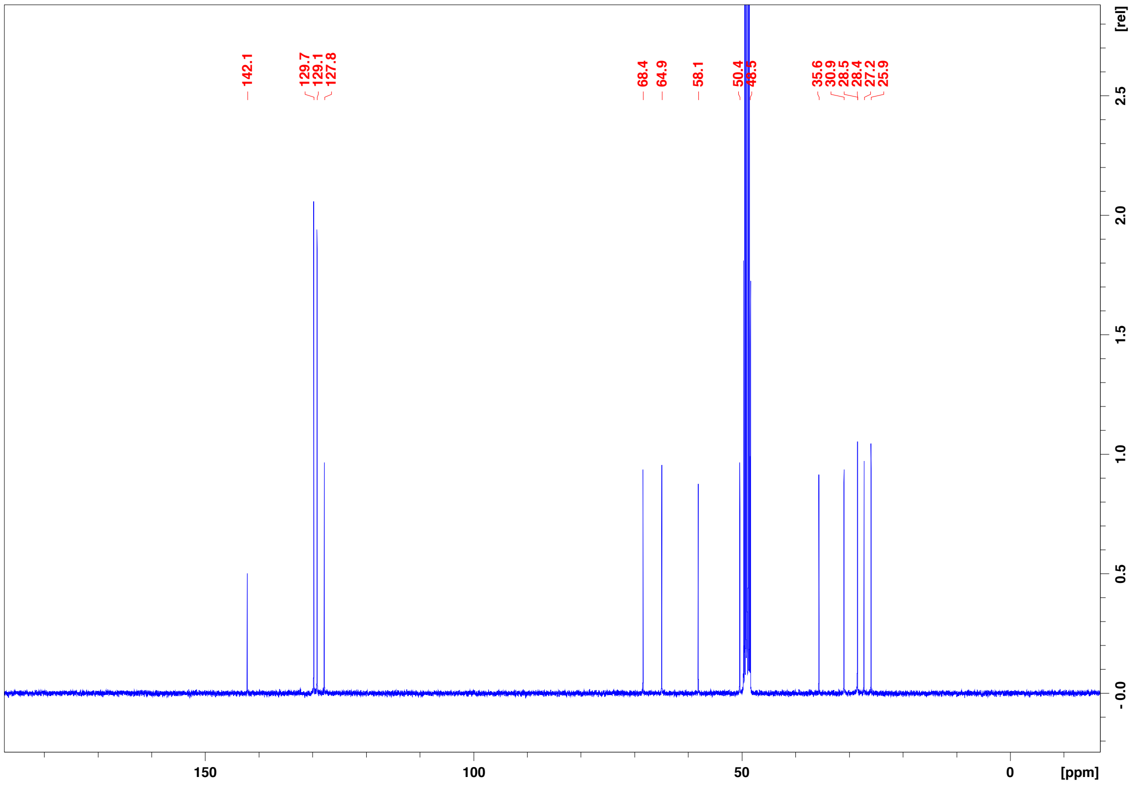

1-benzhydryloctahydro-4H-indol-4-one ((±)-**45**)

^{1}H-NMR

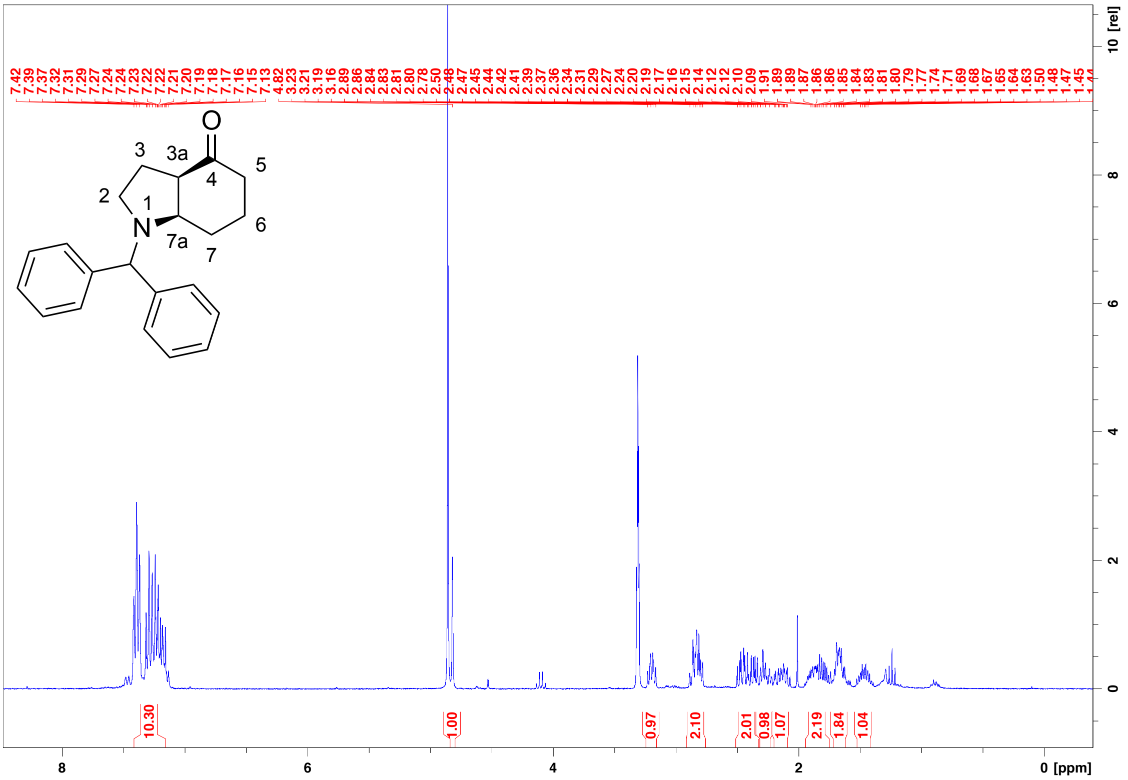

^{13}C-NMR

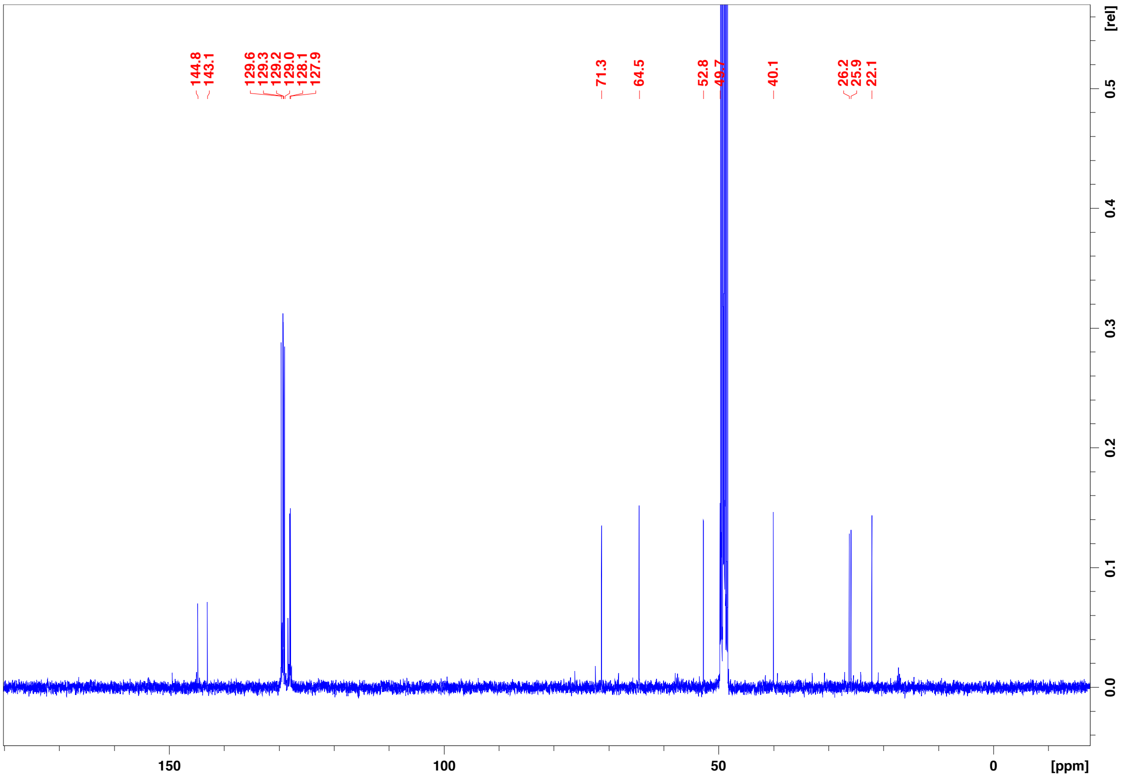

1-benzhydryloctahydro-4H-indol-4-one ((±)-**46**)

^{1}H-NMR

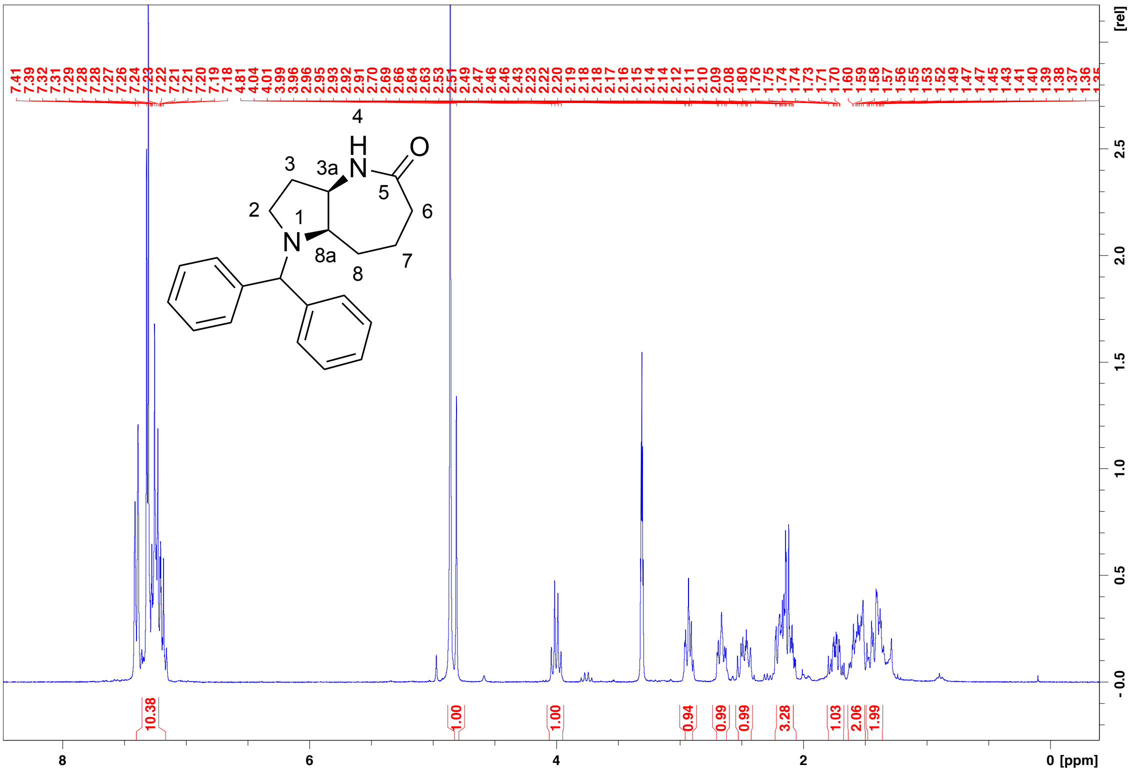

^{13}C-NMR

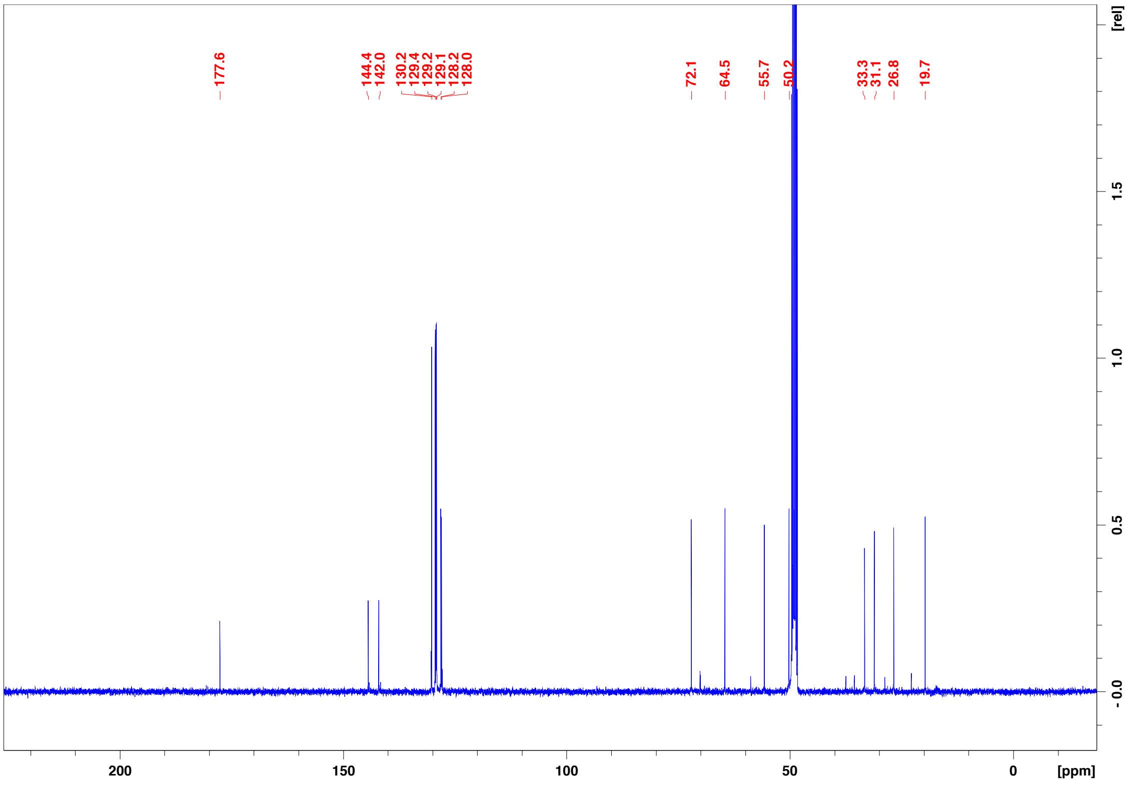

1-benzhydryldecahydropyrrolo[3,2-b]azepine ((±)-**47**)

^{1}H-NMR

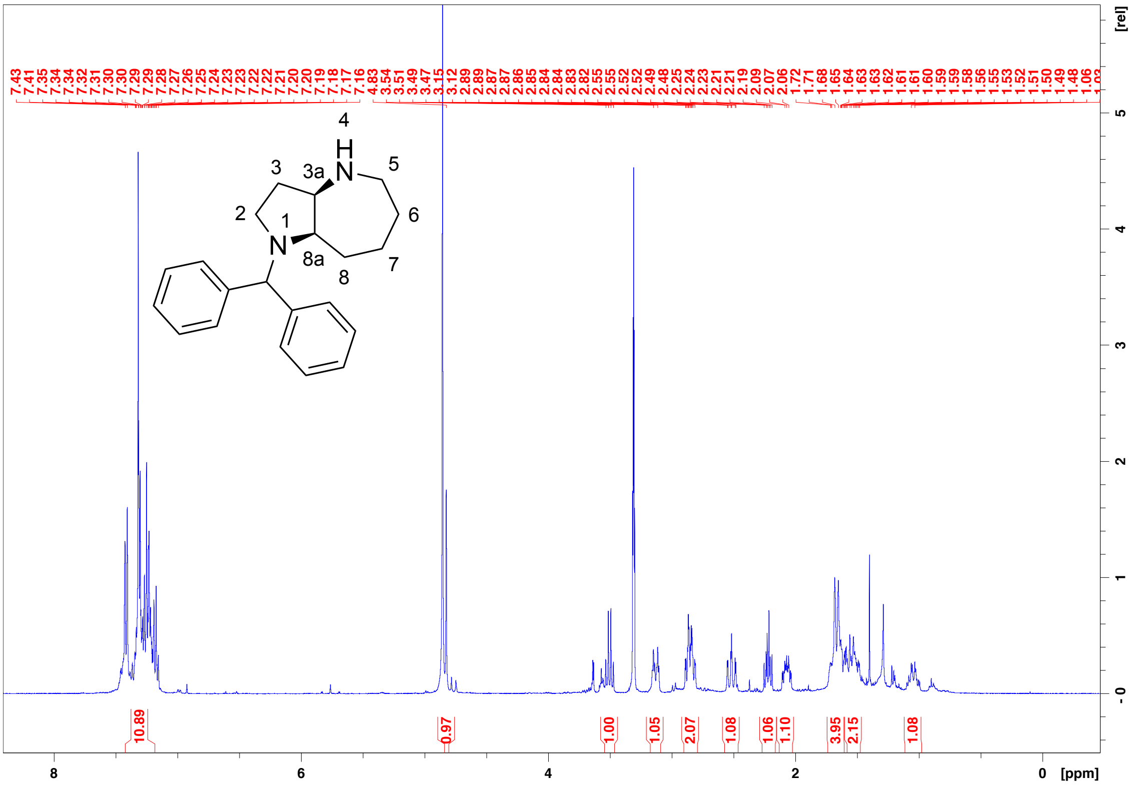

^{13}C-NMR

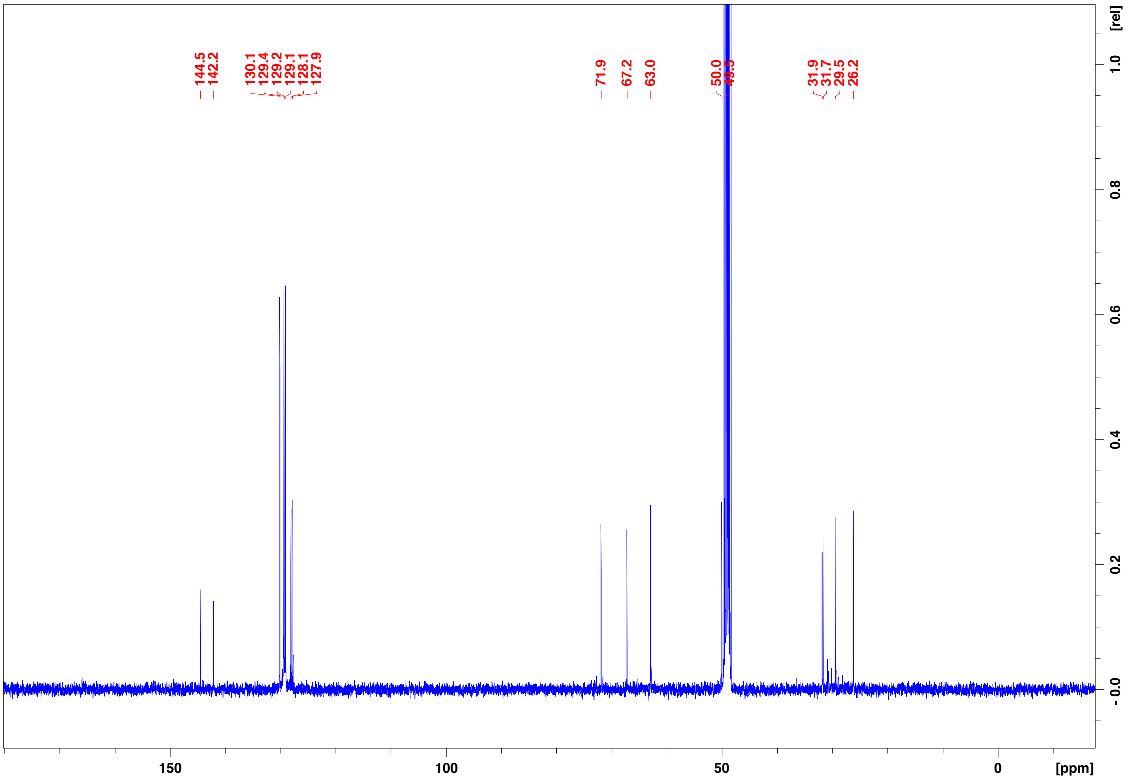

6-methoxy-2,3,4,5-tetrahydro-1H-benzo[b]azepine (**48**)

^{1}H-NMR

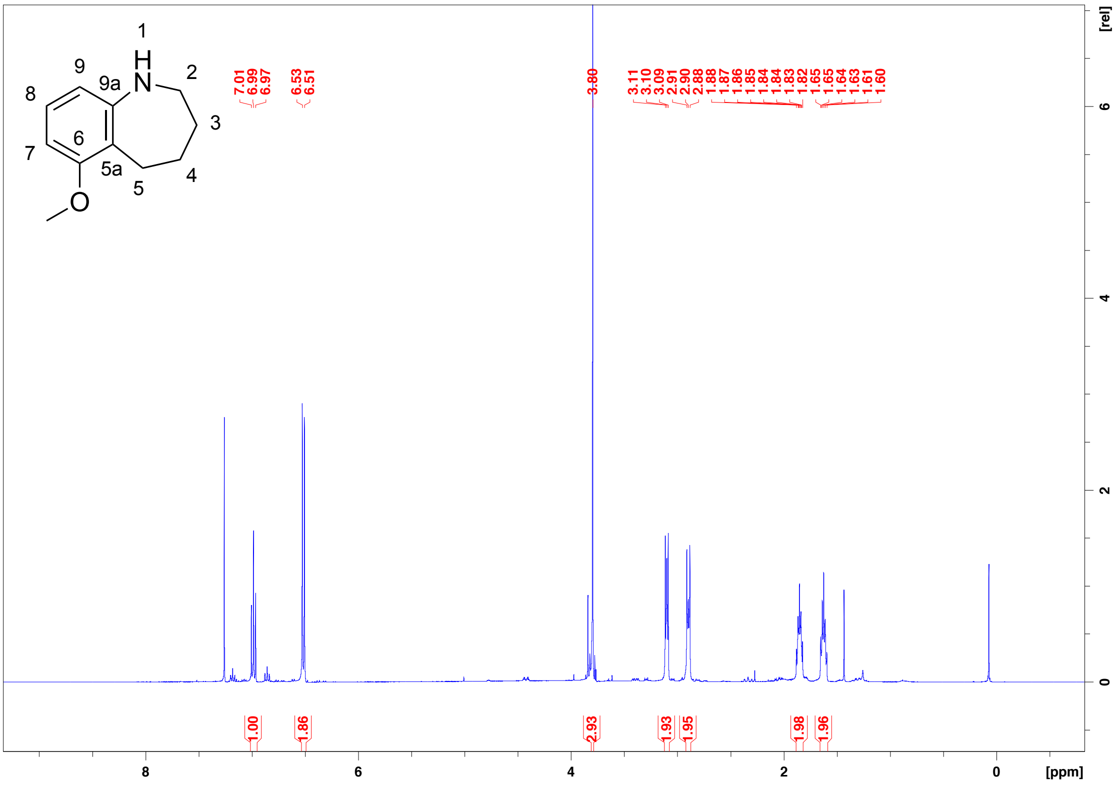

^{13}C-NMR

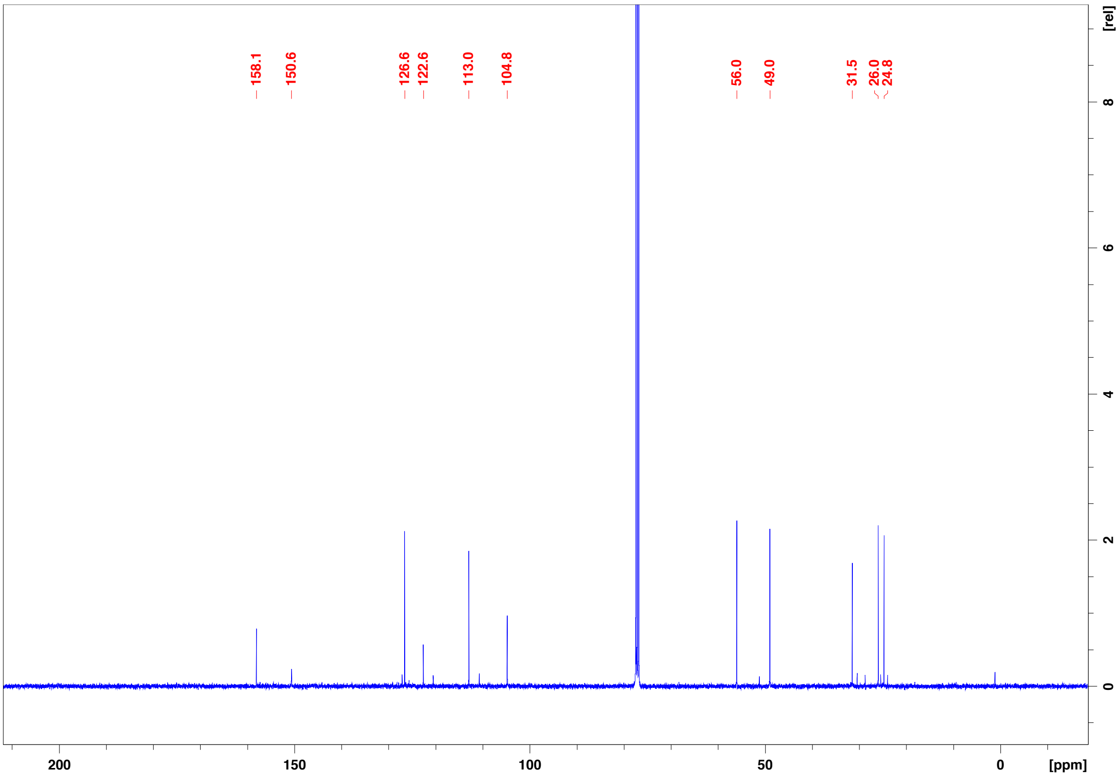

trifluoro-1-(6-methoxy-2,3,4,5-tetrahydro-1H-benzo[b]azepin-1-yl)ethanone (**49**)

^{1}H-NMR

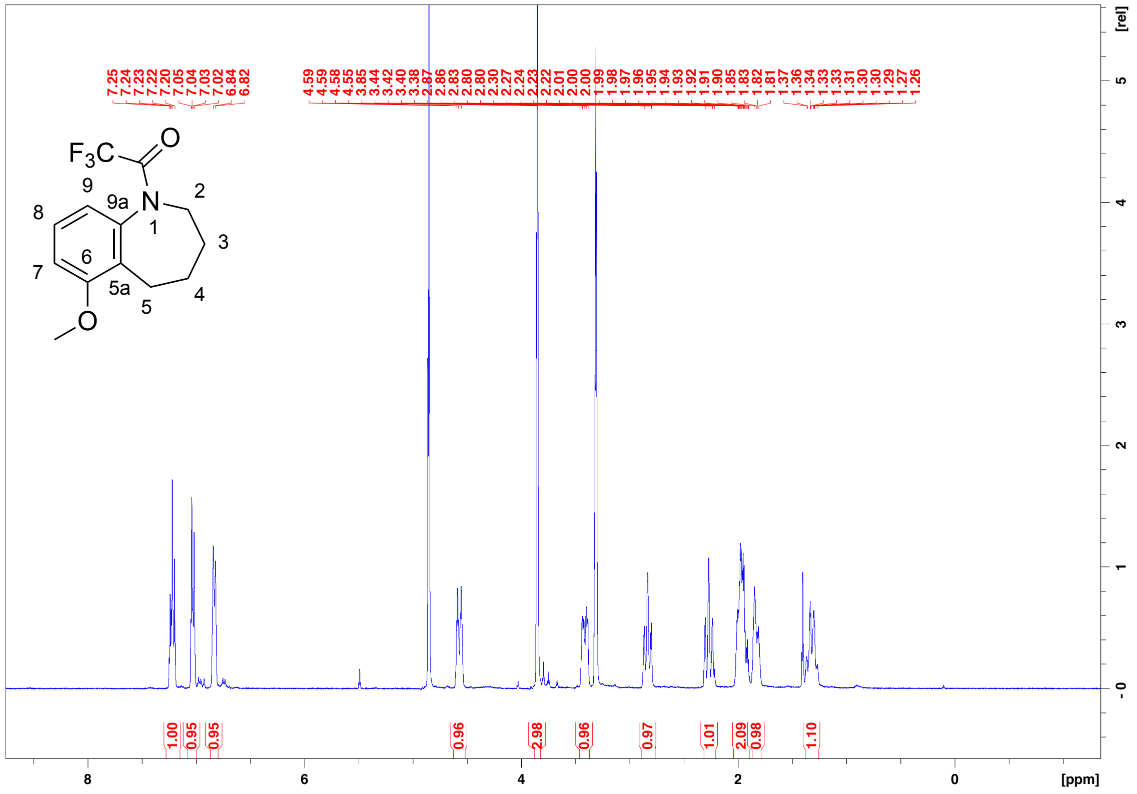

^{13}C-NMR

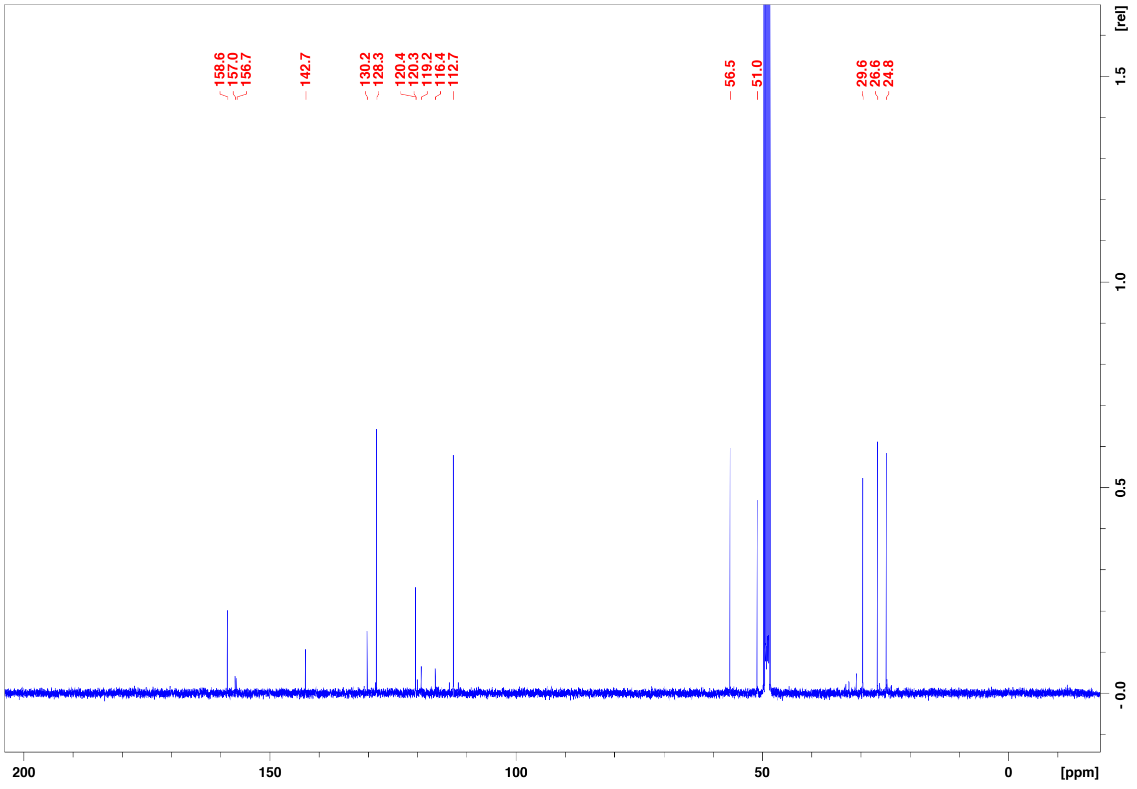

trifluoro-1-(6-hydroxy-2,3,4,5-tetrahydro-1H-benzo[b]azepin-1-yl)ethanone (**50**)

^{1}H-NMR

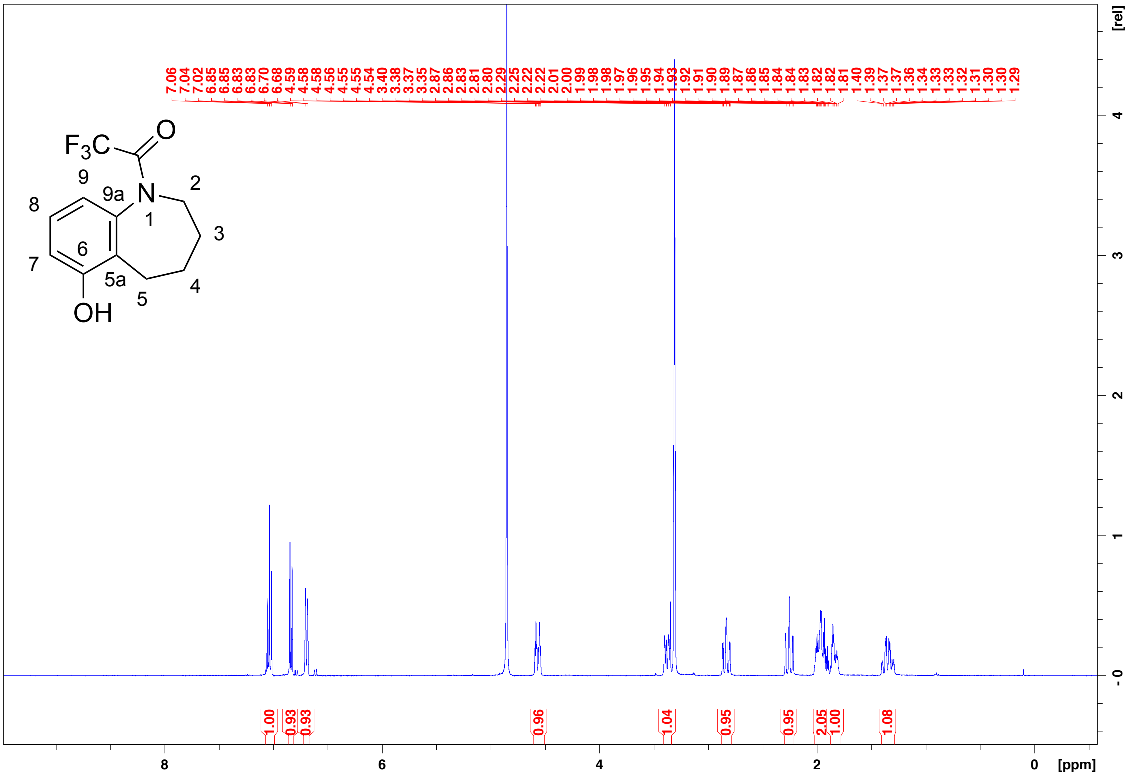

^{13}C-NMR

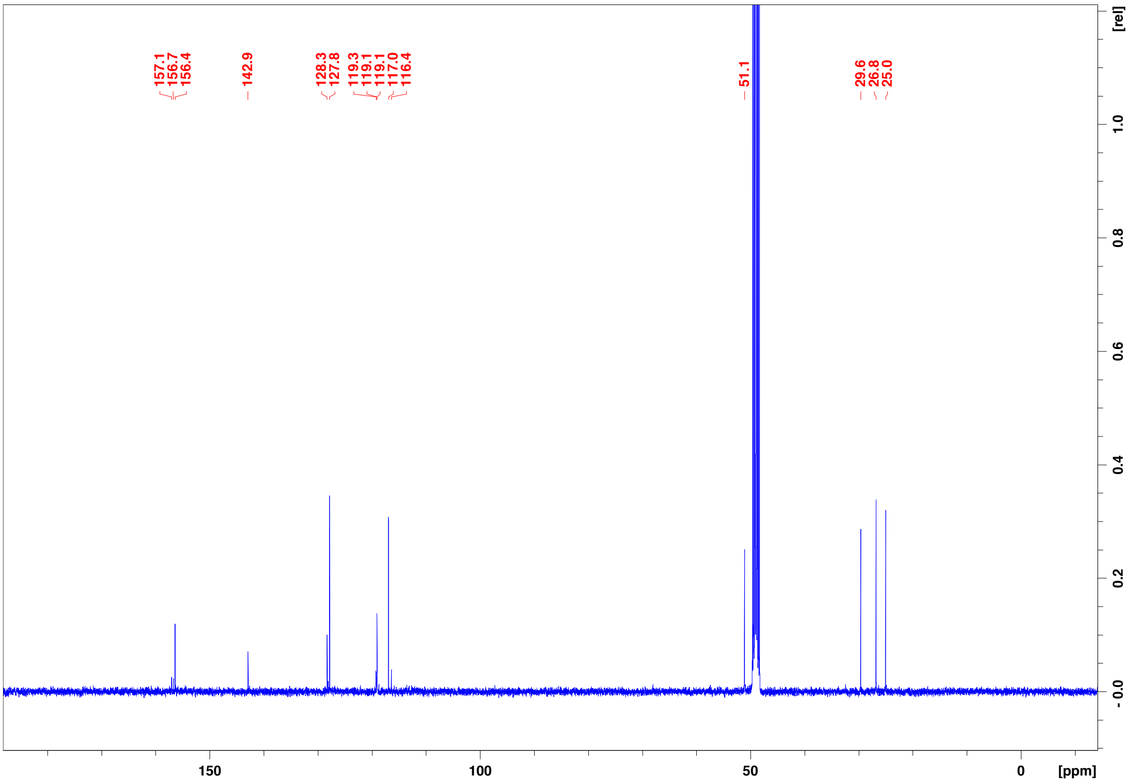

trifluoro-1-(6-hydroxydecahydro-1H-benzo[b]azepin-1-yl)ethenone ((±)-**51**)

^{1}H-NMR

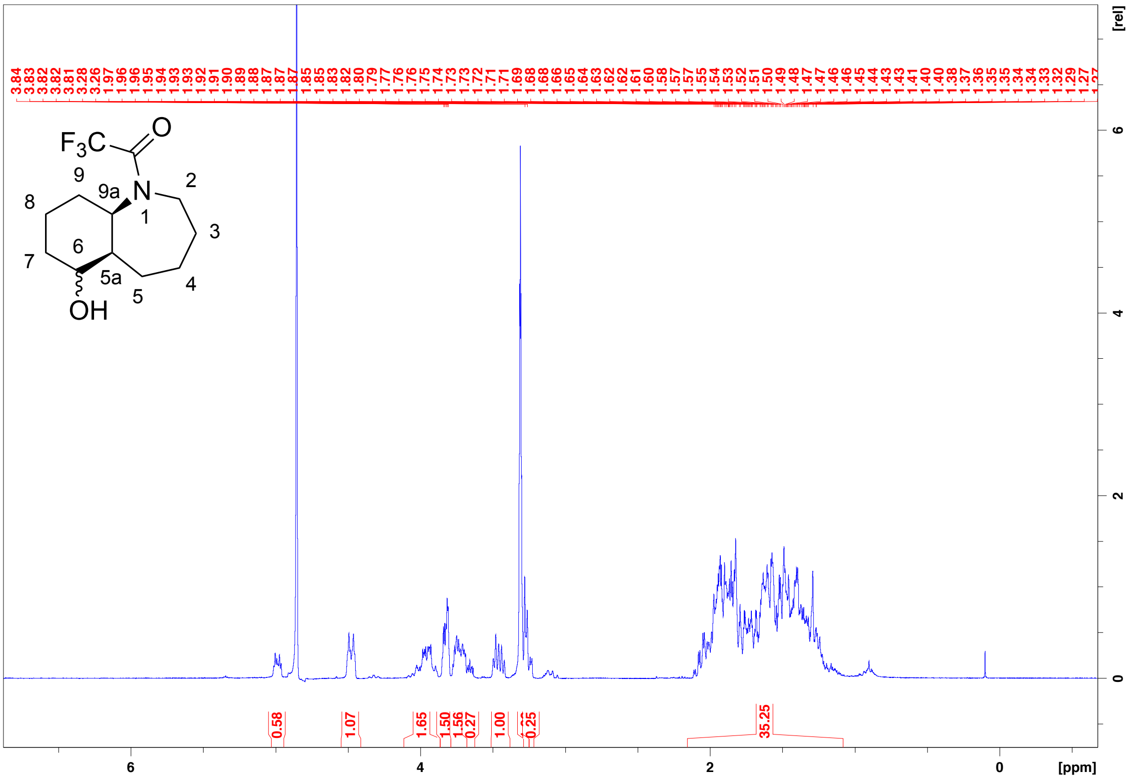

^{13}C-NMR

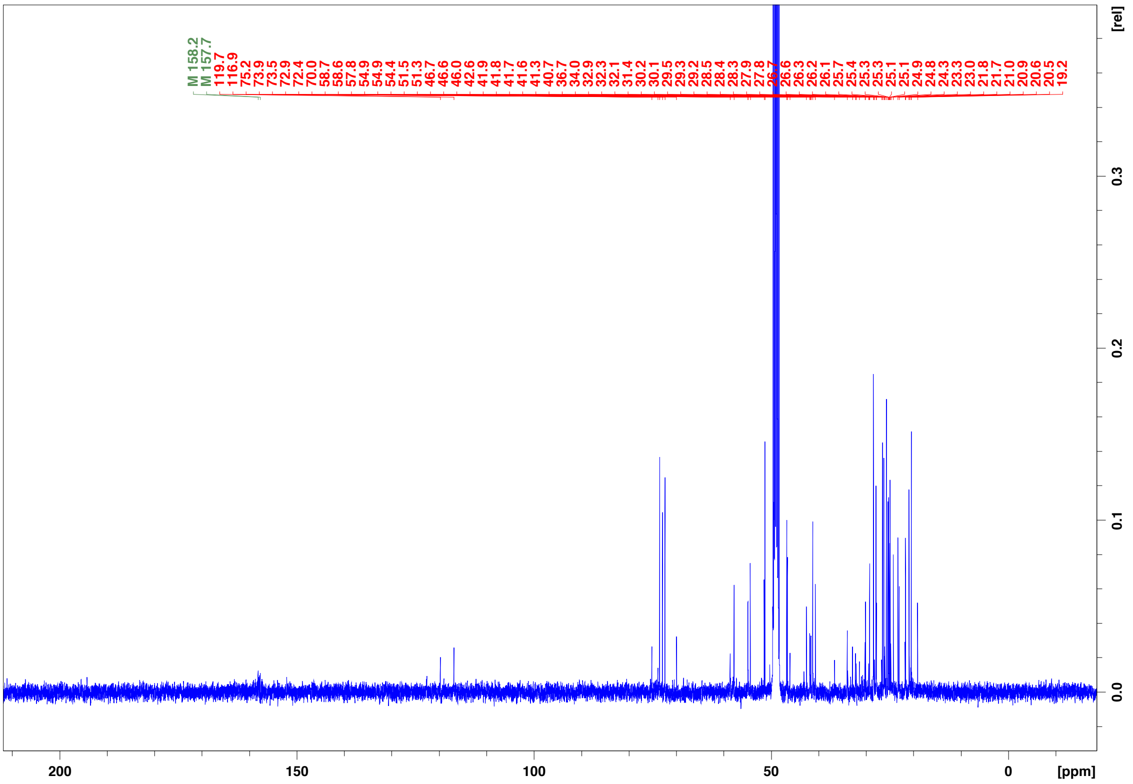

<u>*tert*-butyl-6-hydroxydecahydro-1H-benzo[b]azepine-1-carboxylate ((±)-**52**)</u>

^{1}H-NMR

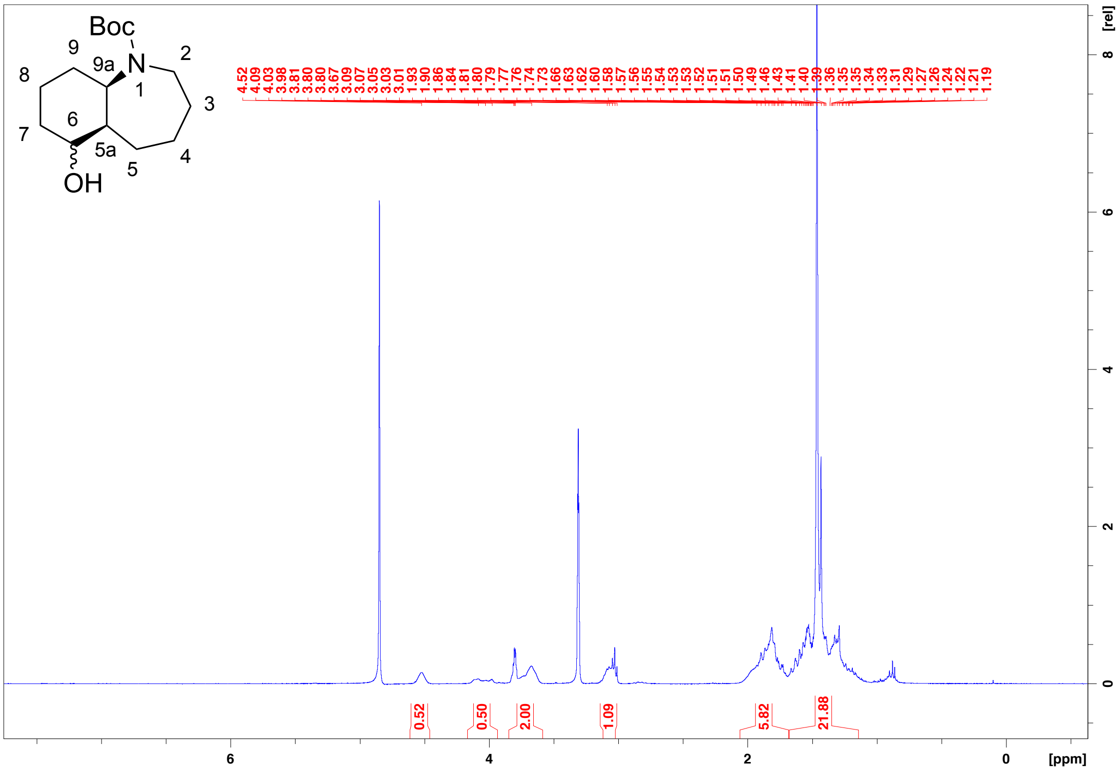

^{13}C-NMR

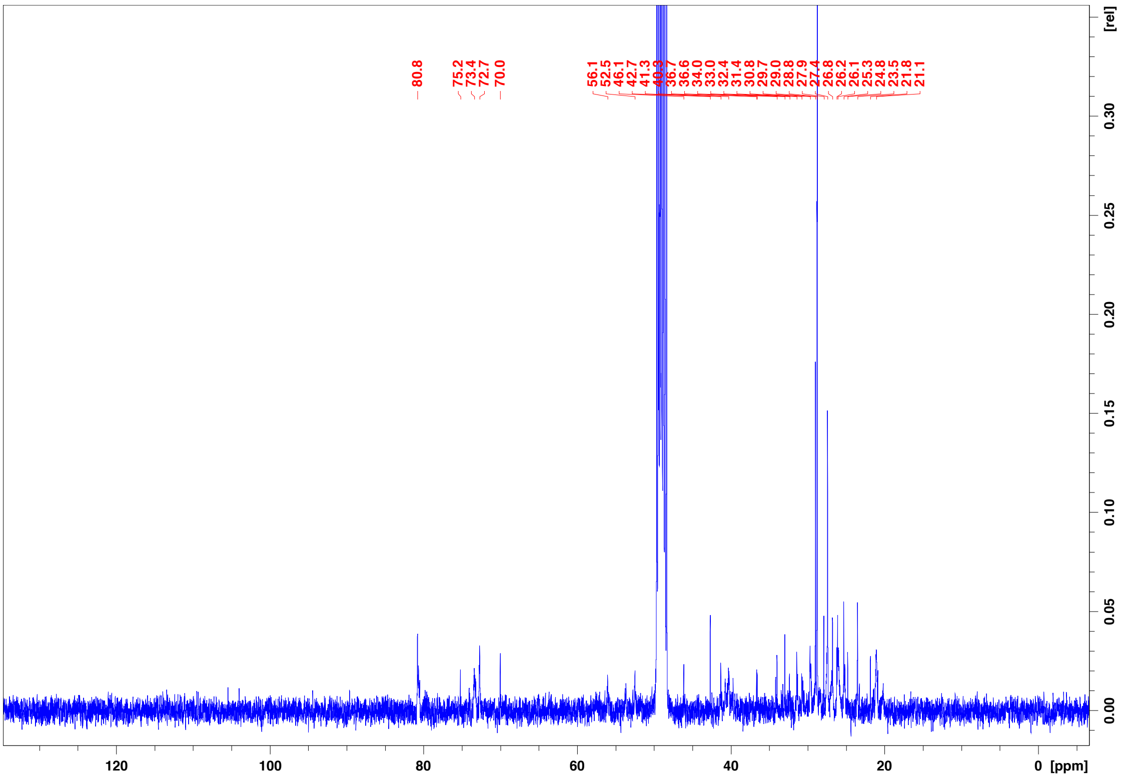

tert-butyl-6-oxodecahydro-1H-benzo[b]azepine-1-carboxylate ((±)-**53**)

^{1}H-NMR

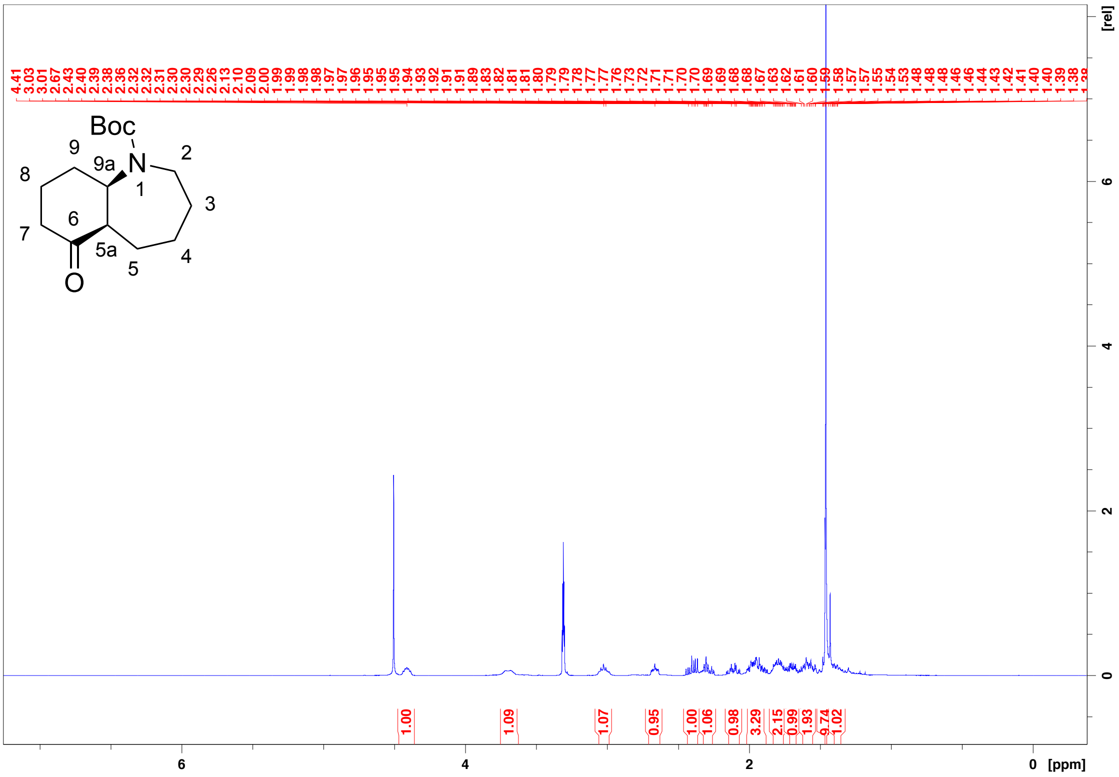

^{13}C-NMR

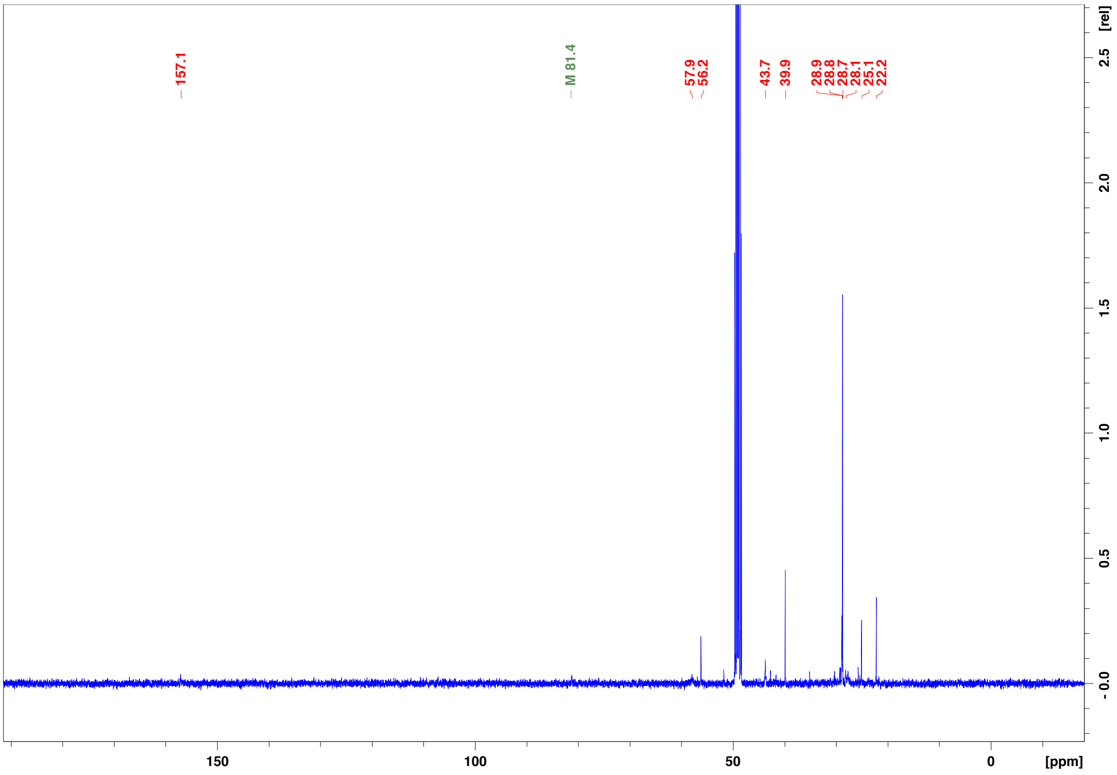

tert-butyl- (E)-6-(hydroxyimino)decahydro-1H-benzo[b]azepine-1-carboxylate ((±)-E-**54**)

^{1}H-NMR

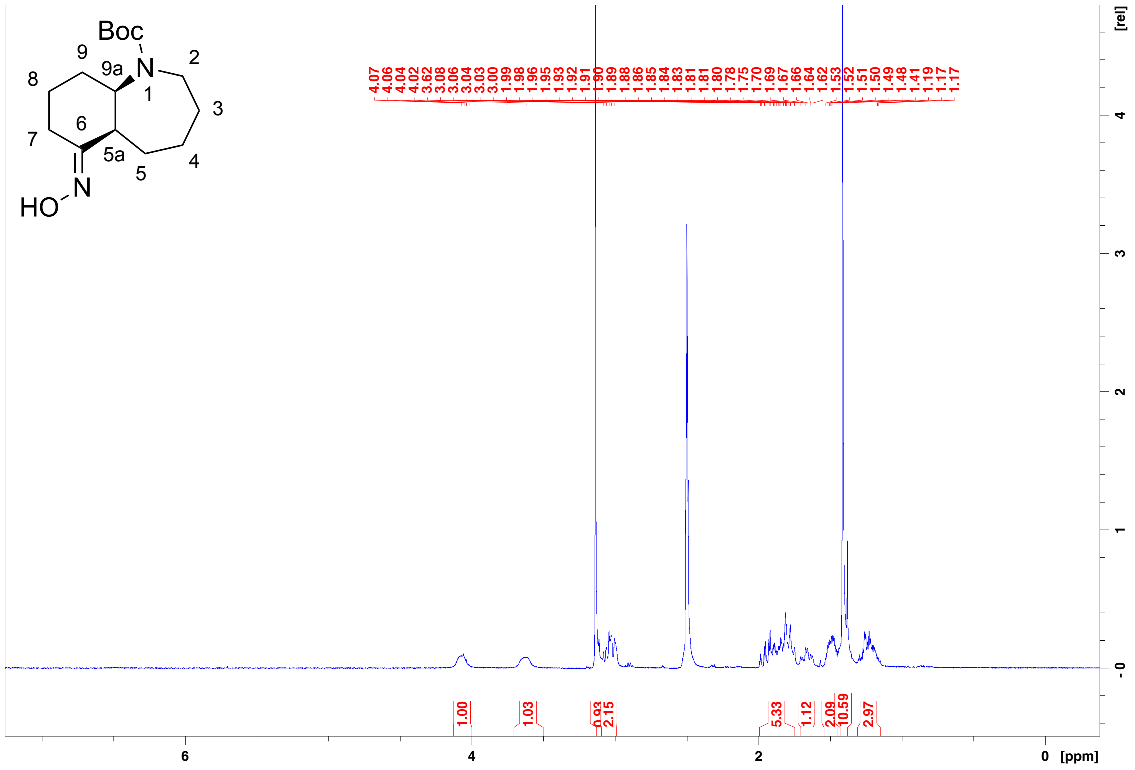

^{13}C-NMR

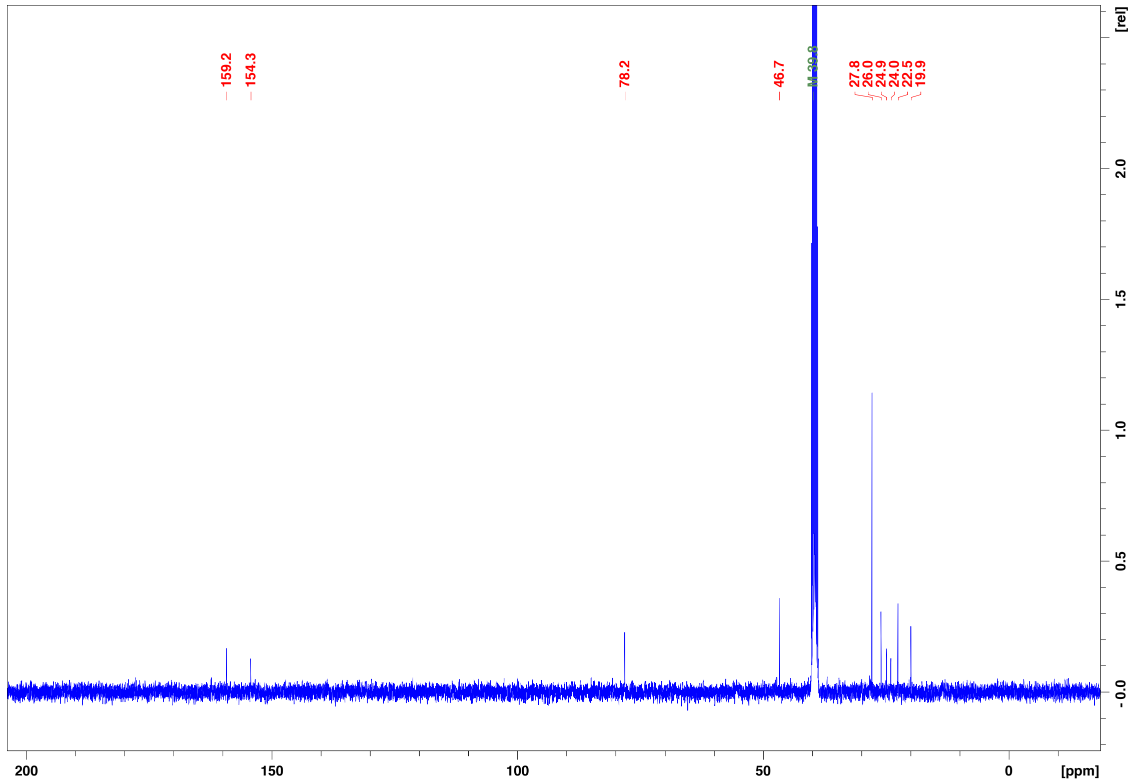

<u>*tert*-butyl-(*Z*)-6-(hydroxyimino)decahydro-1H-benzo[b]azepine-1-carboxylate ((±)-*Z*-**54**)</u>

^{1}H-NMR

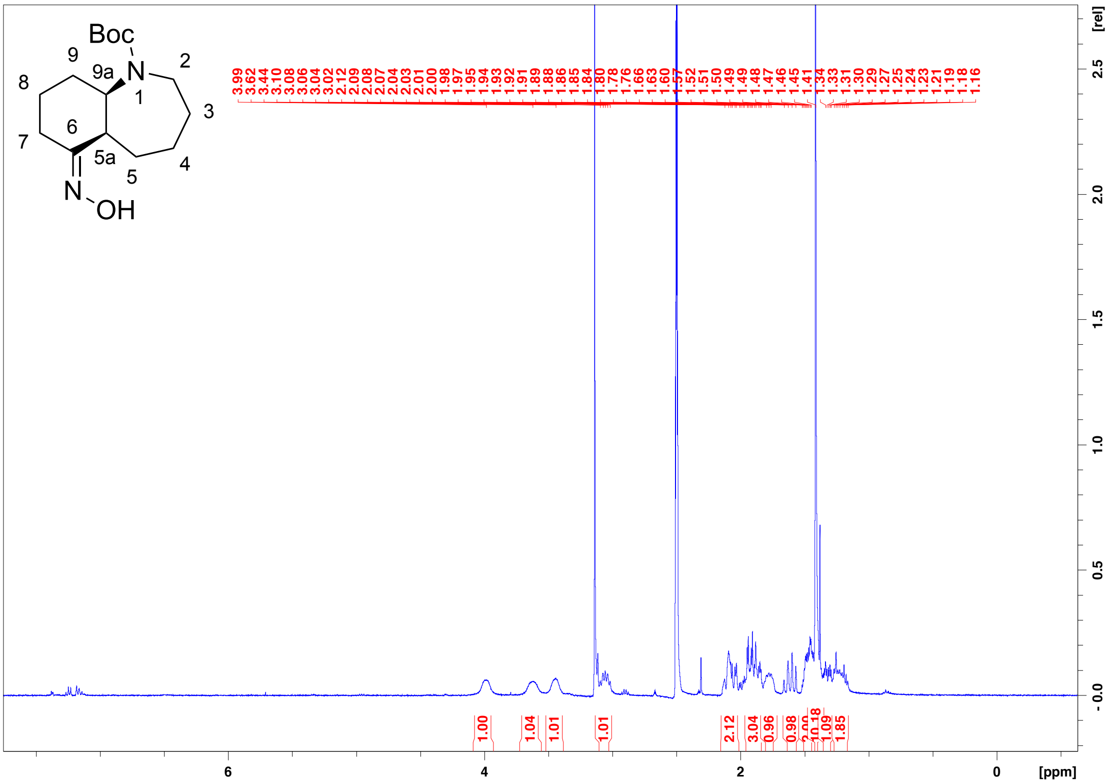

^{13}C-NMR

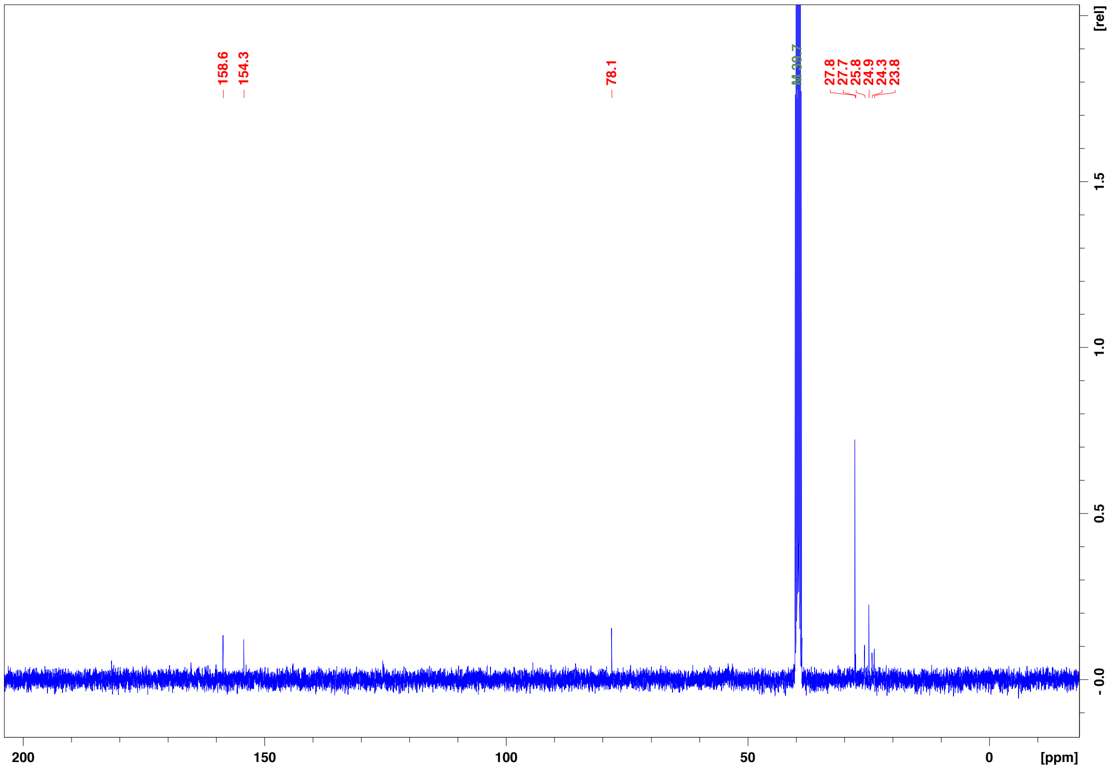

tert-butyl-(3a*R*,8a*R*)-4-(4-chlorobenzyl)octahydropyrrolo[3,2-b]azepine-1(2H)-carboxylate ((*R,R)*-**55**)

^{1}H-NMR

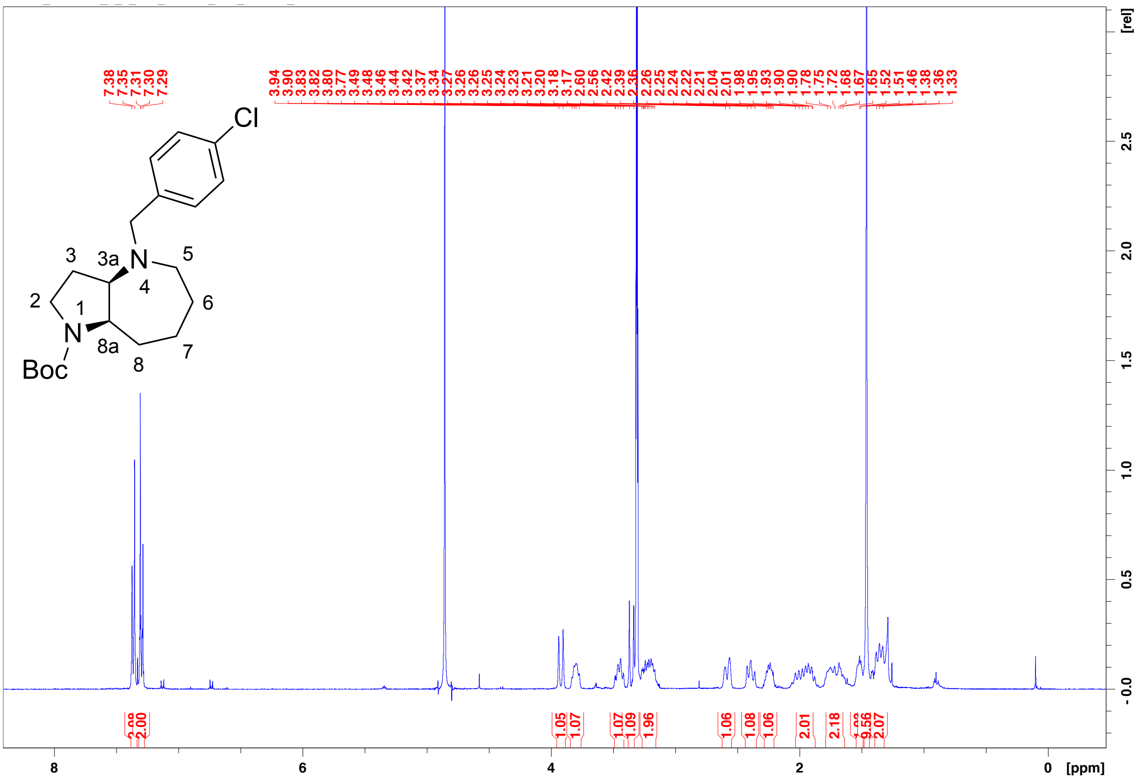

^{13}C-NMR

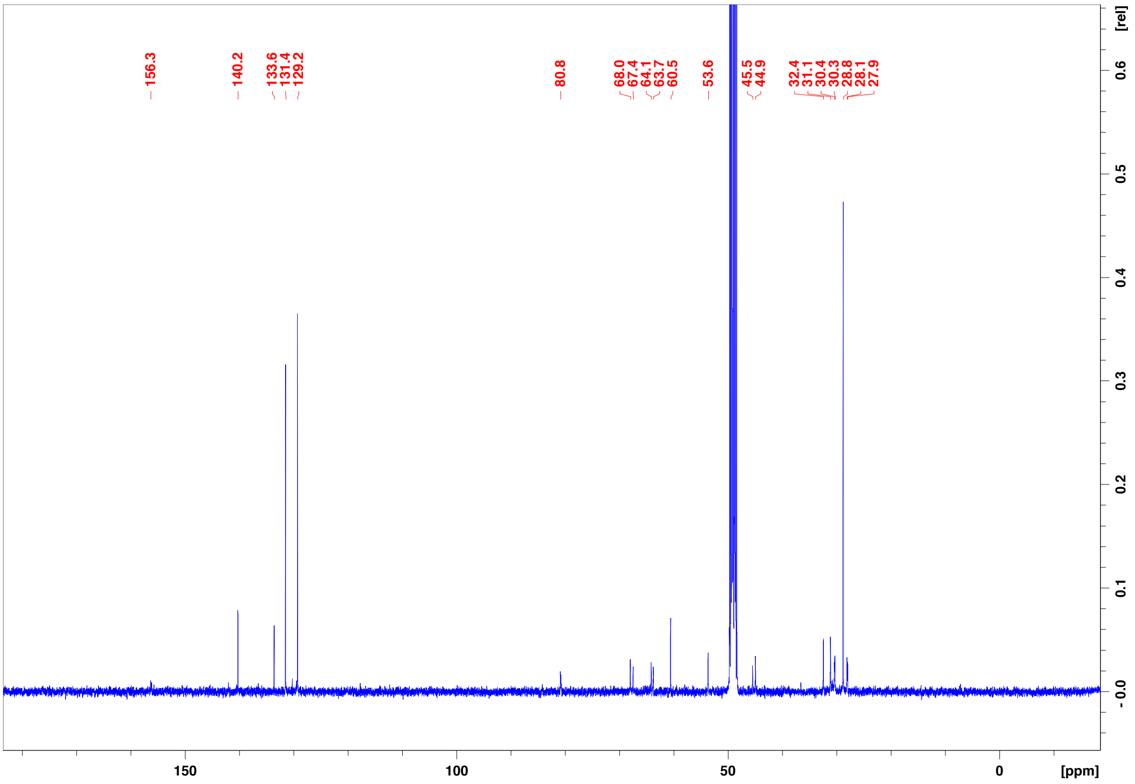

tert-butyl (3a*R*,8a*R*)-4-(3-chlorobenzyl)octahydropyrrolo[3,2-b]azepine-1(2*H*)-carboxylate ((*R,R*)-**56**)

^{1}H-NMR

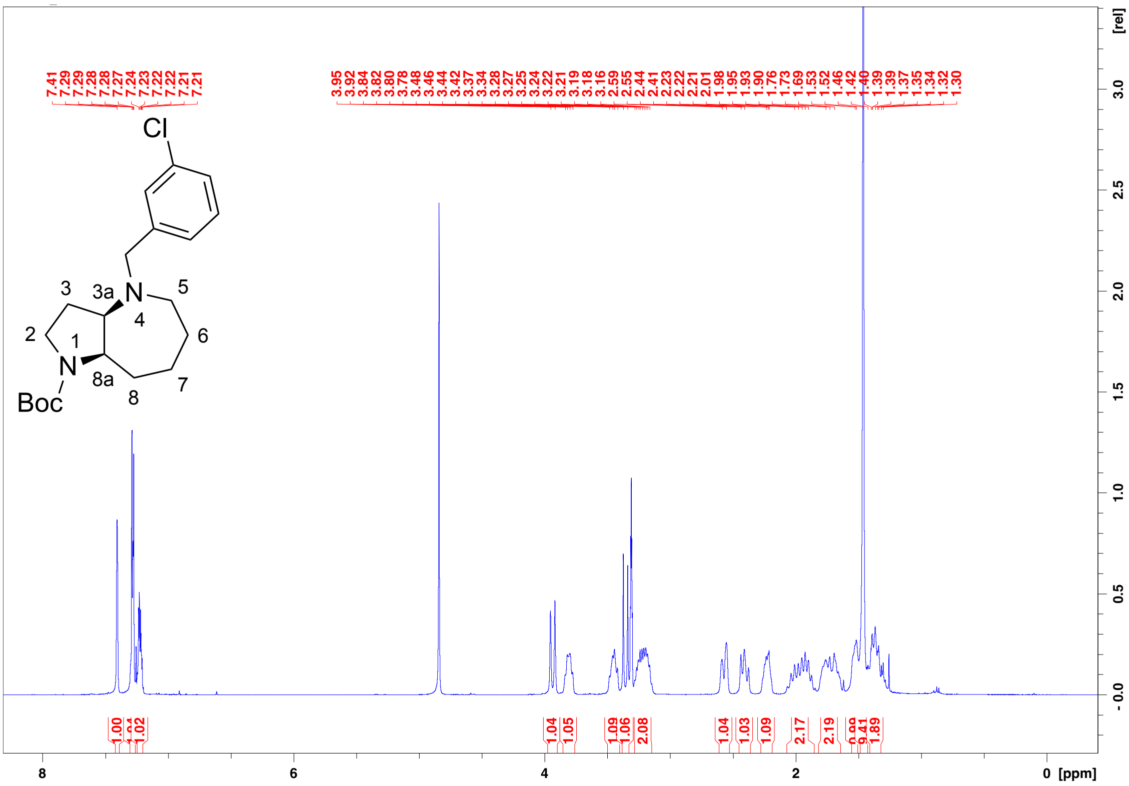

^{13}C-NMR

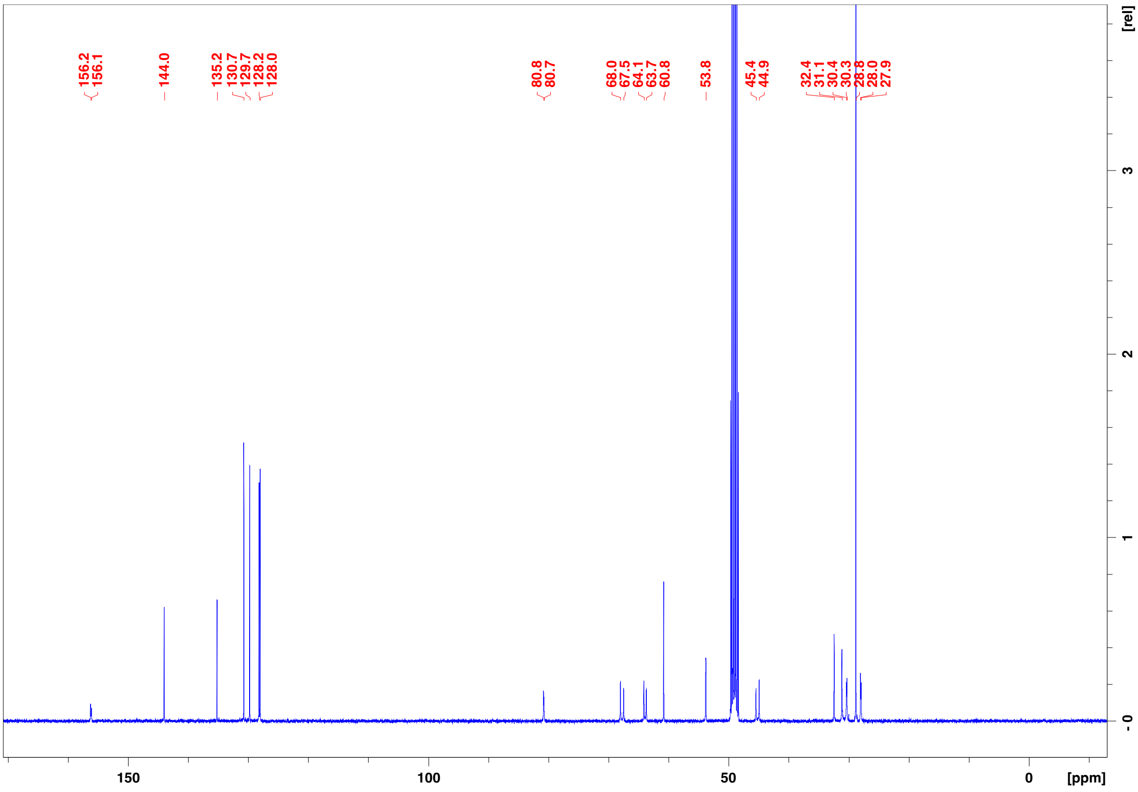

<u>*tert*-butyl (3a*R*,8a*R*)-4-(2-chlorobenzyl)octahydropyrrolo[3,2-b]azepine-1(2*H*)-carboxylate (*(R,R)*-**57**)</u>

^{1}H-NMR

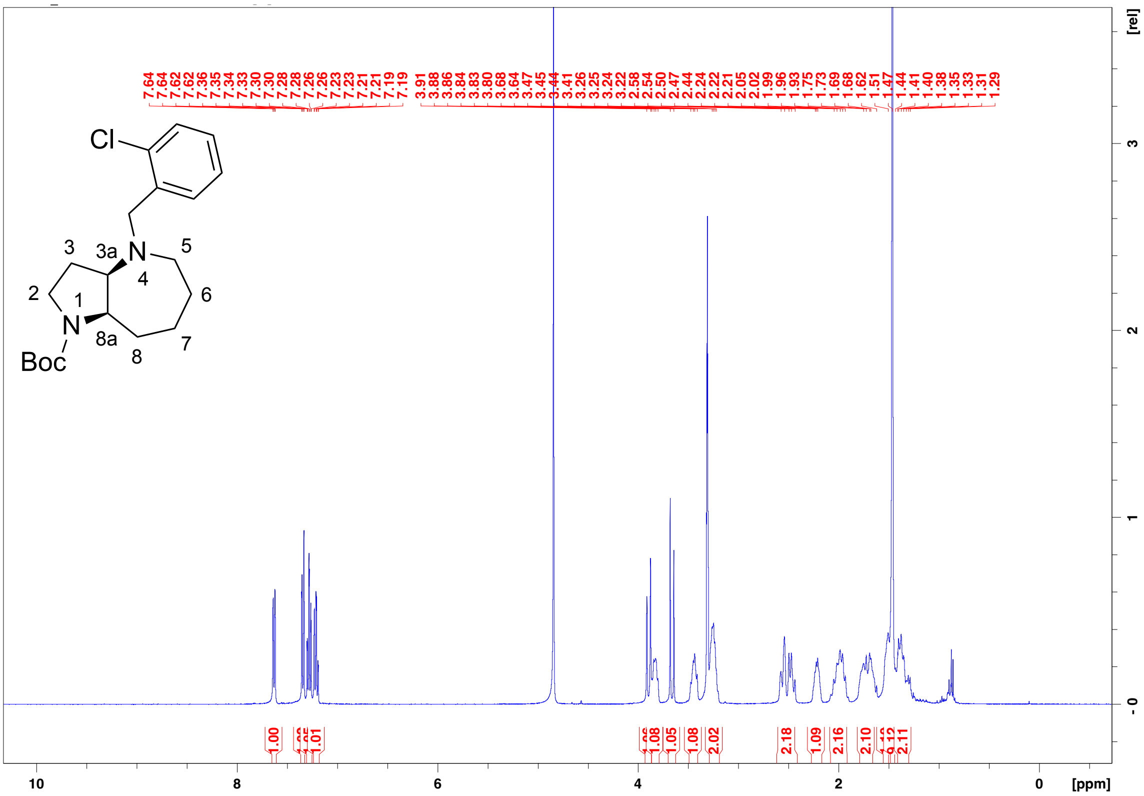

^{13}C-NMR

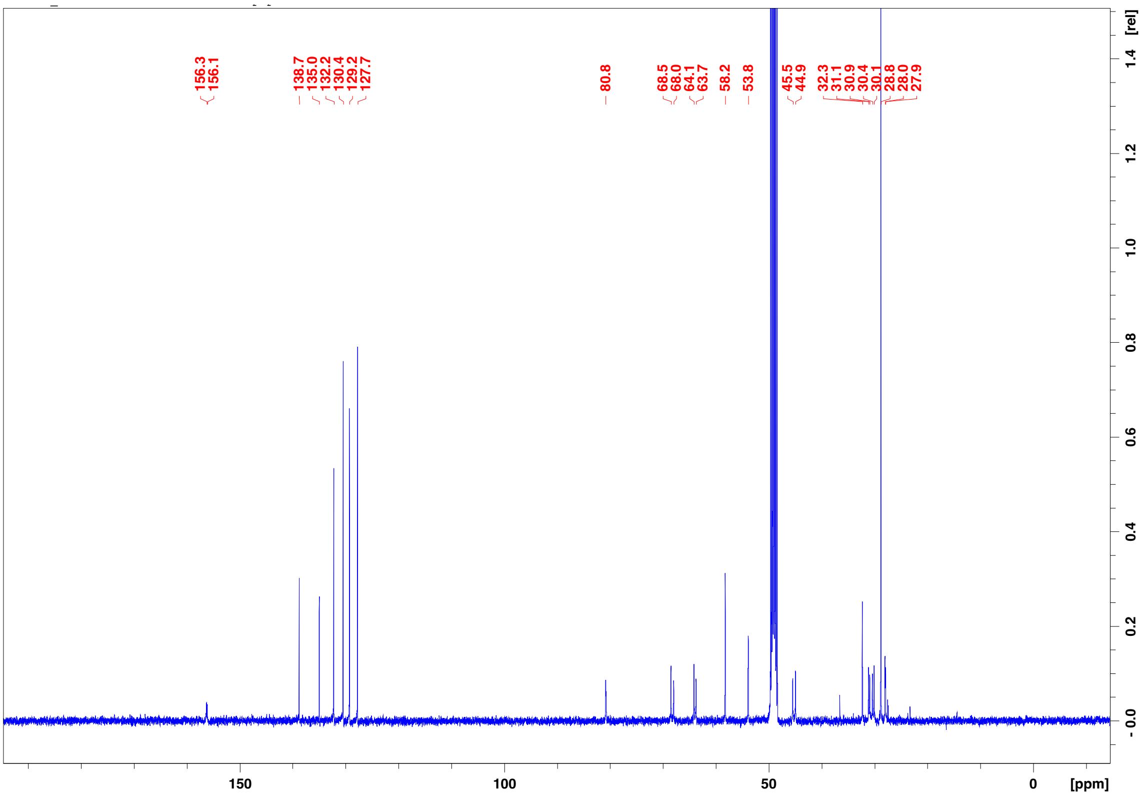

(3a*R*,8a*R*)-4-(4-chlorobenzyl)decahydropyrrolo[3,2-b]azepine ((*R,R*)-**58**)

^{1}H-NMR

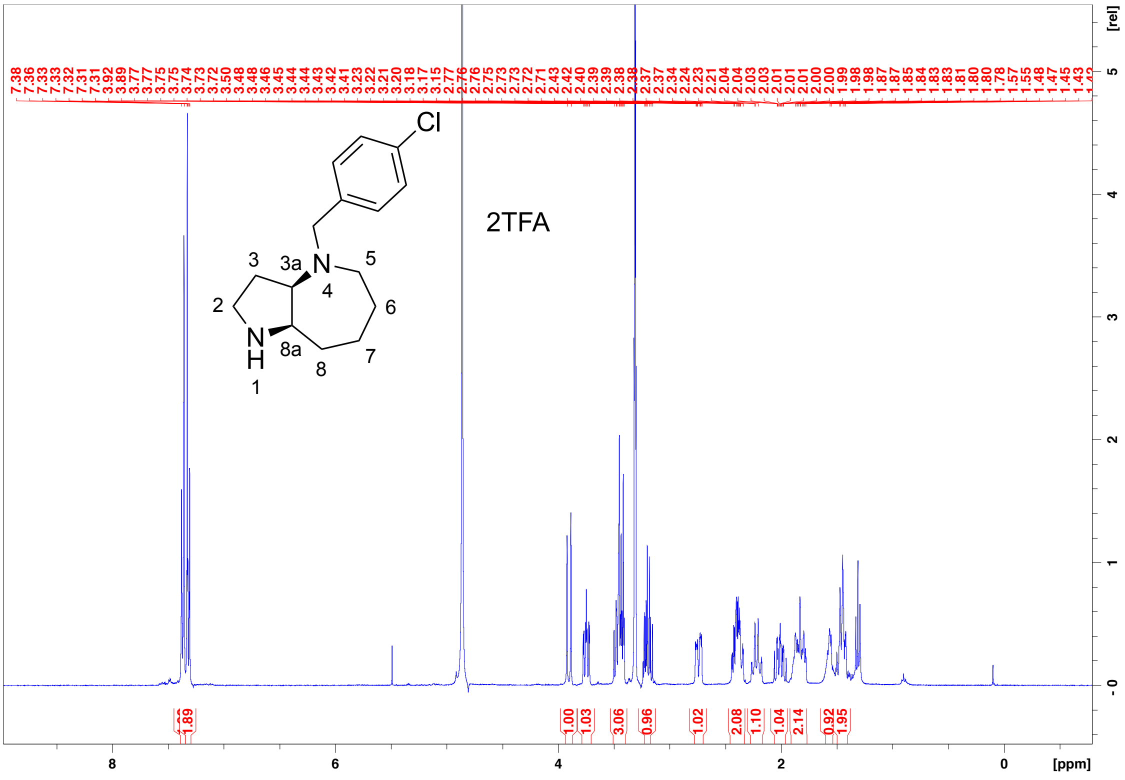

^{13}C-NMR

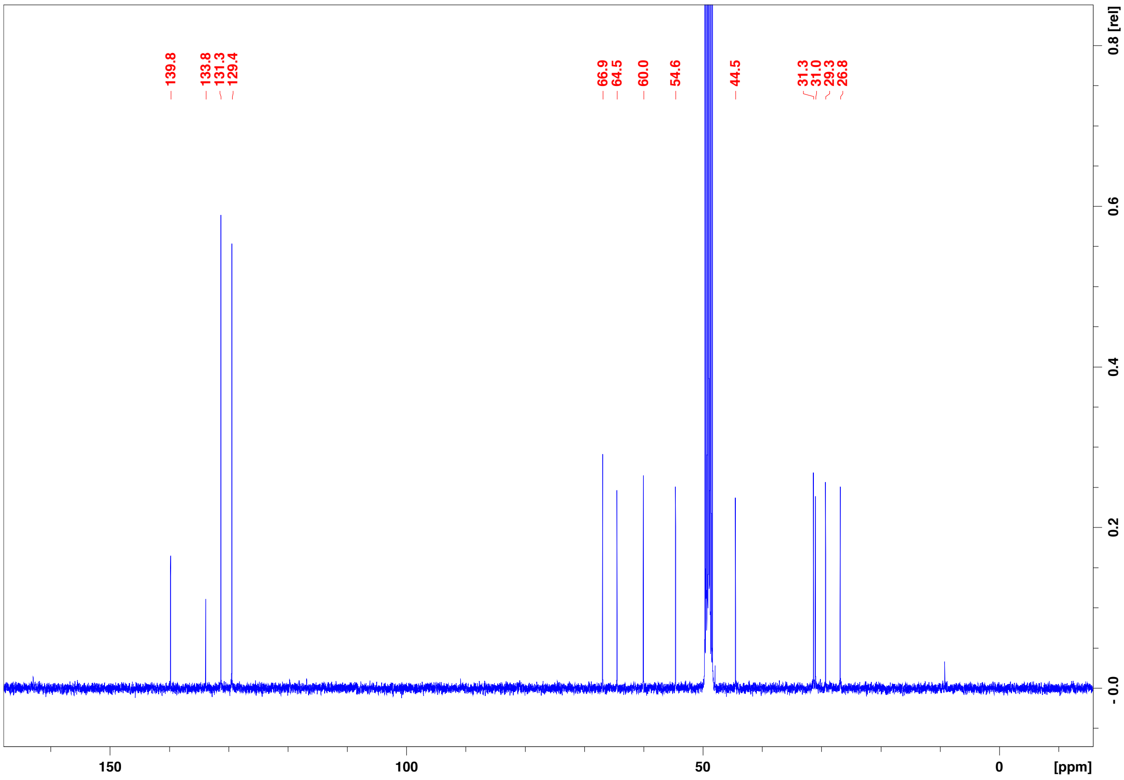

(3a*R*,8a*R*)-4-(3-chlorobenzyl)decahydropyrrolo[3,2-b]azepine ((*R,R*)-**59**)

^{1}H-NMR

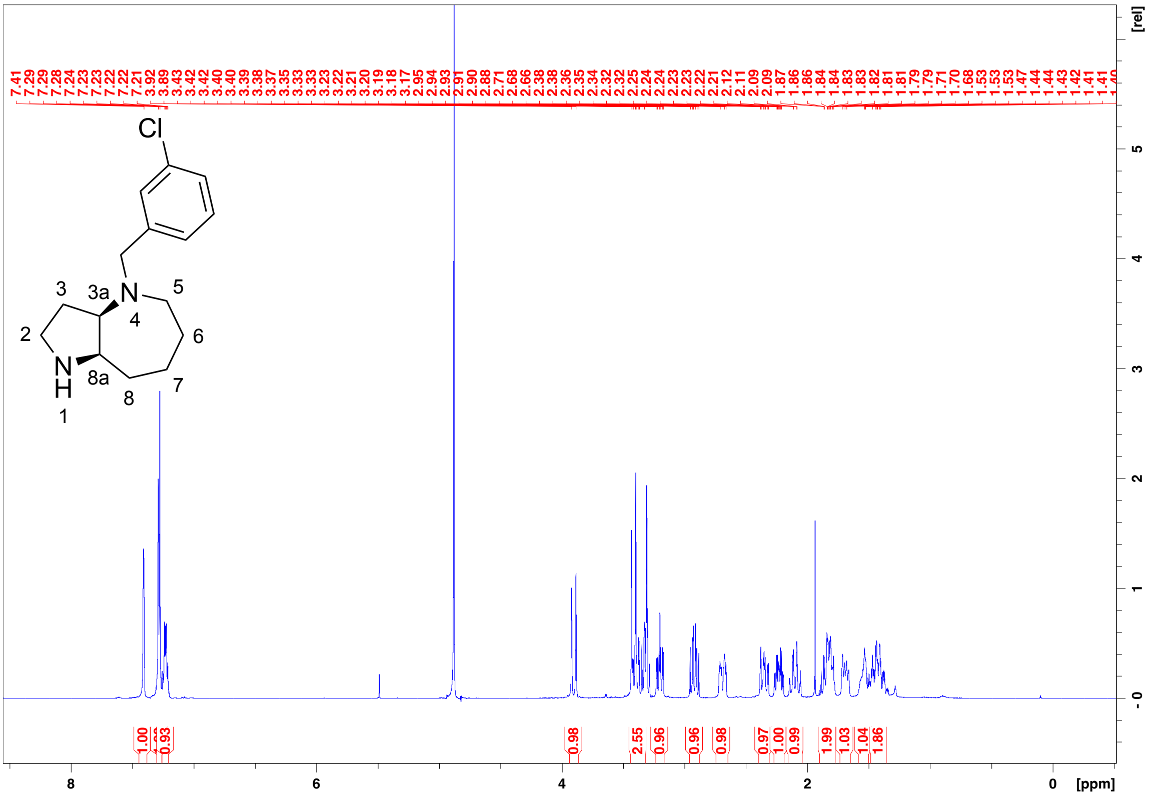

^{13}C-NMR

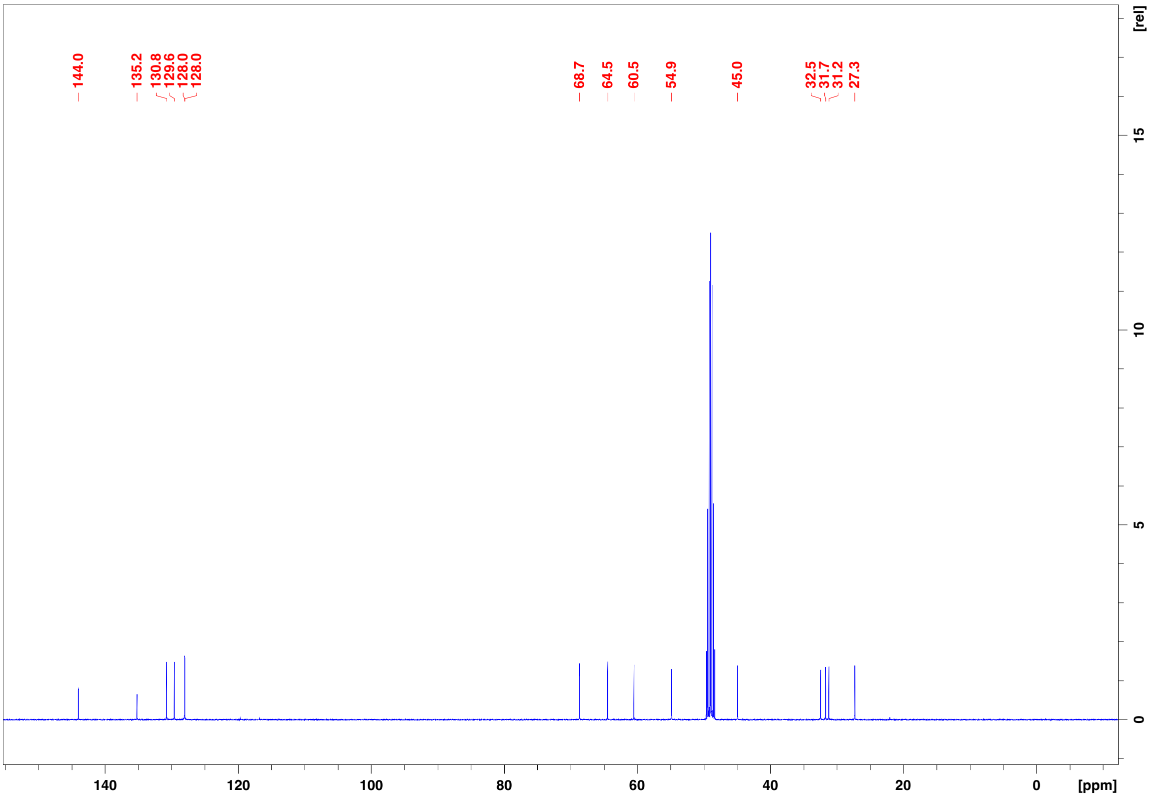

(3a*R*,8a*R*)-4-(2-chlorobenzyl)decahydropyrrolo[3,2-b]azepine ((*R*,*R*)-**60**)

^{1}H-NMR

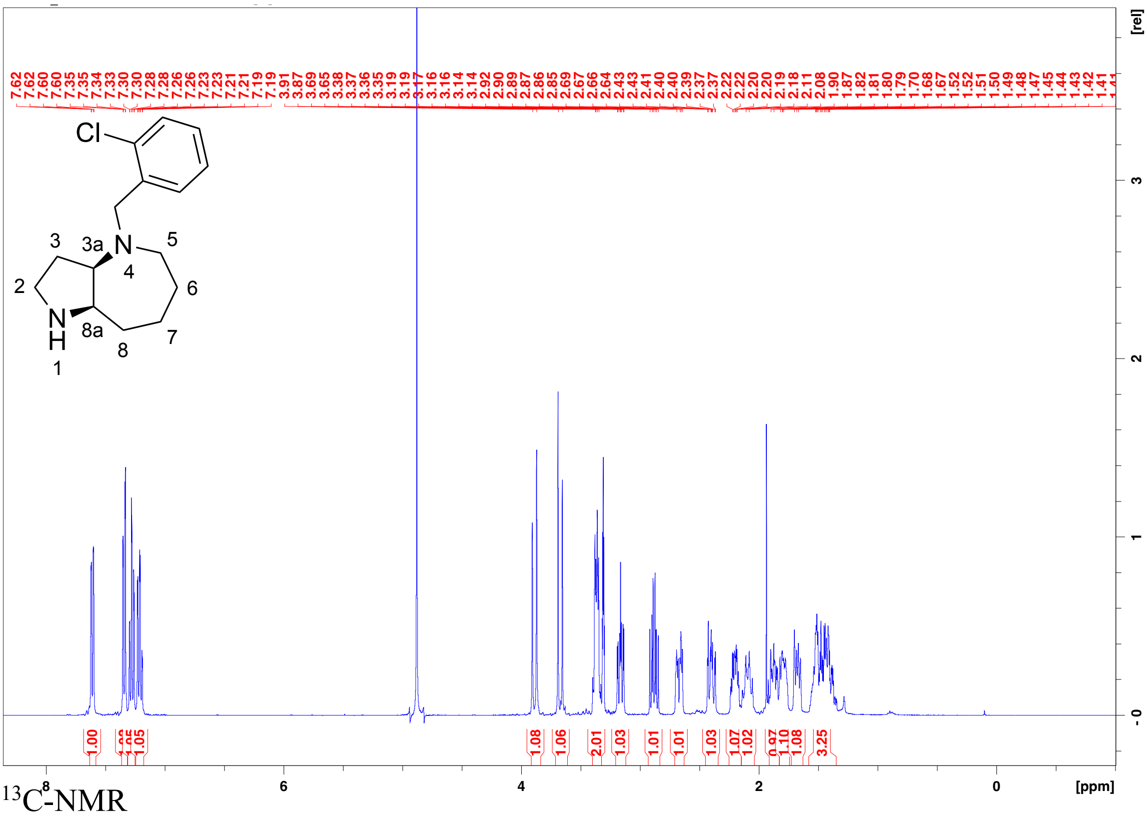

^{13}C-NMR

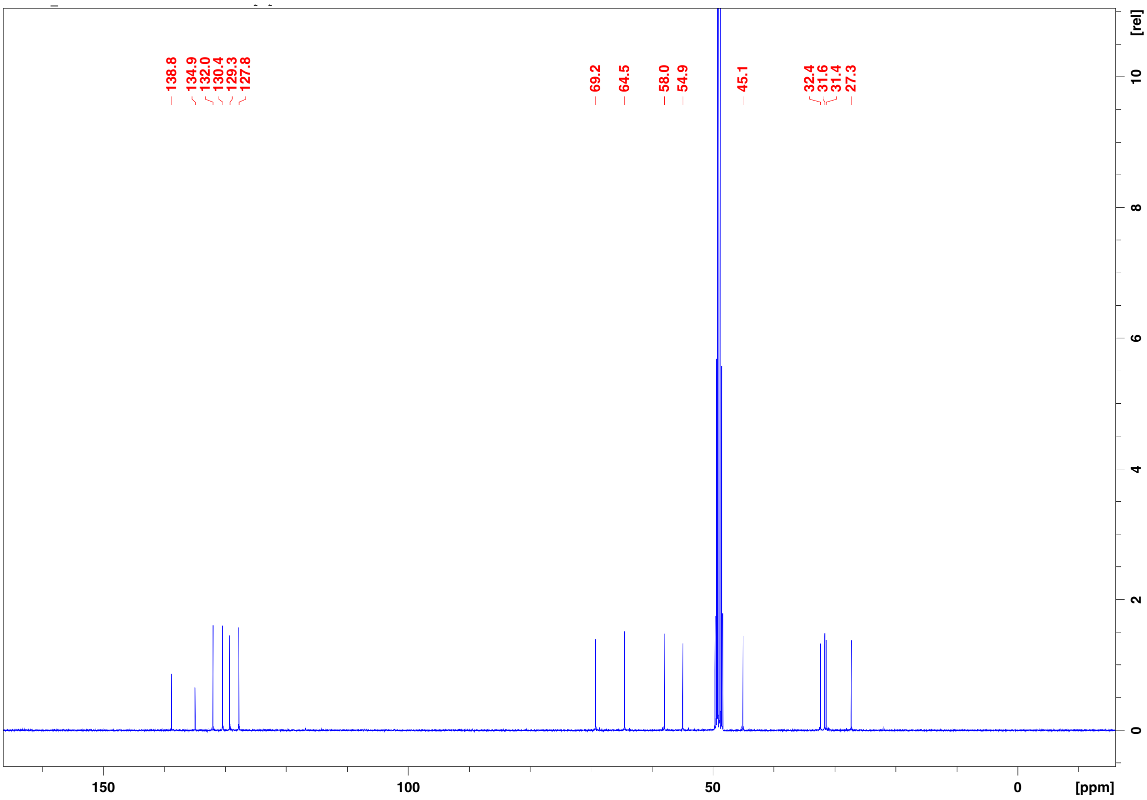

(3a*R*,8a*R*)-4-(3-bromobenzyl)decahydropyrrolo[3,2-b]azepine ((*R,R*)-**61**)

^{1}H-NMR

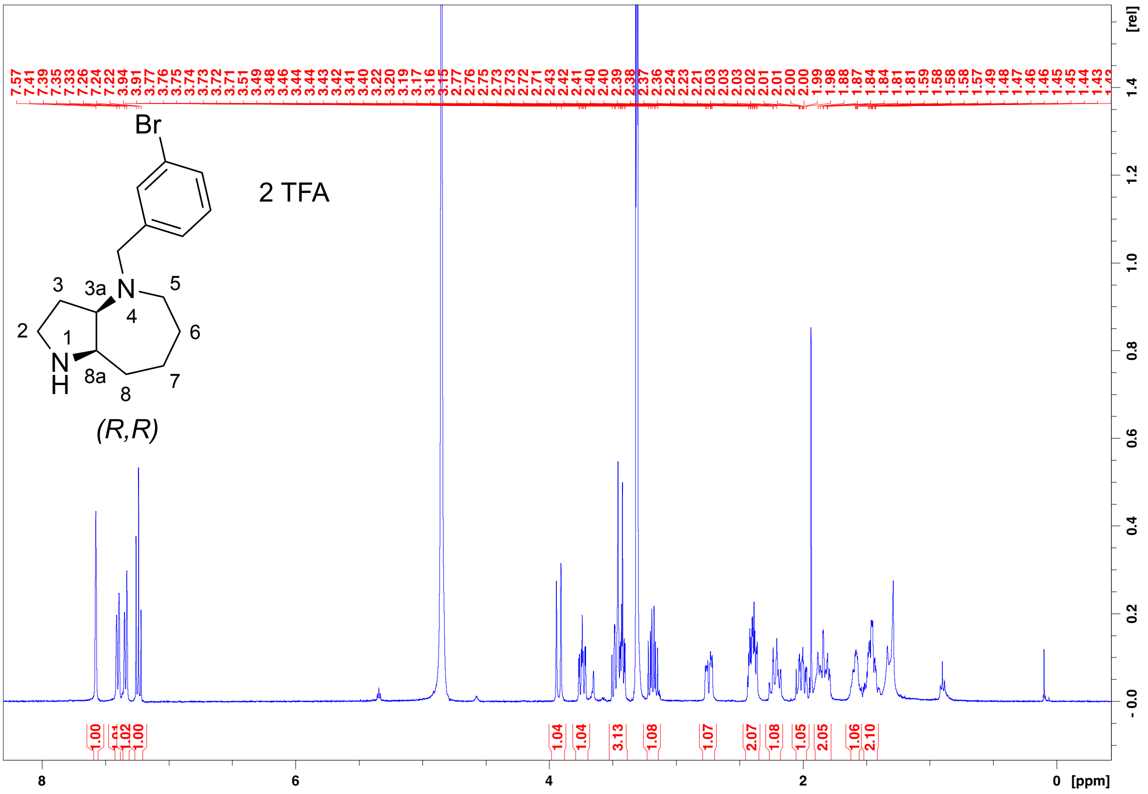

^{13}C-NMR

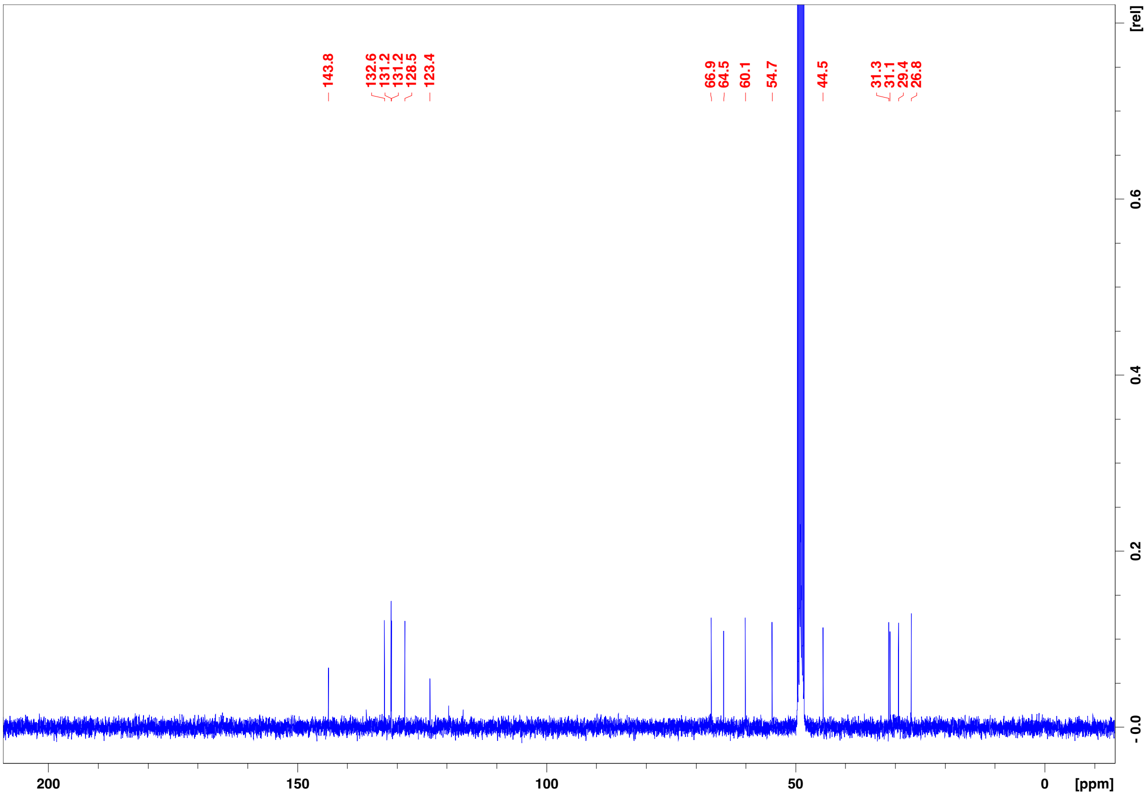

(3a*R*,8a*R*)-4-(2,3-dichlorobenzyl)decahydropyrrolo[3,2-*b*]azepine ((*R,R*)-**62**)

^{1}H-NMR

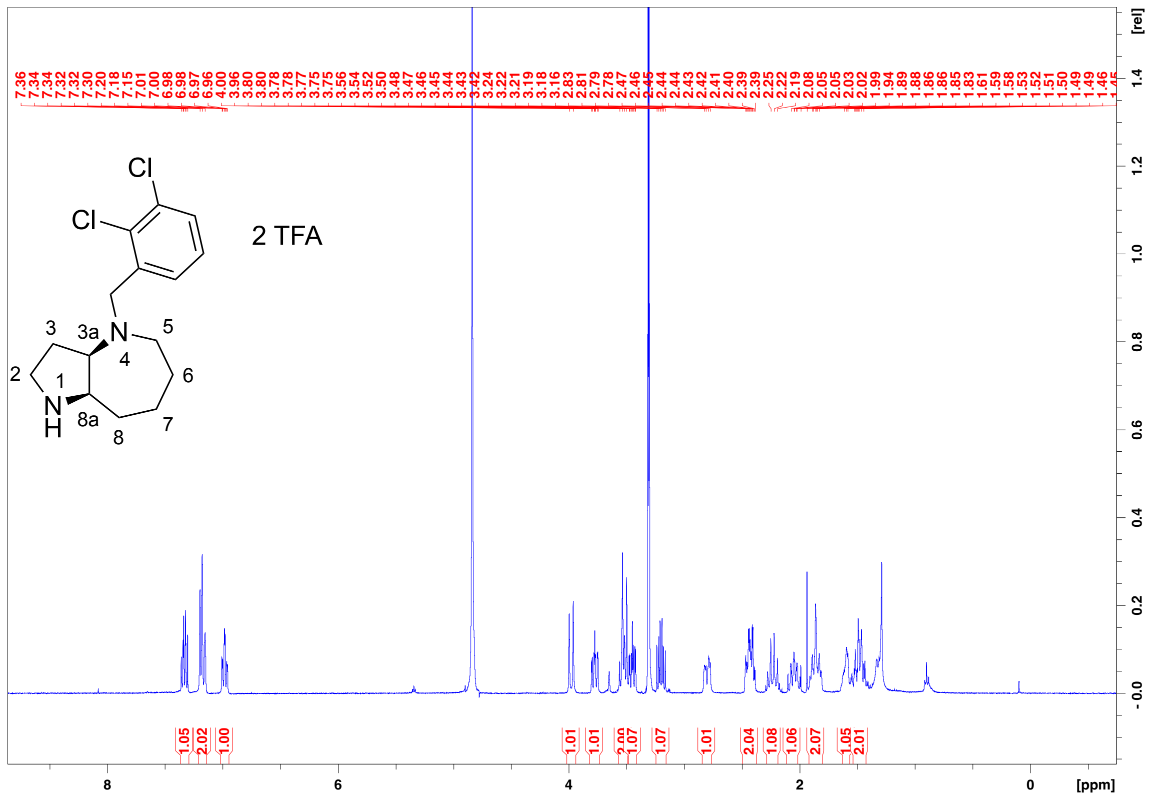

^{13}C-NMR

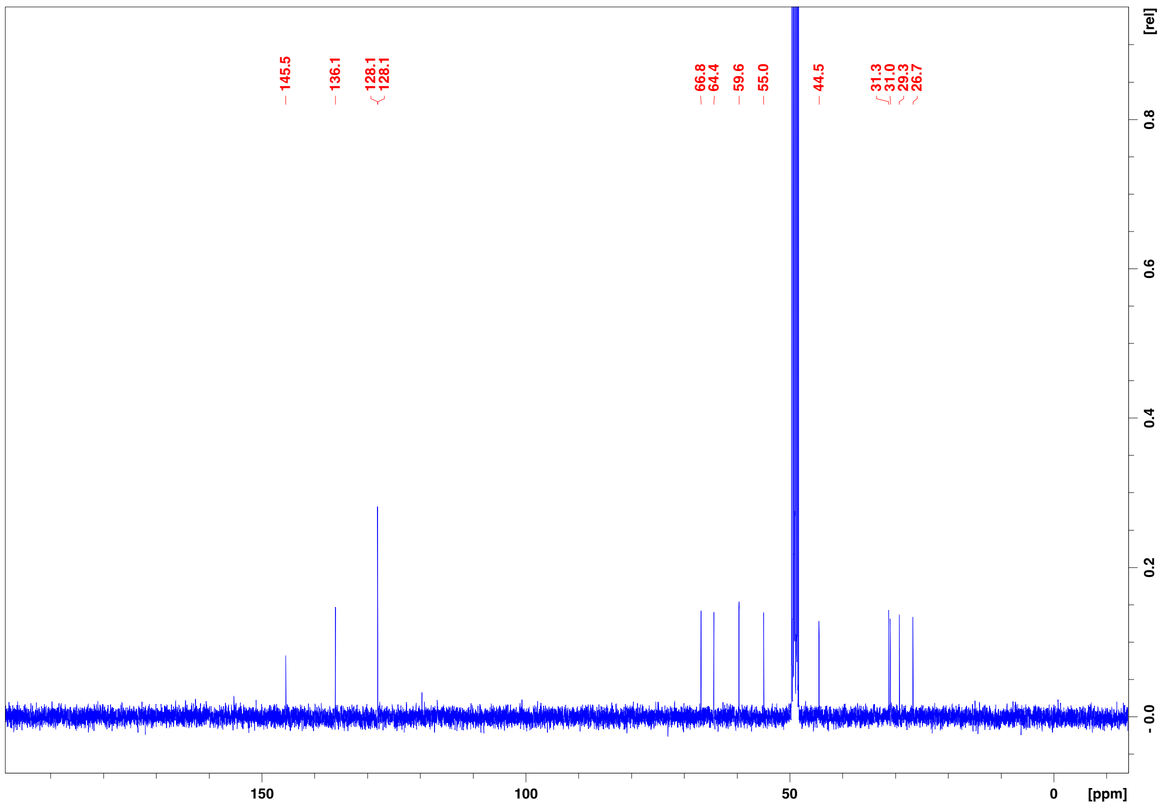

(3a*R*,8a*R*)-4-(3,5-dichlorobenzyl)decahydropyrrolo[3,2-*b*]azepine ((*R*,*R*)-**63**)

^{1}H-NMR

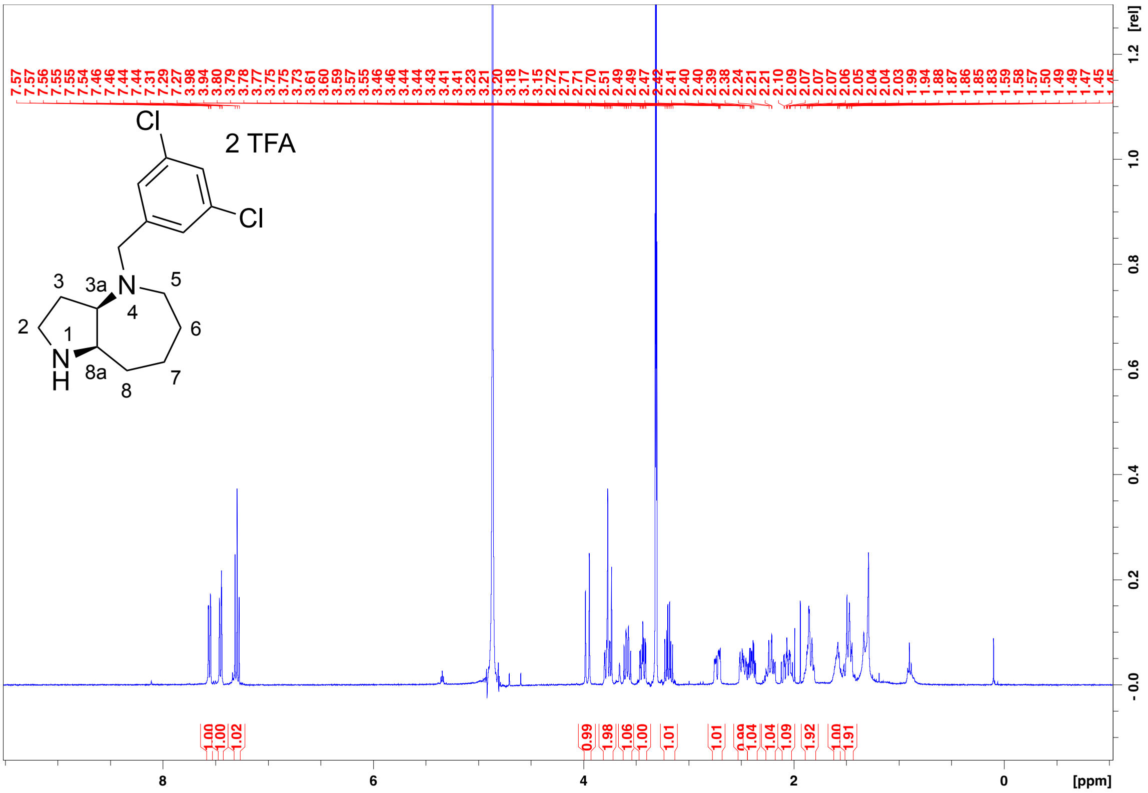

^{13}C-NMR

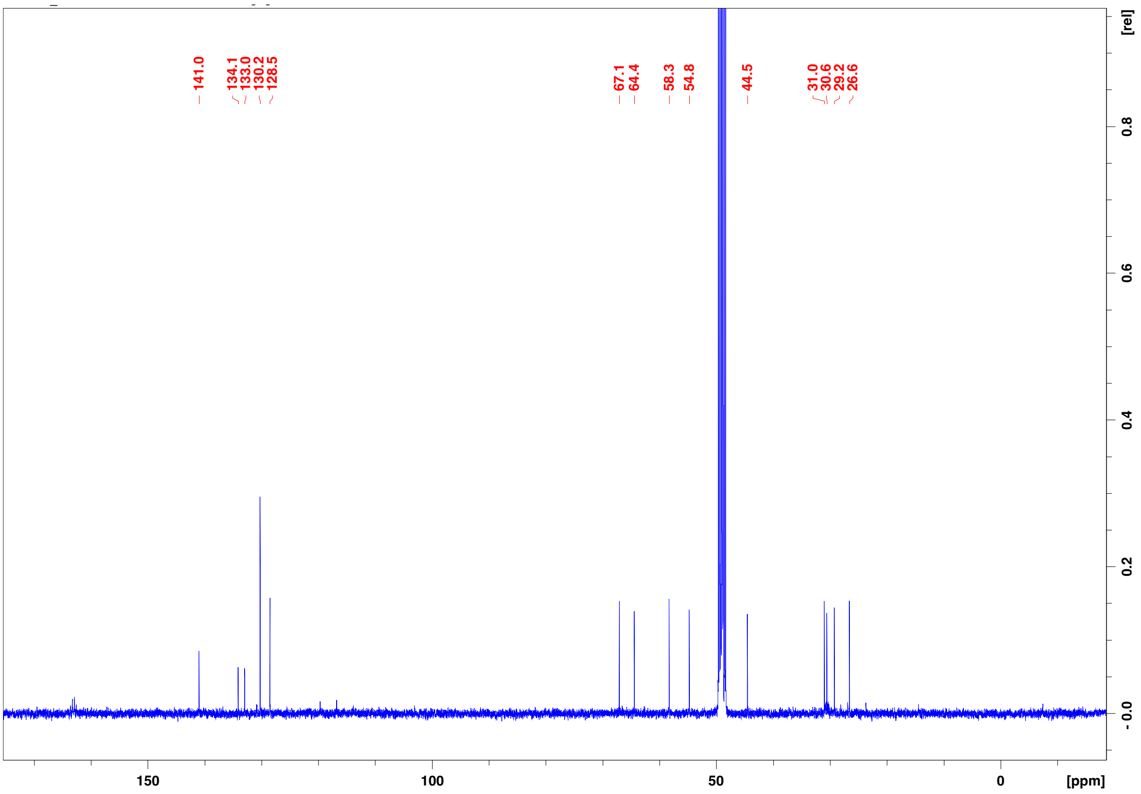

3 Chiral HPLC

tert-butyl-4-benzyloctahydropyrrolo[3,2-b]azepine-1(2H)-carboxylate ((±)-**35**)

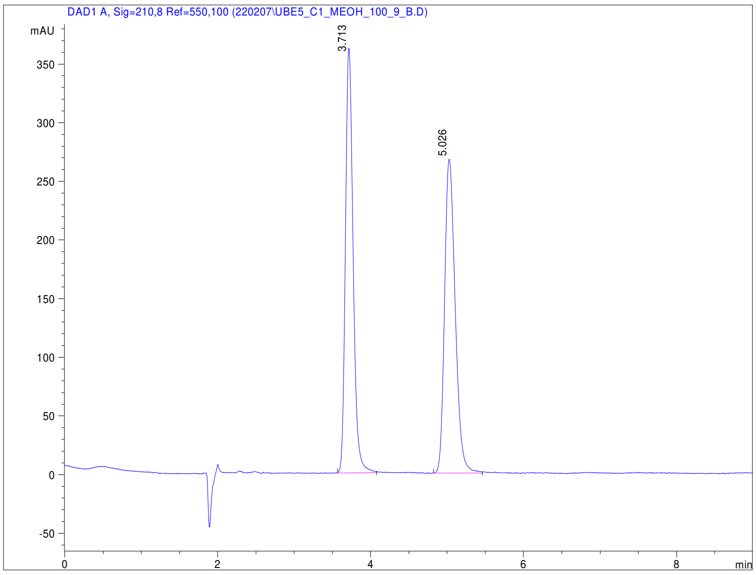

#	Meas. R	Height	Width	Area	Area %	Symmetr
1	3.713	362.446	0.117	2.539e3	49.498	0.774
2	5.026	267.818	0.161	2.591e3	50.502	0.734

tert-butyl-(3a*R*,8a*R*)-4-benzyloctahydropyrrolo[3,2-b]azepine-1(2H)-carboxylate (*(R,R)*-**35**)

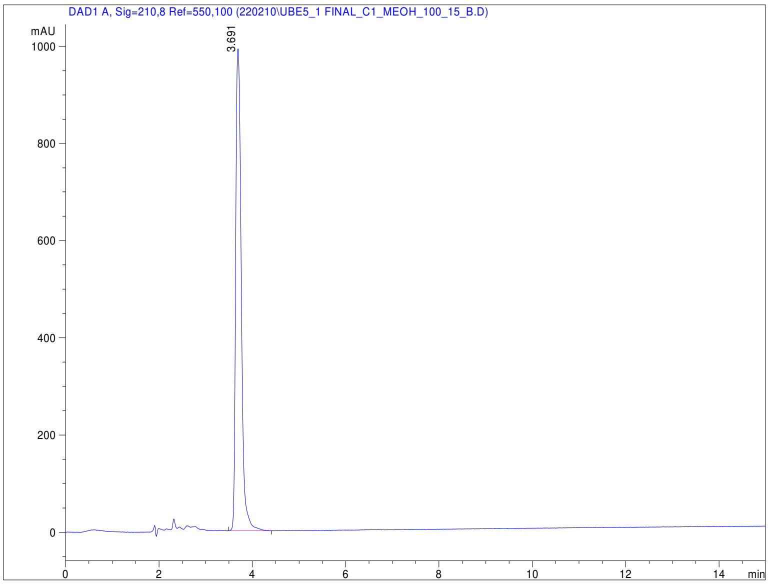

#	Meas. R	Height	Width	Area	Area %	Symmetr
1	3.691	991.943	0.139	8.245e3	100.000	0.706

<u>*tert*-butyl-(3a*S*,8a*S*)-4-benzyloctahydropyrrolo[3,2-b]azepine-1(2H)-carboxylate ((*S*,*S*)-**35**)</u>

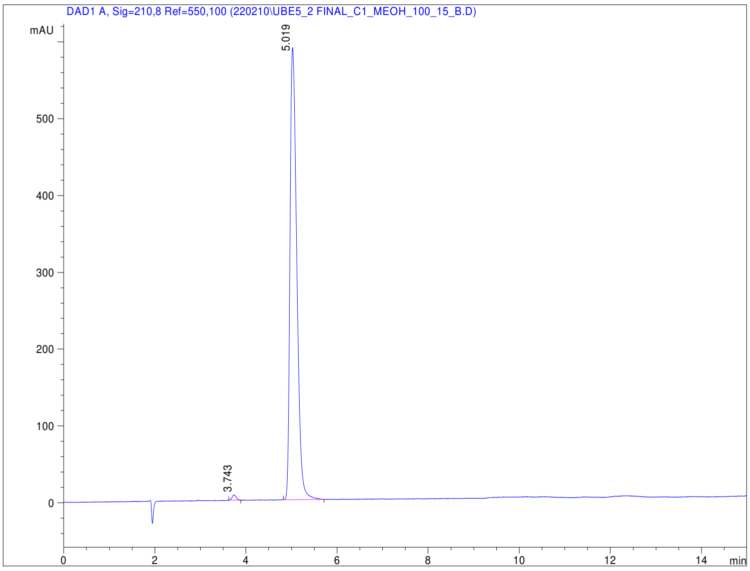

#	Meas. R	Height	Width	Area	Area %	Symmetr
1	3.743	7.158	0.105	45.293	0.739	1.208
2	5.019	588.790	0.172	6.086e3	99.261	0.591

3.1 Chemical Purity

<u>*tert*-butyl-4-benzyloctahydropyrrolo[3,2-b]azepine-1(2H)-carboxylate ((±)-**35**)</u>

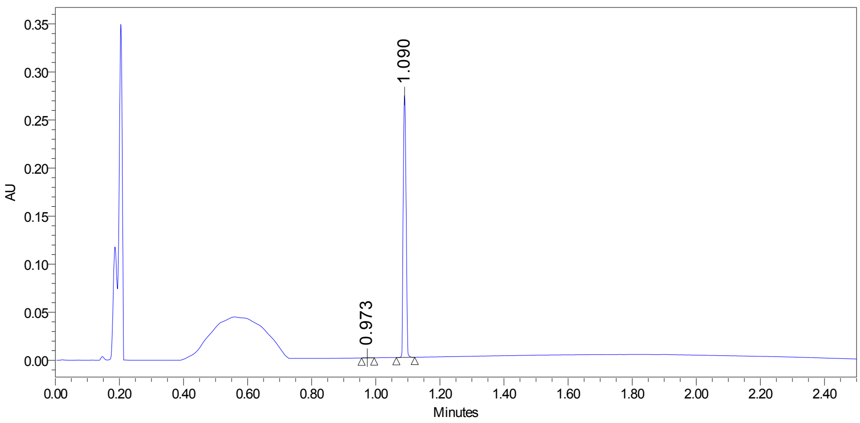

PDA Peak Results

	Retention Time (min)	Area (µV*sec)	% Area	Width (sec)
1	0.97	242	0.14	2.350
2	1.09	170452	99.86	3.450

tert-butyl-(3a*R*,8a*R*)-4-benzyloctahydropyrrolo[3,2-b]azepine-1(2H)-carboxylate (*(R,R)*-**35**)

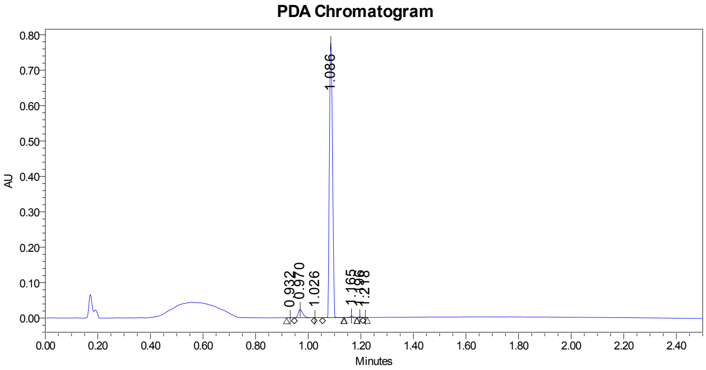

PDA Peak Results

	Retention Time (min)	Area (µV*sec)	% Area	Width (sec)
1	0.93	252	0.04	1.800
2	0.97	28070	4.20	4.500
3	1.03	166	0.02	1.900
4	1.09	635832	95.10	4.850
5	1.16	3070	0.46	2.850
6	1.20	1216	0.18	1.350
7	1.22	16	0.00	1.000

tert-butyl-(3a*S*,8a*S*)-4-benzyloctahydropyrrolo[3,2-b]azepine-1(2H)-carboxylate (*(S,S)*-**35**)

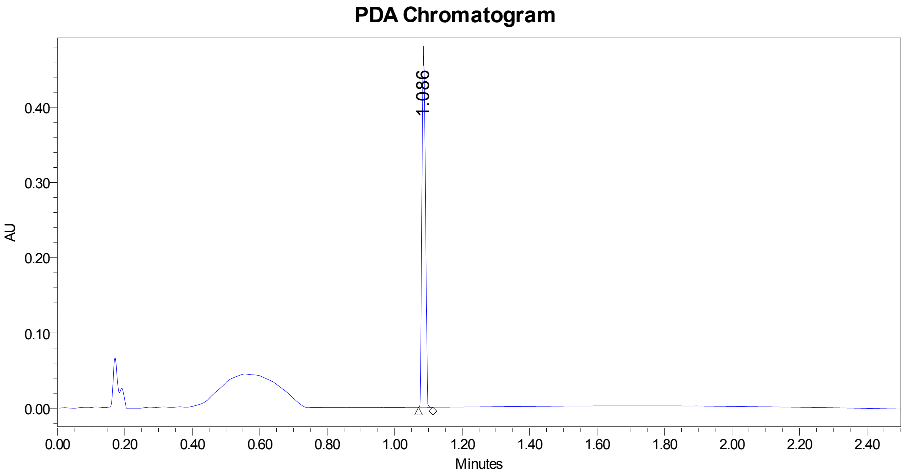

PDA Peak Results

	Retention Time (min)	Area (µV*sec)	% Area	Width (sec)
1	1.09	347521	100.00	2.550

4 Analytical purity for tested compounds

4.1 Table S1

Table S1. Analytical HPLC purity for tested compounds.

Compound	Purification	HPLC Purity (%)	Enantiomeric ratio
**17a**	Silica column chromatography	>99	-
**17b**	Silica column chromatography	>99	-
**27a**	Silica column chromatography	>99	-
**27b**	Silica column chromatography	>99	-
**1a**	Silica column chromatography	>99	.
(*R,R*)-**35**	RP-HPLC/ chiral column	>95	100/0
(S,*S*)-**35**	RP-HPLC/ chiral column	100	98.5/1.5
(*R,R*)-**1a**	Silica column chromatography	>99	-
(*S,S*)-**1a**	Silica column chromatography	>99	-
**1b**	Silica column chromatography	>99	-
**40a**	Silica column chromatography	>99	-
**40b**	Silica column chromatography	>99	-
**44b**	Silica column chromatography	>99	-
**4-chloro** ((*R,R*)-**58**)	Silica column chromatography	>99	-
**3-chloro** ((*R,R*)-**59**)	Silica column chromatography	>99	-
**2-chloro** ((*R,R*)-**60**)	Silica column chromatography	>99	-
**3-bromo** ((*R,R*)-**61**)	Silica column chromatography	>99	-
**2,3-dichloro** ((*R,R*)-**63**)	Silica column chromatography	>99	-
**3,5-dichloro** ((*R,R*)-**64**)	Silica column chromatography	>99	-

Analytical RP-HPLC was performed with an *Ultimate 3000 Rapid Separation* LC–MS System (*DAD-3000RS* diode array detector) using an *Acclaim RSLC 120 C18* column (2.2 µm, 120 Å, 3×50 mm, flow 1.2 mL/min) from *Dionex*. Data recording and processing was done with Dionex Chromeleon Management System Version 6.80 (analytical RP-HPLC). All RP-HPLC were using HPLC-grade acetonitrile and *Milli-Q* deionized water. The elution solutions were *A*) *MilliQ* deionized water containing 0.05 % TFA; *D*) *MilliQ* deionized water/acetonitrile (10 : 90, *v/v*) containing 0.05 % TFA.

5 X-ray crystal deposition

CCDC 2312956, 2303861, 2312959, 2312957, 2312961, 2312960 and 2312958 contain the supplementary crystallographic data for this paper. These data can be obtained free of charge from the Cambridge Crystallographic Data Centre via www.ccdc.cam.ac.uk/structures.

5.1 Figure S2

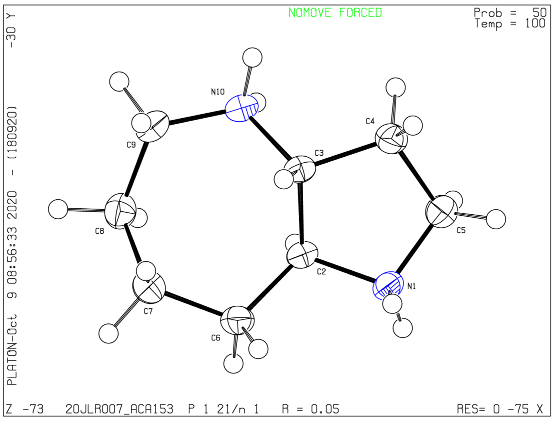

2b

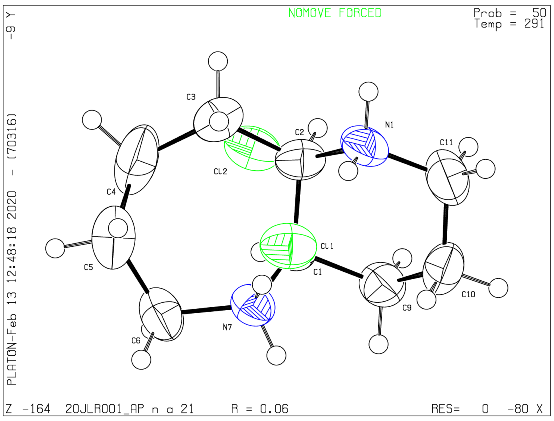

3a

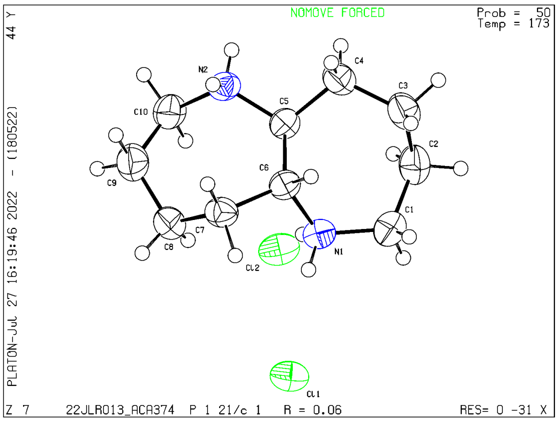

4b

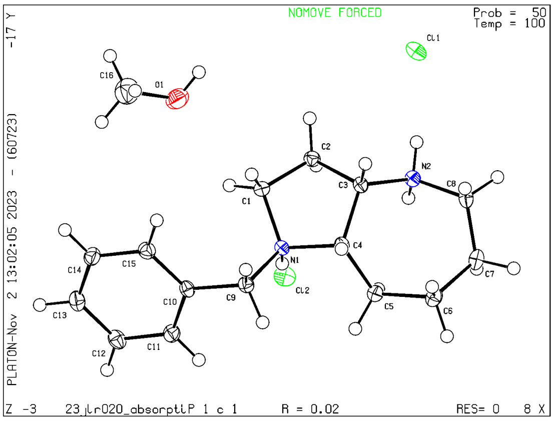

17a

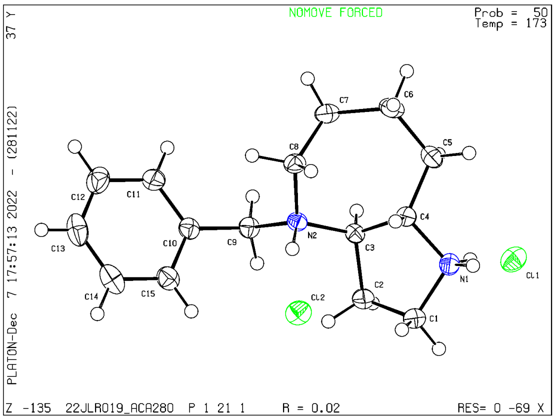

1b

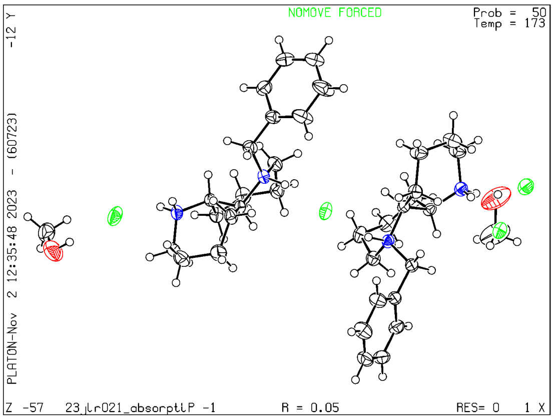

44b

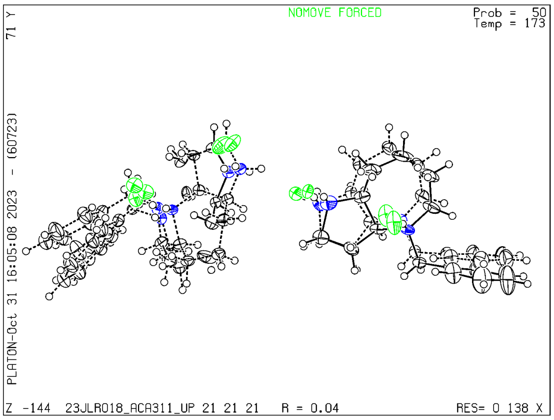

(*R,R*)-**1a**

Figure S2. X-ray crystal structures of **2b** (CCDC 2312956), **3a** (CCDC 2303861), **4b** (CCDC 2312959), **17a** (CCDC 2312957), **1b** (CCDC 2312961), **44b** (CCDC 2312960) and (*R,R*) -**1a** (CCDC 2312958) confirming the ring fusion stereochemistries. The structures are shown as ORTEP with ellipsoids drawn at the 50% probability level. Hydrogen atoms (white spheres, arbitrary radius) were located in the difference Fourier map and refined freely. CCDC codes contains the supplementary crystallographic data for this paper.

6 PPB2 Target prediction for fused azepanes

6.1 Table S2

Table S2. PPB2 Target prediction for fused azepanes.

**Cpd. No.**	**2a/b**	**3a/b**	**4b**	**17a/17b**	**27a/27b**	**1a/1b**	**40a/40b**	**44b**	
**Rings**	5,7	6,7	7,7	5,7	6,7	5,7	6,7	7,7	
***N*-substituent**				5-Bn	6-Bn	7-Bn	7-Bn	7-Bn	
**Target score**[a)]									**Average**
Dopamine transporter[b)]	38[a]	38[a]	38[a]	38[b]	39[b]	36[b]	37[b]	37[b]	38
Norepinephrine transporter[c)]	38	37	37	24	30	16	21	25	29
Serotonin Transporter[d)]	30[d]	35[d]	36[d]	30[e]	30[d]	31[e]	24[d]	24[d]	30
Histamine H3 receptor[e)]	27	30	30	21	21	20	31	34	27
Sigma opioid receptor[f)]	24[h]	16[h]	17[h]	38[g]	29[g]	39[g]	40[h]	37[h]	30

a) For each cpd, the two PPB2 methods were used and each target in the top-20 list was assigned a target score = 20-rank. The sum of the two ranks is given. Target ChEMBL IDs : b) CHEMBL238 for free diamines and CHEMBL338 for N-benzylated amines; , c) CHEMBL222, d) CHEMBL228, and CHEMBL313 for **17a/b** and **1a/b**; e) CHEMBL264; f) CHEMBL360, and CHEMBL287 for **17a/b**, **27a/b** and **1a/b**.

6.2 Figure S3

A)

Targets predicted using NN(ECfp4) + NB(ECfp4). Save Table

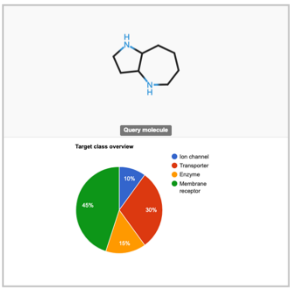

Rank	ChEMBL ID	Common name	Nearest neighbours
1	CHEMBL338	Dopamine transporter	Show NN
2	CHEMBL238	Dopamine transporter	Show NN
3	CHEMBL222	Norepinephrine transporter	Show NN
4	CHEMBL228	Serotonin transporter	Show NN
5	CHEMBL313	Serotonin transporter	Show NN
6	CHEMBL3759	Histamine H4 receptor	Show NN
7	CHEMBL3105	Poly [ADP-ribose] polymerase-1	Show NN
8	CHEMBL240	HERG	Show NN
9	CHEMBL284	Dipeptidyl peptidase IV	Show NN
10	CHEMBL264	Histamine H3 receptor	Show NN
11	CHEMBL3602	Sigma opioid receptor	Show NN
12	CHEMBL4980	Neuronal acetylcholine receptor protein alpha-7 subunit	Show NN
13	CHEMBL1804	Somatostatin receptor 2	Show NN
14	CHEMBL216	Muscarinic acetylcholine receptor M1	Show NN
15	CHEMBL4616	Ghrelin receptor	Show NN
16	CHEMBL2014	Nociceptin receptor	Show NN
17	CHEMBL304	Norepinephrine transporter	Show NN
18	CHEMBL4618	Leukotriene A4 hydrolase	Show NN
19	CHEMBL3066	Gonadotropin-releasing hormone receptor	Show NN
20	CHEMBL339	Dopamine D2 receptor	Show NN

B)

Targets predicted using NN(Xfp) + NB(ECfp4). Save Table

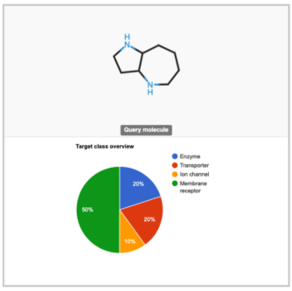

Rank	ChEMBL ID	Common name	Nearest neighbours
1	CHEMBL222	Norepinephrine transporter	Show NN
2	CHEMBL238	Dopamine transporter	Show NN
3	CHEMBL1821	Muscarinic acetylcholine receptor M4	Show NN
4	CHEMBL216	Muscarinic acetylcholine receptor M1	Show NN
5	CHEMBL264	Histamine H3 receptor	Show NN
6	CHEMBL2492	Neuronal acetylcholine receptor protein alpha-7 subunit	Show NN
7	CHEMBL3602	Sigma opioid receptor	Show NN
8	CHEMBL228	Serotonin transporter	Show NN
9	CHEMBL245	Muscarinic acetylcholine receptor M3	Show NN
10	CHEMBL211	Muscarinic acetylcholine receptor M2	Show NN
11	CHEMBL338	Dopamine transporter	Show NN
12	CHEMBL4980	Neuronal acetylcholine receptor protein alpha-7 subunit	Show NN
13	CHEMBL289	Cytochrome P450 2D6	Show NN
14	CHEMBL309	Muscarinic acetylcholine receptor M2	Show NN
15	CHEMBL276	Muscarinic acetylcholine receptor M1	Show NN
16	CHEMBL2035	Muscarinic acetylcholine receptor M5	Show NN
17	CHEMBL4617	Phenylethanolamine N-methyltransferase	Show NN
18	CHEMBL287	Sigma opioid receptor	Show NN
19	CHEMBL284	Dipeptidyl peptidase IV	Show NN
20	CHEMBL1970	Alcohol dehydrogenase alpha chain	Show NN

Figure S3. PPB2 target prediction for **2a/2b**. **A)** Morgan fingerprint ECFP4. **B)** pharmacophore fingerprint XFP and ECFP4.[3]

6.3 Figure S4

A)

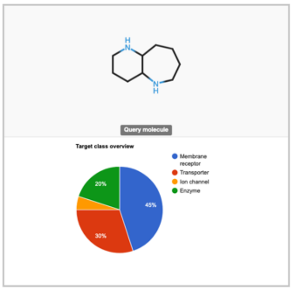

Targets predicted using NN(ECfp4) + NB(ECfp4). Save Table

Rank	ChEMBL ID	Common name	Nearest neighbours
1	CHEMBL338	Dopamine transporter	Show NN
2	CHEMBL238	Dopamine transporter	Show NN
3	CHEMBL228	Serotonin transporter	Show NN
4	CHEMBL222	Norepinephrine transporter	Show NN
5	CHEMBL313	Serotonin transporter	Show NN
6	CHEMBL240	HERG	Show NN
7	CHEMBL284	Dipeptidyl peptidase IV	Show NN
8	CHEMBL3759	Histamine H4 receptor	Show NN
9	CHEMBL264	Histamine H3 receptor	Show NN
10	CHEMBL4616	Ghrelin receptor	Show NN
11	CHEMBL3105	Poly [ADP-ribose] polymerase-1	Show NN
12	CHEMBL3066	Gonadotropin-releasing hormone receptor	Show NN
13	CHEMBL225	Serotonin 2c (5-HT2c) receptor	Show NN
14	CHEMBL4618	Leukotriene A4 hydrolase	Show NN
15	CHEMBL224	Serotonin 2a (5-HT2a) receptor	Show NN
16	CHEMBL3602	Sigma opioid receptor	Show NN
17	CHEMBL249	Neurokinin 1 receptor	Show NN
18	CHEMBL1804	Somatostatin receptor 2	Show NN
19	CHEMBL304	Norepinephrine transporter	Show NN
20	CHEMBL1991	Inhibitor of nuclear factor kappa B kinase beta subunit	Show NN

B)

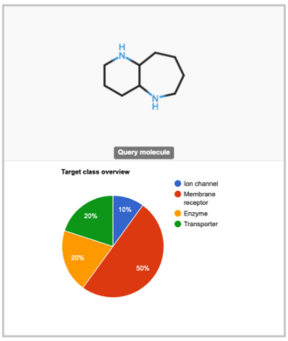

Targets predicted using NN(Xfp) + NB(ECfp4). Save Table

Rank	ChEMBL ID	Common name	Nearest neighbours
1	CHEMBL222	Norepinephrine transporter	Show NN
2	CHEMBL238	Dopamine transporter	Show NN
3	CHEMBL264	Histamine H3 receptor	Show NN
4	CHEMBL228	Serotonin transporter	Show NN
5	CHEMBL1821	Muscarinic acetylcholine receptor M4	Show NN
6	CHEMBL216	Muscarinic acetylcholine receptor M1	Show NN
7	CHEMBL338	Dopamine transporter	Show NN
8	CHEMBL211	Muscarinic acetylcholine receptor M2	Show NN
9	CHEMBL4980	Neuronal acetylcholine receptor protein alpha-7 subunit	Show NN
10	CHEMBL3602	Sigma opioid receptor	Show NN
11	CHEMBL2492	Neuronal acetylcholine receptor protein alpha-7 subunit	Show NN
12	CHEMBL245	Muscarinic acetylcholine receptor M3	Show NN
13	CHEMBL284	Dipeptidyl peptidase IV	Show NN
14	CHEMBL276	Muscarinic acetylcholine receptor M1	Show NN
15	CHEMBL309	Muscarinic acetylcholine receptor M2	Show NN
16	CHEMBL289	Cytochrome P450 2D6	Show NN
17	CHEMBL287	Sigma opioid receptor	Show NN
18	CHEMBL2035	Muscarinic acetylcholine receptor M5	Show NN
19	CHEMBL4617	Phenylethanolamine N-methyltransferase	Show NN
20	CHEMBL4481	Nitric oxide synthase, inducible	Show NN

Figure S4. PPB2 target prediction for **3a**/**3b**. **A)** Morgan fingerprint ECFP4. **B)** pharmacophore fingerprint XFP and ECFP4.[3]

6.4 Figure S5

A)

Targets predicted using NN(ECfp4) + NB(ECfp4). Save Table

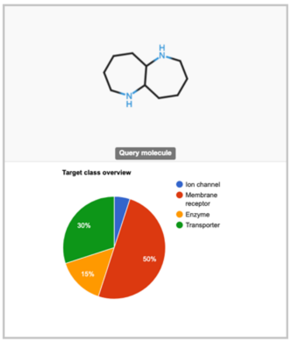

Rank	ChEMBL ID	Common name	Nearest neighbours
1	CHEMBL338	Dopamine transporter	Show NN
2	CHEMBL238	Dopamine transporter	Show NN
3	CHEMBL228	Serotonin transporter	Show NN
4	CHEMBL222	Norepinephrine transporter	Show NN
5	CHEMBL313	Serotonin transporter	Show NN
6	CHEMBL240	HERG	Show NN
7	CHEMBL264	Histamine H3 receptor	Show NN
8	CHEMBL3066	Gonadotropin-releasing hormone receptor	Show NN
9	CHEMBL1804	Somatostatin receptor 2	Show NN
10	CHEMBL3759	Histamine H4 receptor	Show NN
11	CHEMBL304	Norepinephrine transporter	Show NN
12	CHEMBL284	Dipeptidyl peptidase IV	Show NN
13	CHEMBL1855	Gonadotropin-releasing hormone receptor	Show NN
14	CHEMBL4618	Leukotriene A4 hydrolase	Show NN
15	CHEMBL3602	Sigma opioid receptor	Show NN
16	CHEMBL3105	Poly [ADP-ribose] polymerase-1	Show NN
17	CHEMBL224	Serotonin 2a (5-HT2a) receptor	Show NN
18	CHEMBL4616	Ghrelin receptor	Show NN
19	CHEMBL225	Serotonin 2c (5-HT2c) receptor	Show NN
20	CHEMBL2014	Nociceptin receptor	Show NN

B)

Targets predicted using NN(Xfp) + NB(ECfp4). Save Table

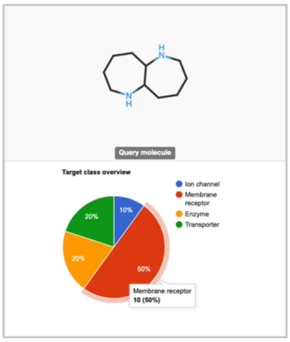

Rank	ChEMBL ID	Common name	Nearest neighbours
1	CHEMBL222	Norepinephrine transporter	Show NN
2	CHEMBL238	Dopamine transporter	Show NN
3	CHEMBL228	Serotonin transporter	Show NN
4	CHEMBL1821	Muscarinic acetylcholine receptor M4	Show NN
5	CHEMBL264	Histamine H3 receptor	Show NN
6	CHEMBL216	Muscarinic acetylcholine receptor M1	Show NN
7	CHEMBL338	Dopamine transporter	Show NN
8	CHEMBL211	Muscarinic acetylcholine receptor M2	Show NN
9	CHEMBL245	Muscarinic acetylcholine receptor M3	Show NN
10	CHEMBL3602	Sigma opioid receptor	Show NN
11	CHEMBL2492	Neuronal acetylcholine receptor protein alpha-7 subunit	Show NN
12	CHEMBL276	Muscarinic acetylcholine receptor M1	Show NN
13	CHEMBL289	Cytochrome P450 2D6	Show NN
14	CHEMBL287	Sigma opioid receptor	Show NN
15	CHEMBL309	Muscarinic acetylcholine receptor M2	Show NN
16	CHEMBL2035	Muscarinic acetylcholine receptor M5	Show NN
17	CHEMBL284	Dipeptidyl peptidase IV	Show NN
18	CHEMBL4617	Phenylethanolamine N-methyltransferase	Show NN
19	CHEMBL4980	Neuronal acetylcholine receptor protein alpha-7 subunit	Show NN
20	CHEMBL4481	Nitric oxide synthase, inducible	Show NN

Figure S5. PPB2 target prediction for **4b**. **A)** Morgan fingerprint ECFP4. **B)** pharmacophore fingerprint XFP and ECFP4.[3]

6.5 Figure S6

A)

Targets predicted using NN(ECfp4) + NB(ECfp4). Save Table

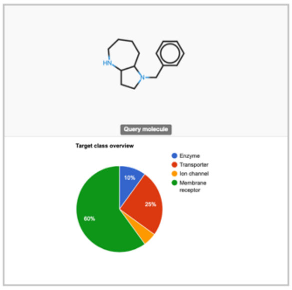

Rank	ChEMBL ID	Common name	Nearest neighbours
1	CHEMBL338	Dopamine transporter	Show NN
2	CHEMBL240	HERG	Show NN
3	CHEMBL287	Sigma opioid receptor	Show NN
4	CHEMBL233	Mu opioid receptor	Show NN
5	CHEMBL222	Norepinephrine transporter	Show NN
6	CHEMBL313	Serotonin transporter	Show NN
7	CHEMBL217	Dopamine D2 receptor	Show NN
8	CHEMBL3602	Sigma opioid receptor	Show NN
9	CHEMBL238	Dopamine transporter	Show NN
10	CHEMBL228	Serotonin transporter	Show NN
11	CHEMBL339	Dopamine D2 receptor	Show NN
12	CHEMBL249	Neurokinin 1 receptor	Show NN
13	CHEMBL264	Histamine H3 receptor	Show NN
14	CHEMBL284	Dipeptidyl peptidase IV	Show NN
15	CHEMBL253	Cannabinoid CB2 receptor	Show NN
16	CHEMBL4618	Leukotriene A4 hydrolase	Show NN
17	CHEMBL225	Serotonin 2c (5-HT2c) receptor	Show NN
18	CHEMBL237	Kappa opioid receptor	Show NN
19	CHEMBL245	Muscarinic acetylcholine receptor M3	Show NN
20	CHEMBL234	Dopamine D3 receptor	Show NN

B)

Targets predicted using NN(Xfp) + NB(ECfp4). Save Table

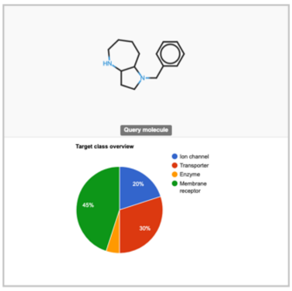

Rank	ChEMBL ID	Common name	Nearest neighbours
1	CHEMBL287	Sigma opioid receptor	Show NN
2	CHEMBL4618	Leukotriene A4 hydrolase	Show NN
3	CHEMBL338	Dopamine transporter	Show NN
4	CHEMBL228	Serotonin transporter	Show NN
5	CHEMBL214	Serotonin 1a (5-HT1a) receptor	Show NN
6	CHEMBL313	Serotonin transporter	Show NN
7	CHEMBL322	Serotonin 2a (5-HT2a) receptor	Show NN
8	CHEMBL264	Histamine H3 receptor	Show NN
9	CHEMBL240	HERG	Show NN
10	CHEMBL4767	Vesicular acetylcholine transporter	Show NN
11	CHEMBL3461	Neuronal acetylcholine receptor protein alpha-10 subunit	Show NN
12	CHEMBL3602	Sigma opioid receptor	Show NN
13	CHEMBL222	Norepinephrine transporter	Show NN
14	CHEMBL238	Dopamine transporter	Show NN
15	CHEMBL4980	Neuronal acetylcholine receptor protein alpha-7 subunit	Show NN
16	CHEMBL2492	Neuronal acetylcholine receptor protein alpha-7 subunit	Show NN
17	CHEMBL231	Histamine H1 receptor	Show NN
18	CHEMBL270	Mu opioid receptor	Show NN
19	CHEMBL233	Mu opioid receptor	Show NN
20	CHEMBL3155	Serotonin 7 (5-HT7) receptor	Show NN

Figure S6. PPB2 target prediction for **17a/17b**. **A)** Morgan fingerprint ECFP4. **B)** pharmacophore fingerprint XFP and ECFP4.[3]

6.6 Figure S7

A)

Targets predicted using NN(ECfp4) + NB(ECfp4). Save Table

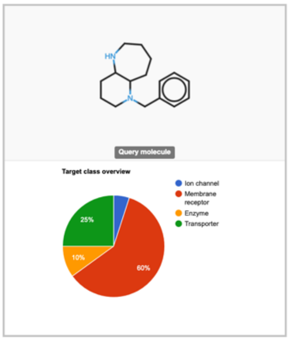

Rank	ChEMBL ID	Common name	Nearest neighbours
1	CHEMBL233	Mu opioid receptor	Show NN
2	CHEMBL338	Dopamine transporter	Show NN
3	CHEMBL284	Dipeptidyl peptidase IV	Show NN
4	CHEMBL217	Dopamine D2 receptor	Show NN
5	CHEMBL240	HERG	Show NN
6	CHEMBL222	Norepinephrine transporter	Show NN
7	CHEMBL3602	Sigma opioid receptor	Show NN
8	CHEMBL287	Sigma opioid receptor	Show NN
9	CHEMBL313	Serotonin transporter	Show NN
10	CHEMBL228	Serotonin transporter	Show NN
11	CHEMBL249	Neurokinin 1 receptor	Show NN
12	CHEMBL264	Histamine H3 receptor	Show NN
13	CHEMBL238	Dopamine transporter	Show NN
14	CHEMBL339	Dopamine D2 receptor	Show NN
15	CHEMBL237	Kappa opioid receptor	Show NN
16	CHEMBL4618	Leukotriene A4 hydrolase	Show NN
17	CHEMBL253	Cannabinoid CB2 receptor	Show NN
18	CHEMBL234	Dopamine D3 receptor	Show NN
19	CHEMBL236	Delta opioid receptor	Show NN
20	CHEMBL2014	Nociceptin receptor	Show NN

B)

Targets predicted using NN(Xfp) + NB(ECfp4). Save Table

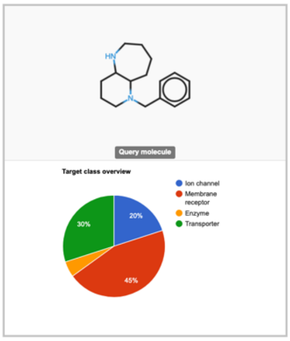

Rank	ChEMBL ID	Common name	Nearest neighbours
1	CHEMBL338	Dopamine transporter	Show NN
2	CHEMBL228	Serotonin transporter	Show NN
3	CHEMBL4618	Leukotriene A4 hydrolase	Show NN
4	CHEMBL3461	Neuronal acetylcholine receptor protein alpha-10 subunit	Show NN
5	CHEMBL287	Sigma opioid receptor	Show NN
6	CHEMBL222	Norepinephrine transporter	Show NN
7	CHEMBL313	Serotonin transporter	Show NN
8	CHEMBL238	Dopamine transporter	Show NN
9	CHEMBL264	Histamine H3 receptor	Show NN
10	CHEMBL322	Serotonin 2a (5-HT2a) receptor	Show NN
11	CHEMBL240	HERG	Show NN
12	CHEMBL4767	Vesicular acetylcholine transporter	Show NN
13	CHEMBL214	Serotonin 1a (5-HT1a) receptor	Show NN
14	CHEMBL265	Dopamine D1 receptor	Show NN
15	CHEMBL2492	Neuronal acetylcholine receptor protein alpha-7 subunit	Show NN
16	CHEMBL211	Muscarinic acetylcholine receptor M2	Show NN
17	CHEMBL245	Muscarinic acetylcholine receptor M3	Show NN
18	CHEMBL231	Histamine H1 receptor	Show NN
19	CHEMBL3602	Sigma opioid receptor	Show NN
20	CHEMBL4980	Neuronal acetylcholine receptor protein alpha-7 subunit	Show NN

Figure S7. PPB2 target prediction for **27a**/**27b**. **A)** Morgan fingerprint ECFP4. **B)** pharmacophore fingerprint XFP and ECFP4.[3]

6.7 Figure S8

A)

Targets predicted using NN(ECfp4) + NB(ECfp4). Save Table

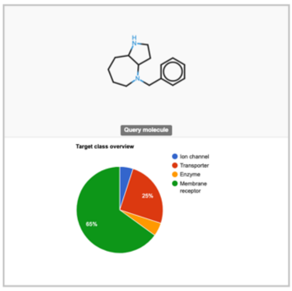

Rank	ChEMBL ID	Common name	Nearest neighbours
1	CHEMBL233	Mu opioid receptor	Show NN
2	CHEMBL287	Sigma opioid receptor	Show NN
3	CHEMBL338	Dopamine transporter	Show NN
4	CHEMBL3602	Sigma opioid receptor	Show NN
5	CHEMBL217	Dopamine D2 receptor	Show NN
6	CHEMBL313	Serotonin transporter	Show NN
7	CHEMBL284	Dipeptidyl peptidase IV	Show NN
8	CHEMBL237	Kappa opioid receptor	Show NN
9	CHEMBL222	Norepinephrine transporter	Show NN
10	CHEMBL339	Dopamine D2 receptor	Show NN
11	CHEMBL240	HERG	Show NN
12	CHEMBL228	Serotonin transporter	Show NN
13	CHEMBL225	Serotonin 2c (5-HT2c) receptor	Show NN
14	CHEMBL234	Dopamine D3 receptor	Show NN
15	CHEMBL238	Dopamine transporter	Show NN
16	CHEMBL249	Neurokinin 1 receptor	Show NN
17	CHEMBL253	Cannabinoid CB2 receptor	Show NN
18	CHEMBL224	Serotonin 2a (5-HT2a) receptor	Show NN
19	CHEMBL236	Delta opioid receptor	Show NN
20	CHEMBL264	Histamine H3 receptor	Show NN

B)

Targets predicted using NN(Xfp) + NB(ECfp4). Save Table

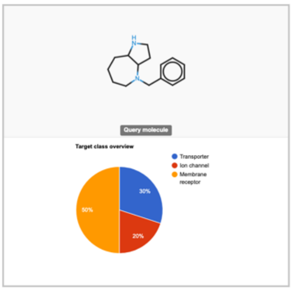

Rank	ChEMBL ID	Common name	Nearest neighbours
1	CHEMBL287	Sigma opioid receptor	Show NN
2	CHEMBL264	Histamine H3 receptor	Show NN
3	CHEMBL338	Dopamine transporter	Show NN
4	CHEMBL3602	Sigma opioid receptor	Show NN
5	CHEMBL313	Serotonin transporter	Show NN
6	CHEMBL322	Serotonin 2a (5-HT2a) receptor	Show NN
7	CHEMBL3461	Neuronal acetylcholine receptor protein alpha-10 subunit	Show NN
8	CHEMBL4767	Vesicular acetylcholine transporter	Show NN
9	CHEMBL214	Serotonin 1a (5-HT1a) receptor	Show NN
10	CHEMBL4980	Neuronal acetylcholine receptor protein alpha-7 subunit	Show NN
11	CHEMBL228	Serotonin transporter	Show NN
12	CHEMBL240	HERG	Show NN
13	CHEMBL233	Mu opioid receptor	Show NN
14	CHEMBL304	Norepinephrine transporter	Show NN
15	CHEMBL339	Dopamine D2 receptor	Show NN
16	CHEMBL2492	Neuronal acetylcholine receptor protein alpha-7 subunit	Show NN
17	CHEMBL222	Norepinephrine transporter	Show NN
18	CHEMBL270	Mu opioid receptor	Show NN
19	CHEMBL245	Muscarinic acetylcholine receptor M3	Show NN
20	CHEMBL3155	Serotonin 7 (5-HT7) receptor	Show NN

Figure S8. PPB2 target prediction for **1a**/**1b**. **A)** Morgan fingerprint ECFP4. **B)** pharmacophore fingerprint XFP and ECFP4.[3]

6.8 Figure S9

A)

Targets predicted using NN(ECfp4) + NB(ECfp4). Save Table

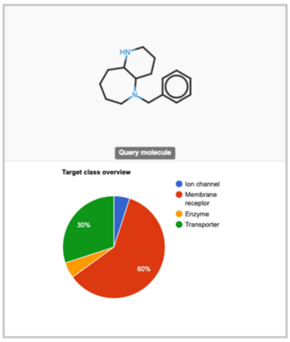

Rank	ChEMBL ID	Common name	Nearest neighbours
1	CHEMBL3602	Sigma opioid receptor	Show NN
2	CHEMBL338	Dopamine transporter	Show NN
3	CHEMBL233	Mu opioid receptor	Show NN
4	CHEMBL339	Dopamine D2 receptor	Show NN
5	CHEMBL237	Kappa opioid receptor	Show NN
6	CHEMBL287	Sigma opioid receptor	Show NN
7	CHEMBL238	Dopamine transporter	Show NN
8	CHEMBL228	Serotonin transporter	Show NN
9	CHEMBL264	Histamine H3 receptor	Show NN
10	CHEMBL240	HERG	Show NN
11	CHEMBL2014	Nociceptin receptor	Show NN
12	CHEMBL249	Neurokinin 1 receptor	Show NN
13	CHEMBL236	Delta opioid receptor	Show NN
14	CHEMBL4618	Leukotriene A4 hydrolase	Show NN
15	CHEMBL222	Norepinephrine transporter	Show NN
16	CHEMBL217	Dopamine D2 receptor	Show NN
17	CHEMBL234	Dopamine D3 receptor	Show NN
18	CHEMBL304	Norepinephrine transporter	Show NN
19	CHEMBL4767	Vesicular acetylcholine transporter	Show NN
20	CHEMBL4124	Histamine H3 receptor	Show NN

B)

Targets predicted using NN(Xfp) + NB(ECfp4). Save Table

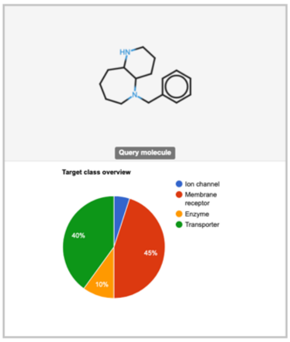

Rank	ChEMBL ID	Common name	Nearest neighbours
1	CHEMBL3602	Sigma opioid receptor	Show NN
2	CHEMBL264	Histamine H3 receptor	Show NN
3	CHEMBL338	Dopamine transporter	Show NN
4	CHEMBL287	Sigma opioid receptor	Show NN
5	CHEMBL238	Dopamine transporter	Show NN
6	CHEMBL222	Norepinephrine transporter	Show NN
7	CHEMBL339	Dopamine D2 receptor	Show NN
8	CHEMBL4124	Histamine H3 receptor	Show NN
9	CHEMBL4767	Vesicular acetylcholine transporter	Show NN
10	CHEMBL228	Serotonin transporter	Show NN
11	CHEMBL4828	Synaptic vesicular amine transporter	Show NN
12	CHEMBL2335	Lysosomal Pro-X carboxypeptidase	Show NN
13	CHEMBL313	Serotonin transporter	Show NN
14	CHEMBL304	Norepinephrine transporter	Show NN
15	CHEMBL401	Glutamate [NMDA] receptor subunit epsilon 3	Show NN
16	CHEMBL211	Muscarinic acetylcholine receptor M2	Show NN
17	CHEMBL315	Alpha-1b adrenergic receptor	Show NN
18	CHEMBL3199	Acetylcholinesterase	Show NN
19	CHEMBL265	Dopamine D1 receptor	Show NN
20	CHEMBL1916	Alpha-2c adrenergic receptor	Show NN

Figure S9. PPB2 target prediction for **40a/40b**. **A)** Morgan fingerprint ECFP4. **B)** pharmacophore fingerprint XFP and ECFP4.[3]

6.9 Figure S10

A)

Targets predicted using NN(ECfp4) + NB(ECfp4). Save Table

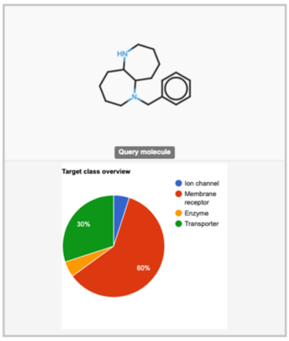

Rank	ChEMBL ID	Common name	Nearest neighbours
1	CHEMBL338	Dopamine transporter	Show NN
2	CHEMBL3602	Sigma opioid receptor	Show NN
3	CHEMBL233	Mu opioid receptor	Show NN
4	CHEMBL238	Dopamine transporter	Show NN
5	CHEMBL339	Dopamine D2 receptor	Show NN
6	CHEMBL228	Serotonin transporter	Show NN
7	CHEMBL264	Histamine H3 receptor	Show NN
8	CHEMBL237	Kappa opioid receptor	Show NN
9	CHEMBL287	Sigma opioid receptor	Show NN
10	CHEMBL2014	Nociceptin receptor	Show NN
11	CHEMBL222	Norepinephrine transporter	Show NN
12	CHEMBL4618	Leukotriene A4 hydrolase	Show NN
13	CHEMBL240	HERG	Show NN
14	CHEMBL217	Dopamine D2 receptor	Show NN
15	CHEMBL234	Dopamine D3 receptor	Show NN
16	CHEMBL304	Norepinephrine transporter	Show NN
17	CHEMBL236	Delta opioid receptor	Show NN
18	CHEMBL249	Neurokinin 1 receptor	Show NN
19	CHEMBL313	Serotonin transporter	Show NN
20	CHEMBL4124	Histamine H3 receptor	Show NN

B)

Targets predicted using NN(Xfp) + NB(ECfp4). Save Table

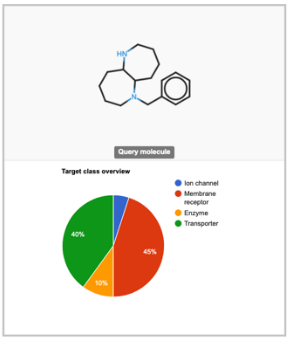

Rank	ChEMBL ID	Common name	Nearest neighbours
1	CHEMBL264	Histamine H3 receptor	Show NN
2	CHEMBL287	Sigma opioid receptor	Show NN
3	CHEMBL3602	Sigma opioid receptor	Show NN
4	CHEMBL338	Dopamine transporter	Show NN
5	CHEMBL238	Dopamine transporter	Show NN
6	CHEMBL222	Norepinephrine transporter	Show NN
7	CHEMBL313	Serotonin transporter	Show NN
8	CHEMBL4767	Vesicular acetylcholine transporter	Show NN
9	CHEMBL339	Dopamine D2 receptor	Show NN
10	CHEMBL4828	Synaptic vesicular amine transporter	Show NN
11	CHEMBL265	Dopamine D1 receptor	Show NN
12	CHEMBL228	Serotonin transporter	Show NN
13	CHEMBL4124	Histamine H3 receptor	Show NN
14	CHEMBL304	Norepinephrine transporter	Show NN
15	CHEMBL401	Glutamate [NMDA] receptor subunit epsilon 3	Show NN
16	CHEMBL2335	Lysosomal Pro-X carboxypeptidase	Show NN
17	CHEMBL315	Alpha-1b adrenergic receptor	Show NN
18	CHEMBL3199	Acetylcholinesterase	Show NN
19	CHEMBL211	Muscarinic acetylcholine receptor M2	Show NN
20	CHEMBL233	Mu opioid receptor	Show NN

Figure S10. PPB2 target prediction for **44b**. **A)** Morgan fingerprint ECFP4. **B)** pharmacophore fingerprint XFP and ECFP4.[3]

8 Biochemical assay procedures

In Vitro Pharmacology: Binding Assays.

Experiments were performed at Eurofins Cerep, Le Bois L'Evêque, B.P. 30001, 86600 Celle l'Evescault, France. Compound binding was calculated as a % inhibition of the binding of a radioactively labeled ligand specific for each target. The results are expressed as a percent of control specific binding:

$$\frac{measured\ specific\ binding}{control\ specific\ binding} * 100$$

As a percent inhibition of control specific binding:

$$100 - \left(\frac{measured\ specific\ binding}{control\ specific\ binding}\right) * 100$$

Experimental conditions:

Dopamine transporter *(h)* (antagonist radioligand):[4] Source: human recombinant (CHO cells), Ligand: [^{3}H]BTCP, Conc.: 4 nM, Kd: 4.5 nM, Non Specific: BTCP (10 μM), Incubation: 120 min, 4 °C, Detection Method: scintillation counting.

Norepinephrine transporter *(h)* (antagonist radioligand):[5] Source: human recombinant (CHO cells), Ligand: [^{3}H]nisoxetine, Conc.: 1 nM, Kd: 2.9 nM Non Specific: desipramine (1 μM), Incubation: 120 min, 4 °C, Detection Method: scintillation counting.

Serotonin transporter *(h)* (antagonist radioligand):[6], Source: human recombinant (CHO cells), Ligand: [^{3}H]imipramine, Conc.: 2 nM, Kd: 1.7 nM, Non Specific: imipramine (10 μM), Incubation: 60 min, RT, Detection Method: scintillation counting.

Sigma (non-selective) (antagonist radioligand): [7], Source: human jurkat cells, Ligand: [^{3}H]Haloperidol, Conc.: 8 nM, Kd: 5.8 nM, Non Specific: Haloperidol (10 μM), Incubation: 240 min, 25 °C. Detection Method: scintillation counting.

In Vitro Pharmacology: IC_{50} Determination. Compound binding was calculated as a % inhibition of the binding of a ligand specific for each target. The IC_{50} values (concentration causing a half-maximal inhibition of control specific binding) and Hill coefficients (nH) were

determined by non-linear regression analysis of the competition curves generated with mean replicate values using Hill equation curve fitting:

$$Y = D + \left[\frac{A - D}{1 + (\frac{C}{C_{50}})^{nH}}\right]$$

where Y = specific binding, A = left asymptote of the curve, D = right asymptote of the curve, C = compound concentration, C_{50} = IC_{50}, and nH = slope factor. This analysis was performed using software developed at Cerep (Hill software) and validated by comparison with data generated by the commercial software SigmaPlot® 4.0 for Windows® (© 1997 by SPSS Inc.). The inhibition constants (K_i) were calculated using the Cheng Prusoff equation:

$$K_i = \frac{IC_{50}}{1 + \frac{L}{K_D}}$$

where L = concentration of ligand in the assay, and K_D = affinity of the ligand for the receptor.

In Vitro Pharmacology: SafetyScreen44 Panel. For detailed experimental conditions of all targets see SafetyScreen44 Panel, Cerep.[8]

In Vitro Pharmacology: Intrinsic Clearance. Intrinsic Clearance (microsomes, S9, cryopreserved hepatocytes, recombinant CYP, recombinant UGT). Metabolic stability, expressed as percent of the parent compound remaining, was calculated by comparing the peak area of the compound at the time point relative to that at time-0. The half-life ($T_{1/2}$) was estimated from the slope of the initial linear range of the logarithmic curve of compound remaining (%) vs. time, assuming the first-order kinetics. The apparent intrinsic clearance (CL_{int}, in µL/min/pmol, µL/min/mg or µL/min/Mcell) was calculated according to the following formula:

$$CL_{int} = \frac{0.693}{T_{1/2} * (\frac{\text{mg protein}}{\mu L} \text{ or } \frac{\text{million cells}}{\mu L} \text{ or } \frac{\text{pmol CYP isozyme}}{\mu L})}$$

Experimental conditions:[9] Intrinsic clearance (liver, microsomes-human), Source: human liver microsomes (0.1 mg/mL), Substrate: Test compound, Incubation: 0, 15, 30, 45, 60 min, 37 °C, Measured component: Test compound, Detection Method: HPLC-MS/MS.

In Vitro Pharmacology: Protein Binding. The peak areas of the test compound in the buffer and test samples were used to calculate percent binding and recovery according to the following formulas:

$$\text{Protein binding (\%)} = \frac{\text{Area}_\text{p} - \text{Area}_\text{b}}{\text{Area}_\text{p}} * 100$$

$$\text{Recovery (\%)} = \frac{\text{Area}_\text{p} + \text{Area}_\text{b}}{\text{Area}_\text{c}} * 100$$

where Area_p = Peak area of analyte in protein matrix, Area_b = Peak area of analyte in buffer Area_c = Peak area of analyte in control sample.

Experimental conditions: Protein binding (plasma, human): Technique: Equilibrium dialysis, Incubation: 4 h, 37 °C, Detection Method: HPLC-MS/MS.

9 Biological assays

.

9.1 Figure S11

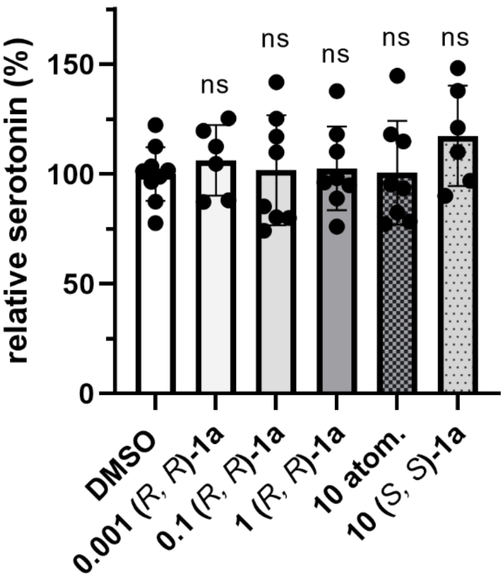

Figure S11. Serotonin (5-HT) uptake assay in PC-12 cells treated with vehicle (DMSO), **1, 100 or 1000 nM** (*R, R*)-**1a,** 10 µM atomoxetine (atom.) or 10 µM enantiomer *(S, S)*-**1a**. Ordinary one-way analysis of variance (ANOVA) followed by Tukey's multiple-comparison test. ns, not significant, **P* < 0.05, ***P* < 0.01 and ****P* < 0.001. Data were expressed as means ± SD.

Mice Behavioural Assays

9.2 Figure S12

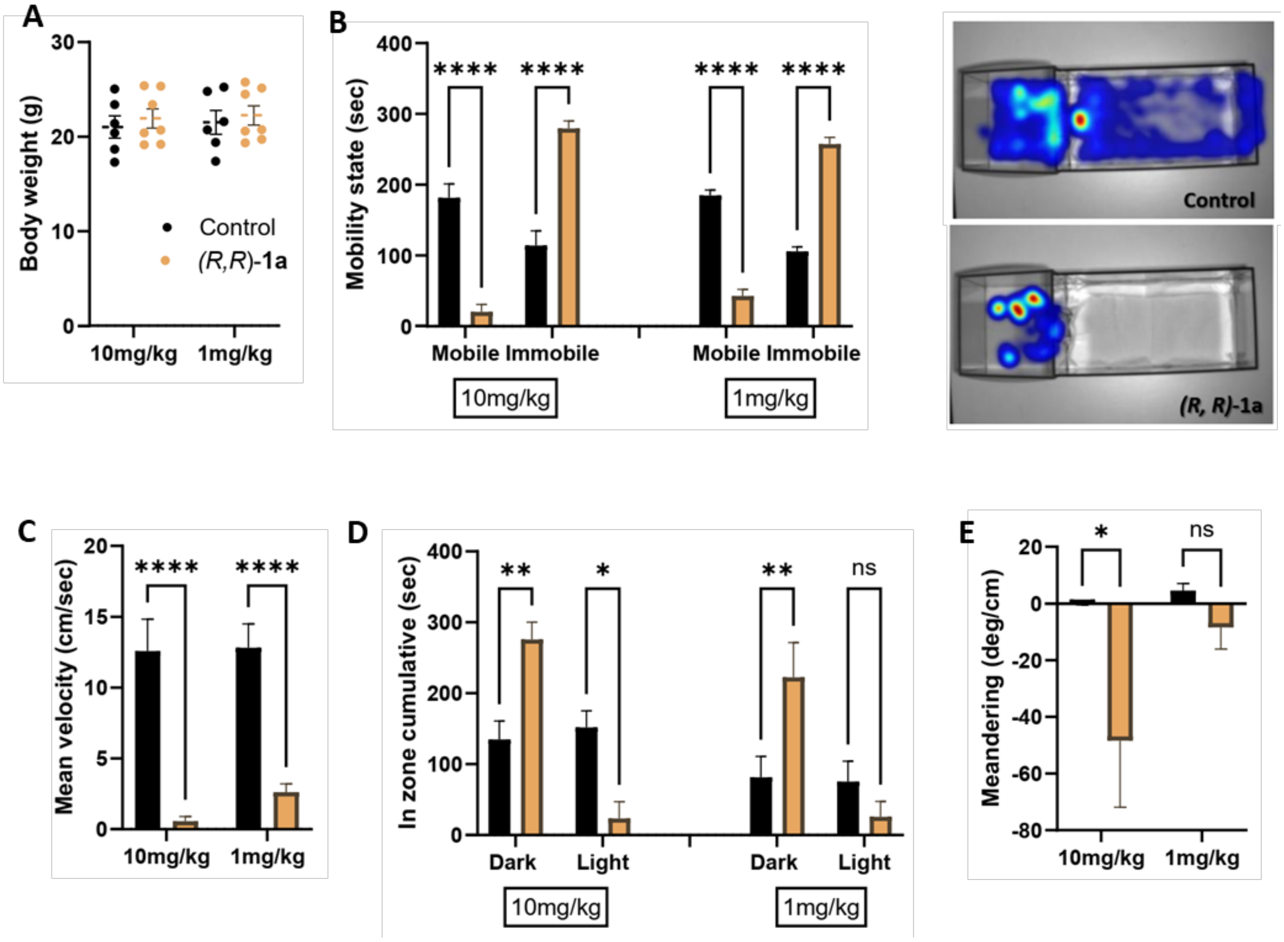

Figure S12. Behavioural examination following acute administration of two doses of *(R, R)*-**1a** in mice. **A)** Body weight of adult male mice treated acutely with 10 and 1 mg/kg (*R, R*)-**1a** **(n=7)**, and control vehicle treated animals (n=6). Behavioural data captured during the light/dark box task: **B)** Mobility. **C)** Mean Velocity. **D)** Time spent in dark and light compartments. **E)** Meandering. Results displayed as means ± SEM. P<0.05*, P<0.01**, P<0.001***, P<0.0001****.

9.3 Figure S13

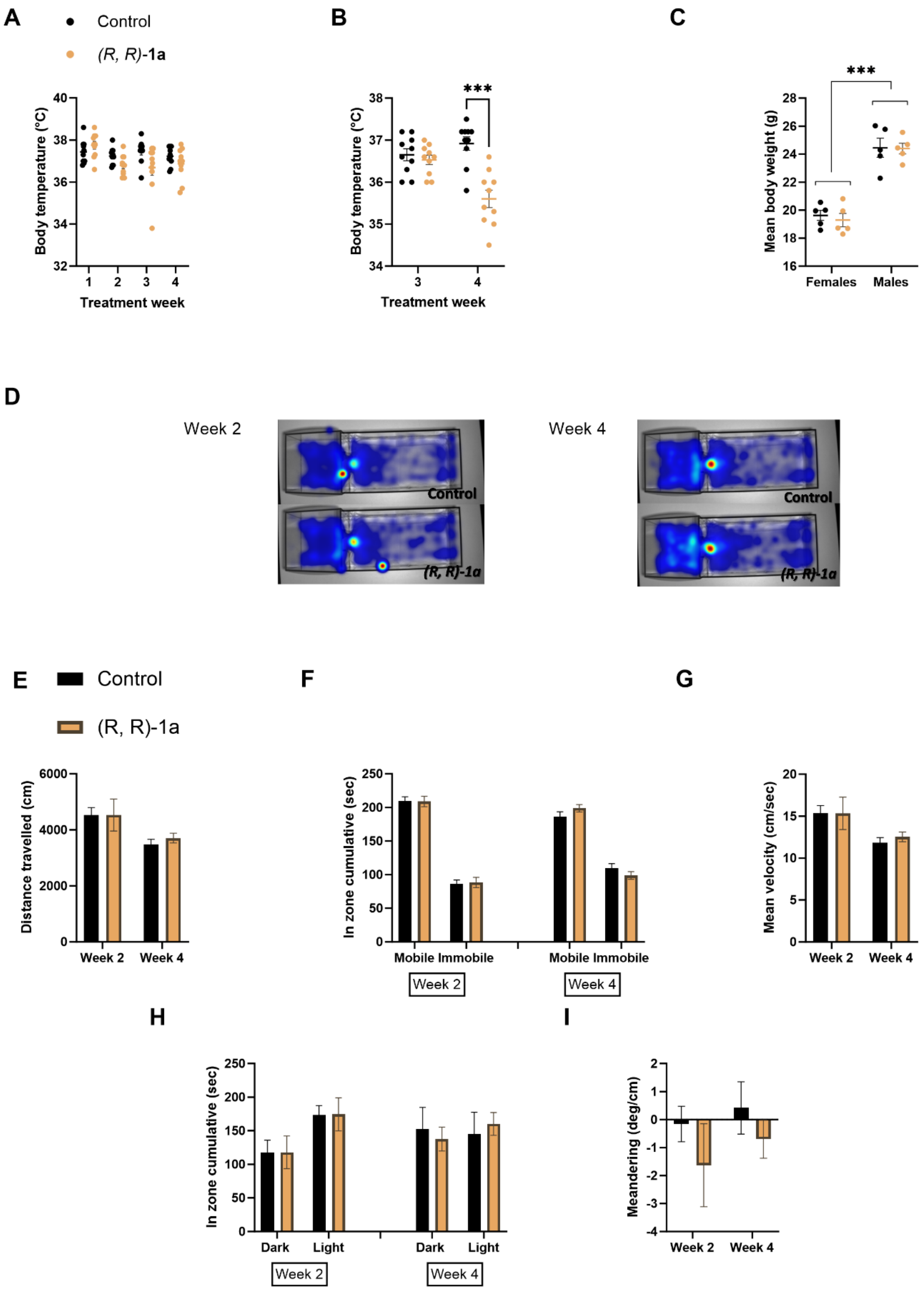

Figure S13. Behavioural examination during a 4-week chronic administration of 0.5 mg/kg *(R, R)*-**1a** in mice (5 males and 5 females/treatment group). **A)** Body temperature. **B)** Body temperature in response to acute administration of 1.0- or 10mg/kg *(R, R)*-**1a** on days 19 and 26 (Week 3 and 4), respectively. **C)** Mean body weight.

D) Representative heatmaps of movement in the respective groups from the light/dark box sessions on days 9 (Week 2) and 24 (Week 4). Behavioural data captured during the light/dark box task (E-I). **E)** Distance travelled. **F)** Mobility. **G)** Velocity. **H)** Time spent in dark and light compartments. **I)** Meandering. Results displayed as means ± SEM. P<0.005*, P<0.001**, P<0.0001***.

9.4 Figure S14

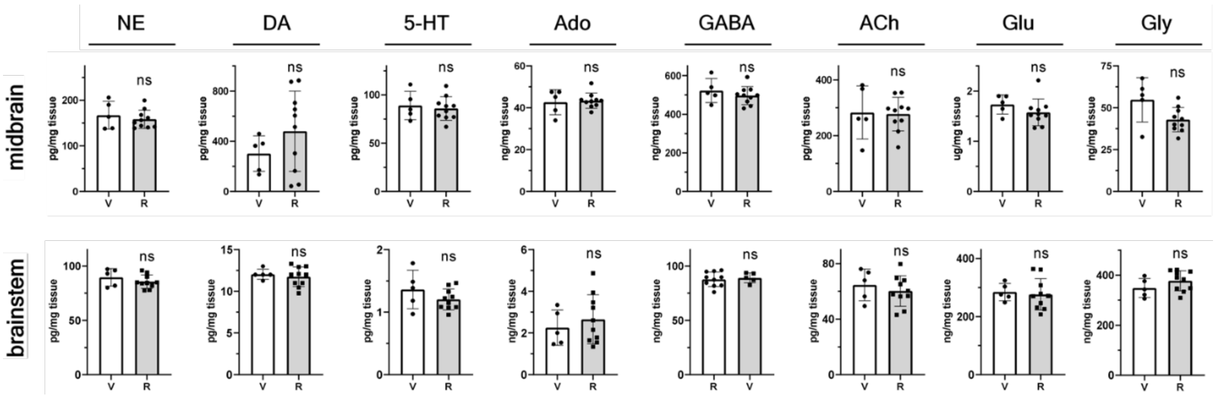

Figure S14. Quantification of neurotransmitter levels in brain regions in mice sacrificed after chronic administration of vehicle control (V) or (*R, R*)-**1a** (R).

9.5 Table S3

Table S3. LLC/MS gradient used for neurotransmitter quantification. Mobile phases were: 5mM Ammonium Formate in Water +0.1%FA, (Mobile Phase A) and 5mM ammonium formate in methanol + 0.1%FA (Mobile Phase B). Flow rate of 0.6ml/min with this gradient

Time (min)	% Aqueous Phase A	% Organic Phase B
0	80	20
1.50	80	20
4.00	5	95
6.00	5	95
6.10	80	20
8.00	80	20

9.6 Table S4

Table S4. Multiple reaction monitoring (MRM) transitions used for quantification of analytes.

**Analyte**	**Precursor ion (m/z)**	**Product Ion (m/z)**	**Polarity**	**DP**	**CE**	**CXP**
(*R*,*R*)-**1a** (syn 5-7-diamine)_1	231.3	91.1	Positive	60	44	13
"	231.3	124.1	Positive	60	26	9
**IS-(*R*,*R*)-ACA481 (2-chloro)**	265.2	124.2	Positive	80	29	7

9.7 Figure S15

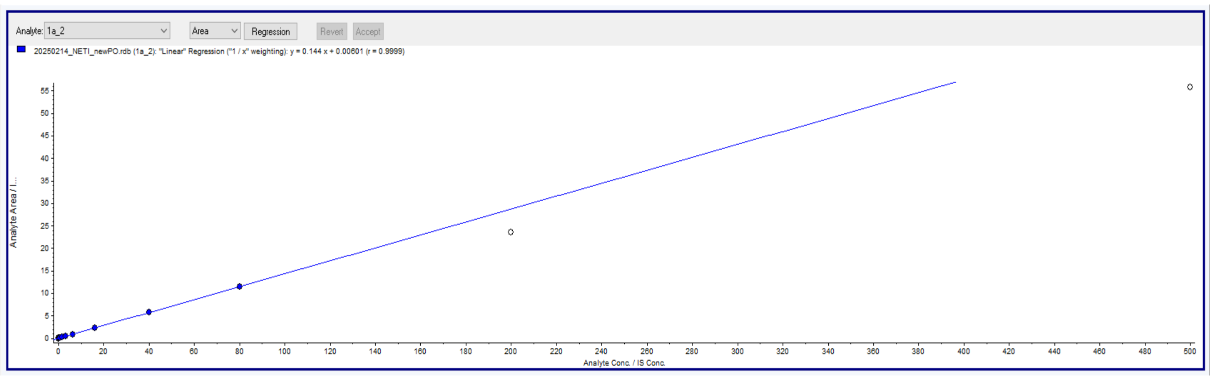

Figure S15. (*R*,*R*)-**1a** (syn 5-7- diamine) calibration curve, linear regression, 1/x weighting, R = 0.9999. y = 0.144x + 0.0601

10 References

(1) *Magic Formulas*. http://www.chem.rochester.edu/notvoodoo/pages/magic_formulas.php?page=aluminum_hydride_reduction (accessed 2022-11-04).

(2) Vranesic, I.; Ofner, S.; Flor, P. J.; Bilbe, G.; Bouhelal, R.; Enz, A. AFQ056/Mavoglurant, a Novel Clinically Effective mGluR5 Antagonist: Identification, SAR and Pharmacological Characterization. *Bioorg Med Chem* **2014**, *22*, 5790–5803. https://doi.org/10.1016/j.bmc.2014.09.033.

(3) Awale, M.; Reymond, J. L. Atom Pair 2D-Fingerprints Perceive 3D-Molecular Shape and Pharmacophores for Very Fast Virtual Screening of ZINC and GDB-17. *J Chem Inf Model* **2014**, *54*, 1892–1897. https://doi.org/10.1021/ci500232g.

(4) Pristupa, ZB.; Wilson, JM.; Hoffman, BJ.; Kish, SJ.; Niznik, HB. Pharmacological Heterogeneity of the Cloned and Native Human Dopamine Transporter: Disassociation of [3H]WIN 35,428 and [3H]GBR 12,935 Binding. *Mol Pharmacol* **1994**, No. 45, 125.

(5) Pacholczyk, T.; Blakely, R. D.; Amara, S. G. Expression Cloning of a Cocaine- and Antidepressant-Sensitive Human Noradrenaline Transporter. *Nature* **1991**, *350*, 350–354.

(6) Tatsumi, M.; Jansen, K.; Blakely, R.D.; Richelson, E. Pharmacological Profile of Neuroleptics at Human Monoamine Transporters. *Eur J Pharmacol* **1999**, *368*, 277–283.

(7) Ganapathy ME.; Prasad, PD.; Huang W.; Seth, P.; Leibach, FH.; Ganapathy V. Molecular and Ligand-Binding Characterization of the σ-Receptor in the Jurkat Human T Lymphocyte Cell Line. *J Pharmacol Exp Ther* **1999**, No. 289(1), 251–260.

(8) *SafetyScreen44 Panel, Cerep*. https://www.eurofinsdiscoveryservices.com/catalogmanagement/viewItem/SafetyScreen44-Panel-Cerep/P270 (accessed 2022-12-01).

(9) Obach, R. S.; Baxter, J. G.; Liston, T. E.; Silber, B. M; Jones, B. C.; MacIntyre, F.; Rance, D. J.; Wastall, P. The Prediction of Human Pharmacokinetic Parameters from Preclinical and in Vitro Metabolism Data. *J Pharmacol Exp Ther* **1997**, No. 283, 46–58.
